# Genome-wide analysis of the SPL/miR156 module and its interaction with the AP2/miR172 unit in barley

**DOI:** 10.1038/s41598-018-25349-0

**Published:** 2018-05-04

**Authors:** Rajiv K. Tripathi, Phil Bregitzer, Jaswinder Singh

**Affiliations:** 1 Plant Science Department, 21111 Rue Lakeshore, McGill University, Quebec, H9X 3V9 Canada; 2 USDA-ARS, National Small Grains Germplasm Research Facility, Aberdeen, ID 83210 USA

## Abstract

The *SQUAMOSA*-*promoter binding like* (*SPL*) gene family encodes transcription factors that have been shown in many species to influence plant growth and development, but information about these genes in barley (*Hordeum vulgare* L.) is limited. This study identified 17 barley *SPL* genes, within eight distinct groups, that are orthologs of *SPL* genes described in *Arabidopsis*, wheat, and rice. Sixteen barley *SPLs* undergo alternative splicing. Seven *SPLs* contain a putative miR156 target site and the transcript levels of the miR156-targeted *HvSPLs* (*HvSPL3*, *13* and *23*) were lower in vegetative than in reproductive phase but this was true also for some *SPL* genes such as *HvSPL6* that were not regulated by miR156. Because *SPL* gene products regulate miR172, which is also involved in floral development, the expression of miR172 was studied. An antagonistic expression pattern of miR156 and miR172b during the vegetative and the reproductive phases signifies their apparent function in barley growth phase transition. Characterization of a barley *mir172* mutant having an abnormal, indeterminate spikelet phenotype suggests the possible feedback role of AP2/miR172 module on *HvSPL* genes. This is the first comprehensive analysis of the miR156/SPL/miR172 axis in barley that provides a basis to elucidate their roles in various biological processes.

## Introduction

Barley (*Hordeum vulgare* L.) is a widely cultivated cereal grain. Cereal inflorescences are known as spikes. Each spike is composed of multiple spikelets formed directly on the main axis^1^. During domestication, both the yield and the architecture of cereal plants have been modified. Plant architecture and grain yield are complex traits that are encoded by many genes and regulatory factors. In the current study, our main research target was to explore the involvement of SPLs in barley growth phase transition from vegetative to reproductive stage. Transcription factors play important roles in plant growth and development by inducing or suppressing the expression of their target genes. The *SQUAMOSA* promoter binding like (SPL) protein family is one of the plant specific transcription factor families and each member shares a highly conserved 76 amino acid long DNA binding domain known as the SBP domain^2,3^. The SBP domain consists of three functionally important motifs, including two zinc-binding sites, Cys–Cys–Cys–His (Zn-1) and Cys–Cys– His–Cys (Zn-2), and a nuclear localization signal (NLS) located at the C-terminus of the domain^3,4^.

The first *SPL* gene was identified in *Antirrhinum majus*, and it controls flowering by binding to the promoter of *SQUAMOSA* (*SQUA*)^2^. Subsequently, multiple *SPL* genes were identified in *Arabidopsis thaliana*^5^, green algae (*Chlamydomonas*)^6,7^, moss^8^, silver birch^9^, tomato^10^, rice^11^, maize^12^, soybean^13^, wheat^14^ and cotton^15^. Studies have identified 16 *SPL* genes in *A. thaliana*^16,17^, 19 in rice^18^, and 28 in *Populus trichocarpa*^19^.

Studies of *A. thaliana* have shown that *SPL* genes have diverse functions in plant growth and development. Constitutive expression of *SPL3* produced very early flowering^20^. *SPL8* affected reproductive development through the genes involved in GA (gibberellic acid) biosynthesis^21,22^. *SPL2*, *SPL10* and *SPL11* have been associated with shoot maturation^23^. *SPL9* and *SPL13* controlled shoot development in *Arabidopsis*^24^. *SPL7* was identified as a central regulator of copper homeostasis^25^. The miR156-targeted *SPL9* promoted sesquiterpene biosynthesis by binding to the promoter region of *TPS21*^26^ and it negatively regulated anthocyanin levels by modulating the expression of the MYB-bHLH-WD40 complex^27^.

*SBP1* silencing in *A. majus* resulted in a late to non-flowering phenotype, and *SBP1-*mediated transition to flowering occurred due to the positive regulation of *FUL*/*LFY* meristem identity genes^28^. Paralogous *SBP1*, *SBP2* and *CNR* genes differentially controlled leaf initiation and reproductive phase transition in petunia^29^. *SPL* genes in monocot plants also have been shown to affect important developmental processes. In rice, overexpression of *SPL14* promoted panicle branching and higher grain yield^30^; *SPL16* regulates grain size, shape, and quality^31^; *SPL13* positively controlled grain weight, length, and thickness^32^; and the interaction of the SPL14 protein with human OTUB1 like deubiquitinase enhanced grain yield^33^. In bread wheat, it was found that the miR156-SPL module regulated bread wheat plant architecture by interacting with a strigolactone signalling repressor gene, *DWARF53*^34^. The maize SBP-box transcription factors *unbranched2* and *unbranched3* alters plant architecture and affect yield traits in maize^35^. Genetic modification of the miR156-SPL4 module controls aerial axillary bud formation, branching, biomass yield, and re-growth after cutting in switchgrass^36^.

MicroRNAs (miRNAs) are non-coding RNAs that can complementarily bind to target sites and repress expression via cleavage or repression of translation^37^. In *Arabidopsis*, 10 of the 16 *SPL* genes are targets of miR156^5,20^ and 11 of the 19 *SPL* genes in rice have been identified as a targets of miR156^18^. The miR156 complementary sites are present in the coding region or in the 3′ un-translated region (3′-UTR). In *A. thaliana*, two miRNAs, miR156 and miR172, regulated the juvenile to adult developmental phase change^38^; *SPL9* and *SPL10* promoted the expression of *miR172b* by binding to its promoter and acted independently of this and its target genes^38^; and the expression of miR156 was higher in the juvenile phase than in the adult phase, whereas the expression of miR172 was lower in the juvenile phase than in the adult phase^38^. The miR172 is known for the regulation of *AP2-*like transcription factors through transcript cleavage and translational repression in *Arabidopsis*^39,40^. Expression of miR172 promotes the vegetative phase change in maize by repressing an *AP2*-like gene *Glossy15*^41^. In barley, suppression of miR172 guided cleavage of *AP2* mRNA produces cleistogamous flowering^42^ and affects spikelet determinacy^43^. Perturbed interaction between *AP2* and miR172 leads to striking differences in the size and shape of the barley spike^44^. The characterization of *SPL* genes has not been conducted for barley as it has for *Arabidopsis* and rice. This is the first comprehensive study of *SPL* genes in barley and includes analysis of phylogeny, motif composition, gene structure, miRNA target site, alternative splicing events and spatio-temporal expression patterns. In addition, the expression patterns of *SPL* genes and of miR156 and miR172 from vegetative to reproductive phases revealed their possible functional relationships. The expression of *AP2* and *SPL* genes in the spikes of *mir172* mutants and its wild-type counterpart golden promise (GP) elucidated their involvement in spike development.

## Results

### Identification of *SPL* Genes in Barley

A total 17 putative *SPL* genes were identified in barley and were designated as *HvSPL*. In this study, *HvSPLs* were specified based on their similarity to wheat and rice orthologs (Table 1). Full length coding sequences of the *HvSPLs* ranged from 339 to 3393 bp (Table S1), and the deduced proteins ranged from 112 to 1130 amino acids (Table S2). The 17 *HvSPL* genes were unevenly distributed on chromosomes 1H, 2H, 3H, 5H, 6H, 7H and Un (Unnumbered) (Table 1).

### Phylogenetic Relationship of *SPL* Genes in Barley, *Arabidopsis*, Rice, and Wheat

**Figure 1:**
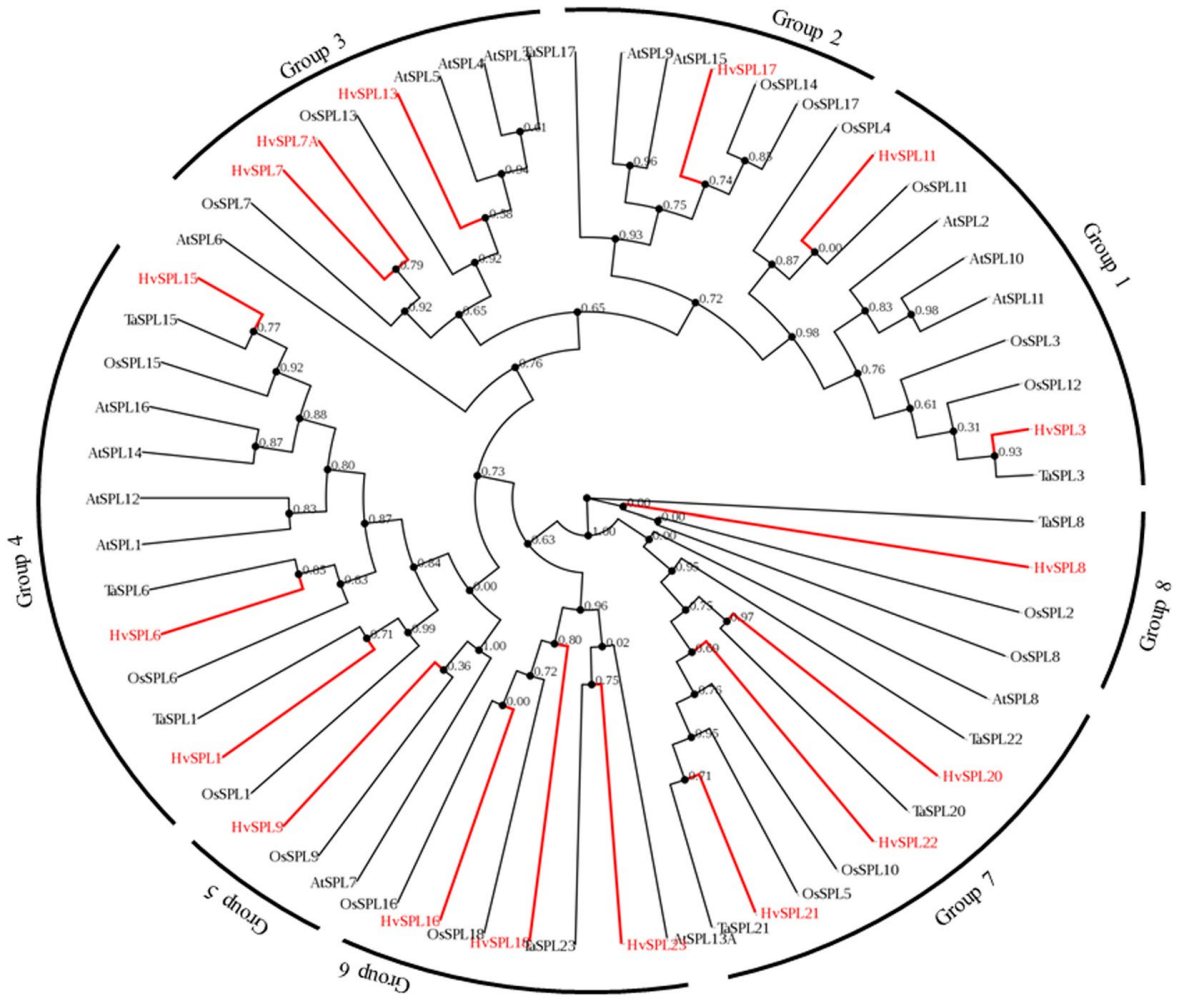
Phylogenetic analysis of SPL proteins based on their SBP domain sequences. The maximum likelihood phylogenetic tree of SPL proteins from *Arabidopsis thaliana* (AtSPL), rice (OsSPL), wheat (TaSPL) and barley (HvSPL) using PhyML3.0. HvSPLs are shown in red color.

A phylogenetic tree was constructed using the maximum likelihood method based on 61 SBP domains sequences from barley, rice, wheat, and *A. thaliana* (Fig. 1; Table S4). The SPL proteins were assigned to one of eight groups with one *Arabidopsis* protein (AtSPL6) outlier. As expected, HvSPL proteins were more closely related to those of wheat and rice than to those of *A. thaliana*. *HvSPLs* were present in all groups. Maximum numbers of *HvSPL* genes (9) were found in the same clades where *SPL* orthologs from wheat were grouped. Other *HvSPLs* were found to be more closely related to rice *SPL* orthologs. Interestingly, *HvSPL11* was orthologous to two rice *SPL* paralogs, *SPL11* and *SPL4* (Fig. 1). Rice *SPL7* was orthologous to two barley *SPL* paralogs, *SPL7* and *SPL7A*. Likewise, *HvSPL17* was orthologs to *OsSPL14* and *OsSPL17* genes.

### Structural Features and Conserved Motif Analysis of Barley *SPL* Gene**s**

Genetic structural diversity may enable the evolution of multi-gene families. To gain further insight into the structure of *HvSPL* genes, we compared the predicted numbers, lengths, and arrangements of introns and exons. At least one intron was present in all *HvSPL* genes except *HvSPL9*, and the number of exons ranged from 1 to 11 (Fig. S1A). We searched for conserved motif sequences present inside of SBP domain and in predicted HvSPL full length proteins, and their compositions and diversities were analysed. Analysis of the conserved domains of HvSPL proteins showed that barley SBP domain has two zinc binding motifs (Cys3His and Cys2HisCys) that include eight conserved cysteine and histidine residues (Fig. S1B). A putative NLS motif was identified at the C-terminus of the Cys2HisCys motif. HvSPL1, 6 and 15 also contained DEXDC and ANK (ankyrin) domain (Table 1). Further investigation discovered ten motif sequences that are less distantly diverged among their groups and their combinations were analysed (Fig. S2). Motifs 1, 2, 7 and 8 are involved in the makeup of the Zn-2, Zn-1, NLS (nuclear localization signal) and joint peptide of SBP domain, but the identities of other motifs are unknown. All predicted HvSPL proteins contain conserved motif combinations within their phylogenetic group, suggesting that the variable amino acids outside of the SBP domains are responsible for the diversity of SPLs in *A. thaliana*, barley, wheat, and rice.

**Table 1:** Characteristics of identified *SPL* genes in *H. vulgare*.

Gene^a^	Gene symbol^b^	CDS^c^ length (bp)	Domain^d^	Deduced protein (aa)^e^	Ch^f^	Position on genome^g^	Exon^h^
*HvSPL1*	HORVU7Hr1G042370	2526	SBP, DEXDC, ANK	841	7H	122553615–122558679	10
*HvSPL3*	HORVU6Hr1G019700	1524	SBP	507	6H	53909817–53916886	5
*HvSPL6*	HORVU5Hr1G117190	2895	SBP, DEXDC, ANK	964	5H	650559269–650565702	11
*HvSPL7*	HORVU2Hr1G097580	630	SBP	209	2H	679640052–679642809	2
*HvSPL7A*	HORVU2Hr1G097610	612	SBP	203	2H	679689970–679692277	2
*HvSPL 8*	HORVU0Hr1G039150	1089	SBP	362	Un	248118980–248122768	3
*HvSPL9*	HORVU1Hr1G060770	978	SBP	325	1H	441051831–441063137	1
*HvSPL11*	HORVU6Hr1G031450	981	SBP	326	6H	133169150–133173306	4
*HvSPL13*	HORVU2Hr1G048280	588	SBP	195	2H	269391017–269400061	3
*HvSPL15*	HORVU7Hr1G051400	3393	SBP,DEXDC, ANK	1130	7H	192879289–192885430	10
*HvSPL16*	HORVU5Hr1G076380	1179	SBP	392	5H	551053196–551057389	3
*HvSPL17*	HORVU5Hr1G073440	1251	SBP	416	5H	539014598–539018210	3
*HvSPL18*	HORVU0Hr1G039170	1203	SBP	400	Un	248141188–248147114	3
*HvSPL 20*	HORVU7Hr1G110980	1350	SBP	449	7H	631945657–631949862	3
*HvSPL21*	HORVU6Hr1G030490	1476	SBP	491	6H	127986525–127990179	3
*HvSPL 22*	HORVU7Hr1G110950	339	SBP	112	7H	631926981–631927880	1
*HvSPL23*	HORVU3Hr1G094730	1290	SBP	429	3H	647368957–647372642	3

^a^Name referred to *H. vulgare SPLs* in this work.

^b^Gene accession number in database.

^c^Length of coding DNA sequence.

^d^Domain predicted by SMART tool.

^e^Length (number of amino acids).

^f^Chromosome position of the *HvSPL* genes.

^g^Location of *HvSPL* genes on barley genome.

^h^Exon number in *HvSPL* genes.

### *Cis*-Regulatory Elements in *HvSPLs* Promoter Regions

Presence of *cis*-regulatory motifs was analysed using 1 Kb upstream genomic sequences of *HvSPL* genes. The identified *cis*-regulatory motifs were belonged to mainly four categories based on functional aptitude namely, light responsive elements, growth and development responsive elements, stress responsive and hormone responsive elements (Table 2). The 17 *SPL* genes were grouped according to phylogeny and *HvSPL9* from Group 5, *HvSPL17* from 2, and *HvSPL18* from group 6 were found to contain plant_AP-2-like regulatory motif. The light responsive elements, G-Box, sp1, GA-motif, GAG-motif, I-box, GC-motif, ACE motif, MNF1 and Box 1 were enriched in most of the genes. In case of growth and development responsive elements, AC-II, CCGTCC-box, ATGCAAAT motif, GCN4_motif, O2-site, CCGTCC-box, Skn-1_motif, circadian and plant_AP-2-like etc. were more abundant. Further, stress responsive elements like ARE, A-box MBS, LTR and HSE were responsive to various abiotic stresses whereas, Box W1 and W-box were reported in biotic stresses. Numerous *cis*-regualtory elements responsive to hormones were identified such as ERE and ABRE were ethylene and abscisic acid responsive elements; TGA element and AuxRE-core were reported as auxin responsive; GARE-motif, P-box and TATC-box as gibberellin responsive and CGTCA and TGACG motifs are related to MeJa response.

**Table 2:** *Cis*-regulatory elements predicted in promoter region of *HvSPL* genes.

Gene	Light Response	Growth and Development	Stress Response	Hormone Response
*HvSPL3*	Box II –like sequence, CATT-motif, GA-motif, GAG-motif,Sp1	5UTR Py-rich stretch, AC-II,CCGTCC-box, GCN4_motif, Skn-1_motif, circadian	A-box, ARE, HSE,MBS, TC-rich repeats, box E	CGTCA-motif, TCA-element, TGACG-motif
*HvSPL11*	ATC-motif, BoxI, GAG-motif, GC-motif, I-box,MNF1,Sp1	CAT-box, GCN4_motif, Skn-1_motif, circadian	ARE	CGTCA-motif, TCA-element, TGA-element, TGACG-motif
*HvSPL17*	ATCC-motif, Box I, CATT-motif, G-Box, GA-motif, GAG-motif, Gap-box,Sp1	HD-Zip 3,Skn-1_motif, plant_AP-2-like	ARE, HSE, TC-rich repeats	TGA-element
*HvSPL13*	ACE,ATCT-motif, Box 4,GA-motif, LAMP-element	ATGCAAAT motif, GCN4_motif, O2-site	Box-W1, HSE, W box	CGTCA-motif, EIRE, TGACG-motif
*HvSPL7*	ACE,CG-motif, G-box, GATA-motif, GC-motif, I-box,Sp1,box II	CCGTCC-box, RY-element, Skn-1_motif	A-box, LTR, box S	ABRE, CGTCA-motif, TATC-box, TGACG-motif
*HvSPL7A*	ATC-motif, ATCT-motif, G-Box, GA-motif, GAG-motif, GC-motif, Sp1,TCT-motif	Skn-1_motif	box S	ABRE
*HvSPL15*	ACE,G-Box, GC-motif,Sp1	AC-II,CAT-box, CCGTCC-box, O2-site,dOCT	A-box, ARE, MBS, TC-rich repeats	ABRE, motif Iib
*HvSPL6*	ACE, GC-motif, MNF1, Pc-CMA2c, Sp1,TCT-motif	CAT-box, CCAAT-box, CCGTCC-box, OCT	A-box, Box-W1, TCCACCT-motif, W box	CGTCA-motif, TGA-element, TGACG-motif
*HvSPL1*	ACE,AE-box, Box 4,G-box, GT1-motif,I-box,MRE,Sp1, TCT-motif	O2-site, Skn-1_motif, circadian	Box-W1, MBS, W box	AuxRE, TCA-element
*HvSPL9*	GC-motif, L-box, MNF1, Sp1	AC-I,AC-II, CAT-box, GCN4_motif,O2-site, Skn-1_motif, plant_AP-2-like	Not found	ABRE, CGTCA-motif, GARE-motif, TGACG-motif, motif IIb
*HvSPL16*	CATT-motif, G-Box,GAG-motif, I-box, SpI	5UTR Py-rich stretch, AC-I,CAT-box, Skn-1_motif	HSE, TCCACCT-motif	ABRE
*HvSPL18*	G-Box,GAG-motif, GT1-motif, I-box,Sp1, TCCC-motif	CCGTCC-box, GCN4_motif, HD-Zip 1,HD-Zip 2, RY-element, circadian, plant_AP-2-like	A-box, ARE, TC-rich repeats	ABRE
*HvSPL23*	ATCC-motif, G-box,GC-motif, MNF1,Sp1	5UTR Py-rich stretch, CCAAT-box, Skn-1_motif	Box-W1, LTR, TC-rich repeats, W box	GARE-motif, motif IIb
*HvSPL21*	ACE, Box I, GT1-motif, TCCC-motif	ATGCAAAT motif, GCN4_motif, Skn-1_motif, TA-rich region	TC-rich repeats	CGTCA-motif, ERE, TCA-element, TGACG-motif
*HvSPL 22*	Box II,G-Box, GC-motif, Sp1, TATCCAT/C-motif	CCAAT-box, CCGTCC-box, Skn-1_motif	A-box, Box-W1, HSE,W box	CGTCA-motif, TATC-box, TGA-element, TGACG-motif
*HvSPL 20*	C-box,G-box, GC-motif,Sp1, chs-Unit 1 m1	AC-II,CCGTCC-box, GCN4_motif, O2-site, Skn-1_motif	A-box, ARE, Box-W1, MBS, TCCACCT-motif, W box	ABRE, CGTCA-motif, GARE-motif, P-box,TGACG-motif
*HvSPL 8*	G-Box, GATA-motif, chs-CMA2a	Circadian	Box-W1,MBS, W box	TCA-element

### MiR156 Family in *H. vulgare* and Their Target Site in *HvSPL* Genes

**Figure 2:**
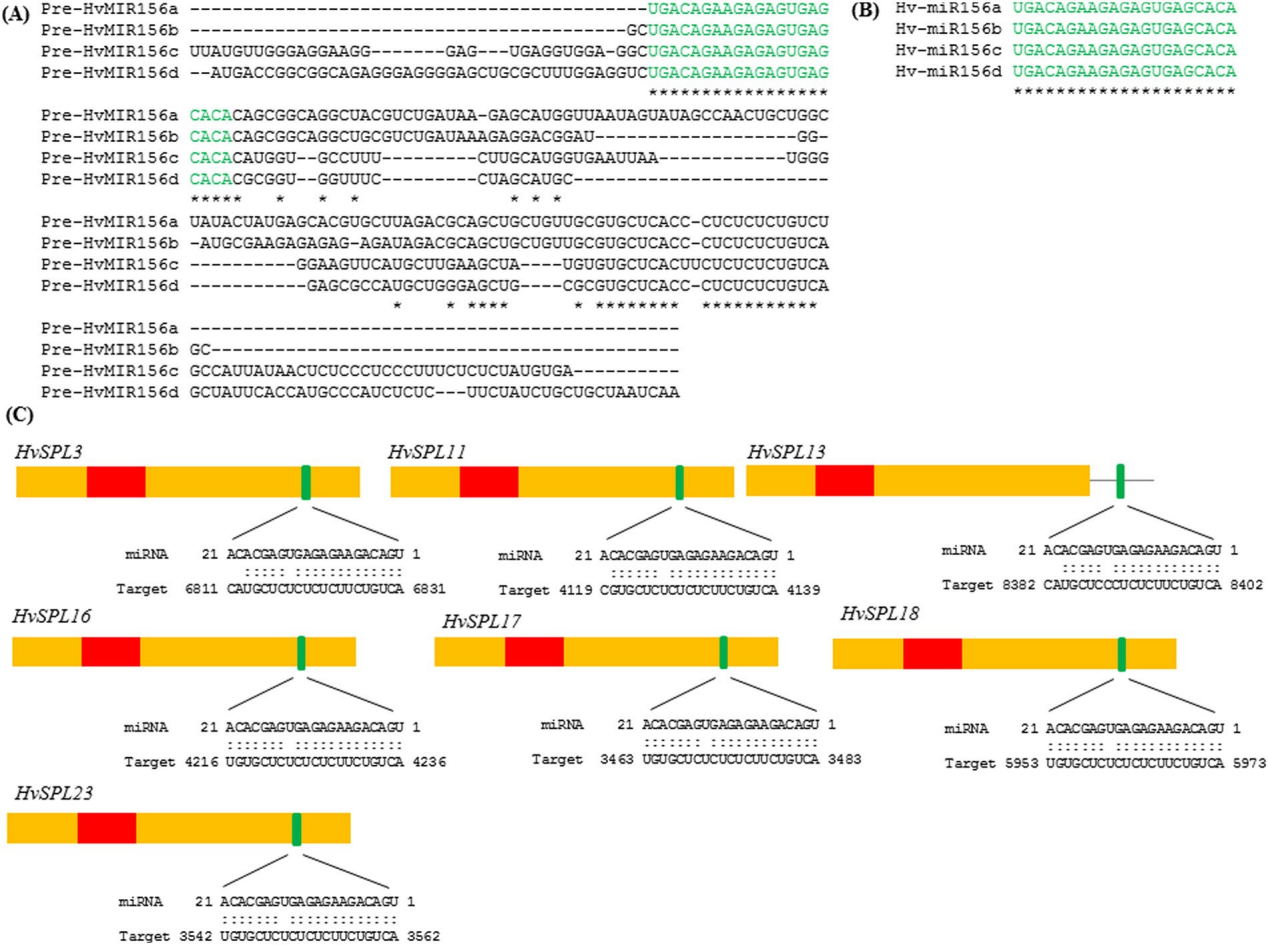
miR156 family members and their target site in Barley SPL genes. (**A**) Alignment of precursor sequences of four miR156 family members. (**B**) Alignment of mature sequences of four miR156 family members. Green colour denotes the mature sequence of miR156a/b/c and d. (**C**) miR156 target site in *HvSPL3, 11, 13, 16, 17, 18 & 23* genes. Yellow box represent CDS, red box SBP domain and line 3′UTR. The miR156 target sites with the nucleotide positions of *HvSPL* transcripts are shown in green. RNA sequence of each complementary site from 5′ to 3′ and the predicted miRNA sequence from 3′ to 5′ are indicated.

Previous studies have reported that a subset of *SPL* genes are regulated by miR156 in plant species including *A. thaliana*^17^, soybean^13^, rice^18^, poplar^19^, and maize^45^. Therefore, miR156 family in barley genome and its target site in *HvSPLs* was studied. Two putative members of miR156 family, Hv-miR156a (accession number MI0016449) and Hv-miR156b (accession number MI0030546) were identified for barley in the miRbase database (http://www.mirbase.org/cgibin/mirna_summary.pl?fam=MIPF0000008) and another two (Hv-miR156c and Hv-miR156d) were identified in the mirEX2.0 database (Fig. 2A, 2B). The mature miR156 sequences of all four members were identical, but divergence was observed in the precursor sequences which showed 71 to 87% homology. Putative miR156 binding sites were found for *HvSPL3, HvSPL11*, *HvSPL16*, *HvSPL17, HvSPL18* and *HvSPL23* in their coding regions and for *HvSPL13* in the 3′UTR (Fig. 2C; Table S5), suggesting that regulation by miR156 is restricted to this subset of *HvSPL* genes.

### Validation of Alternative Splicing Events in *HvSPL* Genes

**Figure 3:**
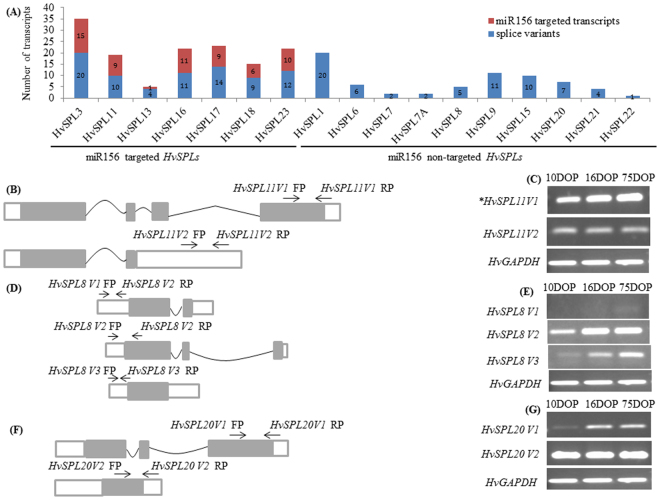
Alternative splicing event in *HvSPL* genes and expression analysis. (**A**) Number of Splice variants in *HvSPL* genes and miR156 target site distribution. (**B**,**C**) The splice variants of barley *SPL11* and their expression pattern in 10, 16 and 75 day old plant. The two variants (*SPL11*V1 and V2) expressed differentially. (**D**,**E**) Splice variant of barley *SPL8* and their expression patterns. The three variants (*SPL8*V1, V2 and V3) of *SPL8* expressed lower in juvenile and higher in reproductive phase. (**F**,**G**) Two splice variant (*SPL20*V1 and V2) of barley *SPL20* and their expression patterns. *Asterisks denote the presence of the miR156 complementary sequence in the splice variants of barley *SPL11* gene. The brown colour box represents coding region, black line denotes intron and the white rectangle denotes 5′ & 3′ UTR regions. The arrow shows the site of forward and reverse primers. For more clarity gel area showing relevant bands were cropped. The full-length gels are presented in Supplementary Fig. S3.

Multiple transcripts can be formed from one gene by selecting different splice sites during mRNA production. Therefore, various transcript isoforms may generate truncated proteins that could influence their stability levels, sub-cellular localizations, protein-protein interactions and other functions. The ensemble database predicted splice variants in 16 of 17 *HvSPL* genes, except *HvSPL22* (Fig. 3A; Table S6). All *HvSPLs* with miR156 binding sites were predicted to produce splice variants (4 to 20 numbers). Similarly, miR156 non-targeted (that is, lacking a miR156 binding site) *HvSPLs* also generated splice variants (1 to 20 numbers) of varying length. *HvSPL1* and 3 produced the highest numbers (20) of predicted splice variants. Interestingly, differences in the miR156 target site among splice variants were also observed. In case of *HvSPL3* (20 splice variants), *HvSPL11* (4 splice variants) and *HvSPL17* (14 splice variants), only 15, 1 and 9 number of splice variants contained miR156 target site.

To distinguish the major and minor splice variants and their differential expression patterns, several *HvSPLs (HvSPL8, HvSPL11*, and *HvSPL20*) were selected at random for analysis by semi-quantitative RT-PCR in 10, 16, and 75 days old (vegetative to reproductive phase) barley plants (Fig. 3B–G). Splice variant 1 of *HvSPL11* (*HvSPL11* V1), which contained a miR156 target site, showed lower expression at vegetative and higher at the reproductive phase and was the major transcript (Fig. 3B,C). However, expression remained constant for variant 2 of *HvSPL11* (*SPL11* V2), which lacked a miR156 target site and was a minor transcript. Similarly, differential expression of splice variants of *HvSPL8* and *HvSPL20* was observed at different developmental stages (Fig. 5D–G). Expression of *HvSPL8* V2 and *HvSPL8* V3 was lower in the vegetative phase but higher in the reproductive phase; these were the major transcripts. Expression of *HvSPL20* V1 was higher at the reproductive stage than in the vegetative stage. Expression of *HvSPL20* V2 was constant in all stages and was the key transcript for *HvSPL20*. These results suggest that splice variants of *HvSPLs* are produced and that they may contribute to the diversity of encoded proteins with the potential to play important roles at various stages of plant growth and development.

### Spatio-temporal Expression Pattern of *HvSPL* Genes

**Figure 4:**
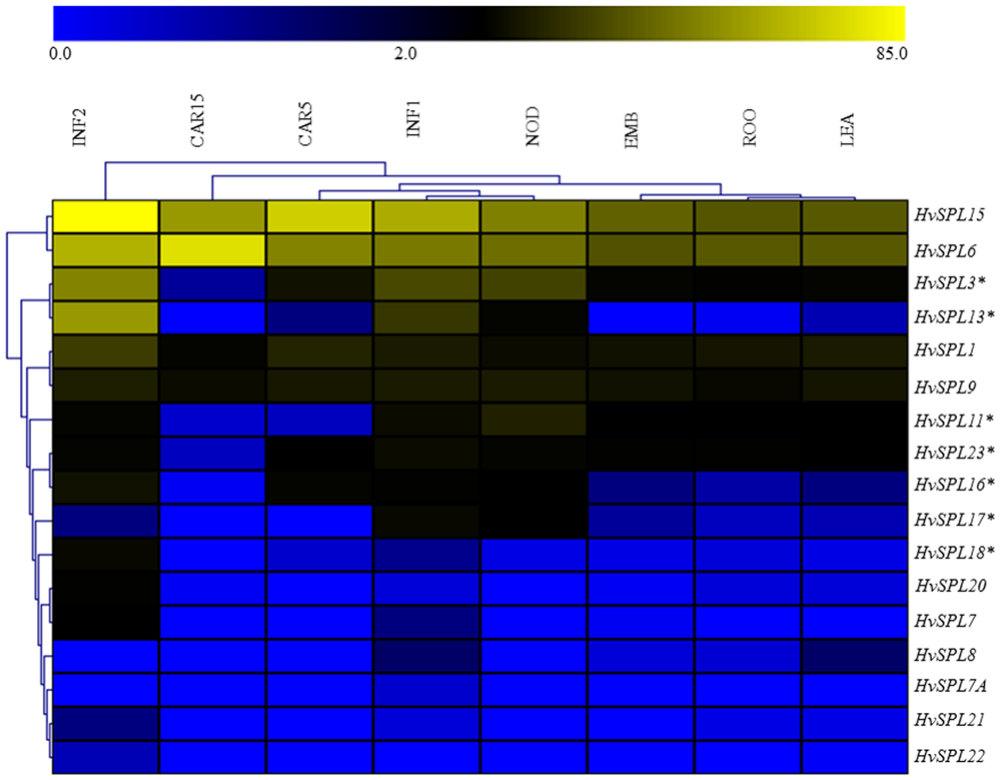
Spatio-temporal expression patterns of *HvSPL* genes in eight different tissues. The colour scale bar at the top of heat map represents FPKM normalized log2 transformed values based on “Morex” RNA-seq data, and represents high and low expression, respectively. EMB, ROO, NOD, LEA, INF1, INF2, CAR5 and CAR15tissues were used for expression profiling and indicated at the top of the heat map. Details about these tissues have been explained in material and method section. *HvSPL* genes that contain miR156 target sites are indicated by (*) *asterisks*.

In the absence of *HvSPL* mutants, the expression patterns of various *HvSPLs* may provide clues about their potential functions. We examined the spatio-temporal expression patterns of *HvSPLs* in eight tissues (4-day-old embryos (EMB), roots (Roo), shoots (LEA), developing inflorescences (INF1; 5 mm) and (INF2; 1–1.5 cm), developing tillers internodes (NOD), developing grain 5 days post anthesis (DPA), developing grain with bracts removed at 5 DPA (CAR5) and 15 DPA (CAR15)) of barley via hierarchical clustering analysis (Fig. 4). Nine *HvSPLs* (*HvSPL1, 3, 6, 11, 13, 15, 16, 17* and *23*), including six that are targeted by miR156 (*HvSPL3, 11, 13, 16, 17* and *23*) were highly expressed and displayed tissue-specific patterns of expression. Interestingly, expression of miR156 targeted *HvSPL18* and miR156 non-targeted *HvSPL7* and *HvSPL20* were unique to INF2 tissue. In contrast, the others (*HvSPL7A, 8, 21*, and *22*) showed very low expression in all tissues. Most of the *HvSPLs* genes (except *HvSPL8* and *21*) were highly expressed in inflorescence 2, whereas expression of only *HvSPL17, 20, 21* and *22*, was higher in inflorescence 1, suggesting their involvement in barley inflorescence development. *HvSPL13* was expressed mainly in the inflorescence, and its expression at the NOD and CAR5 stages was negligible. *HvSPL6* and *HvSPL15* were constitutively expressed at high levels in all tissues.

### Vegetative to Reproductive Phase in Barley: Expression of miR156, miR172 and Specific *SPL* Genes

**Figure 5:**
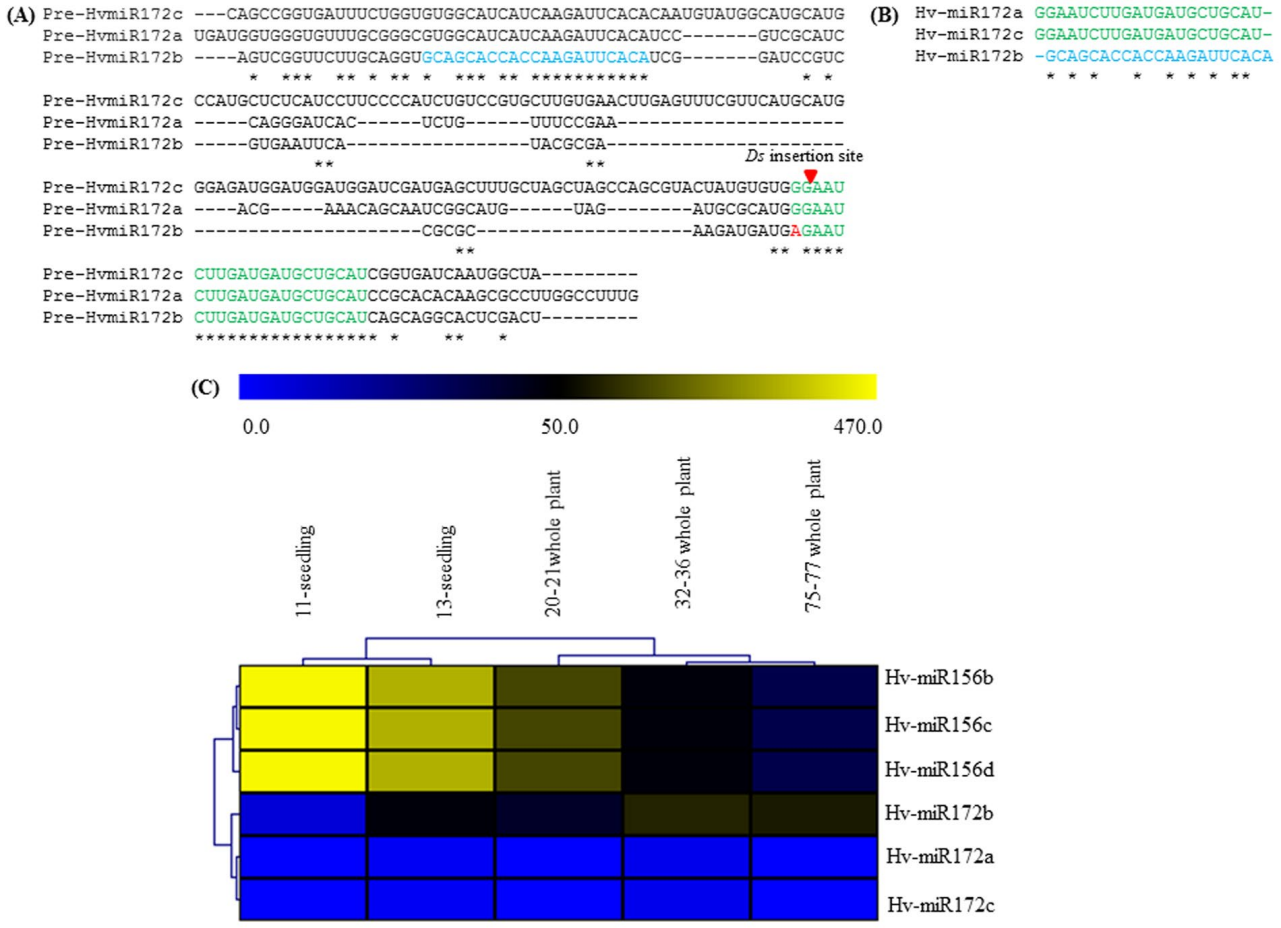
Barley miR172 sequences and expression analysis of miR156 and miR172 family members. (**A**) Alignment of three miR172 (miR172a/b and c) precursor sequences. (**B**) Alignment of three miR172 mature sequences. Green and blue colours differentiate mature sequences of miR172a & c and miR172b respectively. **(C)** Heat map showing the expression profiling of Hv-miR156b/c/d and Hv-miR172a/b/c. Expression data was obtained from publically available database mirex2.0. Antagonistic expression pattern of miR156 and miR172b was observed during vegetative and reproductive phases of barley. The respective transcripts of miR156 and miR172 has been shown in RPM (reads per million). The red triangle indicates *Ds* insertion site in miR172c mature sequence.

The timing of juvenile to adult phase transition in *A. thaliana* is known to be regulated by miR156 and miR172, along with several members of the *SPL* family^38^. We examined their expression patterns in barley during the vegetative to reproductive phase change. The sequences of three miR172 family members (miR172a/b/c) in barley were retrieved from the mirex2.0 database (Fig. 5A,B). The expression patterns of miR156b/c/d and miR172a/b/c in barley tissues collected at 11, 13, 20–21, 32–36 and 75–77 days old plants were examined^46^ (Fig. 5C). As expected, expression of miR156 family members was higher in 11-d-old seedlings stage (vegetative phase) and lower in 70–75 days old plants (reproductive phase). Interestingly, the expression of only miR172b was lower in 11-d-old seedlings and higher in 70–75-d-old-plants. However, the expression of miR172a and of miR172c was lower only in 11, 20–21 and 75–77 day old plants. Thus, miR156b/c/d and only miR172b showed an inverse expression relationship during the vegetative to reproductive phase change, suggesting their contribution in growth phase transition of barley.

**Figure 6:**
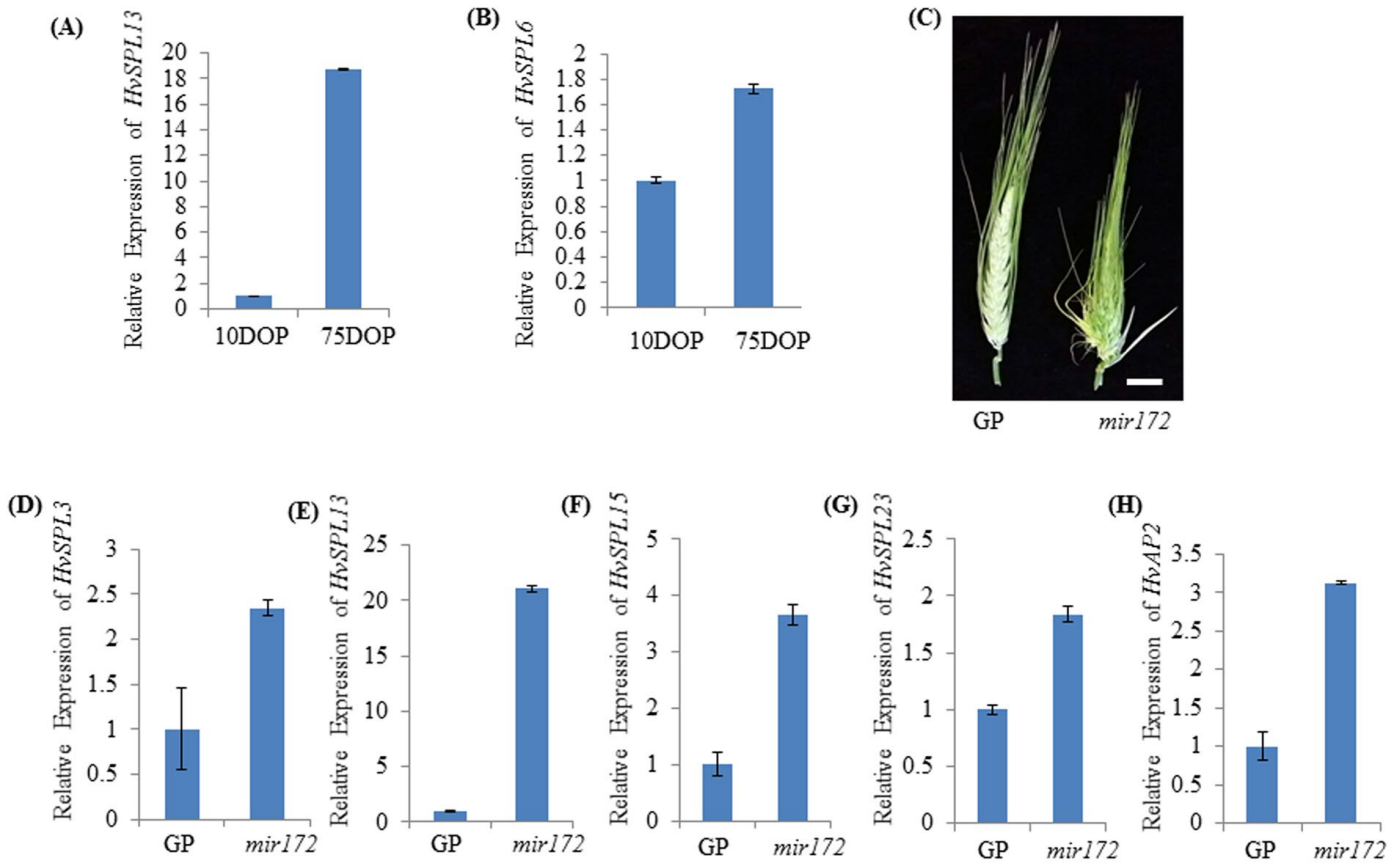
Expression of *HvSPL* genes during growth phase transition in barley. (**A**) Transcript abundance of *HvSPL13* and (**B**) *HvSPL6* at vegetative (10 days) and reproductive (75 days) phases. (**C**) Spike architecture of barley *mir172* mutant and wild type GP. A single 3.6 kb *Ds* insertion in *mir172* mutant was previously identified by Brown and Bregitzer, 2011. This mutant possesses abnormal spike at both apical region (glumes were changed to florets) and basal region (abnormal branched phenotype). (**D–H**) Expression of *HvSPL3, 13, 15, 23* and *AP2* genes in the spike of barley *mir172* mutant and wild type GP. Transcript abundance was measured by qRT-PCR.

To validate the expression patterns of selected *HvSPLs*, barley seedlings were harvested at 10, 16, and 75 days old plants (DOP) and expression of *HvSPLs* was measured by RT-PCR and qRT-PCR (Figs 6A,B and S4). Five *HvSPLs* (*HvSPL3, 6, 13, 15*, and *23*) were selected based on their differential expression *in silico*. All these genes were expressed at low levels during the vegetative phase (10 DAP) and higher levels at the reproductive phase (75 DAP), implying their association with reproductive phase development (Fig. S4). Nevertheless, expression of *HvSPL15* remained stable throughout the development. Further investigation by qRT-PCR showed that the transcript abundance of *HvSPL13* and *HvSPL6* in reproductive phase *vs*. vegetative phase was 18- and 1.7-fold higher, respectively (Fig. 6A,B).

### Interaction Analysis of *miR172, HvSPL* & *HvAP2* Genes

Previously, a barley *mir172* mutant line was developed through transposon tagging system in which a 3.6 kb *Ds* sequence was inserted into the mature sequence of miR172^43^. This insertion produced abnormal spikelet development in that the apical region of spike glumes were converted to partially developed florets and basal region showed abnormal branched phenotype. Comparison of miR172a/b/c precursor sequence with *Ds* flanking sequence suggested us that the *Ds* was inserted in the mature miR172c sequence (Fig. 5A). Since SPL/miR156 module control panicle branching by directly regulating the miR172/AP2 module in rice^30,47^, bract and ear glume development in maize^48,49^ and floral meristem identity in *A. majus*^2,28^, expression of *HvSPL* genes in the *mir172* barley mutant was analysed. The expression of *HvSPL3, 6, 13, 15* and *23* was initially investigated through semi-quantitative RT-PCR and followed by qRT-PCR to investigate expression in the immature spikes the *mir172* mutant and wild-type barley plants (Figs S5 and 6C–G). Expression of *HvSPL3, 13, 15* and *23* was higher in the *mir172* mutant than in the wild-type counterpart. Expression of *HvSPL3, 13, 15* and *23* was further investigated through qRT-PCR which showed 2.3 to 21 fold higher expression in the *mir172* mutant. As expression of *HvAP2* is regulated by miR172, we also examined the expression of *HvAP2* in the spike of wild type and *mir172* mutant lines (Figs S5 and 6H). As expected, expression of *HvAP2* was higher in the *mir172* mutant spike as compared to its wild type counterparts. These results suggest the possible indirect feedback regulation of the AP2/miR172 module on *HvSPL* genes in barley.

## Discussion

*SPL* family genes are plant specific transcription factors and have been identified in many plant species, namely *A. thaliana*, rice, wheat, maize, tomato*, populus*, *chlamydomonas*, silver birch, *Brassica napus*, and soybean^7,9,10,13,14,18,19,45,50,51^. The present study represents the first comprehensive analysis of the miR156/SPL/miR172 regulatory hub in barley. Phylogenetic analysis based on amino acid sequences of conserved SBP domain from wheat, rice, and barley (monocot) and from *A. thaliana* (dicot) classified *HvSPLs* into eight different groups (Fig. 1). SPL proteins belonging to the same group appeared to be more closely related to each other than to those of other groups within a species. Differences in exon/intron structures and SBP domains of *HvSPLs* suggest functional diversity in plant development (Fig. S1A,B). The SBP domain binds to consensus nucleotide sequences TNCGTACAA^17,50^, with GTAC being an essential core sequence present on the promoter of its target genes. The zinc finger motif with two Zn ions binding sites in the SBP domain in barley was Cys3His1 and Cys2His1Cys1, and there was a NLS signal at C-Terminus (Fig. S1B). The NLS signal present at the C-terminus partly overlaps with the second Zn ion binding structure^3^. The size of *A. thaliana* SPL proteins range from 131 amino acids (SPL3) to 927 amino acids (SPL12)^17^. In contrast, the size of barley SPL proteins ranged from 112aa (SPL22) to 1130aa (SPL15) (Table 1). Different *SPLs* may have various numbers of exons/introns even though SBP domain of all land plants is determined by the first and second exons^52^.

Categorization of *HvSPLs* by phylogenetic analysis was supported by classification based on motif composition. The majority of SPL proteins belonging to the same group contained similar motif distributions (Fig. S2) *vs* greater dissimilarity compared to other phylogenetic groups, a finding that is similar to those reported for soybean^13^, wheat^14^, and maize^45^, suggesting that these differences could be important for the function of SPLs. We found that motifs 1, 2,7 and 8 were highly conserved and present in most of the HvSPL proteins, a finding similar to that found in wheat^14^. The diversity in motif composition and variation in the amino acid sequences in the SBP domain reveals that *SPL* is a diverse gene family.

miRNAs play key functions in controlling the transcription of target genes. We identified 4 members of miR156 family in barley and target prediction showed that 7 of the 17 *SPL* genes contained a complementary site for this miRNA (Fig. 2A–C; Table S5). The miR156-complementary site was present in coding regions of *HvSPL3, 11, 16, 17, 18*, *23*, and in the 3′UTR of *HvSPL13*. In *A. thaliana*, 10 of 17 *SPL* genes are targeted by miR156, suggesting that miR156 complementary sites in *SPL* genes are conserved across plant species.

Alternative splicing (AS) acts as a “molecular thermometer” that allows plants to generate efficient transcripts to cope up with environmental perturbations^53^. Recently, AS was identified in barley and its possible function was explored by network analysis^54^. *FLOWERING LOCUS T* (*FT*) in *Brachypodium* undergoes age dependent AS and produces two (*FT2α* and *FT2β*) splice variants^55^ but, only *FT2β* was found to be involved in the regulation of flowering. Similarly, 16 of 17 *HvSPLs* undergo AS and generate diverse transcript and protein sequences (Fig. 3, Table S6). Most of the splice variants of miR156-targeted *HvSPLs* exhibited miR156 complementary sites, implying the existence of alternate splicing-mediated regulation of biological processes in barley (Fig. 3, Table S7). Splice variants of *HvSPL8, 11*, and *20* displayed age-dependent differential expressions, implying their role in barley growth phase transition (Fig. 3B–G). The different splice variants of *HvSPL8, 11*, and *20* were expressed less in the vegetative phase and more in the reproductive phase, revealing their possible association with reproductive development. This is consistent with the previous results for phase transition in *A. thaliana* by *SPL* genes^5,38^. In *A. thaliana, SPL4* promotes vegetative phase change and flowering^5^. Similarly, the male fertility and gynoecium differentiation in *A. thaliana* was shown to be regulated by *SPL8*^56,57^. Exploration of *cis*-regulatory elements in the promoter regions of *HvSPLs* exhibited both conservation and divergence (Table 2). *SPL* genes have been shown to be responsive to light, hormone and abiotic stresses^45,58^. The majority of the *cis*-regulatory elements were involved in response to light signalling, plant growth and development, stresses and hormones. The presence of plant_AP-2-like motif in *HvSPL9, HvSPL17* and *HvSPL18* promoter region suggest the possible feedback regulation of *HvSPL* genes by AP2 like transcription factor. The elements for abscisic acid (ABA), gibberellic acid (GA), salicylic acid (SA), methyl jasmonate (MeJA) and auxin were enriched. Earlier studies in *A. thaliana* indicated that floral transition was regulated by gibberellin guided miR156-targeted *SQUAMOSA* PROMOTER BINDING-LIKE transcription factors^58^. The hormonal pathways phenomenon awaits investigation which has not thus far been reported for the *HvSPL* genes. This result was consistent with previous studies reported in wheat^59^ and *A. thaliana*^60^ which suggested the diverse role of *SPL* gene family on the life cycle of plants.

Upregulated expression of *HvSPL* genes in barley inflorescence revealed their major role in inflorescence development (Fig. 4). Importantly, tissue-specific differential expression of miR156-targeted *HvSPL* genes also suggests that they have possible key role in barley growth and development. Fully developed plants progressed through juvenile to adult (vegetative phase) and reproductive phases that are certainly under precise genetic regulation. In *A. thaliana*, these phases are regulated by miR156 and miR172^38^ *via SPL* genes. The expression pattern of miR156 family members and miR172b in barley vegetative to reproductive phase was also antagonistically related (Fig. 5A–C). Expression analyses of *HvSPL3*, 6, *13*, and *23* in our study are aligned with the expression pattern of miR156 and miR172b during vegetative and reproductive phases suggesting the similar role of miR156-HvSPL-miR172b module in growth phase modifications in barley as observed in *A. thaliana*^38^ and maize^61^ (Figs 6A,B and S5).

The regulation of different aspects of plant developments is contributed by the diversity of *SPL* genes, for instance in bract and ear glume development^48,49^, fruit ripening and grain yield^10,62^, juvenile to adult phase transition and flowering^63,64^, fertility^16^, and embryonic development^65^. To see the effect of AP2/miR172 module on *HvSPL* genes, we examined the expression patterns of *HvSPLs* in the spikes of a *mir172* mutant and its wild type counterpart, which produce indeterminate and normal spikes, respectively (Fig. 6C–G). In our study, *HvSPL3, 13, 15*, and *23* were differentially expressed in the *mir172* mutant when compared to control spikes, suggesting a possible feedback regulation of the miR172/AP2 module. This possibility is in need of further investigation. Down-regulation of *AP2* genes is also mediated by miR172^39^. The up-regulation of *HvAP2* in *mir172* mutant spikes as compared to their wild type counterparts proved its negative regulation by miR172 (Figs S5 and 5H). Recently we have observed that *Ago4_9* genes in barley and wheat are differentially expressed during the reproductive phase^66,67^. As *Ago4_9* is part of the RdDM pathway machinery, it remains to be seen if reproductive phase-specific *SPLs* are also epigenetically controlled via the RdDM pathway. We are currently elucidating these scenarios in reproductive phase development of cereals. We conclude that when inflorescence architecture is altered by down-regulation of miR172, the *SPL* gene expression may be altered as a consequence of that or due to up-regulation of *AP2-like* gene. The results of the current study revealed that the miR156/HvSPL/miR172 module functions as key molecular integrators that affected developmental phase transitions and spike development in barley.

## Materials and Methods

### Identification and Annotation of *SPL* Genes in Barley

The DNA coding sequences (Table S1), protein sequences (Table S2), and DNA genomic sequences (Table S3) of *HvSPLs* were obtained from the Ensemble *H. vulgare* database (http://plants.ensembl.org/Hordeum_vulgare/Info/Index) database. The pHMMER search function was used, with the *A. thaliana* SBP domain (Pfam: PF03110) sequence as the query. The IPK Barley BLAST server (http://webblast.ipk-gatersleben.de/barley_ibsc/) and Phytozome (https://phytozome.jgi.doe.gov/pz/portal.html#!info?alias=Org_Hvulgare_er) databases were also searched by performing TBLASTN using SBP domain sequence as a query. The *HvSPL* gene accession numbers were extracted. The nomenclature of putative *SPL* genes in *H. vulgare* was based on rice and wheat orthologs.

### Gene Structure and Phylogenetic Analysis of *HvSPL* Genes

The Gene Structure Display Server program (http://gsds.cbi.pku.edu.cn/index.php) was used to predict the exon/intron structure of each *HvSPL* gene by comparing their coding and genomic sequences. *SPL* sequences of *A. thaliana* were obtained from TAIR (http://www.arabidopsis.org/index.jsp)^17^. *SPL* sequences of rice were obtained from the rice genome annotation project database. Wheat *SPL* sequences were taken from^14^. The amino acid sequences of conserved SBP domains were selected for phylogenetic analysis. The SBP domain sequences of SPL proteins from *A. thaliana*, rice, wheat, and barley were identified by the SMART tool^68^ and are presented in Table S4. The phylogenetic tree was constructed using the maximum likelihood method using JTT + G as a best model. SBP domain amino acid sequences were converted to the PHYLIP format and analysed with the PhyML3.0 software (http://www.atgc-montpellier.fr/phyml/)^69^, which uses the approximate likelihood-ratio test (aLRT) and depends on a non-parametric Shimodaira-Hasegawa-like (SH-like) approach.

### Conserved Motif Identification, *Cis*-Regulatory Elements, miR156 Target Site Prediction and Alternative Splicing Event Analysis

A search for conserved motifs within HvSPL proteins was performed by using the MEME 4.11.0 tool (http://meme-suite.org/tools/meme)^70,71^ using default settings, except that the maximum width was 50, the minimum width was 6, and the maximum number of motifs to find was 10. The online WebLogo3 platform (http://weblogo.threeplusone.com/) was used to create the sequence logo of the barley SBP domain. The genomic and cDNA sequences of *HvSPLs* were analysed to predict the putative target sites of miR156 using psRNATarget tool (http://plantgrn.noble.org/psRNATarget/?function). Information on alternative splice events for each *HvSPL* gene was obtained from the Ensemble database (http://plants.ensembl.org/Hordeum_vulgare/Info/Index). Promoter regions, defined as the 1000-bp sequences upstream of start codons were searched for *cis-*regulatory elements using the PlantCARE database^72^.

### *In Silico* Gene Expression Analysis of *HvSPLs*

‘Morex’ RNA-seq data was obtained from plant expression ATLAS (https://www.ebi.ac.uk/gxa/plant/experiments) which was generated by International Barley Sequencing Consortium (https://ics.hutton.ac.uk/morexGenes/), and the log2-transformed fragments per kilobase per million fragments measured (FPKM) values were used to study the expression of *HvSPLs* in eight tissues: 4-day-old embryos dissected from germinating grains (EMB); roots (Roo) and shoots (LEA) collected from seedlings (10-cm shoot stage); developing inflorescences (5 mm; INF1 (5 mm) and INF2 (1–1.5 cm); developing tillers at the six-leaf stage (3rd internode, NOD); developing grain 5 days post anthesis (DPA); spikelets with bracts removed at 5 DPA (CAR5) and 15 DPA (CAR15). A heat map of the expression of *HvSPLs* was generated by the average hierarchical clustering method^73^ using the MeV tool (http://www.tm4.org/mev.html).

### Expression Analysis of Barley miR156 and miR172 Family Members

The mirEX2.0 web portal (http://www.combio.pl/mirex) provides a comprehensive platform for the examination of microRNA expression data based on next generation sequencing (NGS) experiments. For barley, data from the two-rowed cultivar Rolap was obtained for five developmental stages: 1-wk-old and 2-wk-old seedlings, whole plants at the beginning of tillering and stem elongation, and at the milk development stage of the kernel^46^. Data are expressed as RPM (reads per million) for the miR172 and miR156 members normalized to all miRNAs identified in the sample. Heat map based expression pattern was generated using the MeV tool.

### Plant Material, Sample Preparation and RNA Isolation

Barley plants were grown on a 14/10hrs-day/night cycle in a controlled growth room, with a day temperature of 25 °C and a night temperature of 20 °C. Tissue samples were collected from 10, 16, and 75 days old plants (10, 16, and 75 DOP, respectively) and immature spikes. The samples were frozen immediately after harvest by immersion in liquid nitrogen, and stored at −80 °C prior to RNA isolation.Total RNA was extracted using the spectrum plant total RNA Kit (Sigma- Aldrich, St. Louis, MO, USA) according to the manufacturer’s protocol. All samples were quantified for RNA concentration on a NanoDrop ND-1000 (NanoDrop Technologies, Wilmington, DE, USA) and electrophoresed on 1% agarose gel to test the integrity and purity. Each sample was treated with DNase I to remove genomic DNA contamination (Invitrogen, USA). The samples were incubated at 23 °C for 15 minutes, followed by the addition of, 1 µl of 25 mM EDTA to each sample, and further incubation at 65 °C for 10 minutes to terminate the reaction.

### First Strand cDNA Synthesis and Quantitative Real-Time PCR (qRT-PCR) Analysis

For each sample, first strand cDNA was synthesized from 1 µg total RNA sample using the AffinityScript QPCR cDNA Synthesis Kit (Agilent technology, Canada). Analysis via qRT-PCR was performed in optical strip tubes using the Mx3000 qPCR system (Stratagene, USA). Each reaction was carried out in a 20-μl volume containing 1 μl diluted cDNA, 5 µM gene specific primers, and 10 μl Brilliant III Ultra-Fast SYBR® Green QPCR Master Mix (Agilent, USA) with the following conditions: 10 min at 95 °C, 40 cycles of 15 s at 95 °C, and 30 s at 60 °C. The expression of *actin* and *GAPDH* were used as internal control^67^. Three technical and two biological replicates were used. The relative level of gene expression was analysed by the 2^−ΔΔCq^ method (Livak and Schmittgen 2001). Barley *actin* or *GAPDH* transcript was used to adjust the relative transcript level for semi-quantitative RT-PCR. The gene‐specific primers used in semi-quantitative RT‐PCR and qRT-PCR for barley *SPL* genes are presented in Table S8. The primers for barley *SPL* genes were designed based on their cDNA sequences. PCR was performed using GoTaq® Green master mix (Promega, USA). PCR for barley *actin* or *GAPDH* was run for 30 cycles, whereas PCR cycles for barley *SPL* genes was run for 34 to 35 cycles. Twelve μl of the RT-PCR products were analysed by 1.2% agarose gel electrophoresis.

## Electronic supplementary material

Supplementary Information
