# Supplementary material for: Genome-wide analysis of the SPL/miR156 module and its interaction with the AP2/miR172 unit in barley
Source: Sci Rep. 2018 May 4;8:7085. doi: 10.1038/s41598-018-25349-0 (PMC5935748; doi:10.1038/s41598-018-25349-0)
Supplement: Supplementary file 1 — Supplementary Information [file 41598_2018_25349_MOESM1_ESM.pdf]

Genome-wide analysis of the SPL/miR156 module and its interaction with the AP2/miR172 unit in barley

Rajiv K. Tripathi[1], Phil Bregitzer,[2] and Jaswinder Singh[1]*

[1]Plant Science Department, 21111 Rue Lakeshore, McGill University, Quebec, H9X 3V9, Canada
[2]USDA-ARS, National Small Grains Germplasm Research Facility, Aberdeen, ID 83210, USA

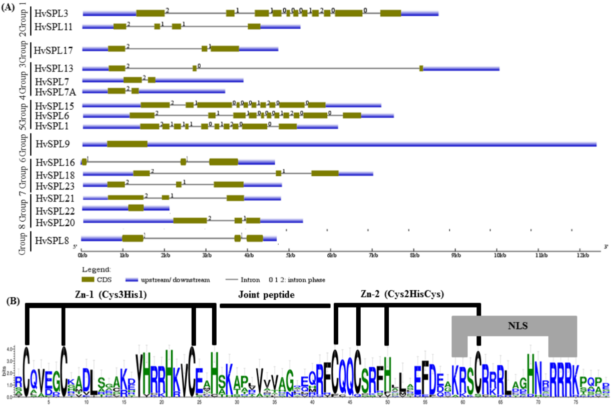

Figure S1. Gene structure and sequence logo of barley SBP domain. (A) The 5' upstream, 3' downstream, exons and introns are represented by boxes and lines, respectively and their sizes can be estimated using the scale bar at bottom. Blue boxes represent 5' and 3' un-translated regions, green boxes represent exons whereas introns are represented with black line. The numbers indicate intron phases. **(B)** Sequence logo of SBP domain was obtained from Weblogo. The overall height of letters represents residue conservation.

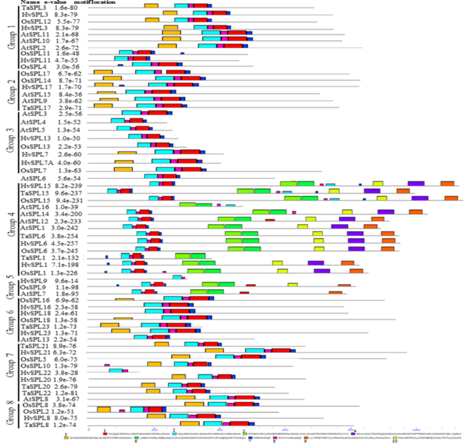

Figure S2. Motif search in SPL proteins. The distribution of 10 conserved motifs is indicated by numbered colored boxes.

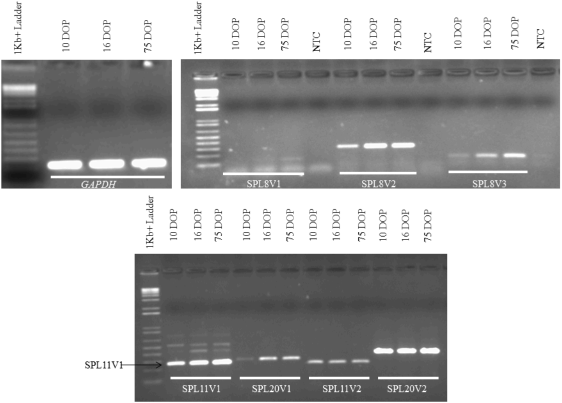

Figure S3. Full length gel images of semi quantitative RT-PCR of alternative spliced variants of *HvSPL8*, *11* and *20* genes in 10, 16 and 75 DOP of barley growth and development. *HvGAPDH* was used as control.

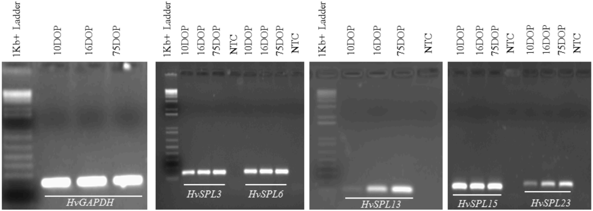

Figure S4. Full length gel images of semi-quantitative RT-PCR of *HvSPL* genes in vegetative and reproductive phases of barley. Expression of *HvSPL3, 6, 13, 15* and *23* in 10, 16, 75 d-old plants. *HvGAPDH* was used as control for semi-quantitative RT-PCR.

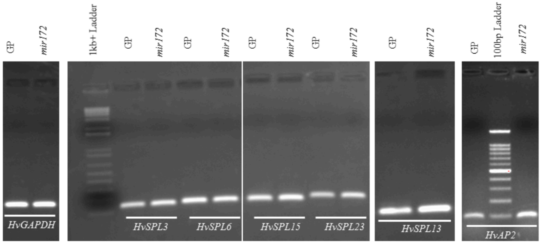

Figure S5. Full length gel images of semi-quantitative RT-PCR based expression of *HvSPL3, 6, 13, 15, 23* and *AP2* in the spike of *mir172* mutant and wild type GP. *HvGAPDH* was used as control for semi-quantitative RT-PCR.

Table S1. Coding sequences of 17 barley SPL genes.

>HORVU0Hr1G039170.6 cds sequence
ATGGACTGGGATCTCAAGATGCCGCCCGGCGCATGGGACCTCGCCGACCTGGAGAGCGAC
GCCGCGCCGGCGCCGGCCGGTGGCATTGCTAATGCGGAGTGCTCCGTGGACCTGAAGCTC
GGCGGGCTCGGCGAGTGCGGCGCTGCCCCGGACACCCGCGGCCTCGGCAAAGCGCCGGCC
GAGGCGGCGGCGTCGCCCAGCGCGGCCAAGCGCCCGCGCGCGTCGTCGGGAGGCGGCGCG
GGGCAGCACCAGCAGTGCCCGTCGTGCGCGGTGGACGGGTGCAGGGCCGACCTGAGCAGG
TGCCGCGACTACCATCGCCGCCACAAGGTGTGCGAGGCGCACTCCAAGACCCCCGTCGTC
ACCGTCGCCGGCCGCGAGATGCGCTTCTGCCAACAGTGCAGCAGGTTTCACCTGCTTACG
GAGTTTGATGAGGCCAAACGCAGCTGTAGAAAGCGTCTTGATGGGCACAACCGTCGCCGC
AGGAAACCGCAGCCGGATCCGATGAATTCTGCAAGTTTTATGACGAGTCAACAAGCATTT
TTGCGAAACCCAGGAACAAGGTTTCCATCATTTCCAACTCCAAGACCGGAGCAAAACTGG
CCAGGGATCATTAAAACTGAGGAGAACCCCTATTACACACATCAGCTCCCTCTAGGCATC
AGTAACAGGCATCATTTTGGTGGCTCTACATCTACTTTCGCGAAAGAAGGACGGAGATTT
CCTTTCCTACAGGAGGGCGAAATAAACTTTGCCACCGGTGTGGCACTTGAGCCTGCAGTG
TGCCAGCCGCTGCTCAAGACGGTAGCTCCTCCCGAGAGCAGCAGCAGCAGCAGCAAGATG
TTCTCCGATGGTCTGACTCCGGTGCTCGACTCGGACTGTGCTCTCTCTCTTCTGTCAGCT
CCGGCAAATTCCTCCGGTATCGATGTTGGCCAGATGGTCCAACAGACTGAACACATCCCC
ATTGCCCAACCTCTGTTCTCCAACCTGCAGCAATTCAGCAGCTCGTCCTGGTTCTCGCGC
ACCCAGGCTTCCACCGGCACCGTCTCAGCGACCGGATTTTCCTGCCCCGTGGTGGAAAAT
GAGCAGCTGAACAATGTCCTAACCTCGGACAACAATGACTTGAACTACAATGGGATTTTT
CATGTCGGAGGCGAAGGCTCCTCAGACGGCGCTCCGCCATCTCTGCCCTTCCCGTGGCAG
TAG
>HORVU7Hr1G051400.2 cds sequence
ATGCAGAGGGAGGTGGGGCCGCAGGTGGCCTCCCCGCTGTACCTGCACCAGATCCAGCCG
CTGCCTCCCCATGCGGCGGCGGCGGCCAGGAAGCGCGGGACCCCGTGGCCCGCCGCGGAC
CCGCCGGAGAACGCCGCGATGGGGGCCGGCGCCGCCGCCGGAGGGAACTGGAACCCCAGC
ATGTGGGACTGGGACAGCCGCGCCTTCACCGCCAGGCCGTCCTCCGACGCGCTCCGCCTC
GGCGGCGGCCTGAACCACCACCAACACCACCACCAGCAGCCGCCGCCGCCGCCGCCGCCG
GCGACGGCTGCCGAGGCGCAGCGGCAGGGGCGCGGAGGTGCCGGTGACCTGAGCCTTCAG
CTGAACCTGCGGGAGGAGGCATCGATGGCGATGGATGTGAGCCCGACAACTACCATGTCT
TCTTCGCCTTCTCCGCCTGCACGGACGTCGCAGGAGCAGGCTGCTAGGCCTAGCAAGAGG
GTTCGGTCTGGATCGCCCGGAACTGCTTCTGGAGGAGGTGGCGGCGGCGGTGCCGGAGGG
AGCGCAAGCGGAGGCGGGAGCTATCCCATGTGCCAGGTGGATGACTGCCGCGCGGATCTC
ACCAGCGCCAAGGATTATCACCGGAGGCACAAGGTGTGTGAGATCCACAGCAAGACAACC
AAGGCGGTAGTTGCCAACCAGATGCAGCGCTTCTGCCAGCAGTGTAGTAGATTTCATCCA
CTCTCGGAGTTCGATGAGGGTAAGAGGAGTTGCAGGCGAAGGCTTGCCGGACACAACCGA
CGACGGAGGAAAACCCAGCCAACAGATGTTGCTTCACAGTTGCTGCTTCCTGAAAACCAA
GAAAACACAGCAAATAGGACACAGGATATTGTCAATCTGATCACTGTTATTGCACGCTTG
CAAGGTGGTAATGTTGGTAAACTACCCAGCATCCCTCCGATACCGGATAAAGATAATCTG
GTCCAAATCATAAGCAAAATAAACTCTATCAATAATGCAAACTCCCTGGGGAAGTCTCCT
CCATCAGAAGCCATTGATTTGAATGCCTCCCATGGGCAACAACAAGATTCCCCTGTTCAA
AATGCAACAAAGGTGGTCGACAAGCAGACTGTGCCATCAACCATGGATTTGTTAACAGTT
CTATCCGGTGCTCTTGGGACATCAACCCCTGAAACCAATACATCTCAGTCCCAAGGGAGC
AGTGATAGCAGTGGTAATAACAAGAGCAAGAGTCATTCAACAGAGCCAGCTTGTGTTGTA
AATTCCCATGAGAAATCAATTCGACCTTTTCCGGCAGCTGGTGTGATAAGGAGCAATAGC
ACCCATGGAAGTCCACCTGAAATATATAAGCAGCCAGACCGAGATACCCATCCATACTTG
TCACTGCAGTTGTTTGGTAATGCCGAGGTGGACATTCCTGTTAAAATGGACACTGCAAAT
AAGTACTTGTCTTCGGAGAGCAGTAATCCTATGGACGAGAGATCTCCTTCATCCTCTCCA
CCTGTAACGCGCACATTTTTCCCCACCCGCTCAGTAAATGAAGGCATCAGACATCCTCGT
ATTGCTGACTATGGAGAAGACGCTGCAACAGCTGAGATCAGTACCACTCGGGCATGGTGT

GCACCACAGCTTGAGCTTTTCAAGGATTCAGAACGGCCAACGGAAAATGGGTCACCACCA
AACCCCACATATCAGTCATGTTATGCCTCAACATCTGGTTCAGACCATTCTCCGTCAACA
TCAAATTCAGATGGACAGGACCGTACTGGAAAGATTATATTCAAGCTCTTTGGCAAGGAA
CCTGGCTCAATCCCTGGGAACCTTCGTGACGAGGTTGTAAATTGGCTGAAACACAGCCCC
ACTGAAATGGAGGGCTACATTCGCCCTGGTTGCCTTGTACTATCCATGTATCTGTCAATG
CCAACTATTGCATGGGATGAACTTGAAGAGAATTTTCTCCAGCGAGTAAACTCGTTAGTT
CAGGCTTCTGATTTGGATTTCTGGAGAAAAGGGAGGTTTTTAGTTCGAAGTGACAACCAG
TTGGTGTCGTACAAAGATGGAATGACCCGCCTGTCAAAATCATGGAGAACATGGAATACT
CCCGAGTTGACCCTTGTGACACCAATTGCTGTTGTTGGTGGGAGAAAGACCTCCCTCGTT
CTTAAGGGCCGTAATCTAACGATCCCGGGCACCCAGATCCACTGTACCAGTGGAGGGAAG
TATATATCAAAAGAGGTATTATGTTCAGCGTATCCGGGTACTATATATGATGATTCAGGA
GTTGAGACCTTTGACTTGCCGGGAGAACCAAATCTTACTCTTGGGCGTTGCTTTATTGAG
GTTGAGAACAGGTTCAGGGGAAACAGCTTCCCTGTTATTTTTGCGAGTAAAAGCATCTGT
CACGAGTTAAGAAACCTTGAAGCTGAACTTGAAGATTCGCGGTTTCCTGATGTCTCTTCA
GATGATCAGGTCCATGATGCTAGGCGGCTAAAGCCAAGGGATCAAGTTCTGCATTTTCTT
AATGAACTTGGCTGGCTCTTTCAGAAGGCCGCTGCATGCACACCTTCCATCGAATCAGAT
GTTTCTGATTCAGAGTTGATTCAGTTCTCTACTGCACGGTTCAGACACCTTCTGTTGTTT
TCAAATGAGCGGGACTGGTGCTCTCTCACTAAAACACTACTCGAAGTTCTTTCCAAGAGA
AGTTTGGTCAGTGAGGAGCTATCACAAGAGACTCTGGAGATGCTCTCTGAGATTCATCTT
CTGAACAGGGCAGTGAAAAGGAAGAGTAGCCACATGGTGCATTTGCTTGTGCAGTTTGTC
GTAATTTGCCCTGACAACTCCAAACTGTACCCTTTCCTTCCAAATTATCCCGGCCCAGGT
GGTTTGACTCCATTACATCTAGCTGCATCCATTGATGATGCAGAGGATATAGTTGATGCC
TTGACAGATGATCCTCAACAGATTGGTTTGAGCTGCTGGCATTCGGTGCTAGATGATGAA
GGGATATCTCCTGAAGTGTATGCGAAGTTCAGGAACAACGGCTCATACAATGAACTTGTT
GCGCGAAAGCTTGTGGACAGGAAGAATAGCCAGGTTACCATTGTGCTCAACAAAGGGGAA
ATTCATATGGATCAACCAGAGAATGCTGGTGCGAATAATTCATCTGGGATCCAAGCATTG
GAAATAAGATCCTGCAGCCAGTGCGCCATTTTGGAGTCTGGCTTGCTAAGACGCCCTATG
CGTTCTCGAGGATTGCTTGCTCGCCCCTATATCCATTCAATGCTTGCCATAGCAGCAGTG
TGTGTCTGTGTCTGTGTATTCATGCGAGCTTTGCTGCGGTTTAATTCTGGTAGGACCTTC
AAGTGGGAGAGGTTGGATTTTGGTCCCACCTAG
>HORVU5Hr1G076380.9 cds sequence
ATTTTCCATGTGATTTATGTCACTATTCATCGGATTTTCATTGTTTCATTTTGGGTTCTC
TTTGCTTTTCATTTTTGTTACTTTGCTTATYTTTMTRTGTGTTTTCTTTGGTGTTCTTCA
ATTTTCATTGGAATTTGTTCCTTTTATGTTTTNNNNNNNNNNNNNNNNNNNNNNNNNNNN
NNNNNNNNNNNNNNNNNNNNNNNNNNNNNNNNNNNNNNNNNNNNNNNNNNNNNNNNNNNN
NNNNNNNNNNNNNTCGTGCGCGGTGGACGGCTGCAAGGCCGACCTGAGCAAGTGCCGCGAC
TACCACCGGCGGCACAAGGTGTGCGAGGCGCACTCCAAGACGCCCCTCGTCGTCGTCGCC
GGCCGCGAGATGCGCTTCTGCCAGCAGTGCAGCAGGTTCCACATGCTTGCCGAGTTTGAT
GAGGCTAAGCGTAGCTGCAGAAAGCGCCTTGATGGGCACAATCGACGCCGCAGGAAGCCA
CAGGTAGATAGCATGAATTCTGGGAGCTTCATGACAACCCAACAAGGGACAAGGTTCGCG
TCCTTCTCGGCTCCAAGGCCGGAGCCAAGCTGGTCCGGGATCATCAAATCCGAGGACAGC
AATCCATTCTACAGCACGCACCAGGTTAACTTCGGCGGCGCCACGTCGTCATACTCCAAA
GAAGGCCGGCGCTTCCCCTTCCTCCACGAAGGAGACCAGATGAGCTTCAGCACCGGCGCG
CCAGCACTCGAGATCCCCGTGTGCCAGCCTCTCCTCAAGGCCGTCGCCCCGCTGCCGCCG
CCGCCCGAGAGCAGCAGCAGCAACAAGATGTTCTCCGATGGACAGTTGACGCACGTGCTC
GACTCCGACTGTGCTCTCTCTCTTCTGTCATCCCCGGCCAACTCCTCCAGCGTCGACGTC
AGCCGGATGGTCCGTCCAAGCGAGCACGTCCCCTCGGTCCCCAGCCTGCAGTTCGGCACC
TCCTCCTGGTTCGCCTGCTCCCAGGCCTCAAGCGGCGGCGGCGCCACCGGATTCGCCGCC
ACCTTCCCCGGCGGCATGGACGGCGAGCAGCTCAACGCCGGCGGCGCGCTGGTCCCCGGC
TCCAACGACAACGAGATGAACTGCCACGGGATCTTCCACGTCGGCGCCGATGGCTCCTCC
GAGGGCACGTCGCCGTCCCTCCCGTTCTCATGGCAGTAG
>HORVU6Hr1G031450.6 cds sequence

ATGGAGTGGACGGCCCCGAAGTCCACCCCGTCCTCCCCGCCCCACCTCCTCTGGGACTGG
GGCGACGCCGCCGCGCTGGGCTCCTCCGGCGAGGCGGCGGGGAGGCGCGGGAAGGAGAAG
CGGGCCAGGGGGGAGGGGGCCGGCGGAGGAGGCGGAGGAGGGGGCGTAAGGTGCCAGGTG
GAGGGCTGTGGGACCGAGCTCGCCGCCGCGAAGGAGTACCACCGGAAGCACCGGGTCTGC
GAGGCCCACACCAAGTCCCCCCGCGTCGTCGTCGCCGGCCAGGAGCGCCGCTTCTGCCAG
CAGTGCAGCCGGTTCCATGGGCTGTCGGAGTTCGACCAGAAGAAGAGGAGCTGCCGGAGG
CGACTGTCTGATCACAATGCTCGGCGCCGGAAGCCACAGCCGGATGCATTCTCCTTTGCA
CCGGCAAGGCTACCGTCGTCATTGATGTTTGATGATAGACGACAAATAAGTTTTGTCTGG
GATAAAGATCCACTTAGCCATGGAAGGCCTTTCCCATGTTCTCCATGGGACAGCCCATCT
GACTTCAAGCCCCCACAAGTGAAGGAAATAAGAGAAGTGTCAATCAATGGACAAGTTCAT
TTTGATAAATCTCATCTACCCAATGCTGTTCCAGCACTGAGTCATGACATAGCTGAGCTG
CTACCAATGAAAGGTCCGGACGCATCTGCAACCGCTTCGAAATTAGGTGGAGCACCGGAT
CTTCAGCGTGCTCTCTCTCTTCTGTCAGCTAGTTCTTGTGGATTACCTGATCCAGTACAG
CAAGCATCTTGTCTCGTCCAATTCACTGGTGCCAGCCAAAACAGCCGGGGTCTGCCTTCA
CCACATGGAGGGAGCCCTCCGTCGGCATCCTGTGCCGAGGGACAGCCCATGGCTCCATCA
CCTCAGTTCGTCCGTTTCACGATGGATGGCGCCAGCAGTGGCTATGAATCCACTTTCTTC
GGCGTAAACCGGATGAATTGA
>HORVU7Hr1G042370.8 cds sequence
ATGTCTAGTGGGCTCAAGAACAAGAAGAAGAAGGGCCACGAGTGGGATTTGAACGACTGG
AGATGGGACGGCAACCTCTTCCTGGCCGCACCGTCGTCAAACGCGGAAGCGCCGTCGGGG
TGCGGCAGCAGGGAGCTCGGGCGGGCTGAGGAAGGGGGCAGTTTTGGTGCTGCTGACAAG
AGCAGGAGGAGGAGGAGGAGAGTTTCCACGGTGGATAACCCCGAGGAGCCTCCACATGAA
AGGATTGCTGTTCGGAGAGGACAGATCAGCGAAGAAGAGAGACCTGCGAATGCAACAGCA
GGTGCATCTTCCAGCTCTGCTCCATCTTGCCTAGTCGACGGCTGCCACGCGGATCTCCGC
GACGGTAGGGACTACCATAGGAGGCACAAGGTGTGTGAGGTGCACACCAAGTCGACTCTT
GTTCGTATCAAAAACATAGAGCATCGGTTTTGCCAGCAGTGCAGCAGGTTTCACCTTGTT
CAAGAATTTGATGAAGGGAAGAAGAGCTGCCGCTCACGTCTAGAAAAACATAATGGGAGG
AGGAGGAAAGCCCAGGCCCAGGCTGTGAGTTCCTCGAGTGAAAATCAGTCCTTAACCAAT
ACCTTACTCCTCTTACTGAGACAACTCGCTGGACAAGATTCTGCTGCTAGCTCGTCTGAG
CAAATCAATGGTCCGAATTTCTTGGTCAATCTTCTGAAGAACCTTGCTGCTGTTGCTGGC
ACACAGGCATGTCAAGATATGCTAAAGGATGCAACATCATCAAATGCTGGTAACTATGTT
GGTAATCAAAGTGGACCACCAGTTCCTGCAGAAGAGCCTCCTATGAAAAGACGTGCGCGG
AATTTTGATCTGAATGACACTTATGTCGAGGAAGATGAGNNNNNNNNNNNNNNNNNNNNN
NNNNNNNNNNNNNNNNNNNNNNNNNNNNNNNNNNNNNNACTGATCTACGCACACAGGTCCTA
AACTGGCTGTCACATTATCCAAGTGACATGGAGAGCTATATTAGGCCTGGTTGTGTAATT
CTAACTATTTACCTTCGTCTTCCTAACTGGATGTGGCATAAGCTTAATGTCGATCCAGCT
CCTTGGATAGAAAATCTTATTAGCATATCCACTCATGGTTTCTGGGAGACAGGATGGTTG
TATGCTAGGGTGCAGGACCGCCTGACATTAAGCTGCAATGGCAGGCTTACGTTGGTGTGT
CCATGGCTACCTGTAATAGGTGACAAGCATCAGATACTGTGTGTAACTCCCATTGCGGCT
TCTTGTAAGTCGACGGCAAACTTCTCAGTGAGAGGCTTCAACATAGCTCAACCAACCACA
AAATTACTTTGTATATTTGGTGGGAAATATTTAGTCCAAGAAGCAACACAAAAACTACAA
GATGATACTGCGGTTCAGCAAGGGCCTCAATGTCTGGCCTTTTCTTGCTCCTTCCCTAGT
ACAAGTGGAAGGGGGTTCATAGAGGTTGAAGATTATGATCAGAGCAGCATTTCATTTCCC
TTCGTTGTCGCTGAAGAATCTATATGTTGTGAGATTCGAATGTTGGAGGAAAAACTGAAT
ATATTTGCATTTGCTAATGTCTTGGAAGGAAGAGAAGATCTGATGGCTTCTCGCAGCCAA
GCCTTGAAGTTTCTACATGAAATAGGGTGGCTTCTTCAACGGAGCCACTCACGAGCTACA
TCATCCAAGGCTCCACAACAACATCGTGTTGTGGGTTTTCTTGCTGCAAGATTTAGATGG
CTCCTGTCCTTTGCGGTCGATCAGGAATGGTGCGGCGTCGTAAAGATGCTGCTGGACACC
TTGTTCCAGGGCAATATTGACGTCCCCTCACCAGTTGAGTTTGTCCTGGGAGAGAGTTTA
GTGTTCAAGGCTGTCAACAAGCGGTCCAAGCCCTTGGTTGACTGCCTATTGAGATACACA
ACGAATTCTGCGCCAGTGGGCGGTGGAGCAGTGGCTACACCAGCTCGGTTCGTATTCACG
CCTGACATGACTGGTCCATCAGATATTACACCTCTCCATAGTGCAGCTACCATCAGTAAT

GCTGCTGCTGTTTTGGATGCTCTAACTGATGATCCTCAACAGCTAGGGATCAAAGCCTGG
AAGAATGCCCGCGATGCGACCGGGTACACTCCAGAGGATTACGCTCGGAGGAGAGGGCAC
ACATCCTACATCCAGATGGTCCAGAACAAAATTAACAGGGGGTTACCTGCAGCCCATGTG
TCTGTTTCCATGACCACCACTGGTATCACCGAAGAGCATGCAGATGCAGGTCGTCCGAAA
TCAACTGATCAGACCATATTTGATGTCGAGAAAAGCCTACCGGGATGCAGACAATGTGTC
CAGCTCCAGCACATTGCCTACCGGCCCTGCCCAAACAGGTTCTTGTCGAACAGGCCTGCG
GTGCTATCCTTGGTCGCCATTGCCACTGTCTGCGTCTGCGTAGGTTTGATAATGCAGAGC
CCGCCGGTCGTCCGCGGTATGCCGGGTCCTTTTCTCTGGAATCGCCTCCGTTGGGGACCC
ACTTGA
>HORVU5Hr1G073440.7 cds sequence
GCGGCGGCGGCGGCAATGGAGACCGGGAGCAGCGGCGGCGGGAGAGGGCCCGGGGACGAC
GTCCACGGGCTCCACTTCGGCAAGAAGATCTACTTCGAGCAGGACGTGGCCGGCGGGAGC
GGCTCCTCCTCCTCCGCCTCCGGTGGCAGGAGGGGCAGGGGCCCCGCTGGGCCAGGAGGA
GGGGGAGGTGGTGGTGGCGGTGGCGGTGGCGGCGGCGGAGGAGGAGGAGCCTCCACCCCC
GCGGCCGGCGGCAGCGCCTCGCAGTCGCAGCAGCCGAGGTGCCAGGTGGAGGGGTGCGGC
GTGGATCTGAGCGGCGGCAAGACCTACTACTGCCGCCACAAGGTGTGCTCCATGCACTCC
AAGGCGCCGCTCGTCGTCGTCGCCGGCATCGAGCAGCGCTTCTGCCAGCAGTGCAGCAGG
TTCCACCAGCTACCTGAATTTGACCAAGGAAAACGAAGCTGCCGCCGACGCCTCGCGGGT
CACAATGAGCGCCGGAGGAAGCCGCCGCCCGGACCTATGTCGACACGCTATGGCCGGCTT
GCTGCGTCCTTCAACGAAGATCCAGGCAGGTTCAGAAGCTTTCTGCTGGATTTCTCGTAC
CCGAGGGCCCCGGCCGGCGTGAGGGACCCGTGGCCGGCCGTTCAGCCCGGCGACCACCGC
ATGCCTGGCACCACCCACTGGCAGGGCCAGGGCAGCCATCATGAACAACACCACGCTCAT
CGCAGTGCAGTTGCGGGATACGGCGACCACCACGCGTACAACGGCCAGGGCAGCAGCTCG
GGGGGAGGCGGGGCGCCGATGATCCCGGCGGGCTTCGAGCTCCCCTCGGACGAATGTATG
GCGGGCGTCGCCGCCGACTCCAGCTGTGCTCTCTCTCTTCTGTCAACTCAGCCATGGGAT
AGTAGTGCCCACAGCTCCAGCCACATCCGGTCCCCGGCAATGTCGACGGCCAGCGCCTTC
CAGGGCAGCCCGGTGGCGCCCTCCGTCATGGCCAGCAACTACATGGCGGCGGCGAGCAGC
AGCGGCTCCTGGGGTAGCCCCCGGGGCGCCAGGAGCATGCAGCAGCAGCACCACCACCAT
CACATGCAGCATGACACGGTGATGAACGAGGTCCATCCGAGCTCGGTTCACCACGGGCAG
TTCGGCGAGCTCGAGCTGGCGCTGCAGCAGGGGAGGGCCACGCCGAACCCGCCGCACGCC
GAGCACGGGGCCTTCAGCCACTCCAGCAACGCGATGAACTGGTCTCTGTAG
>HORVU2Hr1G097610.1 cds sequence
ATGGAAGGAGACGGCGACGCTAGGTCGAGGGCGGGGCTGGCCTGGGATCTCGGGATGCAG
CAGTGGGCGCCCGCCGCCTCGGCTACTTACCCGCAGCATTTCCTGCCGCCGCCGCCGCCG
GGGGTGGCTAGCCACCACCGGGGGGGGGGGGGGCTAGCCTAAAGCTGGGTAAGCGCCCC
TGCTCGCTGGCCGGCGTCCAGGTGGCTCAGGTGGACGGCACCGGGGCTGCCGTGGTGGAA
GGCAAGAGGAAGGAGAAGGCGGGCGCGCCGGCAACGGCGGCCATGCCAAGGTGCCAGGTG
GAAGGGTGCCACATGGTGCTGGCGGGGGCCAAGGAGTACCACCGGCGGCACAAGGTGTGC
GAGGCGCACTCCAAGGCGCCCAGGGTCATCGTGCACGGCGCCGAGCAGCGCTTCTGCCAG
CAGTGCAGCCGCTTCCACGCGATGGCGGAGTTCGACGACGCCAAGCGGAGCTGCCGCCGG
CGCCTTGCCGGCCACAACGAGCGGCGCCGGAAGAGCAACGCCAACGACGCCATGGCCAGG
GGCACTGCGCACGCACACGGTAATCCATGTCATTACTCGCACACGTCCATCAGCGCGAAA
TACTCCTGTTGA
>HORVU2Hr1G097580.1 cds sequence
ATGGAAGGAGACGGCGACGCTAGGTCGAGGGCGGGGCTGGCCTGGGATCTCGGGATGCAG
CAGTGGGCGCCCGCCGCCTCGGCTACTTACCCGCAGCATTTCCTGCCGCCGCCGCCGCCG
GGGGTGGCTAGCCACCACCACCAGCAGCAGCAGCATATGCAGCAGGAGCTCACTAGCCTA
AAGCTGGGTAAGCGCCCCTGCTCGCTGGCCGGCGTCCAGGTGGCTCAGGTGGACGGCACC
GGGGCTGCCGTGGTGGAAGGCAAGAGGAAGGAGAAGGCGGGCGCGCCGGCAACGGCGGCC
ATGCCAAGGTGCCAGGTGGAAGGGTGCCACATGGTGCTGGCGGGGGCCAAGGAGTACCAC
CGGCGGCACAAGGTGTGCGAGGCGCACTCCAAGGCGCCCAGGGTCATCGTGCACGGCGCC
GAGCAGCGCTTCTGCCAGCAGTGCAGCCGCTTCCACGCGATGGCGGAGTTCGACGACGCC

AAGCGGAGCTGCCGCCGGCGCCTTGCCGGCCACAACGAGCGGCGCCGGAAGAGCAACGCC
AACGACGCCATGGCCAGGGGCACTGCGCACGCACACGGTAATCCATGTCATTACTCGCAC
ACGTCCATCAGCGCGAAATACTCCTGTTGA
>HORVU6Hr1G030490.2 cds sequence
TCAAGGCACGCACAAGCACAACCCTTCTGTGCTGCAAAGATACATACACACCCGGCTTCT
TGCTTCCTCACACATCAGTCAGGCCGAGCGTCACCCACCTTTCTCATCCGCGCGCGGGAC
AGCCGGCCAATGATGAGCAGCAGGCTTAACGTCGGCACGATGGCACCGGTTAGCGACGTG
GTCGACTTCGGTTATGCTCCCATACAGTCCTACCCCAACTTTGAGTCGGCCGGCCTGGCC
TTGCCCGGTGACCGGCAGCCACCGTTCCAGCACCATCACCTCTACGATAGCTTCGACTTC
ACCTCTGCTAACTTCTCGTTTCAAGACCCGGCCGCGCTCTTCTCCGGCGACTCGGCGCTC
AGCAACCAGCTCCAGCAGCCCTTCGTCCAGTCGCAGGTCACCATGCCGACGACGGCGTCC
TCGTCGGTGCTGCAAATGCCGATGATGACGCTGCCGGGCATGCTGATGTCTTCCTCCTCG
TCGCTGGCGGCGGACGCATGCACCTTCGGTGGCGGTGGAGGGTTCCTGAAGCGGGAGGAC
GGCGGGCCCTTCTCGGACATCGGCGGTGGCGGGAGGATCGGGCTGAACCTCGGCCGCAGG
ACATACTTTTCCCCGGCGGACGTGCTGGCCGTGGACCGCCTGCTGATGCGCTCCCGCTTC
GGCGGAGCTGGCCCAATGGGTATGCTGGGGCTGGGGCTCGGCGCCGCCGCCCACCACCAC
CAGCCCCCGCGGTGCCAGGCCGAGGGCTGCAAGACTGATCTCTCCGCTGCCAAGCACTAC
CACCGCCGCCACAAGGTCTGCGAGTACCACGCCAAGGCCGCCACCGTCGCAGCCTCCGGC
AAGCAGCAGCGCTTCTGCCAACAATGCAGCCGGTACGTTGTGCTCGCTGAGTTTGACGAG
GCCAAGAGGAGCTGCCGGAAGCGGCTCACAGAGCACAACCGGCGCCGCAGAAAGCCCGCC
GGCGCGCAGGCAGGCAAGGACTCGCCGGCGCCCCCTACCAAGAAGCCAGACGGTAGCATC
ACCAGCTCATACACCGGTGATCACAGGACGAACAAGTCGACGACGGCGGCGGCCTTCTCG
CCGAGCGCCAGCAGCTTCAGCTGCTTTCAGCAGCAGCAGCATGCGCTCGACAACGGCGGC
CAGTCGAACAACGCCACGCCAACGTCGGCGCCTCCGACGCTGTCCCTGGCGGCGCCACCA
TCGCCGCCTCAGCAGAACGACGGCGACTTCGGCTCCGGCCTTGACACCATGCTGCAGATG
CAGCCGCAAGAGGCCGATGAGCAGGGGGAACAGCAGCAGCATCTCATGATGACCTCGCTC
GTGCAGTCGCAGCGGCAGCAGCAGCACCGCGACAGCGGGAACATCTTATCTTGCTCAACG
ACGTCGGCGTCGGATCAGCGTCACCACCACAACAACGGCGGCAGCTGCTGCAACGACAAC
AGCATGCAGCATTTCTTCGAGGTGGACTTCATGTAG
>HORVU6Hr1G019700.3 cds sequence
ATGATAAGTAGATACTACCAGCAGTGCATCATCTTTGTTGTCAGCAGCTTTGACACACAA
GTAGGAGGAGGAGCGGCGCCGGAGATCTTGGGCTCTGGCATGGGCTCGTTTGGGATGGAG
TGGAACCAGAGGAGCTCCGTGCTGTGGGACTGGGAGAATTTCCCGCCGATAGGCGGCGAC
AACCCCAAGAATGCGATGCAGGCCGAGCCAAGATTCGCTGCCGTTGCGGCTGCCATGGGG
AATGAACCGCTCAATTCTTCTGGCGGCAGCGGCACCTTCTCTTCCAGCTCGGAGATGGGG
TATGGCTCCTCCAAGAGCTCCATATCCGCGTCGATCGATTCTTCGTCCAAGGCTGGGAGC
AACATGGAGTTCAGATTTGCGCCCGTCAAAAACCCTGACAGGAACACGAGCGAAAACACT
GAGTTGGGTAAAGTTGACAACACGAGGACTGGAACGTCTCCGTCGTCTGTGGTGGCAGTG
AGCAGTGGAGAGCCGGTGATCGGCCTGAAGCTCGGCAAGAGAACCTACTTTGAGGATGTC
TGTGGAGGGCAGAGTGTCAAGAGCTCGCCATCGGGTGCTGCGAGTGTGCCGAACAAATCT
CCTGCTTTGAGCAAGAAGGCAAAGGCGGAACAACAGAAGGCACATAACTCATACTGTCAG
GTTGAAGGCTGCAAGGTTGATCTCTCTTCTGTTAAAGAGTACCATCGAAAGCATAGAGTA
TGTGAACTTCACTCTAAGGCTCCCAAAGTTGTTGTCGCTGGTCTGGAGCGGCGCTTTTGC
CAACAGTGCAGCCGGTTTCATGCTTTGTCTGAGTTTGACCAGAAAAAGCGAAGCTGCCGT
AGGCGTCTCAATGATCATAATTCCCGCAGGCGGAAGCCACAGCCAGAAGCAATTTCCTTC
AGTTCGTCAAGGATGTCTACGATGTTTTATGATGCAAGGCAACAGCCAAATTTCCTATTC
GGTCAGGCTCCATATGTTCAAATGAGAAGCTGTGGAAGTTCTTCATGGGATGAACCAGGA
GGCTTCAAAGTTACACACACAAAAGCTCCTTGGTTAAAGCCAACAACTGCTGCAGGTGTT
GATGGGATGCATTTATCTAGTCAGCAGATGTCGGACAATGTTATGCCGCATGGTGCACAT
CATGGTTTCGATGGGTTCATGGCATTCAAGGGAACTTCTACAAAGTTCCCTAATCAAGGT
GTCCAAGCTTCCGCTGTCGCCCCCAACTCCAGTGGCGCCCCGGATCTTCAGCATGCTCTC
TCTCTTCTGTCAAGCAACCCAGTGGGTGCTGCCAACCTCCAGCCAACTTCCCAGATGCAC

TCTGGGGTGGCTGCCATTGCCGGCACCCCCAACCCCGCGATGCACGTGCTGGGATCATCG
GCAGGGCTCTGGCTAGACGGCGGGCCACCCCTAGATGATCACCCGCGGTTCCAGGTCTTC
GAGCGCTTGGGGGACCACGACAGCGAGCTCCAGCTCCCAAAGCCTTCCTACGACCACGCC
TCGCACTTCGACCGGATGCACTGA
>HORVU2Hr1G048280.2 cds sequence
CCCTCCGCTTCTTCTCCTTCTCCTTCCACTCATCAGCTAGCTCCACAGTTCCACTTCTGC
GACAGCTCCCCGAGAAGAGCCCTAGCTAGCTTCGTCTTCATCGCGCCTCACCGGAAGGCA
TGGACCGCAAGGACAAGTCTCGCAAGTCCCCCTCAGCAGCGTCCATGGCAGCGCTCGCCG
CGGCAGCTGNNNNNNNNNNNNNNNNNNNNNNNNNNNNNNNNNNNNNNNNNNNNNNNNNNN
NNNNNNNNNNNNNNNNNNNNNNNNNNNNNNNNNGAGAGGTGCGGCGCCGACCTCACCGAG
GCGAAGCGGTACCACCGCAGGCACAAGGTGTGCGAGGCGCACTCAAAGGCTGCTGTCGTG
GTCGTCGCCGGTCTGCGCCAGCGCTTTGTCAGCAATGCAGCCGGTTCCACGAGCTTCTG
GAGTTCGACGACCAAAAGCGCAGCTGCCGCCGGCGCCTGGCCGGGCACAACGAGCGGCGG
AGGAAGAGCTCGGCTGAGNNGAACGGCGGCGACGGGTGCCGCCACGCCGACCAGGACGGC
CGTAGCCACCCTGGGAACCCGCCGCTGAACCATTTCCAGATCAGATAA
>HORVU3Hr1G094730.1 cds sequence
GAATTCTCTGGACACAGAGAGACGTGCGTGCGTGCGCGTGTATTTGGCATGGATTCTTGG
GAGCCCAAGATGCCTTCTTGGGACCTCGGCACGGTGGTCGCGCCCAGCGGCGGCGGGGGA
GGAGGAGGCGGCGGCGGGGCGCTGGACCTGAAGCTCGGCGCGCCGACGAGCTGGAGGCCG
GTCCCGGCGCCGGTTGTATCGGTGCAGCAGCAGCAGCAGCAGCAGCCGCCTTCCGCGGCG
ACGGCGAAGCGTGCCCGGGCGGGGCAGGGGCAGCAGACGGTGCCGGCGTGCTCCGTGGAG
GGGTGCACGGCCGACCTGTCCCGGTGCCGCGAGTACCACCGCCGGCACAAGGTCTGCGAG
GCGCACTCCAAGACGCCCGTCGTCGCCGTCGCCGGCCAGCAGCAGCGCTTCTGCCAGCAG
TGCAGCAGGTTCCATTTGCTCGGGGAGTTCGACGAGGTGAAGAGGAGCTGCAGGAAGCGG
CTCGACGGCCACAACCGGCGCCGCCGGAAGCCGCAGCCGGATCCCCTCAACCCTGCCGGC
CTGTTCGCTAATCACCACGGAGTGCCAAGATTTGCGTCGTACCCGCAGATCTTCTCCCCG
ACGTCCATGGCGGAGCCCAAGTGGCCAGGCGGCATCGCCGTGAAGACGGAGGCCGACGCG
TTCCACGAGCAGTACTACTCCTTCAACGGCGCCGCCTCCCTCTTCCACCACGGCAAGCCG
GAGAGGAAGCACTTCCCCTTCCTGACCGACGGCGGCGACGCCACGTTCGGCTGCCAGCCG
CCGGCGTTCACCATCACCCCTTCCTCGGAGAGCAGCAGCAACAGCAGCCGGCACAGCAAC
GGCAAGACCACCATGTTCGCCCACGACGGGGGCCCGGACCACAACTGTGCTCTCTCTCTT
CTGTCAGACAACCCGGCGCCGGCGCACATCATGAACCCCGCGGAGGCGCATCATCTCGGT
GGCGGCGGCGGCGTGACCATACAGTACGGCGGTGGAGGCGGCGGCAAGGTGGCGCGGCTG
CACAACAACGGCGACGTCTCGCTCACCGGGCTGTCTTACGTGAGCCTGGGTGACAAGGGC
GCGCCCATGTTGTTGCACACGTCTAACAGATCACAACACGCCGCTGCTACCGCTACCACT
GCCGTTACTACAAGCACAGCTCCAGCTCCGACGGCGTCGCAGATGCAGCAGTACCATGGC
TACTACCACCACCAACACCAAGTGGGCGCTGATCAGGGGAACCACCAGGATGCTGGTGGG
ATGCATGCCCTTCCCTTCTCGTCATGGTAG
>HORVU7Hr1G110980.3 cds sequence
ACTCTCACTCTCTCTCCTTGGCCGGCCGGCTCCCTTTTTAACCACCTCTGCGCCCTCCAC
CACCTTACACAGCACAGCACAACCACACTCATTCACCACCCAGCGAGCTCATCACAACCA
CACGCGCCTTCTCCCAGTCCCAGCTCACTCGGGCCGGCCATGATGAGCGGCGGCAGGATG
AACGGCCCGGCGAGCGACGACTTCCCGTTCGGGGCAATGCAGCCGCCGCCCTACGTCGGC
TTCGAGCACGCCGGCGGAGGAGGTGGCCAGCGCCATCAGGGCGCGATGATGTACGACAAC
TTCGACTTCGCGGTCGCGGCGGCCGCCTTCGGCCAGTTCCAGGACGCGCCGCACCACCAG
ATGCTGGCGATGCCGCCGAACGGGAGCGGAGCAGGCGGGCTGGTCCCCATGGCGCCGCCG
CCCATGCCCATGCCCGGGATGCAGCTGCAGATGCCGCCCATGCCCCACGGTATGCACGGC
CACGGCGACGTGTGCCCGTCGATGGGGATGGTGAAGCGCGAGGGCGGCGCCGGGGACGCG
GGGAGGATCGGTCTGAACCTCGGCCGGCGGACCTACTTCTCCCCCGGAGACATGATGGCC
GTGGACCGGGTGCTGATGCGGTCCCGTCTCGGCGGCGTGTTCGGGCTGGGATTCGGCCGC
GCCCACCAGCAGGCGCCCCGGTGCCAGGTGGAGGACTGCAAGGCGGACCTCTCCGGCGCC
AAGCACTACCACCGGCGCCACAAGGTCTGCGAGTACCACGCCAAGGCCGCCCTCGTCTCC

ACCGCCGGCAAGCAGCAGCGCTTCTGCCAGCAATGCAGCAGGTCACACGTGCTCATGGAG
TTCGACGAGGCGAAGCGGAGCTGCCGGAGGCGGCTCGCAGAGCACAACCGTCGCCGGAGG
AAGCCGGCGGCCGGCAGCACGGCGACGTCGTCGAAAGACTCGGCGCCGCCTTCCAAGAAA
CCCAACGCCGGCGCCATCTGCAGCTCCTACACCGGCGACAACAGCAGTTTGAGCGCGACG
AAATCGACCATCTCCTCCAACACGAGCGCGATCAGCTGCCTCCAGCTGCAGGGCCAGGCC
AGGGCGGCAGCTGCGGCGAGGCCGACGGCGCTCACCCTCGGTGCGCCGCCGGAGAAGGAC
GACCAGCAGCAGCAGTTCAGGAACGCCATGCAGCTCCACCACCATCAGGAGCACCAGCAC
TTCATCACCTCCCTCCTGCACGGCAACATCAATAACAGCAACATCCTGTCGTGTTCCTCG
GTGAGCTCCAACACGATGCCATCGGCGGTGGCGGCCAACGGCGAGGACGACAACAACAAC
ATGCATATGTTTGAGGTGGACTTCATGTAG
>HORVU5Hr1G117190.5 cds sequence
ATGGACTTGGAGGCGGCCAGGGTAGGGTCGCAGAGCCGCCACCTGTACGGAGGCGGGCTG
GGTGGCGAGCTTGAGCAGGCCAGGCGCGAGAAGCGGGTGTTCGGCTGGGACCTCAACGAC
TGGAGCTGGGACAGCGAGCGCTTTGTCGCCACGCCGGTGCCCGCGGCAGCGGGGAATGGA
CTGTCGCTCAACAGTTCGCCGTCTTCCTCCGAGGAAGCCGGGCCTGAGGTGGCTAGGAAT
GGTAACGTTAGAGTTGACTCTGATAAGAGGAAGCGAGTGGTTGTCATTCATGATGATGAC
AATGAGAAGGATGATGACCCAGTGGGGAACAGTAGCAATGTGCTCAGCTTGAGACTTGGC
GGCGGCAACTCTGTTGCCGGCGGAGCGGCGGAGGATGGCGGCGTGAACGAGGAGGACAGA
AATGGTAAGAGGATCAGGGTGCAGGGCGGAAGCTCAAACGGTCCGGCGTGTCAGGTGGAG
GGCTGCTGCGCTGACCTTAGCGCGGCAAAGGATTACCATCGGCGGCACAAGGTCTGCGAG
ATGCATGCTAAGGCCAACACTGCGGTGGTCGGAAATACCGTCCAGCGGTTCTGCCAGCAA
TGCAGCAGATTTCACCTTCTTCAAGAATTTGATGAAGGAAAGCGAAGTTGTCGCCGGCGT
TTAGCAGGCCATAATAAACGTAGGAGGAAAACCCGCCCTGAAAGTGCTGTTGGTGGGGCT
CCTATTGAGGAAAAAGTTAGCAGTTATTTATTGTTGAGTCTTCTTGGAATATGCGCCAAT
TTTAACTCTGACAATGCTGAGCGTGTACAAGGTCAGGAGTTGCTATCCAATCTTTGGAGA
AACTTGGGGACTGTTGCAAAATCATTGGATCCAAAAGAACTCTGTAAACTCCTGGAGGCG
TGTCAGAACATGCAAAATGGATCAAATGCTGGGACCTCGGAAGCAGCTAATGCTTTGGTG
AATACTGCTGCAGCAGAGGCTGCAGGACCATCTAACTCTAAGGCGCCTTTTGCGAATGGT
GGTCAATGTGGGCAGTCGTCATCTGCTGTTGTACCAGTACAATCAAATGTTCCCATGGTG
GCAACTACCGAGATTCCAGCATGCAAGTTAAAGAATTTTGATTTGAATGACACTTGCAAC
GATATGGAAGGCTTTGAGGATGGTTCGAATTGCCCATCATGGATACGTCAAGATTCTACT
CAAAGCCCACCACAGACTAGTGGTAATTCGGATTCAACATCAGCTCAGTCACTGTCAAGC
TCAAATGGAGATGCTCAGTGTCGGACTGATAAAATTGTATTCAAGCTTTTTGAGAAAGTT
CCTAGTGAATTACCTCCAATTTTGCGATCACAGATCCTTGGTTGGTTGTCCAGTAGCCCT
ACTGATATAGAGAGCTATATCAGACCTGGCTGTATTATCTTGACAGTTTATCTTCGTTTA
GTTGAGTCTTCATGGAGAGAACTCTCTGAGAATATGAGTGTATATCTGGATAAGCTCTCA
AGTAGTTCTGCTGATAACTTTTGGACATCTGGTTTGGTATTTGTGATGGTACAACATCAA
ATTGCTTTTATGCACAATGGTCAAGTTATGTTGGACAAACCACTGGCACCTAATTCTCAC
CATTACTGCAAGGTTTTATGTGTTAGTCCGGTTGCTGCTCCTTTTTCAGCAACAGTTAAT
TTCAGGGTAGAAGGCTTCAACCTAGTCAGTTCTTCCTCGAGGCTAATTTGCTCAATTGAA
GGGCGTTCTATATTCGAAGAAGACACAGCTATCATGGCTGATGATACCGAGGATGAAGAT
ATTGAATATCTCAACTTCTGTTGTTCTCTCCCTGATACAAGAGGAAGAGGATTCATAGAG
GTTGAAGATAGTGGATTTAGTAATGGTTTCTTCCCCTTCATAGTTGCTGAGCAGAATGTG
TGCTCTGAGGTTTGTGAGCTGGAGGGCATATTTAAGTCATCCAGTCATGAGCAGGCAGAC
AATGACAATGTCATGAATCAAGCTTTAGAGTTTCTACATGAGCTGGGGTGGCTTCTTCAT
AGAGTTAACATAATTTCTAAGCATGATAAAGCGGAGCTGCCTGTAGCGGCCTTTAACCTG
TTGAGATTCAGGAATCTTGGTATATTTGCGATGGAGCGGGAGTGGTGTGCTGTGACCAAA
ATGCTGTTAGATTTGTTATTTGATGGATTTGTTGATGCTGGGTTGCAGTCTCCCAAAGAG
GTGGTATTGTCAGAAAATTTGTTACACTCTGCTGTGCGAGGAAACTCTGCCCGAATGGTT
AAATTTCTGCTGACATACAAGCCGAACAGAGGCCTGAATGAAACTGCAGAGACATACCTA
TTCAGACCTGATGCTCGGGGCCCTTCTGCATTTACACCCCTACATATAGCAGCCGCTACT
AGTGATGCAGAGGATGTATTGGATGCATTGACTGATGACCCTGGACTGGTGGGACTGAAC

GCATGGAGAAATGCGCGAGACGAGACAGGCTTTACCCCTGAAGACTATGCTCGCCAAAGA
GGCAACGATGCTTACATCAATCTGGTCCAGATGAAGATTGACAGGCATCTTGGCAAAGGT
CATGTTGTCCTTGGTGTTCCCAGCAGCATGTGCCCTGCAATTACCGATGGGGTGAAGGCA
GGTGATATCAGCCTGGAGATCTGCAAAGCCATGCCAGTGACAACAACATCCGCCGCAAGG
TGCAACATTTGCAGTCGTCAGGCTAAGATGTACCCAAGTTCCTTTGCGAGGACCTTCCTG
TACAGGCCGGCAATGTTCACCGTGATGGGCGTCGCCGTGATCTGTGTTTGCGTCGGGATC
CTCCTCCATACCCTCCCCAAGGTTTATGCCGCACCAAATTTCAGATGGGAGCTGTTAGAG
CGCGGAGCCATGTGA
>HORVU1Hr1G060770.1 cds sequence
GCTCTCCGTCACGGCGTCACGCCGTCACCAACCGCACGCGGCACGCCGAACTCGAGGGCC
CACGCGCCAGCAGTAGCGCGACTGGCCCCGGCCGCCCTACGCCGCTCACCTACCATTACC
AAAGCCTTTCCTTCCTCCTCCCGCGACGAGACGACCAATTCGCCCGCCCTCGTCGCGTCG
CCGGCCGCCTTCTCCCCCCTCCCCACCACGCCCGACGCCTCCGCGGAACCGCGAACGAGA
CCCAGCGTAGCGAGAGCCGATTGGGTCGCGCGCGCGCCGGGTTTTTGGGGCTAGGGCT
TGGCTCGTGGTCGCGGTTTCCAGTTCCCAGGAAATGGACGCCTCCGACTCCGGCGGGGGC
GGCGCGGCGGCCGATGCGGGGGAACCCGTCTGGGACTGGGACAACCTCCTCGACTTCGCC
ATCCCGGATGACGATTCCCTGCTCCTCCCGTGGGATGACCCACTCGGGATCGAGGGCGAT
CCCACCACCGAGACGGCGCTGCTCCCTGCCCCGCCTCCGCCGCAGCCAGTGGAGCCGGCG
CCTGCGCCACCGTCTCCATCTGTTGAGGCCGGGGCAAGCAGACGCGGCGTCAGAAAGCGC
GACCCGCGGCTGGTGTGCCCTAACTACCTCGCGGGGATTGTGCCCTGTGCGTGCCCCGAG
TTGGATGAGATGGCGGCCGCCGCGGAGGTCGAGGAGGTTGCCGCGGAGGTGCTTGCCGGT
CCGAGGAAGAAGCCCAAGGCCGCCAGCCGGGGTAGCAGCGGGGCGGTAGTCAAAACTGGT
GGTGGTGGCAGTGGCAGTGGAGTGGCTGCCCGTGGGGGAGCAGCAGAAATGAGGTGCCAA
GTGCCGGGCTGTGAGGCGGATATACGGGAGCTGAAAGGGTACCACAAGCGGCACCGGGTG
TGTCTGCGTTGCGCGCACGCGTCTGCCGTCATGCTCGATGGCGTCCAGAAGCGCTACTGC
CAGCAGTGCGGCAAGTAA
>HORVU0Hr1G039150.3 CDS sequence
GGTGGGTACAGCCTCGAGCCCAGCTCCCTGGCGCTCCTCCCCCCGTCCAGCCACGCCACCATCGCTG
CCCACAGCCCCCACGACATCCTCCAGTTCTACC
ACCACCCCACCTCGCACCACTACCTCGCCGCCGCCGCGGCAGGAGGCAACGGCAGCCCCTACGGCC
AATTCGGCGGGGGCGGCGGCGGCGGCGGCGGCTT
CCAGTCTTACTACCACCAGCAGCCGGGGACGGGCGCGCCCGAGTACTACTTCCCCACCTTGGTCAGC
TCGGCGGAGGAGAACATGGCGAGCTTCGCGGCC
ACGCAGCTGGGGCTCAACCTCGGTTACCGGACCTACTTCCCGCCCAGAGGCGGCTACACCTACGGC
CATCACCCGCCCCGGTGCCAGGCCGAGGGCTGTA
AGGCCGACCTCTCGGGTGCCAAGCGCTACCACCGCCGCCACAAGGTCTGCGAGCACCACTCCAAGG
CGCCCGTCGTCGTCACCGCCGGCGGCCTCCACCA
GAGGTTCTGCCAGCAGTGCAGCAGATTCCATCTGCTTGATGAGTTCGACGACGCGAAGAAGAGCTG
CAGGAAGCGCCTCGCAGACCACAACCGCCGTCGA
AGGAAGTCAAAGCCGTCCGACGCCGATGCCGCTGATAAAAGGAGGACACAGGCCAGCAAAGCAGC
AGCAAGTACCAAAGGCAAAGCAGCCGGAAGCAGCA
GCAAGAGCACCGGCACCGGAGACGGGATGGACATTCAGGTGCAGCTGGGGAGCGGAGACCTGTCC
AAAGATCAGGACGAAACCATGGGGCAGGGAGAGGT
GGTGAAAGAAATGCAGGTGGATCCCAAGGGGAAAGCGTCAATGCAGCTGCAGCAGCAGCAACAGC
AAGGACACCACGTCCATGGTCTTCACCTGCAGAGC
CAACATGGCTTCCCTTTCCCTTCGACCTCTGCAGGCTCGTGCTTCCCTCAGAGCCAAGCTGTCTCGAG
CACTGACAACACATCAAATATCGGCCAAGTGC
AGCAAGAACAGCTAGGCTTGGGGTTCCATCAGCACAGCAACATCCTTCAGCTGGGACAGGCCATGT
TTGATCTCGACTTCGATCACTAG
>HORVU7Hr1G110950.1 CDS sequence
GGGAGGATCGGTCTGAACCTCGGCCGGCGGACCTACTTCTCCCCGGGCGACATGATGGCCGTGGAC
CGGCTCCTGATGCGGTCCCGCCTGGGCGGCGTGT

TCGGGCTCGGGTTCGGCGGCGCCCACCACCAGCCGCCGCGCTGCCAGGCCGAGGGCTGCAAGGCCG
ACCTCTCCGGCGCCAAGCACTACCACCGGCGCCA
CAAGGTGTGCGAGTACCACGCCAAGGCGTCCCTCGTCGCCGCCAACGGCAAGCAGCAGCGCTTCTG
CCAGCAATGCAGCAGGTCAGAGCCAGACCCTCCC
TCTCTCTCTCTCTCCCTCCGTAGAAATGGAACAGTAG

Table S2. Amino acid sequences of 17 barley SPL genes.
>HvSPL18
MDWDLKMPPGAWDLADLESDAAPAPAGGIANAECSVDLKLGGLGECGAAPDTRGLGKAPA
EAAASPSAAKRPRASSGGGAGQHQQCPSCAVDGCRADLSRCRDYHRRHKVCEAHSKTPVV
TVAGREMRFCQQCSRFHLLTEFDEAKRSCRKRLDGHNRRRRKPQPDPMNSASFMTSQQAF
LRNPGTRFPSFPTPRPEQNWPGIIKTEENPYYTHQLPLGISNRHHFGGSTSTFAKEGRRF
PFLQEGEINFATGVALEPAVCQPLLKTVAPPESSSSSSKMFSDGLTPVLDSDCALSLLSA
PANSSGIDVGQMVQQTEHIPIAQPLFSNLQQFSSSSWFSRTQASTGTVSATGFSCPVVEN
EQLNNVLTSDNNDLNYNGIFHVGGEGSSDGAPPSLPFPWQ
>HvSPL15
MQREVGPQVASPLYLHQIQPLPPHAAAAARKRGTPWPAADPPENAAMGAGAAAGGNWNPS
MWDWDSRAFTARPSSDALRLGGGLNHHQHHHQQPPPPPPPATAAEAQRQGRGGAGDLSLQ
LNLREEASMAMDVSPTTTMSSSPSPPARTSQEQAARPSKRVRSGSPGTASGGGGGGGAGG
SASGGGSYPMCQVDDCRADLTSAKDYHRRHKVCEIHSKTTKAVVANQMQRFCQQCSRFHP
LSEFDEGKRSCRRRLAGHNRRRRKTQPTDVASQLLLPENQENTANRTQDIVNLITVIARL
QGGNVGKLPSIPPIPDKDNLVQIISKINSINNANSLGKSPPSEAIDLNASHGQQQDSPVQ
NATKVVDKQTVPSTMDLLTVLSGALGTSTPETNTSQSQGSSDSSGNNKSKSHSTEPACVV
NSHEKSIRPFPAAGVIRSNSTHGSPPEIYKQPDRDTHPYLSLQLFGNAEVDIPVKMDTAN
KYLSSESSNPMDERSPSSSPPVTRTFFPTRSVNEGIRHPRIADYGEDAATAEISTTRAWC
APQLELFKDSERPTENGSPPNPTYQSCYASTSGSDHSPSTSNSDGQDRTGKIIFKLFGKE
PGSIPGNLRDEVVNWLKHSPTEMEGYIRPGCLVLSMYLSMPTIAWDELEENFLQRVNSLV
QASDLDFWRKGRFLVRSDNQLVSYKDGMTRLSKSWRTWNTPELTLVTPIAVVGGRKTSLV
LKGRNLTIPGTQIHCTSGGKYISKEVLCSAYPGTIYDDSGVETFDLPGEPNLTLGRCFIE
VENRFRGNSFPVIFASKSICHELRNLEAELEDSRFPDVSSDDQVHDARRLKPRDQVLHFL
NELGWLFQKAAACTPSIESDVSDSELIQFSTARFRHLLLFSNERDWCSLTKTLLEVLSKR
SLVSEELSQETLEMLSEIHLLNRAVKRKSSHMVHLLVQFVVICPDNSKLYPFLPNYPGPG
GLTPLHLAASIDDAEDIVDALTDDPQQIGLSCWHSVLDDEGISPEVYAKFRNNGSYNELV
ARKLVDRKNSQVTIVLNKGEIHMDQPENAGANNSSGIQALEIRSCSQCAILESGLLRRPM
RSRGLLARPYIHSMLAIAAVCVCVCVFMRALLRFNSGRTFKWERLDFGPT
>HvSPL16
IFHVIYVTIHRIFIVSFWVLFAFHFCYFAYXXXCFLWCSSIFIGICSFYVXXXXXXXXXX
XXXXXXXXXXXXXXXXXXXXXXXXXSCAVDGCKADLSKCRDYHRRHKVCEAHSKTPLVVVA
GREMRFCQQCSRFHMLAEFDEAKRSCRKRLDGHNRRRRKPQVDSMNSGSFMTTQQGTRFA
SFSAPRPEPSWSGIIKSEDSNPFYSTHQVNFGGATSSYSKEGRRFPFLHEGDQMSFSTGA
PALEIPVCQPLLKAVAPLPPPPESSSSNKMFSDGQLTHVLDSDCALSLLSSPANSSSVDV
SRMVRPSEHVPSVPSLQFGTSSWFACSQASSGGGATGFAATFPGGMDGEQLNAGGALVPG
SNDNEMNCHGIFHVGADGSSEGTSPSLPFSWQ
>HvSPL11
MEWTAPKSTPSSPPHLLWDWGDAAALGSSGEAAGRRGKEKRARGEGAGGGGGGGGVRCQV
EGCGTELAAAKEYHRKHRVCEAHTKSPRVVVAGQERRFCQQCSRFHGLSEFDQKKRSCRR
RLSDHNARRRKPQPDAFSFAPARLPSSLMFDDRRQISFVWDKDPLSHGRPFPCSPWDSPS
DFKPPQVKEIREVSINGQVHFDKSHLPNAVPALSHDIAELLPMKGPDASATASKLGGAPD
LQRALSLLSASSCGLPDPVQQASCLVQFTGASQNSRGLPSPHGGSPPSASCAEGQPMAPS
PQFVRFTMDGASSGYESTFFGVNRMN
>HvSPL1
MSSGLKNKKKKGHEWDLNDWRWDGNLFLAAPSSNAEAPSGCGSRELGRAEEGGSFGAADK
SRRRRRRVSTVDNPEEPPHERIAVRRGQISEEERPANATAGASSSSAPSCLVDGCHADLR
DGRDYHRRHKVCEVHTKSTLVRIKNIEHRFCQQCSRFHLVQEFDEGKKSCRSRLEKHNGR
RRKAQAQAVSSSSENQSLTNTLLLLLRQLAGQDSAASSSEQINGPNFLVNLLKNLAAVAG
TQACQDMLKDATSSNAGNYVGNQSGPPVPAEEPPMKRRARNFDLNDTYVEEDEXXXXXXX
XXXXXXXXXXXXTDLRTQVLNWLSHYPSDMESYIRPGCVILTIYLRLPNWMWHKLNVDPA
PWIENLISISTHGFWETGWLYARVQDRLTLSCNGRLTLVCPWLPVIGDKHQILCVTPIAA

SCKSTANFSVRGFNIAQPTTKLLCIFGGKYLVQEATQKLQDDTAVQQGPQCLAFSCSFPS
TSGRGFIEVEDYDQSSISFPFVVAEESICCEIRMLEEKLNIFAFANVLEGREDLMASRSQ
ALKFLHEIGWLLQRSHSRATSSKAPQQHRVVGFLAARFRWLLSFAVDQEWCGVVKMLLDT
LFQGNIDVPSPVEFVLGESLVFKAVNKRSKPLVDCLLRYTTNSAPVGGGAVATPARFVFT
PDMTGPSDITPLHSAATISNAAAVLDALTDDPQQLGIKAWKNARDATGYTPEDYARRRGH
TSYIQMVQNKINRGLPAAHVSVSMTTTGITEEHADAGRPKSTDQTIFDVEKSLPGCRQCV
QLQHIAYRPCPNRFLSNRPAVLSLVAIATVCVCVGLIMQSPPVVRGMPGPFLWNRLRWGP
T
>HvSPL17
AAAAAMETGSSGGGRGPGDDVHGLHFGKKIYFEQDVAGGSGSSSSASGGRRGRGPAGPGG
GGGGGGGGGGGGGGGASTPAAGGSASQSQQPRCQVEGCGVDLSGGKTYYCRHKVCSMHS
KAPLVVVAGIEQRFCQQCSRFHQLPEFDQGKRSCRRRLAGHNERRRKPPPGPMSTRYGRL
AASFNEDPGRFRSFLLDFSYPRAPAGVRDPWPAVQPGDHRMPGTTHWQGQGSHHEQHHAH
RSAVAGYGDHHAYNGQGSSSGGGGAPMIPAGFELPSDECMAGVAADSSCALSLLSTQPWD
SSAHSSSHIRSPAMSTASAFQGSPVAPSVMASNYMAAASSSGSWGSPRGARSMQQQHHHH
HMQHDTVMNEVHPSSVHHGQFGELELALQQGRATPNPPHAEHGAFSHSSNAMNWSL
>HvSPL7A
MEGDGDARSRAGLAWDLGMQQWAPAASATYPQHFLPPPPPGVASHHRGGGGASLKLGKRP
CSLAGVQVAQVDGTGAAVVEGKRKEKAGAPATAAMPRCQVEGCHMVLAGAKEYHRRHKVC
EAHSKAPRVIVHGAEQRFCQQCSRFHAMAEFDDAKRSCRRRLAGHNERRRKSNANDAMAR
GTAHAHGNPCHYSHTSISAKYSC
>HvSPL7
MEGDGDARSRAGLAWDLGMQQWAPAASATYPQHFLPPPPPGVASHHHQQQHMQQELTSL
KLGKRPCSLAGVQVAQVDGTGAAVVEGKRKEKAGAPATAAMPRCQVEGCHMVLAGAKEYH
RRHKVCEAHSKAPRVIVHGAEQRFCQQCSRFHAMAEFDDAKRSCRRRLAGHNERRRKSNA
NDAMARGTAHAHGNPCHYSHTSISAKYSC
>HvSPL21
SRHAQAQPFCAAKIHTHPASCFLTHQSGRASPTFLIRARDSRPMMSSRLNVGTMAPVSDV
VDFGYAPIQSYPNFESAGLALPGDRQPPFQHHHLYDSFDFTSANFSFQDPAALFSGDSAL
SNQLQQPFVQSQVTMPTTASSSVLQMPMMTLPGMLMSSSSSLAADACTFGGGGGFLKRED
GGPFSDIGGGGRIGLNLGRRTYFSPADVLAVDRLLMRSRFGGAGPMGMLGLGLGAAAHHH
QPPRCQAEGCKTDLSAAKHYHRRHKVCEYHAKAATVAASGKQQRFCQQCSRYVVLAEFDE
AKRSCRKRLTEHNRRRRKPAGAQAGKDSPAPPTKKPDGSITSSYTGDHRTNKSTTAAAFS
PSASSFSCFQQQQHALDNGGQSNNATPTSAPPTLSLAAPPSPPQQNDGDFGSGLDTMLQM
QPQEADEQGEQQQHLMMTSLVQSQRQQQHRDSGNILSCSTTSASDQRHHHNNGGSCCNDN
SMQHFFEVDFM
>HvSPL3
MISRYYQQCIIFVVSSFDTQVGGGAAPEILGSGMGSFGMEWNQRSSVLWDWENFPPIGGD
NPKNAMQAEPRFAAVAAAMGNEPLNSSGGSGTFSSSSEMGYGSSKSSISASIDSSSKAGS
NMEFRFAPVKNPDRNTSENTELGKVDNTRTGTSPSSVVAVSSGEPVIGLKLGKRTYFEDV
CGGQSVKSSPSGAASVPNKSPALSKKAKAEQQKAHNSYCQVEGCKVDLSSVKEYHRKHRV
CELHSKAPKVVVAGLERRFCQQCSRFHALSEFDQKKRSCRRRLNDHNSRRRKPQPEAISF
SSSRMSTMFYDARQQPNFLFGQAPYVQMRSCGSSSWDEPGGFKVTHTKAPWLKPTTAAGV
DGMHLSSQQMSDNVMPHGAHHGFDGFMAFKGTSTKFPNQGVQASAVAPNSSGAPDLQHAL
SLLSSNPVGAANLQPTSQMHSGVAAIAGTPNPAMHVLGSSAGLWLDGGPPLDDHPRFQVF
ERLGDHDSELQLPKPSYDHASHFDRMH
>HvSPL13
PSASSPSPSTHQLAPQFHFCDSSPRRALASFVFIAPHRKAWTARTSLASPPQQRPWQRSP
RQLXXXXXXXXXXXXXXXXXXXXXXXXXXXXXXERCGADLTEAKRYHRRHKVCEAHSKAAVV
VVAGLRQRFCQQCSRFHELLEFDDQKRSCRRRLAGHNERRRKSSAEXNGGDGCRHADQDG
RSHPGNPPLNHFQIR
>HvSPL23

EFSGHRETCVRARVFGMDSWEPKMPSWDLGTVVAPSGGGGGGGGGGALDLKLGAPTSWRP
VPAPVVSVQQQQQQQPPSAATAKRARAGQGQQTVPACSVEGCTADLSRCREYHRRHKVCE
AHSKTPVVAVAGQQQRFCQQCSRFHLLGEFDEVKRSCRKRLDGHNRRRRKPQPDPLNPAG
LFANHHGVPRFASYPQIFSPTSMAEPKWPGGIAVKTEADAFHEQYYSFNGAASLFHHGKP
ERKHFPFLTDGGDATFGCQPPAFTITPSSESSSNSSRHSNGKTTMFAHDGGPDHNCALSL
LSDNPAPAHIMNPAEAHHLGGGGGVTIQYGGGGGGKVARLHNNGDVSLTGLSYVSLGDKG
APMLLHTSNRSQHAAATATTAVTTSTAPAPTASQMQQYHGYYHHQHQVGADQGNHQDAGG
MHALPFSSW
>HvSPL20
TLTLSPWPAGSLFNHLCALHHLTQHSTTTLIHHPASSSQPHAPSPSPSSLGPAMMSGGRM
NGPASDDFPFGAMQPPPYVGFEHAGGGGGQRHQGAMMYDNFDFAVAAAAFGQFQDAPHHQ
MLAMPPNGSGAGGLVPMAPPPMPMPGMQLQMPPMPHGMHGHGDVCPSMGMVKREGGAGDA
GRIGLNLGRRTYFSPGDMMAVDRVLMRSRLGGVFGLGFGRAHQQAPRCQVEDCKADLSGA
KHYHRRHKVCEYHAKAALVSTAGKQQRFCQQCSRSHVLMEFDEAKRSCRRRLAEHNRRRR
KPAAGSTATSSKDSAPPSKKPNAGAICSSYTGDNSSLSATKSTISSNTSAISCLQLQGQA
RAAAAARPTALTLGAPPEKDDQQQQFRNAMQLHHHQEHQHFITSLLHGNINNSNILSCSS
VSSNTMPSAVAANGEDDNNNMHMFEVDFM
>HvSPL6
MDLEAARVGSQSRHLYGGGLGGELEQARREKRVFGWDLNDWSWDSERFVATPVPAAAGNG
LSLNSSPSSSEEAGPEVARNGNVRVDSDKRKRVVVIHDDDNEKDDDPVGNSSNVLSLRLG
GGNSVAGGAAEDGGVNEEDRNGKRIRVQGGSSNGPACQVEGCCADLSAAKDYHRRHKVCE
MHAKANTAVVGNTVQRFCQQCSRFHLLQEFDEGKRSCRRRLAGHNKRRRKTRPESAVGGA
PIEEKVSSYLLLSLLGICANFNSDNAERVQGQELLSNLWRNLGTVAKSLDPKELCKLLEA
CQNMQNGSNAGTSEAANALVNTAAAEAAGPSNSKAPFANGGQCGQSSSAVVPVQSNVPMV
ATTEIPACKLKNFDLNDTCNDMEGFEDGSNCPSWIRQDSTQSPPQTSGNSDSTSAQSLSS
SNGDAQCRTDKIVFKLFEKVPSELPPILRSQILGWLSSSPTDIESYIRPGCIILTVYLRL
VESSWRELSENMSVYLDKLSSSSADNFWTSGLVFVMVQHQIAFMHNGQVMLDKPLAPNSH
HYCKVLCVSPVAAPFSATVNFRVEGFNLVSSSSRLICSIEGRSIFEEDTAIMADDTEDED
IEYLNFCCSLPDTRGRGFIEVEDSGFSNGFFPFIVAEQNVCSEVCELEGIFKSSSHEQAD
NDNVMNQALEFLHELGWLLHRVNIISKHDKAELPVAAFNLLRFRNLGIFAMEREWCAVTK
MLLDLLFDGFVDAGLQSPKEVVLSENLLHSAVRGNSARMVKFLLTYKPNRGLNETAETYL
FRPDARGPSAFTPLHIAAATSDAEDVLDALTDDPGLVGLNAWRNARDETGFTPEDYARQR
GNDAYINLVQMKIDRHLGKGHVVLGVPSSMCPAITDGVKAGDISLEICKAMPVTTTSAAR
CNICSRQAKMYPSSFARTFLYRPAMFTVMGVAVICVCVGILLHTLPKVYAAPNFRWELLE
RGAM
>HvSPL9
ALRHGVTPSPTARGTPNSRAHAPAVARLAPAALRRSPTITKAFPSSSRDETTNSPALVAS
PAAFSPLPTTPDASAEPRTRPSVARADWVARARRVFGARAWLVVAVSSSQEMDASDSGGG
GAAADAGEPVWDWDNLLDFAIPDDDSLLLPWDDPLGIEGDPTTETALLPAPPPPQPVEPA
PAPPSPSVEAGASRRGVRKRDPRLVCPNYLAGIVPCACPELDEMAAAAEVEEVAAEVLAG
PRKKPKAASRGSSGAVVKTGGGGSGSGVAARGGAAEMRCQVPGCEADIRELKGYHKRHRV
CLRCAHASAVMLDGVQKRYCQQCGK
>HvSPL8
GGYSLEPSSLALLPPSSHATIAAHSPHDILQFYHHPTSHHYLAAAAAGGNGSPYGQFGGGGGGGGGFQS
YYHQQPGTGAPEY
YFPTLVSSAEENMASFAATQLGLNLGYRTYFPPRGGYTYGHHPPRCQAEGCKADLSGAKRYHRRHKVC
EHHSKAPVVVTAGGLHQRFCQQCSRFHLLDEF
DDAKKSCRKRLADHNRRRRKSKPSDADAADKRRTQASKAAASTKGKAAGSSSKSTGTGDGMDIQVQL
GSGDLSKDQDETMGQGEVVKEMQVDPKGKASMQ
LQQQQQQGHHVHGLHLQSQHGFPFPSTSAGSCFPQSQAVSSTDNTSNIGQVQQEQLGLGFHQHSNILQL
GQAMFDLDFDH
>HvSPL22

GRIGLNLGRRTYFSPGDMMAVDRLLMRSRLGGVFGLGFGGAHHQPPRCQAEGCKADLSGAKHYHRRH
KVCEYHAKASLVAANGKQQRFCQQCSRSEPDPPSLSLSPSVEMEQ
>AtSPL1
MEARIDEGGEAQQFYGSVGKRSVEWDLNDWKWDGDLFLATQTTRGRQFFPLGNSSNSSSSCSDEGNDK
KRRAVAIQGDTNGALTLNLNGESDGLFPAKKTKSGAVCQVENCEADLSKVKDYHRRHKVCEMHSKAT
SATVGGILQRFCQQCSRFHLLQEFDEGKRSCRRRLAGHNKRRRKTNPEPGANGNPSDDHSSNYLLITLLK
ILSNMHNHTGDQDLMSHLLKSLVSHAGEQLGKNLVELLLQGGGSQGSLNIGNSALLGIEQAPQEELKQF
SARQDGTATENRSEKQVKMNDFDLNDIYIDSDDTDVERSPPPTNPATSSLDYPSWIHQSSPPQTSRNSDS
ASDQSPSSSSEDAQMRTGRIVFKLFGKEPNEFPIVLRGQILDWLSHSPTDMESYIRPGCIVLTIYLRQAETA
WEELSDDLGFSLGKLLDLSDDPLWTTGWIYVRVQNQLAFVYNGQVVVDTSLSLKSRDYSHIISVKPLAI
AATEKAQFTVKGMNLRQRGTRLLCSVEGKYLIQETTHDSTTREDDDFKDNSEIVECVNFSCDMPILSGR
GFMEIEDQGLSSSFFPFLVVEDDDVCSEIRILETTLEFTGTDSAKQAMDFIHEIGWLLHRSKLGESDPNPGV
FPLIRFQWLIEFSMDREWCAVIRKLLNMFFDGAVGEFSSSSNATLSELCLLHRAVRKNSKPMVEMLLRYI
PKQQRNSLFRPDAAGPAGLTPLHIAAGKDGSEDVLDALTEDPAMVGIEAWKTCRDSTGFTPEDYARLRG
HFSYIHLIQRKINKKSTTEDHVVVNIPVSFSDREQKEPKSGPMASALEITQIPCKLCDHKLVYGTTRRSVA
YRPAMLSMVAIAAVCVCVALLFKSCPEVLYVFQPFRWELLDYGTS
>AtSPL10
MDCNMVSSFPWDWENLIMSNQSKTENEKKQQSTEWEFEKGEGIESIVPDFLGFEKVSSGSATSFWHTAV
SKSSQSTSINSSSPEDKRCNLASQSSPGDSSSNIDFLQVKPSTALEVPIASAESDLCLKLGKRTYSEEFWGR
NNNDLSAVSMNLLTPSVVARKKTKSCGQSMQVPRCQIDGCELDLSSSKDYHRKHRVCETHSKCPKVVV
SGLERRFCQQCSRFHAVSEFDEKKRSCRKRLSHHNARRRKPQGVFPLNSERVFDRRQHTSMLWNGLSLN
TRSEEKYTWGTTYETKPTQMESGFTLSFQRGNGSEDQLFTGSTLSFSAFQTSGGFSAGKSNIQLPDKGVG
ECSGGLHESHDFYSALSLLSTTSDSQGIKHTPVAEPPPIFG
>AtSPL11
MDCNMVSSSQWDWEHLIMSNPSRTEDDSKQLPTEWEIEKGEGIESIVPHFSGLERVSSGSATSFWHTAVS
KSSQSTSINSSSPEAKRCKLASESSPGDSCSNIDFVQVKAPTALEVSVASAESDLCLKLGKRTYSEEYWGR
NNNEISAVSMKLLTPSVVAGKSKLCGQSMPVPRCQIDGCELDLSSAKGYHRKHKVCEKHSKCPKVSVSG
LERRFCQQCSRFHAVSEFDEKKRSCRKRLSHHNARRRKPQGVFSMNPERVYDRRQHTNMLWNGVSLN
ARSEEMYEWGNNTYDTKPRQTEKSFTLSFQRGNGSEDQLVASSSRMFSTSQTSGGFPAGKSKFQLHGED
VGEYSGVLHESQDIHRALSLLSTSSDPLAQPHVQPFSLLCSYDVVPK
>AtSPL12
MEARIEGEVEGHSLEYGFSGKRSVEWDLNDWKWNGDLFVATQLNHGSSNSSSTCSDEGNVEIMERRRI
EMEKKKKRRAVTVVAMEEDNLKDDDAHRLTLNLGGNNIEGNGVKKTKLGGGIPSRAICCQVDNCGAD
LSKVKDYHRRHKVCEIHSKATTALVGGIMQRFCQQCSRFHVLEEFDEGKRSCRRRLAGHNKRRRKANP
DTIGNGTSMSDDQTSNYMLITLLKILSNIHSNQSDQTGDQDLLSHLLKSLVSQAGEHIGRNLVGLLQGGG
GLQASQNIGNLSALLSLEQAPREDIKHHSVSETPWQEVYANSAQERVAPDRSEKQVKVNDFDLNDIYIDS
DDTTDIERSSPPPTNPATSSLDYHQDSRQSSPPQTSRRNSDSASDQSPSSSSGDAQSRTDRIVFKLFGKEPN
DFPVALRGQILNWLAHTPTDMESYIRPGCIVLTIYLRQDEASWEELCCDLSFSLRRLLDLSDDPLWTDGW
LYLRVQNQLAFAFNGQVVLDTSLPLRSHDYSQIITVRPLAVTKKAQFTVKGINLRRPGTRLLCTVEGTHL
VQEATQGGMEERDDLKENNEIDFVNFSCEMPIASGRGFMEIEDQGGLSSSFFPFIVSEDEDICSEIRRLEST
LEFTGTDSAMQAMDFIHEIGWLLHRSELKSRLAASDHNPEDLFSLIRFKFLIEFSMDREWCCVMKKLLNI
LFEEGTVDPSPDAALSELCLLHRAVRKNSKPMVEMLLRFSPKKKNQTLAGLFRPDAAGPGGLTPLHIAA
GKDGSEDVLDALTEDPGMTGIQAWKNSRDNTGFTPEDYARLRGHFSYIHLVQRKLSRKPIAKEHVVVNI
PESFNIEHKQEKRSPMDSSSLEITQINQCKLCDHKRVFVTTHHKSVAYRPAMLSMVAIAAVCVCVALLFK
SCPEVLYVFQPFRWELLEYGTS
>AtSPL13
MDWNFKLSSGYLSGFDQEPDLSPMDGSISFGGSSQSKADFSFDLKLGRNIGNSSSVFGDTEQVISLSKWK
DSALAKPEGSRSSSSKRTRGNGVGTNQMPICLVDGCDSDFSNCREYHKRHKVCDVHSKTPVVTINGHKQ
RFCQQCSRFHALEEFDEGKRSCRKRLDGHNRRRRKPQPEHIGRPANFFTGFQGSKLLEFSGGSHVFPTTS
VLNPSWGNSLVSVAVAANGSSYGQSQSYVVGSSPAKTGIMFPISSSPNSTRSIAKQFPFLQEEESSRTASL
CERMTSCIHDSDCALSLLSSSSSSVPHLLQPPLSLSQEAVETVFYGSGLFENASAVSDGSVISGNEAVRLPQ
TFPFHWE

>AtSPL14
MDEVGAQVAAPMFIHQSLGRKRDLYYPMSNRLVQSQPQRRDEWNSKMWDWDSRRFEAKPVDVEVQE
FDLTLRNRSGEERGLDLNLGSGLTAVEETTTTTQNVRPNKKVRSGSPGGNYPMCQVDNCTEDLSHAKD
YHRRHKVCEVHSKATKALVGKQMQRFCQQCSRFHLLSEFDEGKRSCRRRLAGHNRRRRKTTQPEEVAS
GVVVPGNHDTTNNTANANMDLMALLTALACAQGKNAVKPPVGSPAVPDREQLLQILNKINALPLPMD
LVSKLNNIGSLARKNMDHPTVNPQNDMNGASPSTMDLLAVLSTTLGSSSPDALAILSQGGFGNKDSEKT
KLSSYENGVTTNLEKRTFGFSSVGGERSSSNQSPSQDSDSRGQDTRSSLSLQLFTSSPEDESRPTVASSRK
YYSSASSNPVEDRSPSSSPVMQELFPLQASPETMRSKNHKNSSPRTGCLPLELFGASNRGAADPNFKGFG
QQSGYASSGSDYSPPSLNSDAQDRTGKIVFKLLDKDPSQLPGTLRSEIYNWLSNIPSEMESYIRPGCVVLS
VYVAMSPAAWEQLEQKLLQRLGVLLQNSPSDFWRNARFIVNTGRQLASHKNGKVRCSKSWRTWNSPE
LISVSPVAVVAGEETSLVVRGRSLTNDGISIRCTHMGSYMAMEVTRAVCRQTIFDELNVNSFKVQNVHP
GFLGRCFIEVENGFRGDSFPLIIANASICKELNRLGEEFHPKSQDMTEEQAQSSNRGPTSREEVLCFLNELG
WLFQKNQTSELREQSDFSLARFKFLLVCSVERDYCALIRTLLDMLVERNLVNDELNREALDMLAEIQLL
NRAVKRKSTKMVELLIHYLVNPLTLSSSRKFVFLPNITGPGGITPLHLAACTSGSDDMIDLLTNDPQEIGL
SSWNTLRDATGQTPYSYAAIRNNHNYNSLVARKLADKRNKQVSLNIEHEVVDQTGLSKRLSLEMNKSS
SSCASCATVALKYQRRVSGSQRLFPTPIIHSMLAVATVCVCVCVFMHAFPIVRQGSHFSWGGLDYGSI
>AtSPL15
MELLMCSGQAESGGSSSTESSSLSGGLRFGQKIYFEDGSGSRSKNRVNTVRKSSTTARCQVEGCRMDLS
NVKAYYSRHKVCCIHSKSSKVIVSGLHQRFCQQCSRFHQLSEFDLEKRSCRRRLACHNERRRKPQPTTAL
FTSHYSRIAPSLYGNPNAAMIKSVLGDPTAWSTARSVMQRPGPWQINPVRETHPHMNVLSHGSSSFTTCP
EMINNNSTDSSCALSLLSNSYPIHQQQLQTPTNTWRPSSGFDSMISFSDKVTMAQPPPISTHQPPISTHQQY
LSQTWEVIAGEKSNSHYMSPVSQISEPADFQISNGTTMGGFELYLHQQVLKQYMEPENTRAYDSSPQHF
NWSL
>AtSPL16
MDVMALLTALVCAQGRNEATTNGSPGVPQREQLLQILNKIKALPLPMNLTSKLNNIGILARKNPEQPSP
MNPQNSMNGASSPSTMDLLAALSASLGSSAPEAIAFLSQGGFGNKESNDRTKLTSSDHSATTSLEKKTLE
FPSFGGGERTSSTNHSPSQYSDSRGQDTRSSLSLQLFTSSPEEESRPKVASSTKYYSSASSNPVEDRSPSSSP
VMQELFPLHTSPETRRYNNYKDTSTSPRTSCLPLELFGASNRGATANPNYNVLRHQSGYASSGSDYSPPS
LNSNAQERTGKISFKLFEKDPSQLPNTLRTEIFRWLSSFPSDMESFIRPGCVILSVYVAMSASAWEQLEEN
LLQRVRSLVQDSEFWSNSRFLVNAGRQLASHKHGRIRLSKSWRTLNLPELITVSPLAVVAGEETALIVRG
RNLTNDGMRLRCAHMGNYASMEVTGREHRLTKVDELNVSSFQVQSASSVSLGRCFIEPMPRFAKS
>AtSPL2
MECNAKPPFQWELENLISFGTSTAEVPRKLKPMEWEIDGFDCTSLYSSSFAYAGSSGSDIAHAFSKSSKST
SISSSSAEVRTHNFTSETGESLPGEFAKGIDTSPSLELSFGSGDPVLGLKLGKRTYFEDFWEVENAKGLGLP
VTLASSSVSPVKKSKSIPQRLQTPHCQVEGCNLDLSSAKDYHRKHRICENHSKFPKVVVSGVERRFCQQC
SRFHCLSEFDEKKRSCRRRLSDHNARRRKPNPGRTYDGKPQVDFVWNRFALIHPRSEEKFIWPSSKHVPS
RVLMPQPAKTEISDTEHNRFGLLDPKTKTARAELFSKEKVTISSHMGASQDLDGALSLLSNSTTWVSSSD
QPRRFTLDHHPSSNLQPVAHRSAAQLNSVSGYWQPDPPAVEGPTALHRNGVGQFNENYFSLNQFYN
>AtSPL3
MSMRRSKAEGKRSLRELSEEEEEEEETEDEDTFEEEEALEKKQKGKATSSSGVCQVESCTADMSKAKQY
HKRHKVCQFHAKAPHVRISGLHQRFCQQCSRFHALSEFDEAKRSCRRRLAGHNERRRKSTTD
>AtSPL4
MEGKRSQGQGYMKKKSYLVEEDMETDTDEEEEVGRDRVRGSRGSINRGGSLRLCQVDRCTADMKEAK
LYHRRHKVCEVHAKASSVFLSGLNQRFCQQCSRFHDLQEFDEAKRSCRRRLAGHNERRRKSSGESTYGE
GSGRRGINGQVVMQNQERSRVEMTLPMPNSSFKRPQIR
>AtSPL5
MEGQRTQRRGYLKDKATVSNLVEEEMENGMDGEEEDGGDEDKRKKVMERVRGPSTDRVPSRLCQVD
RCTVNLTEAKQYYRRHRVCEVHAKASAATVAGVRQRFCQQCSRFHELPEFDEAKRSCRRRLAGHNERR
RKISGDSFGEGSGRRGFSGQLIQTQERNRVDRKLPMTNSSFKRPQIR
>AtSPL6
MDSWSYGRSVFMSNETLLPCDTFAKNRRFEQRLSNNDDVLISDMAGNSNGFSAVSITKVVPEEEDEENIS
SSSKFSSQELNRIDFKLRSFLDLGNDDDDTSSRGFALPSKKSRASNLCSQNPLCQVYGCSKDLSSSKDYH

KRHRVCEAHSKTSVVIVNGLEQRFCQQCSRFHFLSEFDDGKRSCRRRLAGHNERRRKPAFYFLPGKRHK
LLRTSQDVVGNKFLENSSLVLPESFPGSLLYRVIDEDDHRTSRLVSFKDEPTCSMFPTNEQNSSRTYESKP
AIYSTEVSSIWDLHETAASRSTRALSLLSAQSQQHLSKFPNTTFSITQPNQNLNHSSSTDYHQMEQPLWID
PGKTNSAGSSSCKGKGTSTVDLLQLSSHLQRIEQQRNYTGDVKQEYNELYFPGS

>AtSPL7

MSSLSQSPPPPEMDIQPPALVNDDPSTYSSALWDWGDLLDFAADERLLVDQIHFPPVLSPPLPPLIPTQTP
AESELDPSPEESGSGSDRVRKRDPRLICSNFIEGMLPCSCPELDQKLEDAELPKKKRVRGGSGVARCQVP
DCEADISELKGYHKRHRVCLRCATASFVVLDGENKRYCQQCGKFHLLPDFDEGKRSCRRKLERHNNRR
KRKPVDKGGVAAEQQQVLSQNDNSVIDVEDGKDITCSSDQRAEEEPSLIFEDRHITTQGSVPFTRSINAD
NFVSVTGSGEAQPDEGMNDTKFERSPSNGDNKSAYSTVCPTGRISFKLYDWNPAEFPRRLRHQIFQWLA
NMPVELEGYIRPGCTILTVFIAMPEIMWAKLSKDPVAYLDEFILKPGKMLFGRGSMTVYLNNMIFRLIKG
GTTLKRVDVKLESPKLQFVYPTCFEAGKPIELVVCGQNLLQPKCRFLVSFSGKYLPHNYSVVPAPDQDG
KRSCNNKFYKINIVNSDPSLFGPAFVEVENESGLSNFIPLIIGDAAVCSEMKLIEQKFNATLFPEGQEVTAC
SSLTCCCRDFGERQSTFSGLLLDIAWSVKVPSAERTEQPVNRCQIKRYNRVLNYLIQNNSASILGNVLHN
LETLVKKMEPDSLVHCTCDCDVRLLHENMDLASDIHRKHQSPIESKVNPPSSGCCCVSSQKDIPSRILNFN
KDPEAGLDCKERIQADCSPDSGGKETDPLLNKEVVMNVNDIGDWPRKSCIKTHSALAFRSRQTMFLIAT
FAVCFAVCAVLYHPNKVTQLAVAIRMRLVHKI

>AtSPL8

MLDYEWDNPSSIVLSGDERNPDSDPTRSSFSFFDPISHYNNDHRHITISPPLLSSFSNQQQQHHLTLYGQTN
SNNQFLHHHHHHHSLYGSTTTTTPYGASDPIYHPHSSAPPASLFSYDQTGPGSGSGSSYNFLIPKTEVDFT
SNRIGLNLGGRTYFSAADDDFVSRLYRRSRPGESGMANSLSTPRCQAEGCNADLSHAKHYHRRHKVCEF
HSKASTVVAAGLSQRFCQQCSRFHLLSEFDNGKRSCRKRLADHNRRRRKCHQSASATQDTGTGKTTPK
SPNDSGVKASSSPSSNAPPTISLECFRQRQFQTTASSTSASSSSNSMFFSSG

>AtSPL9

MEMGSNSGPGHGPGQAESGGSSTESSSFSGGLMFGQKIYFEDGGGGSGSSSSGGRSNRRVRGGGSGQSG
QIPRCQVEGCGMDLTNAKGYYSRHRVCGVHSKTPKVTVAGIEQRFCQQCSRFHQLPEFDLEKRSCRRRL
AGHNERRRKPQPASLSVLASRYGRIAPSLYENGDAGMNGSFLGNQEIGWPSSRTLDTRVMRRPVSSPSW
QINPMNVFSQGSVGGGGTSFSSPEIMDTKLESYKGIGDSNCALSLLSNPHQPHDNNNNNNNNNNNNNNT
WRASSGFGPMTVTMAQPPPAPSQHQYLNPPWVFKDNDNDMSPVLNLGRYTEPDNCQISSGTAMGEFEL
SDHHHQSRRQYMEDENTRAYDSSSHHTNWSL

>OsSPL1

MSSGLKKKGLEWDLNDWRWDSNLFLATPSNASPSKCSRRELGRAEGEIDFGVVDKRRRVS
PEDDDGEECINAATTNGDDGQISGQRGRSSEDEMPRQGTCSSSGPCCQVDGCTVNLSSAR
DYNKRHKVCEVHTKSGVVRIKNVEHRFCQQCSRFHFLQEFDEGKKSCRSRLAQHNRRRRK
VQVQAGVDVNSLHENHSLSNTLLLLLKQLSGLDSSGPSEQINGPNYLTNLVKNLAALAGT
QRNQDMLKNANSAAIASHTGNYVAKGNSLHDSRPHIPVGTESTAEEPTVERRVQNFDLND
AYVEGDENRTDKIVFKLFGKEPNDFPSDLRAQILSWLSNCPSDIESYIRPGCIILTIYMR
LPNWMWDKLAADPAHWIQKLISLSTDTLWRTGWMYARVQDYLTLSCNGNLMLASPWQPAI
GNKHQILFITPIAVACSSTANFSVKGLNIAQPTTKLLCIFGGKYLIQEATEKLLDDTKMQ
RGPQCLTFSCSFPSTSGRGFIEVEDLDQSSLSFPFVVAEEDVCSEIRTLEHLLNLVSFDD
TLVEKNDLLASRDRALNFLHEFGWFLQRSHIRATSETPKDCTEGFPAARFRWLLSFAVDR
EFCAVIKKLLDTLFQGGVDLDVQSTVEFVLKQDLVFVAVNKRSKPLIDFLLTYTTSSAPM
DGTESAAPAQFLFTPDIAGPSDITPLHIAATYSDTAGVLDALTDDPQQLGIKAWKNARDA
TGLTPEDYARKRGHESYIEMVQNKIDSRLPKAHVSVTISSTTSTTDFTEKHASQSKTTDQ
TAFDVEKGQQISTKPPLSCRQCLPELAYRHHLNRFLSTRPAVLSLVAIAAVCVCVGLIMQ
GPPHIGGMRGPFRWNSLRSGPK

>OsSPL2

MDWDAKMPSWDLGTVVGPSGGGGGGGGGGGALDLKLGAPTSWKTTTTVSAASAAPAAVAP
PPPPPASSSSSAAAAGKRARAGQGQQAAVPACSVEGCAADLSKCVRDYHRRHKVCEAHSK
TAVVTVAGQQQRFCQQCSRFHLLGEFDEEKRSCRKRLDGHNKRRRKPQPDPLNPGNLFAN
HHGAARFTSYPQIFSTAASMSPQETKWPANVVKTEAADVFQEPYYHALHLNGAGAAAAAS
IFHHGGNKARKHHFPFLTADHGGGAAAASPLFGCQPFTITPSSESRSSSSSRHSNGKMFA

HDGGLDNCALSLLSDNPTPTAQITIPQPLFAGGGQYGGGGGGDVSLTGLSYVRMAGKDTS
ILAKSATTTATTATTPTTTSAQLQYHGYYHHHVSADQGSSDAAIQALPFSSW
>OsSPL3
MGSFGMDWNQKSSVLWDWENMPPIGNSANENPKNVMLAESKLAGVGVDIGHESGHSSGGT
FSSSSEIGYGSSKSSISASIDSPSKVGNTIELNFASAEEHDKNMDKGKSKVDDTGTSRSP
VVAANRVEPLIGLKLGKRTYFEDVCGGQNVKSSPSGVSVATPSPGLAKKVKVAQQNTQNP
HCQVEGCNVDLSSAKPYHRKHRVCEPHSKTLKVIVAGLERRFCQQCSRFHGLAEFDQKKR
SCRRRLHDHNARRRKPQPEAISLSSSRLSTLLYGDARQQASFLFGQAPYGQMGSCASSWD
NPVPGGFKFTATKAPWSRPTIAAGVDGTHVSNQQASGNVLPHGAHHSFDGLMAFKETNAK
VLNQGMEASAVASGSARGPDFEHALSLLSIDSVGAANLQPGSQIHPGVTAIAGTSNPVMM
PSPAIWQGGLSLDQQAQFQAFDRLGNDDDEDHLQLPKPSYDNSHYDQMN
>OsSPL4
MDWMPPPKPTSPRSPPLLWDWADAAVPGSSSGEVSAAAAAAAAHPGRRRKEKRGRAEEGG
GGGGEVRCQVEGCGVELVGVKDYHRKHRVCEAHSKFPRVVVAGQERRFCQQCSRFHALSE
FDQKKRSCRRRLYDHNARRRKPQTDVFSYASARPPSSLLFDDNRQISFVWNKAPLSHVRP
FAISPWESSSEVGTTDGHIYLDKSHISKSLPAFNTDIDELLPMKDFSAATIWMFFGVLFI
RTAQESCMSFH
>OsSPL5
MAVPAAAVAADVVDFGYAAPMPPPYVGFDPAGMGGERQLFQHGGACHGLYDGGLDFSAAA
AFQEAATLGVGLPGGNLLQSLAPPAAAAATPSSLQMPMMMSLPGLPATAADVYPFGGGGF
VKREDGPVLDVVGGGGGGRIGLNLGRRTYFSPADVLAVDRLLLRSRLGGMGMEMGMGMGV
LGLGLAAAAHHHQPPRCQAEGCKADLSAAKHYHRRHKVCDFHAKAAAVLAAGKQQRFCQQ
CSRFHVLAEFDEAKRSCRKRLTEHNRRRRKPTAGGQSSKDSPPPPPSKKGTDASIASSYT
SCDHHKAAASTTTASGVSCLQELADHHDVGGGHQAAMAAAPPPTLSLAALPPQEEDDEDE
DGGLGNVLMMQQHHQRRRLQHDGDGDDDVAAAAAHHHLMRSLARQQQQHRHSSGCSNNND
GDDDDHNNNNNILSCSSASDQQNSSNNNNNMHFFEVDFI
>OsSPL6
MEAARVGAQSRHLYGGGLGEPDMDRRDKRLFGWDLNDWRWDSDRFVATPVPAAEASGLAL
NSSPSSSEEAGAASVRNVNARGDSDKRKRVVVIDDDDVEDDELVENGGGSLSLRIGGDAV
AHGAGVGGGADEEDRNGKKIRVQGGSPSGPACQVEGCTADLTGVRDYHRRHKVCEMHAKA
TTAVVGNTVQRFCQQCSRFHPLQEFDEGKRSCRRRLAGHNRRRRKTRPEVAVGGSAFTED
KISSYLLLGLLGVCANLNADNAEHLRGQELISGLLRNLGAVAKSLDPKELCKLLEACQSM
QDGSNAGTSETANALVNTAVAEAAGPSNSKMPFVNGDQCGLASSSVVPVQSKSPTVATPD
PPACKFKDFDLNDTYGGMEGFEDGYEGSPTPAFKTTDSPNCPSWMHQDSTQSPPQTSGNS
DSTSAQSLSSSNGDAQCRTDKIVFKLFEKVPSDLPPVLRSQILGWLSSSPTDIESYIRPG
CIILTVYLRLVESAWKELSDNMSSYLDKLLNSSTGNFWASGLVFVMVRHQIAFMHNGQLM
LDRPLANSAHHYCKILCVRPIAAPFSTKVNFRVEGLNLVSDSSRLICSFEGSCIFQEDTD
NIVDDVEHDDIEYLNFCCPLPSSRGRGFVEVEDGGFSNGFFPFIIAEQDICSEVCELESI
FESSSHEQADDDNARNQALEFLNELGWLLHRANIISKQDKVPLASFNIWRFRNLGIFAME
REWCAVTKLLLDFLFTGLVDIGSQSPEEVVLSENLLHAAVRMKSAQMVRFLLGYKPNESL
KRTAETFLFRPDAQGPSKFTPLHIAAATDDAEDVLDALTNDPGLVGINTWRNARDGAGFT
PEDYARQRGNDAYLNMVEKKINKHLGKGHVVLGVPSSIHPVITDGVKPGEVSLEIGMTVP
PPAPSCNACSRQALMYPNSTARTFLYRPAMLTVMGIAVICVCVGLLLHTCPKVYAAPTFR
WELLERGPM
>OsSPL7
MEGNGCGGSGATPRGVVGMHWAPVVTSPPSPQPPFLPPAPCRPDVQMQQQGGLTCLKLGK
RPCFWGGDGAGQVAQGSGGGGGGGGGGSADQGKRKEKAATAVPVVPRCQVEGCDITLQGV
KEYHRRHKVCEVHAKAPRVVVHGTEQRFCQQCSRFHVLAEFDDAKKSCRRRLAGHNERRR
RSNASEAMARGSAHPHGMPVLGHGFPPYGLPTSSAGALSLLSSARATGPWLMPTPDISAR
SSAALDELIAENRAALLSWQFFSDRQPPPAGRPTGRSPGSETAGGWHAHLQARPPPPGAG
GQHENQSGHVTLDLMQATTAAGGSGAPFRPVPARPPKEGGDAGCTSDAWTPSPMEGARVV
>OsSPL8

MMNVPSAAAASSCDDFGYNATPPPPPSLLPIMDQDGGGGSIQRDHHQHHNHQQLGYNLEP
SSLALLPPSNAAAAAAHHATIAHASPHDLLQFYPTSHYLAAAGGAGGGGNPYSHFTAAAA
AGSTFQSYYQQPPQDAPEYYFPTLVSSAEENMASFAATQLGLNLGYRTYFPPRGGYTYGH
HPPRCQAEGCKADLSSAKRYHRRHKVCEHHSKAPVVVTAGGLHQRFCQQCSRFHLLDEFD
DAKKSCRKRLADHNRRRRKSKPSDGEHSGEKRRAQANKSAATKDKAGSSSKNAGIGDGFE
TQLLGGAHMSKDQDQAMDLGEVVKEAVDPKGKASMQQQQQAHHGIHQQSHQQHGFPFPS
SSGSCLFPQSQGAVSSTDTSNIAQVQEPSLAFHQQHHQHSNILQLGQAMFDLDFDH
>OsSPL9
MDAPGGGGGGGGGGGVDAGEPVWDWGNLLDFAVHDDDSLVLPWGDDSIGIEADPAEAAL
LPPAPSPQPAEAEAEAAGPASLPSSMQAEGSKRRVRKRDPRLVCPNYLAGRVPCACPEID
EMAAALEVEDVATELLAGARKKPKGAGRGSGAAVGGSGGGASRGTPAEMKCQVPGCEADI
RELKGYHRRHRVCLRCAHAAAVMLDGVQKRYCQQCGKFHILLDFDEDKRSCRRKLERHNK
RRRRKPDSKGILEKDIDDQLDFSADGSGDGELREENIDVTTSETLETVLSNKVLDRETPV
GSDDVLSSPTCAQPSLQIDQSKSLVTFAASVEACLGTKQENTKLTNSPVHDTKSTYSSSC
PTGRVSFKLYDWNPAEFPRRLRHQIFEWLSSMPVELEGYIRPGCTILTVFVAMPQHMWDK
LSEDTGNLVKSLVNAPNSLLLGKGAFFIHVNNMIFQVLKDGATLTSTRLEVQSPRIHYVH
PSWFEAGKPIDLILCGSSLDQPKFRSLVSFDGLYLKHDCRRILSHETFDCIGSGEHILDS
QHEIFRINITTSKLDTHGPAFVEVENMFGLSNFVPILVGSKHLCSELEQIHDALCGSSDI
SSDPCELRGLRQTAMLGFLIDIGWLIRKPSIDEFQNLLSLANIQRWICMMKFLIQNDFIN
VLEIIVNSLDNIIGSELLSNLEKGRLENHVTEFLGYVSEARNIVDNRPKYDKQRQVDTRW
AGDYAPNQPKLGISVPLAESTGTSGEHDLHSTNAASGEEENMPLVTKALPHRQCCHPETS
ARWLNAASIGAFPGGAMRMRLATTVVIGAVVCFAACVVLFHPHRVGVLAAPVKRYLSRNY
SS
>OsSPL10
MMSGRMNAAGDESPFPFGAMQAPGPGAYVGFDHGAAAVAAAAAAAQRAGMLQHHHHHMYD
GLDFAAAMQFGGGQDAPPHPQLLALPPSMAAPPPPPMPMPLQMPMTMPMPGDVYPALGIV
KREGGGGGGQDAAAGRIGLNLGRRTYFSPGDMLAVDRLLMRSRLGGVFGLGFGGAHHQPPR
CQAEGCKADLSGAKHYHRRHKVCEYHAKASVVAASGKQQRFCQQCSRFHVLTEFDEAKRS
CRKRLAEHNRRRRKPAAAATTAVAAAKDAAAAPVAAGKKPSGGAATSYTGDNKNVVSMSA
AKSPISSNTSVISCLPEQGKHAAAAARPTALTLGGAPPHESSAPQIGAMLHHHHHHQQDH
MQVSSLVHINGGGGGGSNNILSCSSVCSSALPSTATNGEVSDQNNDNSHNNGGNNNNMHL
FEVDFM
>OsSPL11
MECNPVSSTTSSSLLWDWDATASAEPPPPPGKRGGRDSSSASASAKRGRSAAAAGDAAAV
AAEAPRCQVEGCGLELGGYKEYYRKHRVCEPHTKCLRVVVAGQDRRFCQQCSRFHAPSEF
DQEKRSCRRRLSDHNARRRKPQTDVFAFGSGTLPRSLFDDRQQISFAWDNNAPLNHANTT
SSSSWTSDLQLSQVMDISKRSRKAGADSANIRLSNALPTLCHDTNELLPIKGADASETAS
KLDGALDVQRALSLLSASSRGLTDPGHQTSSIIQFTNSNQNSTLPSVPSEGNSNVPFWVD
GQHQAVEPQVFQFTMDTGNTVFPDLERIKPSYESSMFGLNQIH
>OsSPL12
MASFGMNWNQKSPVFWDWENPAPFGPNTMENPKSIPHPEPRGVVVAAANHGSTNSSGGTF
TSSSELANGSSKSSLSASFDSSSKLGNSLEFRFASVKGHGKNMCKDGEAGRVEDSGTSPA
VAVSHGEPVIGLKLGKRTYFENVCGGQNVKSSSAASGVTCPSTVVKKMKVSQQSTQSSYC
QVEGCKVDLSSAREYHRKHKVCEAHSKAPKVIVSGLERRFCQQCSRFHGLAEFDQKKKSC
RRRLSDHNARRRKPQQEAISFGSSRLATMFYDARQQTDIYFGQSPFGQVRSNAISSCDNL
GGFKFTEAKLPWMKPMKTIGLEDLNFSTLQMPGNVVSHTVHHHDFDGLIPFKGNTPKVLN
QGVDPACAVVSSNSNGAPDLRRALSLLSSDSWGPADVQAGSQVHPGGVMPPLAVAAATVT
APTNPVSVMHALHPSTGGGGFWQDGDDPPPLDHASQAQAFMHPGNGSSSGYGHLH
>OsSPL13
MDRKDKARKNFSSSSSSSAASMAALAAAAAAGDGGAALPSPMEEDKKPRLVASSLAPVAG
GGGGGSSSSAAVAAGASSSSSSSSVAAAARRGAGRAGGGAPSGGGGGPRCQVERCGVDLS
EAGRYNRRHKVCQTHSKEPVVLVAGLRQRFCQQCSRFHELTEFDDAKRSCRRRLAGHNER

RRKSAADTAHGENCRHADQDAGRSHQGTGNPPFQIR
>OsSPL14
MEMASGGGAAAAAGGGVGGSGGGGGGGDEHRQLHGLKFGKKIYFEDAAAAAGGGGTGSGS
GSASAAPPSSSSKAAGGGRGGGGKNKGKGVAAAAPPPPPPPPRCQVEGCGADLSGIKNYY
CRHKVCFMHSKAPRVVVAGLEQRFCQQCSRFHLLPEFDQGKRSCRRRLAGHNERRRRPQT
PLASRYGRLAASVGEHRRFRSFTLDFSYPRVPSSVRNAWPAIQPGDRISGGIQWHRNVAP
HGHSSAVAGYGANTYSGQGSSSSGPPVFAGPNLPPGGCLAGVGAATDSSCALSLLSTQPW
DTTTHSAAASHNQAAAMSTTTSFDGNPVAPSAMAGSYMAPSPWTGSRGHEGGGRSVAHQL
PHEVSLDEVHPGPSHHAHFSGELELALQGNGPAPAPRIDPGSGSTFDQTSNTMDWSL
>OsSPL15
MQREVGPQVAPPMFLHQIQPLPPHATAAKKRGNPWPAAAVAAAEAKGGGNWNPRMWDWDS
RALTAKPSSDALRVNAGLSHHQQQQQQSPPAAAKAAEALRQGGGGSGGLNLQLGLREDAA
TPMDVSPAATTVSSSPSPPASSAPAQEPVVRPSKRVRSGSPGSASGGGGGGGGGGGNSGGG
GGSYPMCQVDDCRADLTNAKDYHRRHKVCEIHGKTTKALVGNQMQRFCQQCSRFHPLSEF
DEGKRSCRRRLAGHNRRRRKTQPTDVASQLLLPGNQENAANRTQDIVNLITVIARLQGSN
VGKLPSIPPIPDKDNLVQIISKINSINNGNSASKSPPSEAVDLNASHSQQQDSVQRTTNG
FEKQTNGLDKQTNGFDKQADGFDKQAVPSTMDLLAVLSTALATSNPDSNTSQSQGSSDSS
GNNKSKSQSTEPANVVNSHEKSIRVFSATRKNDALERSPEMYKQPDQETPPYLSLRLFGS
TEEDVPCKMDTANKYLSSESSNPLDERSPSSSPPVTHKFFPIRSVDEDARIADYGEDIAT
VEVSTSRAWRAPPLELFKDSERPIENGSPPNPAYQSCYTSTSCSDHSPSTSNSDGQDRTG
RIIFKLFGKEPSTIPGNLRGEIVNWLKHSPNEMEGYIRPGCLVLSMYLSMPAIAWDELEE
NLLQRVNTLVQGSDLDFWRKGRFLVRTDAQLVSYKDGATRLSKSWRTWNTPELTFVSPIA
VVGGRKTSLILKGRNLTIPGTQIHCTSTGKYISKEVLCSAYPGTIYDDSGVETFDLPGEP
HLILGRYFIEVENRFRGNSFPVIIANSSVCQELRSLEAELEGSQFVDGSSDDQAHDARRL
KPKDEVLHFLNELGWLFQKAAASTSAEKSDSSGLDLMYFSTARFRYLLLFSSERDWCSLT
KTLLEILAKRSLASDELSQETLEMLSEIHLLNRAVKRKSSHMARLLVQFVVVCPDDSKLY
PFLPNVAGPGGLTPLHLAASIEDAVDIVDALTDDPQQIGLSCWHSALDDDGQSPETYAKL
RNNNAYNELVAQKLVDRKNNQVTIMVGKEEIHMDQSGNVGEKNKSAIQALQIRSCNQCAI
LDAGLLRRPMHSRGLLARPYIHSMLAIAAVCVCVCVFMRALLRFNSGRSFKWERLDFGTI
>OsSPL16
MEWDLKMPPAASWELADELENSGGGGVPAAVSSSSAAVGGGVNAGGGGRQECSVDLKLGG
LGEFGGGGAQPRVAVAGEPAKGKGPAAAATGAAAAASSAPAKRPRGAAAAGQQQCPSCAV
DGCKEDLSKCRDYHRRHKVCEAHSKTPLVVVSGREMRFCQQCSRFHLLQEFDEAKRSCRK
RLDGHNRRRRKPQPDPMNSASYLASQQGARFSPFATPRPEASWTGMIKTEESPYYTHHQI
PLGISSRQQHFVGSTSDGGRRFPFLQEGEISFGTGAGAGGVPMDQAAAAAAASVCQPLLK
TVAPPPPPHGGGGSGGGKMFSDGGLTQVLDSDCALSLLSAPANSTAIDVGGGRVVVQPTE
HIPMAQPLISGLQFGGGGGSSAWFAARPHHQAATGAAATAVVVSTAGFSCPVVESEQLNT
VLSSNDNEMNYNGMFHVGGEGSSDGTSSSLPFSWQ
>OsSPL17
MATGGSGGGGGGGGGGDDVHGLKFGKKIYFEQDAAASASAAAVESSSTSSGGGGKKGKGV
AAAAAPPPPLPPRCQVEGCGVDLSGVKPYYCRHKVCYMHAKEPIVVVAGLEQRFCQQCSR
CSVHMVRFHQLPEFDQEKKSCRRRLAGHNERRRKPTPGPLSSRYGRLAASFHEEPGRSRS
FVVDFSYPRVPSSVRDAWPAIQPSDRMSGSIQWQGGHELHPHRSAVAGYSDHHAFSSHGG
SAAGAPMLHHPAFELTSGGCLAGVATDSSCALSLLSTQPWDTTQSTSSHNRSPPMSSTAS
AFGGGNNPVSPSVMASNYMAASPGWNSSSRGHDGARNVHLPPPHGVVLNEVPPGSVHHGH
FSGELELALQGGAPSNRPEAEHGSGSGAFSHSTNAMNWSL
>OsSPL18
MDWDLKMPVSWDLAELEHNAVPNMAAAASAAEPGIAAVAASRGAPGRPECSVDLKLGGLG
EFGAADALKEPAAAAKAPVSSAAAAASVAKVPPSTSTLKRPRGGGGGGGGGQCPSCAVDGC
KADLSKHRDYHRRHKVCEPHSKTPVVVVSGREMRFCQQCSRFHLLGEFDEAKRSCRKRLD
GHNRRRRKPQADSMSSGSFMTSQQGTRFASFTPPRPEPSWPGIIKSEETPYYSHHHHPHP
VMTSRQPHFVGSPSSATTAAFSPKEGRRFPFLHEGDQISFGGGGGAAAAATLEISVCQTT

VVAPPPPESSSSNKMFSSDGLTTATTTTTAHHHHHHHQVLDSDCALSLLSSPANSSSVD
VSRMVQPSPAAAAGAEHHHHHQIPMAQPLVPNLQQQFGGSSPWFASSPAAAAVAGGGFAC
PSMDSEQQQQQQLNAVLVPGSNENEMNYHGMFHVGGEGSSDGTSPSLPFSWQ
>TaSPL1
MSGGLKNKKKKGHEWDLNDWRWDGNLFLATPSPNADAPSGCGSR
ELGRAGEGCSFGAAADKSRRRRRRRVTTVDNPEECSNTAIHNERNAVWRGQIGEEEGP
ASATAGASSSSAPSCQVDGCHADLSDDRDYHRRHKVCEPHTKSTLVRIKNIEHRFCQQ
CSRFHLVQEFDEGKKSCRSRLATHNRRRRKAPAEAVNSLGENQSLTNTLLLLLRQLAG
QDSASSSEQINGPNLLVSLLKNLAAVAGTQACQDMLKDATSSNAGNYVGNQSGPPVPA
EEPPVKRRAQNFDLNDAYVEEDESRTDKIVFKLFGKQPNDFPADLRAQILNWLSHYPS
DMESYIRPGCVILTIYLRLPNWMWGKINVDPAPWIENLISISTHGFWETGWLYARVQD
RLTLSCNGRLMLVSPWRPVIGDKHQILCVTPI
>TaSPL3
MEWNQRSSVLWDWENFPPIGENPKNAMQADPRFAAVAATMGNEP
LHSSGGSGTFSSSSEMGYGSSKSSMSASIDSSNRAGNNMEFRFAPVKNPDRNTSKNTE
LGKVDNTRTGTSPSPVVAVSSGEPVIGLKLGKRTYFEDVCGGQNVKSSPSGAASAPNK
SPALGKKAKAEQQKPHNSYCQVEGCKVDLSSVKDYHRKHRVCELHSKAPKVVVAGLER
RFCQQCSRFHALAEFDQKKRSCRRRLNDHNSRRRKPQPEAISFSSSRMSTMFYDARQQ
PNFLFGQAPYVQMRSCGSSSWDDPGGFKVTHTKAPWLKPTTAAGVHGIHLSSQQMSDN
IMPHGAHHGFDGFMAFKGTCTKFPNQGVQASAVASDSSGAPDLQHALSLLSSNPVGAA
NLQPSPQMHSGVAAIAGAPNPAMHALGSSTGLWLDGSQPLDDHPRFQVFERLGDHDSE
LQLPKPSYDHASHFDRMH
>TaSPL6
MEAARVGSQSRHLYGGGLGGELEQARREKRVFGWDLNDWSWDSE
RFVATPVPAAVGNGLSLNSSPSSSEEAEAEVARNGNGRVDSDKRKRVVVIHDDDNEKD
EDPVGNSNNVLSLRIGGNTVAGGVAEDGGVNEEDRNGKKIRVQGGSSSGPACQVEGCC
ADLSAAKDYHRRHKVCEMHAKANTAVVGNTVQRFCQQCSRFHLLQEFDEGKRSCRRRL
AGHNKRRRKTRPENAVGGTPIEEKVSSYLLLSLLGICANFNSDNAEHLQGQELLSNLW
RNLGNVAKSLDPKELCKLLEACQSMQNRSNAGTSEAANALVNTAAAEAAGPSNSKAPF
ANGGECGQTSSAVVPVQSNATMVATTETPACKFRNFDLNDTCNDMEGFEDGSNCPSWI
RQDSTQSPPQTSGNSDSTSAQSLSSSNGDAQCRTDKIVFKLFEKVPSELPPILRSQIL
GWLSSSPTDIESHIRPGCIILTVYLRLVESSWRELSENMSVYLDKLSSSSADNFWTSG
LVFVMVRHQIAFMHNGQVMLDRPLAPNSHHYCKVLCISPVAAPYSATVNFRVEGFNLV
SSSSRLICSIEGRCIFEEDTAIMADDAEDEDIEYLNFCCSLPDTRGRGFIEVEDSGFS
NGFFPFIVAEQNVCSEVCELESTFKSSSLEQPDKDNAMNQALEFLHELGWLLHRVNII
SKHDKVESPVPTFNLLRFRNLGIFAMEREWCAVTKMLLDLLFDGFVDAGLQSPKEVLL
SENLVHSAVRGKSARMVRFLLTYKPNKNLKETAETYLFRPDARGPSAFTPLHIAAATN
DAEDVLDALTDDPGLVGLNAWRNARDEIGFTPEDYARQRGNDAYINLVQKKIDRHLGK
GHVVLGVPSSMCPGITDGVKAGDISLEICKAMPMTTTSASRCNICSRQAKMYPNSFAR
TFLYRPAMFTVMGVAVICVCVGILLHTLPKVYAAPNFRWELLERGAM
>TaSPL8
MMNLPAAAGNSCDEFGYGAPNPPPPSLFPIMDHQEGGGGIHREH
DHHHHHLGGYTLEPSSLALLPPSSHATIAAHSPHDILQFYHHPTSHHYLAAAAAGGNG
SPYGQFGGGGTAGGFQSYYHQQVGTGAPEYYFPTLVSSAEENMASFAATQLGLNLGYR
TYFPPRGGYTYGHHPPRCQAEGCKADLSGAKRYHRRHKVCEHHSKAPVVVTAGGLHQR
FCQQCSRFHLLDEFDDAKKSCRKRLADHNRRRRKSKPSEADAADKRRTQASKAASTKG
KAAGSSSKSTGTGDGMDIQVQLGSGDLSKNQDETMGLGEVVKEMQVDPKGKASMQQQQ
GHHGHGLHLQSQHGFPFPSSSAGSCFPQSQAVSSTHNTSNIGQVQQEQPGLGFHQHSN
ILQLGQAMFDLDFDH
>TaSPL15
MAMDVSPTTTLSSSPSPPARASQEQAARPSKRVRSGSPGTASGG
GGGGGGGGSASGGGSYPMCQVDDCRADLTSAKDYHRRHKVCEIHSKTTKAVVGNQMQR

FCQQCSRFHPLSEFDEGKRSCRRRLAGHNRRRRKTQPTDVASQLLLPENQENAANRTQ
DIVNLITVIARLQGGNVGKLPSIPPIPDKDNLVQIISKINSINNANSLAKSPPSEAID
LNASQGQQQDSSVQNATKVVDKQTVPSTMDLLTVLSGALGTSTPETNTSQSQGSSDSS
GNNKSKSHSTEPACVVNSHEKSIRPFPAAGMLRSSSTHGSPPEVYKQPDRDTHPYLSL
QLFGNNEDIPVKMDTANKYLSSESSNPMDERSPSSSPPVTHTFFPTRSVNEGIRHPRI
ADYGEDGATVENSTTRAWCAPPLELFKDSERPTENGSPPNPTYQSCYASTSGSDHSPS
TSNSDGQDRTGKIIFKLFGKEPGSIPGNLRDEVVNWLKHSPTEMEGYIRPGCLVLSMY
LSMPTIAWDELQENFLQRVNSLVQASDLDFWRKGRFLVRSDNQLVSYKDGMTRLSKSW
RTWNTPELTLVTPIAVVGGRKTSLVLKGRNLTIPGTQIHCTSAGKYISKEVLCSAYPG
TIYDDSGVETFDLPGEPSLTLGRCFIEVENRFRGNSFPVIFANKSICQELRNLEDELE
DSRFPDVSPDDQVHDARRLKPRDQVLHFLNELGWLFQKAAACTPSTKSDVSHSELIQF
STARFRHXLLFSNERDWCSLTKTLLEILTKRSMVSDELSHETLEMLSEIHLLNRAVKR
KSSHMVHLLVQFVVICPDNSKLYPFLPNYPGPGGLTPLHLAASIDDAEDIVDALTDDP
QQIGLSCWHSVLDDEGLSPEVYATFRNNGSYNELVTRKLMDRKNSQVTIVLNKGEIHI
DQPGNVGANNASGIQALEIRSCNQCAILESGLLRRPMRSRGLLARPYIHSMLAIAAVC
VCVCVFMRALLRFNSGRSFKWERLDFGPT
>TaSPL17
MEIGSGGDGSGGGGGDDQQRHGLKFGKKIYFEDATGTGGGSGSG
GVNASLSKPPAGSGRKGKAVAAGGASGSAPPRCQVEGCGVDLSGAKQYHSRHKVCSMH
TKEPRVVVAGLEQRFCQQCSRFHQLPEFDQGKRSCRRRLAGHNERRRKAPPGPLATRY
GRLAASFEEPGRFRSYLLDFSYPRVPSSVRDAWPGARPGYRMPGEIQWQGNLDLRPHT
GAATGYHGHHAYSSHGGGFPGPELPPSGCLAGVAADSSCALSLLSTQPWDNTPHGASH
DHRSAGFDGHPVGVSPSIMASNYMPPPASPWGGSRGHEGGRNAPHQQLPHDVPLHEAH
HPAGSSQHGHFSGELELALQGYRPAPGPRGGDHSSGGGAFDHPGSSSNWSL
>TaSPL20
MMSGGRMNAAASDDFPFAAMQPPPYVGFEHAGGGGQRHQGAMMY
DNFDFAAAADAFGQFQDAPHHQMLALPPSGNGAGGLVPMAPPPMPGMQLQMPPMAMHG
HGDVYPAMGMVKREGDAGRIGLNLGRRTYFSPGDMMAVDRVLMRSRLGGVFGLGFGRG
HHQAPRCQVEDCRADLSGAKHYHRRHKVCEYHAKASLVSAAGKQQRFCQQCSRFHVLT
EFDEAKRSCRRRLAEHNRRRRKPAAGSTAASSKDSAPPSKKPNAGAISSSYTADNNTL
STTKSTISSNTSAISCLQQGQAGAVAPTRPTTLTLGASPENDDHQQQLHHHQEQQHFI
TSLLHNNINNSNILSCSSVSSSTMPSAAAANGEVSDQNNDNANNNMHMFEVDFM
>TaSPL21
MMSSRLSGGTMAPVSDMTDFGYAPMQSYPNFEPAGMVMPGDRQP
PFQHHHLYDSLDFNAAAFSFQDPVALFSSGSAFSNQLQQPFLQTQVSMPTMASSSLLQ
APMMTLPGMLTSSSASPVDAYTFGGGGSAGFLKREEGGPFSDVGGGGRIGLNLGRRTY
FSPADVLAVDRLLMRSRFGGAGAMGMLGLGLGAAAHQHQTPRCQAEGCKADLSAAKHY
HRRHKVCEYHAKAATVAASGKQQRFCQQCSRFHVLAEFDEAKRSCRKRLTEHNRRRRK
PAGAQGKDSPPPSKKADASITSSYTGDHRTNKSTTGAAFSPSASGFSCLQQQQQQQH
EIDNGGQSSNATPTNLSLAAPPPPPPPQDDAGFGAGLDTMLLIQQQGPDEQEDEQQQQ
QHFMMTSLVQSHRQQQQQGDRGNILSCSTTSPSDQRRHQNDGDSCCNGNSMQHFFEVE
FM
>TaSPL22
MMSGGRMNAAAGDDFPFAAMQQPPYVGFEHAGMVQAGGGGGGGG
QRHHQGAMMYDNFDFAAAAAAFGQFQDAPHHQMNGGGAGGLLPMAPPPMPGMQLQMPT
MGMHGHGDVYPALGMVKREGGGGAGDAGRIGLNLGRRTYFSPGDMMAVDRLLMRSRLG
GVFGLGFGGAHHQPPRCQAEGCKADLSGAKHYHRRHKVCEYHAKASLVAAAGKQQRFC
QQCSRFHVLTEFDEAKRSCRKRLAEHNRRRRKPASSSTTATSKDSAPPSKKPNPGAIS
SSYAADNNNLSATKSTISSNTSAISCLQQHDQSKAAAAAARPMTLTLGEPREKDDHQH
LSNAMQLHHHHQEQQHFITSLLQQNNNGNGNSNILSCSSVCSSGLPSAGAANGEVSDQ
NNNSNHGGGNNNNNNMHLFEVDFM
>TaSPL23

MDSWEHKMPSWDLSTVVAPSGGGGGGGGGALDLKLGAPTSWRPV
PAAAVASVQQQQQPPPPSAAPAPAKRARAGQGQQTVPPCSVEGCTADLSRCREYHRRH
KVCEAHSKTPVVAVAGQQQRFCQQCSRFHLLGEFDEVKRSCRKRLDGHNRRRRKPQPD
PLNPAGLFANHHGTPS

Table S3. Genomic sequences of 17 barley SPL genes.

>HORVU0Hr1G039170.6 genomic sequence
ATTTCTCGGGGCAAGGTCTGGTTGTAGTAGTACTATTATATTTATACAGTATGATTATTA
CGCGTGACTAACTTTTAATAAAAACAAGTGAAAACAACAAGCAACAACCCTAGACTAGTA
GAGTAGTAGGAAGGAACAGCTCCGTTTTGTTTCTAGACTAGGCTAGTGTAATGCTTGCAA
CTGCAAGCTTGCCACTAAGGAAAAGCCCAGAATACAAAATGATTTCATTGATATCACGGC
ACAAATGTGTGGCTCACGACCTGCCAAACAAGGAAACAACCTGTGGACTGTGGTCGGCTT
GATTAGCTTTAGTGATGCGTGTGTTATTTTAACCTGTTGAGCCATGCAGAAAGGAAGTGG
TCAGCTAGGCCGGCTAGGGGCAACTTGTGTTTCTGATAAACATGCATGCCCCCTTCAGAC
AGATAGCACTTGGCCAAGAAAATATTCTACTTATAAAATCAGACGATCCCTCCCGTGTAG
TATACGTACCAGTAGGAAAGAAGAAGAAAGGTAGTAGTAAAAAAACCAAACCTCCAATAA
TTGTAGCACGACCCTTCCATGGCAGTGTTAAAGGCGAACATGGCCCGTCTCCATGCCTGT
CAGGGATTGAATGGCTGCAACCCCAGCGACACAAATCATCTCTCTCCCTCTCTCCCTCTC
TCGGCGCAAGTACTTGTACTGACAACACCCCAGCTTAGCTTGTATTGTTGGCCCATATCT
TTGTTGCTCGTGGCACCCTCCCCGGACATTGGACGGGCGCCATTGCTGCTGTTTTTATTT
GACATCTTTTGTTTGGTGCGGCAATTAACCCACAGATCCAGCGCCCGCTACCGCTAGCCA
GTAGCCAGTGGGTGGTATGGTAGCCTAGAGTAGAGAGTAGAGACGGTACTGCTGCCAGGC
AGCCTCCTTGCAAAACAGCCACCCTCGGCAGCTCGGAATGAGAGGGACAGGGGTGAGAGC
GAGGGGCGAGGCCGAGGCCCAACACACAAGCACAAGCTCCAGACAGCGACCCGAGTTGCC
TTTGCCTGTGGCTGTGTGGGTGTGTGGGAGATGAAAGCGAGGGCTCGGCCATAACTAGGG
ATCGAGCTACTGCCCAGCTAGCTGCTCCTGCTCGGCCTAGGCCTACGCCAACCGGGCTCA
GCTGAGGCTGAGGCTGGCTTGCGCTGTACGCGTCCGTGTCCTCCGCGCGCCGGCAGGGGG
TGACTACGTGAGCGTGAGCGTGAGCGCGAGCGCGGCATGGACTGGGATCTCAAGATGCCG
CCCGGCGCATGGGACCTCGCCGACCTGGAGAGCGACGCCGCGCCGGCGCCGGCCGGTGGC
ATTGCTAATGCGGAGTGCTCCGTGGACCTGAAGCTCGGCGGGCTCGGCGAGTGCGGCGCT
GCCCCGGACACCCGCGGCCTCGGCAAAGCGCCGGCCGAGGCGGCGGCGTCGCCCAGCGCG
GCCAAGCGCCCGCGCGCGTCGTCGGGAGGCGGCGCGGGGCAGCACCAGCAGTGCCCGTCG
TGCGCGGTGGACGGGTGCAGGGCCGACCTGAGCAGGTGCCGCGACTACCATCGCCGCCAC
AAGGTGTGCGAGGCGCACTCCAAGACCCCCGTCGTCACCGTCGCCGGCCGCGAGATGCGC
TTCTGCCAACAGTGCAGCAGGTAATTGTTTCACCCGCCAATTCTCTTCCAACCACGCCAC
CACCCGCCGCTTAGTAGGAGATGGAGCAAATCAAATCGTAGCTCTTCTTCTTTTTTTCTT
TTTTCGCATCATCATCATTCTCTCTACACTTTGCTCATCCATCCCTTCGTCGTCCGTCCA
CCGATCCATGATCAGATCGCTACCGTGACATTATATGGTTTCTTTTCCCTCTCTTTTTTC
TTTTCTTTGTTTGAAGTTGAACACAATTCGGCATGTCTCGTATCGAGGATGCACTTGTTT
ACCTTTCCCGGCATCATGCCTGGCCTCTCGTTTGCTCCTGTCACCCTTTATCATGGCAGC
TTGTGTTACCTGAATTATTGCGACGCTTTTTCTTTCCGTTTCGCCCCCCCTCCTACCACC
GCTTTTACGCTGCTCAGAGCTTGTTGGACTGGAGCAATTTGCTCCTCCGTCAATTCCTAT
CAGCACAGCTTTTGCACTGCTCAGGATCGCCCCCACCACTTTCTGAATCCTGGCCCTATT
CATGAGAGTTCAGCACTGCACAAAAAGCTACGTACATTGGCTCTCCATCTGCCAAAGATC
AAGATCGCACGGGCGCCCAGATGATCATCAATCGATCAGCCTACTGAATCTGTCCCTGTC
TCTGGTTTCCAATTGCCGCGTTAAATCTTTTCCCAAGGTCGGTTGTGTTGTGTGGTTTTT
TCTCGGTGCGCAACACTGTTCTGATATCGGTTTCAGCAACGTGCCGCCTCGTGATCGACG
ATAACCCGGACGGACGGAAACGCCTGATATGATTTGCCCGCGCCTCCTTTCCCTTTTCCA
TGTGCAAACTAGCTAGAAATAGTTGGATCCATGTGCATTTCCTGTGGATTTCCTGGCCGC
GACAGCATGTTTTGTGTGGGTCTGGGACTGTCCATTACTTAAACATGGCAGGTATATCCG
GCCTGCCTCATTCATCACGCGCCAAGAACTGGCCTACCACCACCCGAGGCCTCTGTTTCA
AACGAAAAGGCCATGCTAACTCCCAAACCTAGAAGGTTTATAGTACCACTACATAAGTGC
TTACACTGCAAAATACAGTGTGGACGAATCGAGGCATTTCCGTACGATAAGAGGAGGACC
ATGCGTGCATGTTCACAGTCCACGGATGCATGTATACTGTATTTTCTAAGCCGAGGTTGT
CCAGGCTTGCAACCAGCTGAGGACCTGAAAGTTTTTCATTTTTAGTTTCATTAAAAGGTT
TAATGGTTGTGCCAACTTTCGCAAAATAAAATAAAATAAAATGGTAGTGCCAACTGAACA

TGATCTATCCTAAATATAGGGCAACACCAACCCATATAGTGTGATCTAGTATTCTAGTGG
TTCGGTTGATGACCACGAGAAAAATGAGATCTGTATAAGCTCTTGCATCCTGGTCAATTT
GGAATTTGGACTTCATGGGAGCCCTTATAGCAAGTTCTTTTAATTAAGATAAACCATTTC
TTAAAAGTGCAGGGTGAGATCCTACTAATCATGTTAAAGTTTCATTGTCTACGACTCTAC
GTTGTGGGAGAGCATTTCTAGGTCATTTAAGTATCGTGCGGTCGTCCTCGGAAAAGGAAA
ACAAGACGGGTATTATATCATGATAATATCTTACTTTTAAACGTTAGTGTCAGCGTACTA
AATAACATATGAAAATTTGCTTATTTTTTCAGGTGCTTTGGGACCACTTGACTCTGTTTT
TATTTGACTTCTACTTGTGCAATTATCATTCGAAGATAGCTTCACTGTATTTGTCTACAT
GTGTTAGCCATAAGACTGAGATAATCACATTCTATGAGCACAACTAGTTGGATCAAATTT
GTCACATTTTTTATTTAAGAAAAAATACAAGAAAGATTCAGGTGTCCATTTTTTAAATGT
AGAATGTATATCCTCTAACCGGAACTTGAACCATCAGTATTATTGTTATTTCGGATTCTA
GTCTCTTGCATTATTTCTTGCATTTGTAATATTTTCGTATCTTGGTCCACTTTAAAGTAC
AGTTGTGTTTATTCTCATTTTAATCTTGCTGAACAAAGTAGACGATTCTGTTCTATCTTT
ACACCTTTACAACTTGATAGGCTGCTAATGTGTTCCTCTCATCATTTTATTGCCTTGATT
CATGAATATCTTACCCAGTGGGGACAGTTTCCAATCATTGATTGATCCGGCAGTTAGTGC
AGATTTTATAAATTAAGTGCGCACATTGTCCCAGCAAACTTGCATGCTTCTATACATATG
GTTTCAGACACACTATACTGTGGTCCATGTTCAACAGGCATTGCAATATTTTTGTCTGCC
GTTGACAAATCTCAGGTACAATTTTTTTTTTGATAAAGAGGTACAATATTTTTATCTAAG
ATAAAGGCCCCAAGCACTGAAAGGAGTGATTGACAGATGAATTAAGTGGTCCCTTTGTGT
ACTGTACATTATATATTCCAACAGTATGCGTGAAGAATATTAAGTATTCTGACAGTATAT
AGTTTGGTGATTTCAGCGAGAGCATATATTTATATTTCGAAACACGTGCCTAACTACCTA
GGAGCATCTTATCTTGTTATAAAATCATGCTAGGATAAGGAATATCTTATTTAGTTTTCA
ACAAAGACTAGCAATGTTGTCTCTCAGGACTTAAGAGCCAAGGAAAAAGCTACTTCCTAT
TTAAAATCTTAGTTGGTGTGTTCTTTAATAAAGAAGGGAGCATGAGACCCCAGCATTACA
GAAAGCGTCAAGGATAACAGTGTGTTGTTAAGTTTTGGATAAATGTTCTAAGTTATGGAA
CATTTTCCATTCTCATTTTGTTAACTCCCTATCAGATACTTGGGAGTTGATATTATCCCT
TTATTTTTGACAGGAAAACTCTTGAGGTATTGCTTATTCACTTCTTGTAGCGGTAGCTAT
GCTTTTCGAAGTTGCTGGCAACACACAAAATTGACATGTTTGTATATCAACATTCAGCAT
GTTATACAGTTTACTGTTCAGCACTGCATTAGCTTAATTTCCTGGTGTTGTGGCCTGATA
TTAATCCTTCATGCATAAGCAATGATTGGTGTCTTGATCATGAGTTTTATTTACTTAGGT
TTCACCTGCTTACGGAGTTTGATGAGGCCAAACGCAGCTGTAGAAAGCGTCTTGATGGGC
ACAACCGTCGCCGCAGGAAACCGCAGCCGGATCCGATGAATTCTGCAAGTTTTATGACGA
GTCAACAAGGTTACATTTTTTTACTACTGCTCTATATTTTAACAGTTCATACATGATTGG
AAATATGGAATTGTCTGTCAGACATATTGTAAATCATTACTCACCTAGCATGTTGACCTA
TTGCATTGACCTTCAGTACCTTCCAGCTTGTAAACCACCCCGTATAATTCTTCACCAAGT
TGCACATTTGAACCTTATTCAGCAGTTTTCCTTCTATAAATTAGTCGCAAGTGGGATTAG
TATTCAGAACGAACAAGGATAACACCTTAGTTACCCATATAACCTACCTAATTCATAAAG
GCTGGATGAAGTGTAAATAAATATAGAGTCAAAGTTTTAATTCATTAGGAAAAAAGCCAC
TCTTTACCTTCTTCACAGTTACTTCCTCCGTTCACAAATACAAGATGTTTTGTATATTTC
AATATGGATTACATACATACGGACGGAAATTAGTGAACAAACAAAGTAAAACGTGTCTAT
ATATACATCGATTCACAAAAAGGTTAGGACATCTTATATTTGTGAACGGAGGGAGTAGAG
TCCTAGTGCCTGAAACACTCATATATTACTCCCTCCGTAAATAAATATAAGAGTGTTTAG
ATCATTAAAGTAATGAAATGAAACACTCTTATATTTTTTTACAGAGGGAGTAAATGTTA
ATTTTTGATAATGCCTGAAGAAAGATTTTACTCATTCTATCTCCATACGTATCTGTAGCA
TTTCCATTATCTTCTTACCACAACCAATTGACATTTTTGCGAAACCCAGGAACAAGGTTT
CCATCATTTCCAACTCCAAGACCGGAGCAAAACTGGCCAGGGATCATTAAAACTGAGGAG
AACCCCTATTACACACATCAGCTCCCTCTAGGCATCAGTAACAGGCATCATTTTGGTGGC
TCTACATCTACTTTCGCGAAAGAAGGACGGAGATTTCCTTTCCTACAGGAGGGCGAAATA
AACTTTGCCACCGGTGTGGCACTTGAGCCTGCAGTGTGCCAGCCGCTGCTCAAGACGGTA
GCTCCTCCCGAGAGCAGCAGCAGCAGCAGCAAGATGTTCTCCGATGGTCTGACTCCGGTG
CTCGACTCGGACTGTGCTCTCTCTCTTCTGTCAGCTCCGGCAAATTCCTCCGGTATCGAT
GTTGGCCAGATGGTCCAACAGACTGAACACATCCCCATTGCCCAACCTCTGTTCTCCAAC

CTGCAGCAATTCAGCAGCTCGTCCTGGTTCTCGCGCACCCAGGCTTCCACCGGCACCGTC
TCAGCGACCGGATTTTCCTGCCCCGTGGTGGAAAATGAGCAGCTGAACAATGTCCTAACC
TCGGACAACAATGACTTGAACTACAATGGGATTTTTCATGTCGGAGGCGAAGGCTCCTCA
GACGGCGCTCCGCCATCTCTGCCCTTCCCGTGGCAGTAGTTTTCAGTGGCTGTTCATGTG
TTGCTTACAGTAGAGCGCATCTTTTCTCCCTAGTGATCAACATTTGCTTCTGTTCAATCT
GAATCATTCCTTTCACTTGTATTCCTTTTACTTGCCAGCATTCGTGTGAACAGATTCAGC
ATAAATCAATTCCTCGAAATCTTATGGTGTCAAGTGACAACTGTGGTACTTTTTTTTTC
TTAGAAAAGGAGGATAAGGAGCATGACCCCCGGCCTCTGCTTCTGAAAGATGCATGCAAT
CACTTTATTAATTATTTATAAAGATCTTACAAAATAATATATCAGTGTGTCTCAAGCCAC
CATCTTAACAACATACGTCGCTACTCTTATTCATTGATGAAAGGGTGCTGAATGTCAGAG
CCGAATACCAAACATATATCGCAACAAAGCCTAACATCTAAATACGGATGCCCTCACCAA
GTCACATAACAGGTCTGGGGTCCAAGCAGATCTGACATACTCTCATAGGTCGTCGCTGCC
GTCTTCCATCAATTCATCTTAAGAAAATGACTTCTATACCACTGAGGCAAGCATCAATTG
CTCATTTGCCATCGAACAAAGCTCCACTGCTCATATACCAGACACAAACGAATTCTAGTG
CTTATTTGCCATCGCGGTTAGTCCATCATCAGAATCTCTACGTTTTCTGTTAGTCAATCT
GAGAATTCCAGTTTTACCCTCGTCTTATCTCTTCTCTTAGAATAGAAAAAACCCAGAAGC
AACACCACACCACCCGACCAGTTCACGCACGCACACAGCAGCGGCCATGGCGGAACCGCC
GCCGCCGCCGCTCCCCCAGCGCATGCCCCTCCTCCCTCTCCCGCTCC
>HORVU7Hr1G051400.2 genomic sequence
AAATTAATGGACATCCAACGCATGCGGAGCCGAGAATGCATATCCAACGCATAATTTAAC
CATTTCAATTTGATAGCTGACCACATTTGCCATGTGGTTGCTAAAGGGTGAAGTTATTTT
TTAACCTTCTAGTCATGATATATAATTTTGAAGGATTTTTCGTTGATAAGCGATCATACT
ACGCACACTAATAAGAAGTTGCTTTTCTTTAGCACTAAAGTTGCCTAAATATCATCCCAT
AGTTGTTTAAAAAAATATTAAAATTTATCCATATGTGATCTAGTTTTGAAGAGCTCGTCG
TAAGAAACACAACCGTAAAAACGGATAGTTGTTGTAAGGCTCGGTTCAAAAGTCATACTA
TTTTTTAAATAAAATAAAGTAAAGTTGAATGACTTCCTGCATAGGGTGTTCTTGTTCCAA
CTAAATAGTACAGCTACATTTTTTGGTATTTATAAAAATATATATTTGCGTACTCGTACT
ACACGTGTCAGGCCAAGTACAATGATGAAGCAGGACGTAGTACCGTCCGGGGAGGCCCGT
TCGCGCAGAGGATACGAGGCAGGCCAGGCAGCCAGCCACTGCGACGTACCGGCACCAGCA
GGCAAAGCGACCCGAGAGGAGCGGGCAGGGGGCAAGGGGGGACGCACCCGCACCGCGGCA
CGGACGCCGCGGTACGAGTACGAGTGGTGGTGCGGTTTGACAGGCCCCGCCCCCGAGAGC
AGAGGGCCAGCAGCATCAGTCGTACTACCAGCGCCACTGCACCCGTGCCTATCGCCAACT
CCCGCGATGCGGCCTCGCTCGCCCCCAGCCAGCCAGACCCGCCCGTACGGCCCGCGGCGT
GGGACGGCGGGGCGCCACCACCCACCCCCGCGCGTTCTTTCCCTCTCGCCCCTGCCCCGC
CTCCCCAATTACGCGCCCATTGCCACGCCACGCCACGCCACGCCACGCGAACCCTCCCTC
GCCCGCTACACGCTACGCTAGTTGAGCAGCTAATCCCAACCCGAACCCCGTGCGCAGCTG
CGCTCGCCCTCCCTCCCACCCGTCCCGTCTCTTCGCCTCGCCCCGATTCCTCCCCCCTCC
CACCCACACTCGGTCTTCGTCTCCCCCGACCCCCTCCCTCACCCCTCACGCCTCTCGCCA
GCGCCGCGCTCTCGCGTCCCGCCCGCCGTTTCCGCCATTTCTCCGGGCTGCTGCCCACGC
CCCGCGTCCGTTTTCTCCCCGCGGGGGCGAGATGAGGTGAGGCGGTTTTCTTCCGGGACT
TCGCCGAGCTTCCGTGCGCGCAAAGGTAACTGACTAACAAACCAACTGCTCCGATCGAAT
CCCCCGCTCCCGCTCGTCGGCGATCGTCGGTTTTGATCCGTGCCGCGGTGCTGGGCTGGC
GCAGATCCGAAGGGACAGCCAGCCGGGTGTGCTAGGAAGCGCAGGACCGCGGGTGATGCA
GAGGGAGGTGGGGCCGCAGGTGGCCTCCCCGCTGTACCTGCACCAGATCCAGCCGCTGCC
TCCCCATGCGGCGGCGGCGGCCAGGAAGCGCGGGACCCCGTGGCCCGCCGCGGACCCGCC
GGAGAACGCCGCGATGGGGGCCGGCGCCGCCGCCGGAGGGAACTGGAACCCCAGCATGTG
GGACTGGGACAGCCGCGCCTTCACCGCCAGGCCGTCCTCCGACGCGCTCCGCCTCGGCGG
CGGCCTGAACCACCACCAACACCACCACCAGCAGCCGCCGCCGCCGCCGCCGCCGGCGAC
GGCTGCCGAGGCGCAGCGGCAGGGGCGCGGAGGTGCCGGTGACCTGAGCCTTCAGCTGAA
CCTGCGGGAGGAGGCATCGATGGCGATGGATGTGAGCCCGACAACTACCATGTCTTCTTC
GCCTTCTCCGCCTGCACGGACGTCGCAGGAGCAGGCTGCTAGGCCTAGCAAGAGGGTTCG
GTCTGGATCGCCCGGAACTGCTTCTGGAGGAGGTGGCGGCGGCGGTGCCGGAGGGAGCGC

AAGCGGAGGCGGGAGCTATCCCATGTGCCAGGTGGATGACTGCCGCGCGGATCTCACCAG
CGCCAAGGATTATCACCGGAGGCACAAGGTGTGTGAGATCCACAGCAAGACAACCAAGGC
GGTAGTTGCCAACCAGATGCAGCGCTTCTGCCAGCAGTGTAGTAGGTAATGATCGTGCCT
GCAAACACCTCCTCAAAAATTTCAGTGGCATGATTCTTTGTTATGATGCCACCTTTAGGG
ATTTATGTCTCCTCATGATTTGAATGGTTTCTTAGTTTGTGTATTGGGCCATAGAAATGG
TTTCTCTATCCAATGATGGCAGTGTTTATCTAGTTTCATACTGTATCATTTACCACGTCC
GTTCCAAAATACAAGTCATTTTAGAGATTTCACTAAAGACTCCATACGGAGCAAAATGAG
TGAATCTACACTCTAAAGTATGTCTATATGCATCCGTATGTAGTCTGTAGTGAAATCCCT
AAAAAAAAAACACTTAATAGAGTATTTAGGAACGGAGGGAGTAGTTACTAAAAGTCCATT
CTTATGGTCAATCATTTAGATTTCATCCACTCTCGGAGTTCGATGAGGGTAAGAGGAGTT
GCAGGCGAAGGCTTGCCGGACACAACCGACGACGGAGGAAAACCCAGCCAACAGATGTTG
CTTCACAGTTGCTGCTTCCTGAAAACCAAGAAAACACAGCAAATAGGACACAGGATATTG
TCAATCTGATCACTGTTATTGCACGCTTGCAAGGTACTTGACTCCTACTGAACTTGGTAC
ACTCTTTCACTACTTGTTGATTTTAACCTGAGTACTATTTTTCCTGTCTCTGCATCGACA
GGTGGTAATGTTGGTAAACTACCCAGCATCCCTCCGATACCGGATAAAGATAATCTGGTC
CAAATCATAAGCAAAATAAACTCTATCAATAATGCAAACTCCCTGGGGAAGTCTCCTCCA
TCAGAAGCCATTGATTTGAATGCCTCCCATGGGCAACAACAAGATTCCCCTGTTCAAAAT
GCAACAAAGGTGGTCGACAAGCAGACTGTGCCATCAACCATGGATTTGTTAACAGTTCTA
TCCGGTGCTCTTGGGACATCAACCCCTGAAACCAATACATCTCAGTCCCAAGGGAGCAGT
GATAGCAGTGGTAATAACAAGAGCAAGAGTCATTCAACAGAGCCAGCTTGTGTTGTAAAT
TCCCATGAGAAATCAATTCGACCTTTTCCGGCAGCTGGTGTGATAAGGAGCAATAGCACC
CATGGAAGTCCACCTGAAATATATAAGCAGCCAGACCGAGATACCCATCCATACTTGTCA
CTGCAGTTGTTTGGTAATGCCGAGGTGGACATTCCTGTTAAAATGGACACTGCAAATAAG
TACTTGTCTTCGGAGAGCAGTAATCCTATGGACGAGAGATCTCCTTCATCCTCTCCACCT
GTAACGCGCACATTTTTCCCCACCCGCTCAGTAAATGAAGGCATCAGACATCCTCGTATT
GCTGACTATGGAGAAGACGCTGCAACAGCTGAGATCAGTACCACTCGGGCATGGTGTGCA
CCACAGCTTGAGCTTTTCAAGGATTCAGAACGGCCAACGGAAAATGGGTCACCACCAAAC
CCCACATATCAGTCATGTTATGCCTCAACATCTGGTTCAGACCATTCTCCGTCAACATCA
AATTCAGATGGACAGGTAATTTATATCAAAGCTTGCCATTTCAGTTGATTATGTCACAAG
CCAAGGTGTCATTTATTATTTTAGCCACGCTTTCAATCTGATCCGCACATGATTTTCTAT
TTCATAGGACCGTACTGGAAAGATTATATTCAAGCTCTTTGGCAAGGAACCTGGCTCAAT
CCCTGGGAACCTTCGTGACGAGGTATGACCACGAAACTGTGCCAACATGGTAACTGGAAG
GCACTCTTCCAGCGTATTTCATTCACCATATAATATAAATGTTTCTCCTTCCTTGCTCAG
GTTGTAAATTGGCTGAAACACAGCCCCACTGAAATGGAGGGCTACATTCGCCCTGGTTGC
CTTGTACTATCCATGTATCTGTCAATGCCAACTATTGCATGGGATGAAGTAAGTCTGCTG
CTACACTAACAAAACCGATTGCATGCAATGAAATTCTTGCAAATTTATCTAACGTTGTAT
GATTTTTAATGCAGCTTGAAGAGAATTTTCTCCAGCGAGTAAACTCGTTAGTTCAGGCTT
CTGATTTGGATTTCTGGAGAAAAGGGAGGTTTTAGTTCGAAGTGACAACCAGTTGGTGT
CGTACAAAGATGGTATACATAACTACCATCTCTGTGTTCTTCTCTATTCCTGCTCACTCT
TTGGTGGGTTGACTATGAATTCGTTTTGGTTGATATCAAATTTGTATTTCAGGAATGACC
CGCCTGTCAAAATCATGGAGAACATGGAATACTCCCGAGTTGACCCTTGTGACACCAATT
GCTGTTGTTGGTGGGAGAAAGACCTCCCTCGTTCTTAAGGGCCGTAATCTAACGATCCCG
GGCACCCAGTAAGTTCCCTGCTGTGAAATAGTTTCTGACCGGGAGGATTATTGTGAATGT
TTTGTGCTTGACCACTGACTCTGAATCTATGCAGGATCCACTGTACCAGTGGAGGGAAGT
ATATATCAAAAGAGGTATTATGTTCAGCGTATCCGGGTACTATATATGATGATTCAGGAG
TTGAGACCTTTGACTTGCCGGGAGAACCAAATCTTACTCTTGGGCGTTGCTTTATTGAGG
TTTGTACTTTGTACCTATGCGGTTGCTCTAGATATGTAAAGATTACTATAGTTAGCACTA
GGAAGGTGATTACAACTATGAATGTTCATACGTGCAGGTTGAGAACAGGTTCAGGGGAAA
CAGCTTCCCTGTTATTTTTGCGAGTAAAAGCATCTGTCACGAGTTAAGAAACCTTGAAGC
TGAACTTGAAGATTCGCGGTTTCCTGATGTCTCTTCAGATGATCAGGTCCATGATGCTAG
GCGGCTAAAGCCAAGGGATCAAGTTCTGCATTTTCTTAATGAACTTGGCTGGCTCTTTCA
GAAGGCCGCTGCATGCACACCTTCCATCGAATCAGATGTTTCTGATTCAGAGTTGATTCA

GTTCTCTACTGCACGGTTCAGACACCTTCTGTTGTTTTCAAATGAGCGGGACTGGTGCTC
TCTCACTAAAACACTACTCGAAGTTCTTTCCAAGAGAAGTTTGGTCAGTGAGGAGCTATC
ACAAGAGACTCTGGAGATGCTCTCTGAGATTCATCTTCTGAACAGGGCAGTGAAAAGGAA
GAGTAGCCACATGGTGCATTTGCTTGTGCAGTTTGTCGTAATTTGCCCTGACAACTCCAA
ACTGTACCCTTTCCTTCCAAATTATCCCGGCCCAGGTGGTTTGACTCCATTACATCTAGC
TGCATCCATTGATGATGCAGAGGATATAGTTGATGCCTTGACAGATGATCCTCAACAGGT
AAGCCCCGTATAGTTTAACCTTTCTTTATCATAGTTATTTCACATTGATAACTCTTCTGA
TTTTTCTGCTTAAATTACTGCAGATTGGTTTGAGCTGCTGGCATTCGGTGCTAGATGATG
AAGGGATATCTCCTGAAGTGTATGCGAAGTTCAGGAACAACGGCTCATACAATGAACTTG
TTGCGCGAAAGCTTGTGGACAGGAAGAATAGCCAGGTTACCATTGTGCTCAACAAAGGGG
AAATTCATATGGATCAACCAGAGAATGCTGGTGCGAATAATTCATCTGGGATCCAAGCAT
TGGAAATAAGATCCTGCAGCCAGTGCGCCATTTTGGAGTCTGGCTTGCTAAGACGCCCTA
TGCGTTCTCGAGGATTGCTTGCTCGCCCCTATATCCATTCAATGCTTGCCATAGCAGCAG
TGTGTGTCTGTGTCTGTGTATTCATGCGAGCTTTGCTGCGGTTTAATTCTGGTAGGACCT
TCAAGTGGGAGAGGTTGGATTTTGGTCCCACCTAGACATTGGAGAATGCCGTATTACTTA
GCTGTGTGATGTTATTAAGGAACTACCTTACAAGAACCAGAGGGTGTGGAACCAGATTGC
TTTTAACTGCTCCACTTTCCATGGAATTCTGTCCATGGTTATGGATGGTGAAATGTAATA
GTTGCATGGATAAAACCTTGCCGGAAGAAAGTGTTGGTATGTGGGGATAGTCATCAGCTT
CTAAGGGGAGAGTTGAGAATCAAGAAAAGGGGGGCCTGCCAAAAGAAAAACTTGGGCAGA
GTTGGCACATAGTTTTGTGATGAGGGAGGAAGCTACCAGAGCTTTGTCAATGTCAGGAGC
AATAAGACAGAGGCAACAATAGTTCTGTGGTTAATCAGTAAGTAACCCTTTGATTTCTTT
TTGCTGGTGTAAGTGTGAATCTGCTGTTTCATGTATCATAAGATGGTATTTATTTATCTT
GGTCTTTTGTTGTTACTAAATCTTTTTTTGTTTACCAAATACAGTGCTTACTGTTTTGTC
TTGTGTTTAGACGGTAGCAGGGGTATAAATGGTCGATAGTGTCAGTAGCTAAATAGGAAT
GAGGCAGACTTACATGTGTATCCGGATCTATTTGGTTTATTGGATTACCATCAACAACTC
TCCAGTTCCCGTTTTGATAGAAAGCCTGGGTGTCTATCTTTCTTTCTTTCTCTGCGTGCA
TCTTCTTGTTAAATATGCAATGCCAGTTATGCTGATTCGCTTCCCTGTTTTGATTGAAAG
CTACGTGAAAACAGTGTTGCAACTATGCCATGCCTGTGGTGGGGCATCTGCATCTGGGCG
TAAAAAGGCTGTAGCCTAGGCGTTGTGCTGTGTTATCCTTGCGGGCGCGGGGGGCTCGTG
ATGCGAATGAGATGCCGGCCGGCGCTTTTCCGGATCGCGCCGTGTGCGGATCGCCGGGCG
GACACGCCGGCCAGATTCCGGCGAAGCTGGCACGCCCCGGGCGGGCAGAAGCCGGACCCA
GACGCGTGATCTGCACAGCACGCGCGGTAGGAAACGATGCCTTTCCCTGGCCCGGGGGCG
CATGTGTGCGTTCCACGGCCGGAGTCGCGCGGCCTATGGCTTTCGTCCCTCCTCCCCTTT
GTCCATCCCACATGCCTCTCGGTCTCGGATTGTACGCTTGCTTCCCAGCCACTCACACAT
GCAGCCACGATCGCATCTCTCGCCTACAGCGATGGCACGCAGTGCCGCCGTCCGTCCGTC
CGTCCGTCCTCATCGATTCGCTCCGCCAGCCTTGATCGGCCGCTTTACGCACGACGTCTA
GTGGCGGCCTAGCTGCTACTGGCACAGCATCAGCATGTGAGCGCCCCTCTTTCCAGCCTC
TCTCGCCCACGCTCACCGTTCG
>HORVU5Hr1G076380 genomic sequence
NNNNNNNNNNNNNNNNNNNNNNNNNNNNNNNNNNNNNNNNNNNNNNNNNNNNNNNNCCCTT
TATATAGTGGAGGAAAATATCTAAATGTTATCCCCACTCACAGCCCGAGCAAACCGATAC
TAGCGCTGCTAGGAGCGGTACTATCGCTTCTAGGAGCGGTACTACCGCACGGTAGATCCA
CCAACCAGAGAAGTAAAAAAATTACTTCCGTACCTACCACCGCTGAGGGTGCGTTTGTGC
AAAAGTCCGAGAGAGCGGTAGTAAAAAACTACTACCGCCCACAGAGCGGTACTACTATTG
CACCTGCGGTACAACCGCTCGCACTTTGCACAAACAGGGGAAACAAGGTGGAGCTCCATT
GAAGCGAGGGAAAGATGGTGCAAAAGAACAGATGTGTACGTGTTGATTCCACCCTAATCT
TTCCGAAGTGGCCCCCTCTTAATAGTACGGCTTTTCTATGATTTAAATTCACCAAAAAGA
AACGTAGAAAACAGCCGTCTTTGATAGACTCCGAGGGGCAGTGAACCGTCTTGTGCCTAG
ACATGAATTATCTGAAATGCTCAATACACACGATTAGTCGCACACGCATTGTCATCAATC
GCTAAAATCATTTAGAGAGAAATATGCCCTAACATTAGTTATTTAATTATTTTCCATGTG
ATTTATGTCACTATTCATCGGATTTTCATTGTTTCATTTTGGGTTCTCTTTGCTTTTCAT
TTTTGTTACTTTGCTTATYTTTMTRTGTGTTTTCTTTGGTGTTCTTCAATTTTCATTGGA

ATTTGTTCCTTTTATGTTTTNNNNNNNNNNNNNNNNNNNNNNNNNNNNNNNNNNNNNNNN
NNNNNNNNNNNNNNNNNNNNNNNNNNNNNNNNNNNNNNNNNNNNNNNNNNNNNNNNNNNN
TCGTGCGCGGTGGACGGCTGCAAGGCCGACCTGAGCAAGTGCCGCGACTACCACCGGCGG
CACAAGGTGTGCGAGGCGCACTCCAAGACGCCCCTCGTCGTCGTCGCCGGCCGCGAGATG
CGCTTCTGCCAGCAGTGCAGCAGGTAACCAACAATCACCCGCCAAAACTGCAAAAAATTC
CCTCATTCCCATCCATCTCATATCAGCAACCAATCCTCCTTGACTGTGACACCGGTGAAA
GAAAAGAACACCATTGCTTGCTTGAACTGTCGCCATGAAAATACTTGCTGCATGAGAAGA
ACTGTCTGATTGGCCTCACCAGTCATTTCTTTCACACACGCATTTTTTTCTCCTTTTTTA
CCGCTGATCGTCGTTTCCTTTTTTAAAAGGCTGAAAAGGCCCTGGCTGAAACTAACCAGT
TGCTCCCAGTCCAAGCAAGGAGGGCTACTGAATGAGCTGAGCATATCTCACTGTTCTTAC
AAGGATGCACCAGTAACTTTTCCTTTATCCGGCACTAACTTTTCAGCCTTTCCATATGGA
TCAAGTACCCCCCATGTTTTTTACTTTGGGCAACCAGTTGCATACCTGATTCATAACCCT
CCCATTTTCCCGCTTTTGCACCAGTAACTTTCTAGCCCACGGGTGTGAATTATTACCAGC
GAAAAGTGGCTGATGCACTCCATGTGCCAAAGATCAGCTGCACCAGTCCTTTTTATGATT
TGCTAGCTGCCACATTTTCAAACCTGCCCTTGGATTCTCCCCTTTCCTGAATTATTATTT
TTGGCTCTGGTTTGAAGCTCAAGAGTGGAGCATAATTTCTGACATTGACAAATATGTTAC
TGCGACTTTCATTCTGGCAAACAAAAAATCACCACAGATTTTCCATGACATTGACAAAGA
TGTCACTACTGCTTTTATTCTGGCTAAAGAAAACAGGCGCAATGCTCTGCTGGGGAGTCC
CCAGAAAAATCATCACATATTTTAAACCAACTAGTAGGGAGACAAGAGGGTTAATCTGTG
TTAACCAAGCCACTGTTAACCGTAGGTTACTCTAGGATGAACCCATTTTACCCTTGAGGT
GGTTTATGTCTGACAAGACTGCCCCCCGTTGCTGAATTAGTAGTAAAACCACCAGATCCA
TGCGAATTCTTTGGGTTTACTCGTGGGGTTATTGTGTGCAGCAGTTTTTGTTGTGTGGGG
ACTGTTCACTCCTTAAAGATGTTGCTCAACACATGACCTGTTTTATTAAATTCTTGGCCT
ACCCCATCTGCCATGACCGCATCATGCTTACTAGCCTTGTTTCTTTCCACATGAGATGCC
GGACCACCGGAGCGAATTGTCGAAACAAATACGATCCCACATCTTGCATTTTGGTGGAGC
CGGTTGTCTCCGAATTGAATTTTTTTGGGTTTTCTGGTTAGCTGAATCATTGGAGCCCAA
CAAAAGCCAACCGGCTCCTGAAAATTCAAGGAATACAAAAACCACCCGTCAATCATTTGG
TCGTCTTCTCCCGATAAAAGATTGAAAATTTTCAGTTGGGACGCGTTCGCAATGATTGGG
CGCCCATCAGGCACACGATCAGGCAATAGTGCGCCGGGTGGAGCGAAATTTGGATCACCA
CGCTGTGCTCCATCAGTGTTCCTACATACTACTTACATACAGGCTGGTGCCGCACTGACA
TACGCCTGTTGGAACCTATGCCGTGGTCCAAATGCCCAACAGGAGTAGCACTAATTTGCA
GCGCTTTGCAGGCCCAGTAGAAAACTTTTTGTAAAGCCAAGCCCACCCCCCATGCACTCA
GCTTTGTTAACTGACGCATTGAAACCCGAGTGGTCCAGCTGCTCGTGTGTGTGTGTACTC
TTTTCTTGGTCGAATCTGTGTTCTGTTTTAATGTTTGGCAACAAACAAGTATCCGGACCA
TTCTGTTTGAGTGCCGTGTTGGTCCATGTAGTTTTTGATTATGAGTTACTCATATATGAT
ATGCGTACGATCAATACAGCAGCTTATATTCACCGTTATTGGTCCATGATGGCACCATGG
CGGGTTATCATGCCATTGTAGCACTTTCCTGTATTATGAGAGTAATGCTTTTATTTTCTT
ATAACAAGGTTATGCGACGTTGTATTCGCATGGTCTTCAAGTGTGTTCTGATTTCGTCTC
TTCACGTTATTACAATCATGTGTGTTTGGTATGTCATCAACAGTTTCAGTAGATACTGAT
TAGTACACACTCATATATGGTTAGCTCTTCCTTCGTACTCCTTTGCACGTAGATGTCCAT
CTTTCTTATCGTGAATCTCTTATTCCATAGGTTCCACATGCTTGCCGAGTTTGATGAGGC
TAAGCGTAGCTGCAGAAAGCGCCTTGATGGGCACAATCGACGCCGCAGGAAGCCACAGGT
AGATAGCATGAATTCTGGGAGCTTCATGACAACCCAACAAGGTACTCTTCTTGTCTAACT
ACCTCTTTTTTATTACCATGTTGGTTGCTACTATTATACATAGCTAAAATAAACAAAATC
ATATAGTAACCGCAGAACCAGAAACTTCGGTTATAAAATACATCAATTCAAATGCAAACC
CTCACCAAAATGCTCATGTTACCTGTAGTTGTTCGTACATCTTTCGTGCAAAACTAAGGT
TTCATAATTTTGTTGGGGCATGATTTCTCTTGTATAACCTATAACAAGCTTGACCTGCAT
TTTGCATGGAGCTACGGCCGTTTTCCTATAAGGACGAAAGTTGGCATAAACATGCTGCTC
TGTGTTTAATTATGTTTAATCATGGGTTTATGGTATAAATGAAATGCGGGATGTACTGTT
CTTCAGTAGCCACAAGAGCACATTGTAACCTGAGCATTCAAATGAACAGGCTGAGTGCAC
AATGCTGCATTTTCAGTCTTTTACGTTGCTGCTACGTATGTTTGAATACTGTACTATTTG
TCTGATGCTCGGTCGTTTCATGGCGGCCGTTCAGGTTTGTATTCAAATGCAGGGACAAGG

TTCGCGTCCTTCTCGGCTCCAAGGCCGGAGCCAAGCTGGTCCGGGATCATCAAATCCGAG
GACAGCAATCCATTCTACAGCACGCACCAGGTTAACTTCGGCGGCGCCACGTCGTCATAC
TCCAAAGAAGGCCGGCGCTTCCCCTTCCTCCACGAAGGAGACCAGATGAGCTTCAGCACC
GGCGCGCCAGCACTCGAGATCCCCGTGTGCCAGCCTCTCCTCAAGGCCGTCGCCCCGCTG
CCGCCGCCGCCCGAGAGCAGCAGCAGCAACAAGATGTTCTCCGATGGACAGTTGACGCAC
GTGCTCGACTCCGACTGTGCTCTCTCTCTTCTGTCATCCCCGGCCAACTCCTCCAGCGTC
GACGTCAGCCGGATGGTCCGTCCAAGCGAGCACGTCCCCTCGGTCCCCAGCCTGCAGTTC
GGCACCTCCTCCTGGTTCGCCTGCTCCCAGGCCTCAAGCGGCGGCGGCGCCACCGGATTC
GCCGCCACCTTCCCCGGCGGCATGGACGGCGAGCAGCTCAACGCCGGCGGCGCGCTGGTC
CCCGGCTCCAACGACAACGAGATGAACTGCCACGGGATCTTCCACGTCGGCGCCGATGGC
TCCTCCGAGGGCACGTCGCCGTCCCTCCCGTTCTCATGGCAGTAGGAGTCTCGAGTGAAG
ATCAGAGGCTGTTCTTGGGTTCTTTGGTGATCAGTAGGCAGTAGCTAGCTGTGCCCCTT
TTAGTGATCAGGCCTACGTCCCGTTCTTTCTGTGTTTATTCTCTCTCCTTTTCACGTTTC
TATGTCTTGGACTTGATCTCTCGCTATCAGAATGAATAACTTTGCATGATTCAGAGGGAA
ATCGTATACGTACTTCTGAATTTCACTGATGCATTGGAGACTAGATTTAGACAGCCTTGT
CTTTCCTTTGTGCAAGATGCAAGAACGATTCGCCTCAGGACTATTATTTTGTTCTATATA
TAGTTTCAAAGCTTTTAAATCTGGATTCAAAAAGAAAAATGTACTTGAAGTTCTAAATAG
TTCACCTCCCATTAAGAAAGATTCATATAATGTAAATTTTGTAATGTAATTTGTGAACAA
AATGCCCATATCATCCATGATCAACACATTTAAACAAATGTTCACGCATTTAAAAATATG
TTTTTGACATTGTAAAAAATGTCCACACAATTTTTAAAATCTTAGTACAATTTAACAAAA
TGTACACATGTTTACCAATATGTTCGTGAGATTTCAAGAAATATTCATGATAATGCAAAA
ACAAAATCTCACAATTCGTAAAAGATGTTCTTACCATTCGAAAAAAATTATCAAGACATA
AAGAAATTTCCGCGTGTTTGAAATATGTTTCATTACATTATAATTTTTTTATAAAGTGT
AAAACTTTGTTCACATGAATTTAGAAAAATGTTCATAACATATACAAAAATGTTGGTTCC
ATCTACAAAAATTATAGCACTTAGAATATATTTGCAGTTTTTAAAAATGTTCAA
>HORVU6Hr1G031450.6 genomic sequence
ACCGACACGCTACTCGTCTTGTCCAAACTCAAGTCATGAGTGGCGAAGAGGTAGAACACA
AAACACACATGCCACATTACAACTATATCTGAACACCTCGGAGCACGGCAAAATCTTCAA
ACCAAACTAGATCAACACACCCTCCACGCATAGTGCCTAGGAGATCGACAAATGGACAAT
ATGAGCTAGCCTATCGAAACATCACAAATGAGTGCCCAGGAGATCGACAAGTAGACAACA
TGAGTAAACCTATCTTGCAGAAACACCGCAAATGAAGCGTCAATGATGAAGTACCTAGCA
TCCCGGATGTGCATACCAACAACAATAACCAACGCATGATAACACGGTGCCCCGAGCCTC
CCCACCAAGGCAAACGAGGCACCACACTAGGTGATCCTCCATGAGACATCCTGAGCACTC
AAGCCGTGTGTCCAAGGATAGAATGTTAATGTTAATAATACTTTGTTTTATAGTAAAAGT
TATTTTTAATTAGATAAAGCATCAAATATAGGTTTGAAAAATGCACAAGCTGTCTCCAGC
TATCCACAATCCACTTATGGCAAGCCAAGCATAGAAAAATGGTGGGAAAGAACAAGAAAG
GGAAAAATGAGAGAGAGAGGGGACCGAACACTAAAAAAGACCTGCCTTTTCTATTCCAAG
TCTCTCTTCCCCCATCCCTGCTTCTCTTCTTCTCCTGCACAGTCCACACTGTTCATCTCC
TCTTCGATCCCCAAATCCCAAGCTGACCAACCAAGCCCCCGCCCCGCATGGAGTGGACGG
CCCCGAAGTCCACCCCGTCCTCCCCGCCCCACCTCCTCTGGGACTGGGGCGACGCCGCCG
CGCTGGGCTCCTCCGGCGAGGCGGCGGGGAGGCGCGGGAAGGAGAAGCGGGCCAGGGGGG
AGGGGGCCGGCGGAGGAGGCGGAGGAGGGGGCGTAAGGTGCCAGGTGGAGGGCTGTGGGA
CCGAGCTCGCCGCCGCGAAGGAGTACCACCGGAAGCACCGGGTCTGCGAGGCCCACACCA
AGTCCCCCCGCGTCGTCGTCGCCGGCCAGGAGCGCCGCTTCTGCCAGCAGTGCAGCCGGT
GGGTGTTTCTTGACGCCCCGAGGTTGTTCGCCGGAGTTTTGTTCCTGCACCCTGGTTGCT
CGCCGGAGCCGTTCTTTTCTTTGACTTCAGCGATTGCTCCCCCTCTTTCTATCACTCTGC
AAAGCTGCACGGTCGCACCTTTCCGCGATTGCTCTTTTGCTCCACTCCGTGCGTGTGCGT
GTCTTTCGTTCTTGGAATTTCGGGAAACAGGAGATTCGACAAGCCGTCTATCTGCGAATT
CTGCACTTCCTTATAGACCATGATGCCGTGCTCTAGTTCCGTTCTCCGCCAGTTGATTAA
TCCGGAAACCTTGTGTTTTCGTGTGTGTCCGGAAACTGTGGTTATCCTGTGCGAGTACGG
TCTGTTTCCCGATTTGGGAGGCGAAGATGCCTTTTCGCCACAATTATAGTACTGTCGACC
TGTGGAGAATCGTGCTGCATGGCATGGCTGAACGGCTGTGCAATACCTGTTTCCTGTTTT

GGCAGGGCGCTTGTCCCGTGCGCGTGCACTTTTAGGCTGAAATTCTCTTGTGCTGTGGCT
ACTGTGATCCAGTTACTGTGAAGATTTCTCTTCTGTGTATATATACTCTTAGATCTTAGT
TACTACATATATATGGGTAGTCTATATACTACTCGTATGTACTAGTACTGTCTGTGGTTT
TGCTGATTGCATGGCTTTTGGTGATCAAGGTTCCATGGGCTGTCGGAGTTCGACCAGAAG
AAGAGGAGCTGCCGGAGGCGACTGTCTGATCACAATGCTCGGCGCCGGAAGCCACAGCCG
GATGCATTCTCCTTTGCACCGGCAAGGCTACCGTCGTCATTGATGTTTGGTAATTTATCC
ATACTTTTAGTGGGTAATTATGCGAGATTTGTATTATTTTTCCTTACTCTCCTCATAGCT
CTGAAACTACTGCTCTTTTATGCTTAATGATTGGCTGGTTGTGAGTTGAACTTCTTTAAT
AAATTGTGTTTGGGCTAGGGATGAGTTTTGGCTTTCATTAATACCATGATTAGTCAAAAT
TTATCTTCAATAGTTGATATATGAGATGATAGCTAGTCGGTTGAATTATTTTTGCTTTCT
TGTACTTTTATCAATAAATGCTTTTGCCATTGTGCATTACAGATGATAGACGACAAATAA
GTTTTGTCTGGGATAAAGATCCACTTAGCCATGGAAGGCCTTTCCCATGTTCTCCATGGG
ACAGCCCATCTGACTTCAAGCCCCCACAAGTGAAGGAAATAAGAGAAGTGTCAATCAATG
GACAAGTTCATTTTGATAAATCTCATCTACCCAATGCTGTTCCAGCACTGAGTCATGACA
TAGCTGAGCTGCTACCAATGAAAGGTATGATTTGTCAATCATTTTGTAGCTATCACAGAA
ATGTTCTCATCGATGAAGAAGCTGGCACATTTATTACGGGAAAAATGATACATCTGTAAT
GTAGGCCTCTTTGATTCATGGGAAAGTACAAAGTAGGATTGCAGTGCCATGTCATCTTGA
ATCCCACAGGATTGAAGGATGTTTGGTTGCACATAGAAAATAATAATACAGGAATCATCC
CAACTGGTTTTAGTCAATTGATTTTTTTAGAAGGGAGTACAGAAAATCTGTAGGAAAAAT
TCATGTGGGTTACAATCCTACAAGTTGAACAACGAGCATAGGAAAAGTTCTTTAGGATGA
CAAACGTATTGAATATCTATGAAAATCCTTGAATCGAAGAGAGCAAACTCTGGTCCTCTG
CTTGGCAAAGTCGTCACTGTAAAAATGTGGTTCCGGTCCACCCACTCAAACCGAGGGAGA
TTCTTTTTTGGCCTTCACTCAATCTCTAACATGTATATCTTACATTACAAAGCTTACTTT
CAACTCCTGCTGTTCACGAACATTTATAGTTTCCAACAGTGGAAATGTGTCCCAATGAAA
AAATGTCATCATTGTTAGTCTTGGTTCCAAAGCCATGATGGCTCTATGTTTAGGAATCAG
GATAAATAATCATTGTAGATTAAATAAACTGTACTGATACAGAAAAAGAAGGTTTTATGC
TAGATTGTGTGGCAAAATTTGTGTTTTGTGGAGTTTCAGCCTGAGGTGATTTGGCTTAAT
TTGTTTTTCAGTATTGACATATACTAGTTTTCCTGTACCTACATATAAACTTCAGTATGC
AACACTTGAATATATGTAGTTATATCAAAATGTCCTACTTACGACATGATTAATCAGTTA
ATCACTACTATGAGTTTCCGTTGGAACAACTTGCACTTACATTGCACTGGATTAAGTGGA
CTTCAGTCTTCAGGTATAGCTGAAATAGCAAATGATTTGTCCTACTTACGACATGATTAA
TCAGTTAATCACTACTATGAGTTTCCGTTGGAACAACTTGCACTTACATTGCACTGGATT
AATTGGACTTCAGTCTTCAGGTATAACTGAAAAAGCAATGATAAATTGATAATTCCTATG
ATACCAAATTTTGTTATAACAAACACCACACATTTGGAGCTACAATCAATTGCAAACCTG
TGGTTATACAGCTATGTAATGCATACAGAGTGTTATCAAATAGCTGATGTTTAAGATTCA
AATGTCTGTCCTGTCCCATTTGAAAAAGTGCATGTCAATACCAAAGTGAACTCCTAAATT
CACGTTGGTATGTCCATTCTCTAAGGTCCTTAACAGTGGGTAATGAACTGTTGAGTTTCA
TGCTTCAAACAGTTAAAACCTAAAGCTTAAGCTGATGAGGAAAGGTGGGCAATATACTCA
CAATTCAACATGGAGGACTAGTTATATATAATCAAATATGCTGCAGTTTGCTTGTCAACA
ATACTACTTAATTGTTCTCGATTGAAGTATATCTCAAATGCATATGAGCCACATGATGTT
CGTTCACCTTTGCAGATATCTCTGCTCCCAACACTTGGATGTTCTTGAGTGCCCATACTT
TGGTGATGGAATCCTTTTGATTGACCGATTGATCAATTTCTGTAGGTCCGGACGCATCTG
CAACCGCTTCGAAATTAGGTGGAGCACCGGATCTTCAGCGTGCTCTCTCTCTTCTGTCAG
CTAGTTCTTGTGGATTACCTGATCCAGTACAGCAAGCATCTTGTCTCGTCCAATTCACTG
GTGCCAGCCAAAACAGCCGGGGTCTGCCTTCACCACATGGAGGGAGCCCTCCGTCGGCAT
CCTGTGCCGAGGGACAGCCCATGGCTCCATCACCTCAGTTCGTCCGTTTCACGATGGATG
GCGCCAGCAGTGGCTATGAATCCACTTTCTTCGGCGTAAACCGGATGAATTGATACGCGA
GACGTATGCGCCACTGTGTGATTTGGATCTACGGCATGGAAGAGCTTATGCAAGAACTGC
TCCCGTCAACTACTAGAGTGGAAAATCTCACTGTTCATGTTACCTACCTACATACTCATT
GGTTACCTTGTTGTTTTCCGATGTACAACCCTGCCGCCGATTGTTGGCTCGTTTTGTAAC
CATTGTTGATGGATCTGGACACAATTTGTCTCATCAAGTGATGACTTTGTATATTTTCTT
GTCCCTGACAAGCCATTTGCACAAGTAATCTCCATTACTAATTTGTGGCGATTTTTTTTC

CTTTGCTGCTGTAGCAAGGACGATATGATTTCATGTTTGTTATGAAGAAAACACGAGGAT
TTCAAAGACGATATTGCGGCGATATTTGCACTAATAATCTCCATGATTTGCTGTTGTATC
GTAAATGATATGATTTCATATTTGTTATGCGGATAACACCAATTCGTTTTATCGTGCAGG
CAGAAGAGAACAGTCGAATTTGACTTGCCTGCAATTTAAACTATTTTCTTTCTTTCTGTA
GAACGTGCACACACATACATCAAAGAACAGACTAATCATACAATACCAGGTAAAGAAAGC
GATCTTGTCTCCTCAAAGCAAACAATCCAAACAGCTTGTTAGGACACACAAGATGTAGAA
ACCAGTCGTCATCACAGTTGTCTCCAATATAAATCAGCTTGAATCAGACCAGTATGTGGC
TGATGTTGAGGCAGAACAGCCAGGGCAAGCCACTATACTATACAGCTCAACAAGATGTGA
TTTCCAAAACGAAACCACCAGCTGGATTGCTCATATGTTAAAGTGTCGCCATGATTATCA
TAAACTTTGGATCAAGTGACAGTGATTGATCCAATTGACAGGTTACCAACCAATCAAGAA
TTGGTGTTAAAACAAAAGCAAGAATTTTATGAACCACAATATAGTCTCCAGGCAACCAAC
ACCGCAGAAATCAACAT
>HORVU7Hr1G042370.8 genomic sequence
CGGTCGCACTATTGAAGGCAAAAAAAAGCGTGCGCGGTATAAGTGGTTCAGCGCACGGGG
CGAAATGGCATCACACGCCGTTTTATTTCGTCTATCCGCTCCCGCGGCACACTCGAATGC
CCGCGCCGCATCCTCTCCGACAGCGCCGCTTTCGCTCCGCCCGCGCTACTGTCGGCGAAT
TCGCCCGATGGACGACCGCGTGGACATCCCCTCACCCCTCCCTACACCCGCGACCTCGCC
GCTGCCGCCGCGCAAACCCTAGCACGCGAAGCTTTGGCTTCGCCTCCGTCGGTGCTCCTT
CAACTACCGGTCTTGCGCGTGGTCTTTTCATGGCGCCACAGACGATCTCGGCGGCAGGTG
GCGTCGTGCCGGCGCCCGTCGTGAAGCCACGCGCCCCCCCTCTCGAAACTCCCAAATGTG
GCGATGGAGAAGACGGGCAAGGTGTCCGGGAAGAAGAACAAGGCGGCGGACATCTCCTCT
TGTTTTGATGTTTGTAATATCACCGTATTGTCAAAAATGAACATATTGCAAGCGCCGGCT
ATTCGCGCGCGCTGTAGTTTAGCGCGCCTGCTAGAGCGCTCGCGCACCCTATTTTTTACG
GCGACCCCTGGAGCCAGCACTCTCCGGCGTGCTAAAAATCACTATTTCAACATGCCAAAC
TGTTTTTAAGGTGTGGGATGATTGCGCGGCTGTTGGAGATGTTCTTAGCCAAGATGCACT
ATTGCGGTGGGCGAGCACGCTGGGTAGTGGGGATGGGAGAGGGGTCTCGCTGGTACGAGC
TGTTTTTCTTTTTTGTTGGCGAAAAAGTGTGATGGACGGTCGTCAAACGCAGCATATCGT
GTGGTGTGGGAGGCACCGATCGGTCGACCGACGCCTAGTACCTGCCCTCGCCGAAGAGGG
CGCGCAGGCGTTGACCCTCACAGGCGTCAGCAGAAGGAGAGCGCACGGGCTCAGCCGAAG
CATCACCGGCAGCCGTCCTGCCGGCCCATCTCCACCCCCCTGAATGAAAGACAAAGGAGT
GTCCTGCTCTTCCAAGGCTCAAATGGCGTGGTGTCACTGTCCTACTCCCAAGCCGAGACG
AAGCCGCCAAGCGCCAGTTAAGGCGGAAGAAAAGGAGGCCTCCCCGATGCGAATACGCCG
GCGCAGGGAGGTCCTGCCTGCCCAGGATACTTCGCCCCAGGTTCTGCCAATCCCCACTCT
TCGCCTCCCCTTGATTCTTGACTGATTTTTCTGAGCTCACGATTCGGTTCCAAGATAGCA
AGATAGTCCGGTCGCGCATGGTGGCCTGTGCATGACTAACCCGGCAGCGCTTACGCATAT
GTACAGTTGGGGAATTTAGTTGAAAAATTTCGAGATATTTGGCTGTTCGTGCCTAGAACA
GAACAGGGGAAGGGGGGTTGGCGAGAATGTCTAGTGGGCTCAAGAACAAGAAGAAGAAGG
GCCACGAGTGGGATTTGAACGACTGGAGATGGGACGGCAACCTCTTCCTGGCCGCACCGT
CGTCAAACGCGGAAGCGCCGTCGGGGTGCGGCAGCAGGGAGCTCGGGCGGGCTGAGGAAG
GGGGCAGTTTTGGTGCTGCTGACAAGAGCAGGAGGAGGAGGAGGAGAGTTTCCACGGTGG
ATAACCCCGAGGAGCCTCCACATGAAAGGATTGCTGTTCGGAGAGGACAGATCAGCGAAG
AAGAGAGACCTGCGAATGCAACAGCAGGTGCATCTTCCAGCTCTGCTCCATCTTGCCTAG
TCGACGGCTGCCACGCGGATCTCCGCGACGGTAGGGACTACCATAGGAGGCACAAGGTGT
GTGAGGTGCACACCAAGTCGACTCTTGTTCGTATCAAAAACATAGAGCATCGGTTTTGCC
AGCAGTGCAGCAGGTCAGTTAGCAATAAATTTCTTCGTATCAAGTTTTTCATGTTATGGT
TTGAAGGGAAATCATTTGACATTTGTTCTAGGTTTCACCTTGTTCAAGAATTTGATGAAG
GGAAGAAGAGCTGCCGCTCACGTCTAGAAAAACATAATGGGAGGAGGAGGAAAGCCCAGG
CCCAGGCTGTGAGTTCCTCGAGTGAAAATCAGTCCTTAACCAATACCTTACTCCTCTTAC
TGAGACAACTCGCTGGACAAGATTGTAAGTTTCTCGGATTAATTCTATGTGCAGTACAGT
GGTACACATTGATTGTTACCTGTCCATATGCGATCGATCCATGCTCATATAACCTCAGTG
TTATGTCGTCGCAGCTGCTGCTAGCTCGTCTGAGCAAATCAATGGTCCGAATTTCTTGGT
CAATCTTCTGAAGAACCTTGCTGCTGTTGCTGGCACACAGGCATGTCAAGATATGCTAAA

GGATGCAACATCATCAAATGCTGGTAACTATGTTGGTAATCAAAGTGGACCACCAGTTCC
TGCAGGTAAATAGCCTTTGATTAACTGATGCTGCTGCTGCTGTTGCACAAGGCAAATAAG
CACACATTCTGAAATTAAATGATGTGTGCACCACTTGACATGTGTCCCCAGAAGAGCCTC
CTATGAAAAGACGTGCGCGGAATTTTGATCTGAATGACACTTATGTCGAGGAAGATGAGG
TACATTATTTAGGTTGATTCTCTTGGGTTTCCNNNNNNNNNNNNNNNNNNNNNNNNNNNN
NNNNNNNNNNNNNNNNNNNNNNNNNNNNNNNNNNNNNNNNNNNNNNNNNNNNNNNNNNNN
NNNNNNNNNNNNNNNNNNNNNNNNNNNNNNNNNNNNNNNNNNNNNNNNNNNNNNNNNNNN
NNNNNNNNNNNNNNNNNNNNNNNNNNNNNNNNNNNNNNNNNNNNNNNNNNNNNNNNNNNN
NNNNNNNNNNNNNNNNNNNNNNNNNNNNNNNNNNNNNACTGATCTACGCACACAGGTCAT
TTATTGTACGCAGATTGTAAATAGTGCCTCTCTTGACAACTTTATCTCTTCTGAATTTTT
GGATCAAATTGTTTTGATCTGCAGGTCCTAAACTGGCTGTCACATTATCCAAGTGACATG
GAGAGCTATATTAGGCCTGGTTGTGTAATTCTAACTATTTACCTTCGTCTTCCTAACTGG
ATGTGGCATAAGGTAGCGCCGTGTTATATAAATACTAGTGTTTAAATAAGCTGGTTACTA
GTGAACAAACTACTTACTAATTCAAATAGCAAAACCTGAACTATGAACCTTGGATAGAGA
AGCATAATCTTTACTTACTATTATTTTCCTTTTGTCTGAATTTCCCTAAAGCTTAATGTC
GATCCAGCTCCTTGGATAGAAAATCTTATTAGCATATCCACTCATGGTTTCTGGGAGACA
GGATGGTTGTATGCTAGGGTGCAGGACCGCCTGACATTAAGCTGCAATGGTTCGTTTGCC
TTATTTTTCTCTTTTTACACCTTTTTTTCCCTATTTTTATCTGTATATGTACACAACCAA
CAGTTTCCTATGTTTGTGGTTTCCCTTTCTTTGCAGGCAGGCTTACGTTGGTGTGTCCAT
GGCTACCTGTAATAGGTGACAAGCATCAGATACTGTGTGTAACTCCCATTGCGGCTTCTT
GTAAGTCGACGGCAAACTTCTCAGTGAGAGGCTTCAACATAGCTCAACCAACCACAAAGT
ATGTACCAGGCATCACTCTGCACTGACCTGACCTTGATTTTATTTCACTGACAATAATGA
TGATACTCGTTCCAGATTACTTTGTATATTTGGTGGGAAATATTTAGTCCAAGAAGCAAC
ACAAAAACTACAAGATGATACTGCGGTTCAGCAAGGGCCTCAATGTCTGGCCTTTTCTTG
CTCCTTCCCTAGTACAAGTGGAAGGGGGGTTCATAGAGGTGAATTATCTTCTACATTCTTT
CAGTTGTCTCTCTTTATACATCCTCATTTCCCTTTTTAAATTACGTACTTTCTTGGTTCC
TTTTCCAGGTTGAAGATTATGATCAGAGCAGCATTTCATTTCCCTTCGTTGTCGCTGAAG
AATCTATATGTTGTGAGATTCGAATGTTGGAGGAAAAACTGAATATATTTGCATTTGCTA
ATGTCTTGGAAGGAAGAGAAGATCTGATGGCTTCTCGCAGCCAAGCCTTGAAGTTTCTAC
ATGAAATAGGGTGGCTTCTTCAACGGAGCCACTCACGAGCTACATCATCCAAGGCTCCAC
AACAACATCGTGTTGTGGGTTTTCTTGCTGCAAGATTTAGATGGCTCCTGTCCTTTGCGG
TCGATCAGGAATGGTGCGGCGTCGTAAAGATGCTGCTGGACACCTTGTTCCAGGGCAATA
TTGACGTCCCCTCACCAGTTGAGTTTGTCCTGGGAGAGAGTTTAGTGTTCAAGGCTGTCA
ACAAGCGGTCCAAGCCCTTGGTTGACTGCCTATTGAGATACACAACGAATTCTGCGCCAG
TGGGCGGTGGAGCAGTGGCTACACCAGCTCGGTTCGTATTCACGCCTGACATGACTGGTC
CATCAGATATTACACCTCTCCATAGTGCAGCTACCATCAGTAATGCTGCTGCTGTTTTGG
ATGCTCTAACTGATGATCCTCAACAGGTACTTGCACTGAACTCTCATGCTATTTTTTTTC
CTGAAACGATAAATTCTCAAAAAAAGAAGAAAAACATAGTGTGTGTGCTAATATTAAACT
TATTGAGTAGTCACAGCAGATTTGTACATTGGTGGTTGGATCAAAGATGGAGTTGCCTTC
AACATTTTCATTTACTAATGAGAATTTGTTGCAGACATTGAAACTGTGGTTACTGAGTGG
CTTCTGCTTGCAATATTTTCTCCTTGTCTTTCCTTTTCGTTCCTGAACTCACTCGGCCTA
AATTTGACAGCTAGGGATCAAAGCCTGGAAGAATGCCCGCGATGCGACCGGGTACACTCC
AGAGGATTACGCTCGGAGGAGAGGGCACACATCCTACATCCAGATGGTCCAGAACAAAAT
TAACAGGGGGTTACCTGCAGCCCATGTGTCTGTTTCCATGACCACCACTGGTATCACCGA
AGAGCATGCAGATGCAGGTCGTCCGAAATCAACTGATCAGACCATATTTGATGTCGAGAA
AAGCCTACCGGGATGCAGACAATGTGTCCAGCTCCAGCACATTGCCTACCGGCCCTGCCC
AAACAGGTTCTTGTCGAACAGGCCTGCGGTGCTATCCTTGGTCGCCATTGCCACTGTCTG
CGTCTGCGTAGGTTTGATAATGCAGAGCCCGCCGGTCGTCCGCGGTATGCCGGGTCCTTT
TCTCTGGAATCGCCTCCGTTGGGGACCCACTTGAAACGCCCAATCAGCTGCAGATTCTGC
TAGTCTGTACACTCGAGAGTGAGGATGCTTGTGATAGACCAAAGTGCAATCTCTGTACAT
TATATGAGTTGTGGAGTGCCTAACTCTGCTTAGGTAGCAGTAGTTCTATTGGATATTCGA
GCTTTTCATTGTTGTACTGTGAGTCAGAGAAACAATCACAAAGGTGGTTGTCTAGGGAAA

GCAGATCTATTTTCCCCTCTCTTGTGTATATTAATTACTCCCTCCGTCTCATAATATAAG
AGCGTTTTTGCCACTAGTATAGTGTCAAAAATGTCCTTATATTATGACACGAAGGGAGTA
ACGTCTATGCTCCAACAAGTAATGTTCTGTGGATCACTAGGTGGCAAACCTATTGCAATA
ATAGTTTTAATCTCATCTGCATGAGGCAAATCTTTAGTAAGATTTTGTTTAGAGAACTTA
GAGAAATTTCAACTTTATCATCTGCAAGTTAAACATCCACTTTTTTTCTTGGCAATCAAC
TACAGTTCCAGAAGTACTCAAGAAAAGTCCAAAGATCATGGGACAAAAATTATCTTGTTT
AGTTCCAAGCACTAAGAAATCACTGGGATACTTAGTCTAACCACAAAGAACTTTAACATC
TCTAATAATTCACATTATTGATATGCTAGACATGTCTACAAGCTTAATAGTCAAATCAAT
ACGGAATATCCAATTCTGAGCCTGGGCTCATCTGCATCCGGTCAGAAAAAAATTCAAACAA
AATTCAAAAAATTCCAAATTTTTTGTGCAAGGTAGATAATTTGATGCGTGAGGAGCGCTC
CAAATTTTAAACCATTTGGACATCTAGGTCTCAGCAGAAAAGACAAATTGAAGTCCGAAC
AGTACATGAACAATACACTTTTTAGAAGATCTTAAATTTATCTTTTTTGTCGAGAGCTAC
TCGGATGTCCAAATAATTTGAAATTTGGAGCGGACATCTCGCACCAAATTCTCTACTATG
ATAAAAAAATGGAATTTTTAAAAAA
>HORVU5Hr1G073440.7 genomic sequence
CAAGACACAAAAGGAAATGCAGTTTGCCCTGATGCGTGCAACCCCTCCCTCGTGCGCTTG
CACGCGGCGCCCGCCCGCGTATAACAAGTGGAGCCGGGCCGGGCTGCCACAGCCGTCCCA
TGCTCCCACAAATACTATACTGCCTCCTCGAGATGAGCTACTCGTCTAAAAAGTAGACGA
CGATGATAAGAGGCAAAGAGCAGAGCGTAGTAGCGGCCGCTAGAGGGCGGCGTTTTGGCT
ATAAAAGGGTGGCAGCATCAGCAGCTGGGGAGAGTGAGGTGCGTGTGCGTGTGCGTGTGT
GGAGCAGAATAGTGGGAGCGCGTGAGCAAAGCACACTGCACCACACACTAAAAAAAGGGG
TTTATTTTAAGCGCGGCGCCACCATTGCTGTCGCCTCCTCCCTCCGCCGCTTTCCATCTT
TTCGCTCCCACCCCCCTTCCCTTCCCTTTGCCTCTCTCTCTCTCTCTCTCTCTCTCTCTC
TGGTGCTGNNNNNNNNNNNNNNNNNNNNNNNNNNNNNNNNNNNNNNNNNNNNNNNNNNNN
NNNNNNNNNNNNNNNNNNNNNNNNNNNNNNNNNNNNNNNNNNNNNNNNNNNNNNNTACGGGC
GTTTTCTTTCACAAAGGAGGAGGGGGACCGGCGGCGGCGGCGGCGGCAATGGAGACCGGG
AGCAGCGGCGGCGGGAGAGGGCCCGGGGACGACGTCCACGGGCTCCACTTCGGCAAGAAG
ATCTACTTCGAGCAGGACGTGGCCGGCGGGAGCGGCTCCTCCTCCTCCGCCTCCGGTGGC
AGGAGGGGCAGGGGCCCCGCTGGGCCAGGAGGAGGGGGAGGTGGTGGTGGCGGTGGCGGT
GGCGGCGGCGGAGGAGGAGGAGCCTCCACCCCCGCGGCCGGCGGCAGCGCCTCGCAGTCG
CAGCAGCCGAGGTGCCAGGTGGAGGGGTGCGGCGTGGATCTGAGCGGCGGCAAGACCTAC
TACTGCCGCCACAAGGTGTGCTCCATGCACTCCAAGGCGCCGCTCGTCGTCGTCGCCGGC
ATCGAGCAGCGCTTCTGCCAGCAGTGCAGCAGGTACTCTCCTATCCTCTCCTTATCACAA
GCGGCAACCAATTTGAGCCGAGCCTCGGATTGCTCCATTTTTGGTCCTTCCGGACTTGCC
GCGTGTTCTTGCGCCACTGTTACACTCGTGACACACACGCCTCGATGCCGGCGTAGTGGT
TGATGGATCCTGCAAATATACTGTATTTTTCTCTCCCACGACTACCTCCCAGCATGTGGC
TAGCTACTACCACCAGCAGCCAGCATGCAAGTGTAAAAAAGCTGCTCAGGTTCTGCAATT
GGCCGGCCAGCCTAAGCATTTTGCTCTACTTCCTTGCTTTGCCACATCCAGAAAACATAG
AAATTCTCAAGCGATGACGCATGGCGGTGTGTAGTGCCGTAGTGGGCATTGGTGAAAAGA
AAACAAACAAATAAAACAATGTAACATTTGGTTGGGCAGGTAGAATCTGACCAGAATAGT
AAGGGACAGAAAGGTGTGCAACCTGAAATTCTTGTCCGAGTGGCGCATATAGTAAGTCGA
GATTTCTTTTTTCCTCTCTTCCTTATTCTACTGTAGGCTATACTAGGATGTGAGAAGAGA
ATGGGTAGTTCCAGCAGTAGTACAGCCATGGTACCAGTGCTACTGTAGTAACTTAACACC
CTACCCTGGTGATATTCTTATACTCCTACGTTGGTTGGAAGTGAAATTAGGTAAAGAAAA
ATGGAATGCCAATGGATTATCAGGTTGAAATCAGGGTGCACTAATCAGAAACCATCAACT
ATTGTTTAGTTCATCTCCTCTGTGTACGAATACGGTGTTTGTTTTGAGATTTATTTGAAT
GCATGATTCTAAAAACGCGGGAATAGGAAAAACATAGATTGGGAAAAAGCATAGGATTGG
AATGTCATGCTCATATCAATCCTACAGGATTTTGGGCTGTTTGATTGCATCGTAGGAAAA
ACAAAGGATTTTCTCAAAGATGTTTGAGTGGATGCTAGAAATCCTATAGAAAGTAGTACA
AAAATAGTACTCCCTCCGTTCCTAAATATAAGTCTTTTAAGATATTTCACTATGAGTCTA
CATAGAGCATAATGAGTGAATCTACACTCTAAAGTATGTCTATATACATCCGTATGTAGT
CCACTAGTGAAATCTCTTTAAAGACTTATATTTAGGAACGGAGGGAGTAATGGGAAAAAT

TCCTAAGAATTCCAATCCTCCAAAATTCTATGAAAATCCTCTGAATCAAAGGAGCCCTAA
TGTTTGAAGATAAAGCATCACTTATGACTGTTGTTTGAAAGATGAAAACATCACTTATGA
CTGCTGTGGTAGCATAAGCCTTTGCACGTTTCCACTATCTAGGAATGATTACAGGTGTTC
TATCCACATGGTCCTCTTTCCCCCCCTGGTCGTGATGCGTGGATCTAATCATGAAAAGTC
GTTTCTTTGGTTACTGCATTAGTCTAAGAAAAACCCATTGGTTTTGCTTGCGCCGATGCA
GCTTTTCACATGCCGTGTGATTTGCCCTGATAGTTTATTCATTTGATGCATGCTTGGTAT
TAGGATACTAAGCTAGAATCTGGCTCTTGATTGCCGGTGTTAGCGCTCCCCCTCTTTTCA
TGTGCACGAAGTTTTTTATATGGATTATCAGAATCAATTTGATATGCAATTGGTGTAGCT
AGTGTGGAAGAGTCAGACACTCCTCACTCTGCTTTTGTTGCGTGCAATTGATTATTGTCG
TCCATCTCTTGTTCACTGTCTAAGTGGATCTATATGAAATTCCTACTTACCGTGACCCTT
CGGTCTTTGTCAATTCGCATTGGCTCTGCTTTGAATGGATCTGCACTATAGTAAAGCCAC
CATGTGGAGTTCGTACGTACATGCGGTAGCAATAATCCTTTGTGATCACAGGTTCCACCA
GCTACCTGAATTTGACCAAGGAAAACGAAGCTGCCGCCGACGCCTCGCGGGTCACAATGA
GCGCCGGAGGAAGCCGCCGCCCGGACCTATGTCGACACGCTATGGCCGGCTTGCTGCGTC
CTTCAACGGTAAGAATGTTATATCACAGGATCCACACGCTCGCAGTTTGAACGAAACGGT
ATTGACTCTGCCGTTGTTGGAAATTTTTGTTCAGAAGATCCAGGCAGGTTCAGAAGCTTT
CTGCTGGATTTCTCGTACCCGAGGGCCCCGGCCGGCGTGAGGGACCCGTGGCCGGCCGTT
CAGCCCGGCGACCACCGCATGCCTGGCACCACCCACTGGCAGGGCCAGGGCAGCCATCAT
GAACAACACCACGCTCATCGCAGTGCAGTTGCGGGATACGGCGACCACCACGCGTACAAC
GGCCAGGGCAGCAGCTCGGGGGGAGGCGGGGCGCCGATGATCCCGGCGGGCTTCGAGCTC
CCCTCGGACGAATGTATGGCGGGCGTCGCCGCCGACTCCAGCTGTGCTCTCTCTCTTCTG
TCAACTCAGCCATGGGATAGTAGTGCCCACAGCTCCAGCCACATCCGGTCCCCGGCAATG
TCGACGGCCAGCGCCTTCCAGGGCAGCCCGGTGGCGCCCTCCGTCATGGCCAGCAACTAC
ATGGCGGCGGCGAGCAGCAGCGGCTCCTGGGGTAGCCCCCGGGGCGCCAGGAGCATGCAG
CAGCAGCACCACCACCATCACATGCAGCATGACACGGTGATGAACGAGGTCCATCCGAGC
TCGGTTCACCACGGGCAGTTCGGCGAGCTCGAGCTGGCGCTGCAGCAGGGGAGGGCCACG
CCGAACCCGCCGCACGCCGAGCACGGGGCCTTCAGCCACTCCAGCAACGCGATGAACTGG
TCTCTGTAGGGACGGGAGACATGGGACGTTGGGCGGCCTGAGTTTTGTGAGCCGTAGACT
TTGCCGATCTGGATTTCAGGACGGGGAGGGAACCCGTAGCTGATAGAGCACAGGGGTTTC
CCGCCGTGTAGCCCAATTTTGTCCATGGACTCAATACCACATATGTATGTACGTGTGTGT
GAAACTCCATTGCTTTATTAATTCGTTTGTCAGAGGACTAGTAATCTGATGATCTCGCCG
TCGTCCTCCTCGTCGCTGTTTCGGTTGCTATTTTAGCCAGCCTCCTCGTCGCTGTTTCCG
TTGATATTTTCGCCAATTTACATGCGTTATTTCTTTACATAAGCAAAAGCGCAGGGGACA
TACATACATATGCTCACTTCTATGCATGTTTATTTTTATCAGAAGAAGGGCTTTTCCTTC
GGAATTCTACTAGCACGTATGCCTGTGCGTTGCAATAAAATATTTTCTTTTTTACAAGAA
GCAACATGAGAGACATGATGCGTCCTTCAAAAAAAGTCTCCGCAGCGGTGACCCACAGAT
CAGGCTCTAAGTGGGACGCTCCGCGGAGACCCGCGTCCGCCTTGCATCTGGTCGCGTAGA
TTAAGTGGGATACTTGTCAAAGCCCACATATTATCATGTGGGATATGTGGATTTAGTGGG
ATACCTACTAAATTTATGTCGATATACCCTGGTATTGAAATACGTCGGCGTTGTTATCTG
AGATGCTGAGCTACCATGACCGGTTTCTTGGGAGCTCTAACAGATCATGTGCATGATGCG
CACGTACAGCCGGCATGCCTTGGCCTAGTGCTCTTGTGCCGTTGTACTGTGCATCACCAA
TTTTCCTTCTCCTATCAATTGCAGATTCCTCCTTTTGGAACTTCTTCTTCTAGATTCTTG
ATTGATGCCTGGGGTAGGCCTCTTCTCGCAGTCCTTTCGAGCGGCGCCGGCGGCAGGAAT
GGTGGAGAAGGAA
>HORVU2Hr1G097610.1 genomic sequence
TTATCGGAGGTCTCATACCTCTCGAGCCGGGCGTTCTTCTGAAAGATAAACTTCAACTCC
TGGAACATCTCAAATGCTCCATGATGCTCAAAGCGACGTTGAAGTCCCGGTTCTAAGCCA
TACAAGACTGCACATTGAACTACTGAGTAGTCCTCCTTACGTGGTAACCAAGCGTTCTTA
ACATCCTGATCAGCCGTAGCGGGTGGTTCATCTCCTAGCGCAGCATTAAGGACATAATCC
TTCTTCCCAGCTTGTAAGATTAGCTTAAGATTACGAGCCCAGTCTACAAAGTTGCTTCCA
TCATCTTTCAACTTAGCTTTCTCTAGGAACGTATTAAAATTTAGGGTGACTGTCGCGTGA
GCCATGATCTACAACACAAATATATTCAAAGTGGACTTAGACTATGTTCAAGATAATTAG

AGTTTAACTTAATCAAATTACTCACTAAACTCCCACTCAAAAAGTACATCTCTCTAGTCA
TTTGAGTGGTTCATGATCCNNNNNNNNNNNNNNNNNNNNNNNNNNNNNNNNNNNNNNNNNN
NNNNNNNNNNNNNNNNNNNNNNNNNNNNNNNNNNNNNNNNNNNNNNNNNNNNNNNNNNNNC
CTAGCCACCGCCGGGGGAACAGCACATGGAAGGAGACGGCGACGCTAGGTCGAGGGCGGG
GCTGGCCTGGGATCTCGGGATGCAGCAGTGGGCGCCCGCCGCCTCGGCTACTTACCCGCA
GCATTTCCTGCCGCCGCCGCCGCCGGGGGTGGCTAGCCACCACCGGGGGGGGGGGGGGGC
TAGCCTAAAGCTGGGTAAGCGCCCCTGCTCGCTGGCCGGCGTCCAGGTGGCTCAGGTGGA
CGGCACCGGGGCTGCCGTGGTGGAAGGCAAGAGGAAGGAGAAGGCGGGCGCGCCGGCAAC
GGCGGCCATGCCAAGGTGCCAGGTGGAAGGGTGCCACATGGTGCTGGCGGGGGCCAAGGA
GTACCACCGGCGGCACAAGGTGTGCGAGGCGCACTCCAAGGCGCCCAGGGTCATCGTGCA
CGGCGCCGAGCAGCGCTTCTGCCAGCAGTGCAGCCGGTACGTAAATTCTTTATGAAACAC
GCCCAAACTTCACCCTGTGCTAGCTAGATCCATCCATTATGTACACGTACACGTACGTGC
GTACCTGCAGAGTCTCTCTCCTACGCTAGTTAGCGTTGACTCTATTGTCTGCGTTTACAT
GCGTGCAGCTTCCACGCGATGGCGGAGTTCGACGACGCCAAGCGGAGCTGCCGCCGGCGC
CTTGCCGGCCACAACGAGCGGCGCCGGAAGAGCAACGCCAACGACGCCATGGCCAGGGGC
ACTGCGCACGCACACGGTAATCCATGTCATTACTCGCACACGTCCATCAGCGCGAAATAC
TCCTGTTGACATGGATGTGACAGCGTACAAGTTAGAGAGAAGAAACATTGTTAAGAGCAA
CTCCAACGCGCGCGGATCCATAGGAACGACGTTTTTCGGTTTTCTGTCCGTTTTAATTCG
ATCGCGCGTTTAAGATTTATAAATCGGCACTACGTCTAACGTCCATAATCCATTTTATGA
CAGCATATTAGTTAGAGCAACTTTAACGTGCCGACACAAACAAATGACATTATCGTTCGT
TTTTTGTCCGTTTAAGTCGGTCGCCCGTTCGGGGTCCGTCTTGTTTAACATTTGGATCGG
CAGTGCGCCAACGCTCATGACCTATTTCATGTCTGCGTACATATTTGAAAAGACTCGCGG
CCGTAGATCAAGACAGTTGCCATGAAGTCGTCCAAAGTTCATGTCGGTACCATGATCGAG
CGGGAAGCGGTCCAGGTCCAGGCCAACCTGATCTTGCACCAAGGCGGCATGCATGCCAAC
TTGATCATAGGTCGACAGCGTGAACGGTCCTCGGTCGTCCTCGTTTTGGGTCTCTTCGGG
GTCGTAGCCAGCCGTGGCGGCACCGTCAGCGGCCACTGTATCACCCTCGAAGATGATGCT
TTGCATGAAACAGTTGCCCCTCTCGTCGTCGACGGCCACCGACATTCTGCCGAACAGATT
ACGGACGTCGGGGAGCATGTCGGCTGACATCTCCCGCGTGTGTTTTCACGGCCCACCCGA
GATGAGCCACCGGCTACTGGCGTGACGTTGAGGTCGATGGGCGCGGACACGGGTATGGAA
GGTGCCACCGTGCTCACCTTCGGTGAGCATTCCCCGAAGAGTCGGGAGGCTTCCGGGTAG
TCATGGAAGCCGAGAATCGGGCGAGTCGCCGACGCGTCGGGCGACTCTGGTAGCACCATC
CAAGGAAACACACGGTGTTGGCCGCGGCCACGACAGCGCTGACGAGCCCGTGCTGGCCAG
GGTTTAACCCTAGGTACAATAGCACCTCCCTTGTTGCCGCCGCGACGTGGGGGACAGTGT
CCTCCTGCTGCGCGACTGCGGGTATGGCTGCGGCGGCCGTTGCTTCATCCCTTGCATCCG
CTGCGTGCCTCCGGCACTTTATCTTCGCCGACCCAGTAGCCCGCTCCTCGGCGTCAGCTT
CTTCTTCTTCTTGGGCGCGGTGGCGGTCTTGCGGGGGGCGTCGGGCTTGCCTTTGCCGGA
GGGGACGGTCATGATGCCGAACAAGGCAAGGGACGCGAGGCCGACTGTGGCATCGAGGTC
TTCGTCCATGGCGAGCGGCCATTAGCGCACGCGGGCGGAAGAGTTTTGGGGAAAGTGGAA
GGAAATGGTGGAGGCTTTGCCACCGACTAATGGCCACGGAAGTAGTTTGCATGCGGCGCG
CACGTCCATCTCGTGTCCGCACCGACGTAAATCAGACTCAAAAATGGATCGGAAATGAAT
CGTGAGGATGATAAAAAATGGATGCGCGTCCGTTTGAATTAACGTGTTTGGTCGATTTTT
CTGTCCGCCGCGATCCAAACGAATGCGCTCAGANNNNNNNNNNNNNNNNNNNNNNNNNNN
NNNNNNNNNNNNNNNNNNNNNNNNNNNNNNNNNNNNNNNNNNNNNNNNNNNNNNNNNNNN
NNNNNNNNNNNNNCACTGCTGACTTTTTGTGTGTTCAAAATGCACCATTCAAAACCACGT
CATCAATTTTCAACTATTTGTGACTTCATTTGGTATTTTTCATGCATTTATTGATATTTT
TTTTGAACTAAATGACCCAGAAATTGAAAAGCACTAAAAATAAACTCTGAATAGGTTGAA
AGTTGGCATGGTATCATCATTTCACCCACATAGCATGTGCTAAAAAGTTGAGAGGGTTGC
GGCAAAAACGGGACGCATTTCGTGTACAAATCGGACAATCTCTTTCAAAGTATCATAGTT
TTACGAAAACTCATCTGTTACAAAAGGGATTTCATTTTTTTGAACTTATTTGAACTCCAG
ACTATTTGTGTGTTCAAAATGCACCATTCAAGGCACATCATCAATTTTAACTATTTCCGA
CTTCATTTGGTATTTTTCATGCATTTACTAATTTTTTAGCTAAATGACCCTTAATATGAA
AAAGACTACAAATGAACTCTGAAAATGT

>HORVU2Hr1G097580.1 genomic sequence
TTCCCCAGCCAATAGGGCCTTCGCCACGCCCACTGCTGACACTGCCAACCCGGACCACCC
GACGTACCGGCCCCATCCTGCTCTGCCCCTCCCACTGCATGCATGTACACCCCGCTCTGC
TGCGCAGGCCGGCCCTTTCCCCCCTCACTGCTCCGTTACATCGCCGCACTCCGTCCATCC
CCCATGACGTGTGGGCCATGATCTACCTGGTCCCACCTTTCGGCGAGAGTGCACGGCATA
GTACTGGTACGCGGTGCGGAAACATCCGTGGTGGCGTCCACTTCCGGATATGGCATCTTT
GTGCAAGTGGGAGGGGGCGCCACCCTCGTTTCGCTTCCCGCACCAGGCTGGAGCGCCGGT
CGCCGACGGGTGGGCCGGTGGCGTGGGTCAGGATAAGGTGGTCCCACGCGCCAGCGATTA
AAGGCCGGCCCGGGCGGGCTGCTAGGAAGGAGCAGTGCGTGTTGTCGCGCCGCGCGCGGC
TTTCGATGCGGCGGTGGTGGCAGTCATGTCCGTCCGTCCCCATGGGGGTGCGCAACGCGG
CCGGGTCCCGCCGCGGATTGAACCCCTCAATGGCGCCCATCCATGTGGCGACGGGCCCTG
CGCTGTCACGAGCCTGCGTCGTACCGCCTCCCCTACTTGCAGCAGCCAGCGGCAAGAGGC
TACAGTGGTGTGCGAGTACACTCCTGCTGCCAGCGGCCAGCTGCGCCTGCGTGTGTGTGA
GGACAGATCCCACCTGTCAGTACGGCGCTGCCGTGCCTTCCACACCAGTCACCAGTGCGC
TGCTCCTCCCCGGGGAGCTCGCCTCCCTCGCGCGCGCGCGAGCGAGCGAGCCACCTTTCC
TACTACTACTAGTAGCTTTTATATCGGCGCCAGTGGGATAGCCTACCTCTGCCTGTACTT
ACAGCAGGAGCAGCCGTCGCCGCAATTTCCACTTGCTCGTTTAGTCGCCACAGCCACAGC
CACAGCCACAGCCACAGCGGTTTTAGCCTAGCCACCGCCGGGGGAACAGCACATGGAAGG
AGACGGCGACGCTAGGTCGAGGGCGGGGCTGGCCTGGGATCTCGGGATGCAGCAGTGGGC
GCCCGCCGCCTCGGCTACTTACCCGCAGCATTTCCTGCCGCCGCCGCCGCCGGGGGTGGC
TAGCCACCACCACCAGCAGCAGCAGCATATGCAGCAGGAGCTCACTAGCCTAAAGCTGGG
TAAGCGCCCCTGCTCGCTGGCCGGCGTCCAGGTGGCTCAGGTGGACGGCACCGGGGCTGC
CGTGGTGGAAGGCAAGAGGAAGGAGAAGGCGGGCGCGCCGGCAACGGCGGCCATGCCAAG
GTGCCAGGTGGAAGGGTGCCACATGGTGCTGGCGGGGGCCAAGGAGTACCACCGGCGGCA
CAAGGTGTGCGAGGCGCACTCCAAGGCGCCCAGGGTCATCGTGCACGGCGCCGAGCAGCG
CTTCTGCCAGCAGTGCAGCCGGTACGTAAATTCTTTATGAAACACGCCCAAACTTCACCC
TGTGCTAGCTAGATCCATCCATTATGTACACGTACACGTACGTGCGTACCTGCAGAGTCT
CTCTCCTACGCTAGTTAGCGTTGACTCTATTGTCTGCGTTTACATGCGTGCAGCTTCCAC
GCGATGGCGGAGTTCGACGACGCCAAGCGGAGCTGCCGCCGGCGCCTTGCCGGCCACAAC
GAGCGGCGCCGGAAGAGCAACGCCAACGACGCCATGGCCAGGGGCACTGCGCACGCACAC
GGTAATCCATGTCATTACTCGCACACGTCCATCAGCGCGAAATACTCCTGTTGACATGGA
TGTGACAGCGTACAAGTTAGAGAGAAGAAACATTGTTAAGAGCAACTCCAACGCGCGCGG
ATCCATAGGAACGACGTTTTTCGGTTTTCTGTCCGTTTTAATTCGATCGCGCGTTTAAGA
TTTATAAATCGGCACTACGTCTAACGTCCATAATCCATTTTATGACAGCATATTAGTTAG
AGCAACTTTAACGTGCCGACACAAACAAATGACATTATCGTTCGTTTTTTGTCCGTTTAA
GTCGGTCGCCCGTTCGGGGTCCGTCTTGTTTAACATTTGGATCGGCAGTGCGCCAACGCT
CATGACCTATTTCATGTCTGCGTACATATTTGAAAAGACTCGCGGCCGTAGATCAAGACA
GTTGCCATGAAGTCGTCCAAAGTTCATGTCGGTACCATGATCGAGCGGGAAGCGGTCCAG
GTCCAGGCCAACCTGATCTTGCACCAAGGCGGCATGCATGCCAACTTGATCATAGGTCGA
CAGCGTGAACGGTCCTCGGTCGTCCTCGTTTTGGGTCTCTTCGGGGTCGTAGCCAGCCGT
GGCGGCACCGTCAGCGGCCACTGTATCACCCTCGAAGATGATGCTTTGCATGAAACAGTT
GCCCCTCTCGTCGTCGACGGCCACCGACATTCTGCCGAACAGATTACGGACGTCGGGGAG
CATGTCGGCTGACATCTCCCGCGTGTGTTTTCACGGCCCACCCGAGATGAGCCACCGGCT
ACTGGCGTGACGTTGAGGTCGATGGGCGCGGACACGGGTATGGAAGGTGCCACCGTGCTC
ACCTTCGGTGAGCATTCCCCGAAGAGTCGGGAGGCTTCCGGGTAGTCATGGAAGCCGAGA
ATCGGGCGAGTCGCCGACGCGTCGGGCGACTCTGGTAGCACCATCCAAGGAAACACACGG
TGTTGGCCGCGGCCACGACAGCGCTGACGAGCCCGTGCTGGCCAGGGTTTAACCCTAGGT
ACAATAGCACCTCCCTTGTTGCCGCCGCGACGTGGGGGACAGTGTCCTCCTGCTGCGCGA
CTGCGGGTATGGCTGCGGCGGCCGTTGCTTCATCCCTTGCATCCGCTGCGTGCCTCCGGC
ACTTTATCTTCGCCGACCCAGTAGCCCGCTCCTCGGCGTCAGCTTCTTCTTCTTCTTGGG
CGCGGTGGCGGTCTTGCGGGGGGCGTCGGGCTTGCCTTTGCCGGAGGGGACGGTCATGAT
GCCGAACAAGGCAAGGGACGCGAGGCCGACTGTGGCATCGAGGTCTTCGTCCATGGCGAG

CGGCCATTAGCGCACGCGGGCGGAAGAGTTTTGGGGAAAGTGGAAGGAAATGGTGGAGGC
TTTGCCACCGACTAATGGCCACGGAAGTAGTTTGCATGCGGCGCGCACGTCCATCTCGTG
TCCGCACCGACGTAAATCAGACTCAAAAATGGATCGGAAATGAATCGTGAGGATGATAAA
AAATGGATGCGCGTCCGTTTGAATTAACGTGTTTGGTCGATTTTTCTGTCCGCCGCGATC
CAAACGAATGCGCTCAGACGAAATAGGGTCGGCCCTTTGGAGTTGCTTTTAGGCCAACTC
CACCGCGCNNNNNNNNNNNNNNNNNNNNNNNNNNNNNNNNNNNNNNNNNNNNNNNN
NNNNNNNNNNNNNNNNNNNNNNNNNNNNNNNNNNNNNNNNNNNNNNNNNNNNNNNNNNNN
NNNNNNNNNNNNNNNNNNNNNNNNNNNNNNNNNNNNNNNNNNNNNNNNNNNNNNNNNNNN
NNNNNNNNNNNNNNNNNNNNNNNNNNNNNNNNNNNNNNNNNNNNNNNNNNNNNNNNNNNN
NNNNNNNNNNNNNNNNNNNNNNNNNNNNNNNNNNNNNNNNNNNNNNNNNNNNNNNNNNNN
NNNNNNNNNNNNNNNNNNNNNNNNNNNNNNNNNNNNNNNNGCCAACTCCACCGCGCGATC
CGAAAGGGACGTCCGGTTTTTATCCGTTTGGGGTCGATTTAGATGGACGTACGTGGGGTG
GACAGACAATTTACGTATCTGCGCCCAACACGCTCGAACAACCGTGACCCTAAACGGTCC
GCCCAGCTCGCCATGCCTCGCCGCCGCACTCGTCCCGTCCGCCCAACTCGTGTCGCCTCC
CCGCGCAACTATGCCCGCCTGTGCCACAGCTTGCCCCGCCTCGNNNNNNNNNNNNNNNNN
>HORVU6Hr1G030490.2 genomic sequence
TGACAAAAATTACAAGTTAACCCATTGTTTTCATTATATTTATGAAATACATATATATTT
GTATATAAAAAAGCATACACAAAATATGATACATACTATCAAAAAAATAATATNNATATA
TATATATATATACATATACATATAATATATCTATGGGAACNTATATATATCATACGAGAA
ATTTATGTTATATATGCATCAACATGTATAGTATGTAGTAGCGTAAAATATGTTTGAAAT
GAAAAAAATGAATGTAAAACACAAAATGAAAAAAAAAAAAAACTATAAACTTTAAGCCCC
AAACAAAAACAATACAAAATCTATAGTCCCGGATGATGACACCAACCGGGATTAACAATT
CTTCAGCCATCGTCACATGTCTAGAGTCACATTACGGGGCTTTAGTCCCAAGGTGTGGCT
AATAAAAACTAAAAGTCAAATTCTTTAGTCTTCACTCTTTAGTCCCGGTTGACGATCCGG
GACTGAAGGCCGTTTTTAACGGAGACTAAAGATTCGTCCTCCACTAGTATGGAACATGGA
GGGAGACAAACACCCCATAAACACACCTTTCTCTAGATCTCTCTCTCTTTCCTCCGCCAC
CTTTTTAGCCTCAAGGCACGCACAAGCACAACCCTTCTGTGCTGCAAAGATACATACACA
CCCGGCTTCTTGCTTCCTCACACATCAGTCAGGCCGAGCGTCACCCACCTTTCTCATCCG
CGCGCGGGACAGCCGGCCAATGATGAGCAGCAGGCTTAACGTCGGCACGATGGCACCGGT
TAGCGACGTGGTCGACTTCGGTTATGCTCCCATACAGTCCTACCCCAACTTTGAGTCGGC
CGGCCTGGCCTTGCCCGGTGACCGGCAGCCACCGTTCCAGCACCATCACCTCTACGATAG
CTTCGACTTCACCTCTGCTAACTTCTCGTTTCAAGACCCGGCCGCGCTCTTCTCCGGCGA
CTCGGCGCTCAGCAACCAGCTCCAGCAGCCCTTCGTCCAGTCGCAGGTCACCATGCCGAC
GACGGCGTCCTCGTCGGTGCTGCAAATGCCGATGATGACGCTGCCGGGCATGCTGATGTC
TTCCTCCTCGTCGCTGGCGGCGGACGCATGCACCTTCGGTGGCGGTGGAGGGTTCCTGAA
GCGGGAGGACGGCGGGCCCTTCTCGGACATCGGCGGTGGCGGGAGGATCGGGCTGAACCT
CGGCCGCAGGACATACTTTTCCCCGGCGGACGTGCTGGCCGTGGACCGCCTGCTGATGCG
CTCCCGCTTCGGCGGAGCTGGCCCAATGGGTATGCTGGGGCTGGGGCTCGGCGCCGCCGC
CCACCACCACCAGCCCCCGCGGTGCCAGGCCGAGGGCTGCAAGACTGATCTCTCCGCTGC
CAAGCACTACCACCGCCGCCACAAGGTCTGCGAGTACCACGCCAAGGCCGCCACCGTCGC
AGCCTCCGGCAAGCAGCAGCGCTTCTGCCAACAATGCAGCCGGTACGTACGTTTATCCTC
TGCACAATTTTCCACTTCATGCACACACCGGGATGGATGGGTCATGGATGCATGCAAAGT
TAGCTGGGAGACGAAATAACCAGCAGTAGGAAGGCTGCGAGCTTTCCGTCGGTAACACCT
ATATGCTGTGATCATATCAAGTCAGCATGTCATATATCAGAAATGAGGTCTTTCAAAAAA
AAAATATCAGAAATGACGCACGTCAGATCAGATCGTTTTTCTCTGAGCGTATCGGCATCT
GCATCACATTATCCTGTTTACTTAGGTAGCAGCACCCCCATCATTGCATTGCATTGCATT
CCAATAGAATTCGACCAGATAGCTAAACCTACTCTGTGCAGATACATGGCGTGTATATAC
ATACATCTGTACGCATGTGTGTGCATGTATTGTGTGAATAATTGTTTTCTTTTCTGGGTA
CATTTCTTGGAATAGGTTTCATGTGCTCGCTGAGTTTGACGAGGCCAAGAGGAGCTGCCG
GAAGCGGCTCACAGAGCACAACCGGCGCCGCAGAAAGCCCGCCGGCGCGCAGGCAGGCAA
GGACTCGCCGGCGCCCCCTACCAAGAAGCCAGACGGTAGCATCACCAGCTCATACACCGG
TGATCACAGGAGTAAGCCCACTCCGCTGATACATGTCTAGATCTAAACACTGGATATATG

CTTATTATTCCCTGTTTTTGAAGCTTGATTAGTTCAAGTTGACCCAGAAAGCTAGCTAGC
TAGAAACACCCTCAGTCGTTGTTAGGGCTTAGCGAGTATTATACGATGTAAACTAAGTAA
AGAACAACAATTCCTCAAAAAAATGTAAAGAACAACAACATAGTGTAATTCTCAATTGTC
TGAAATTTTCGTAAATATATTTATATGTGTCTCTTGAAATGGCATCACTAAAAGCTAACC
TAATAAAATAATATAGTACTACTACTAGTCTCTCCATTCCTAAATATAAGTCTTTTTAGA
AGATTTCATGAGAAAACTACATTCAGAGCAAAATAAATAAATCTACACTCTAAATATGTC
TACATACATCTGTATATAGTCCCTTAATGAAGAGAATGGCGTTTTGGCTCCCGGGAGCCA
TGGAGGCCGAAATGTTTGAAGAATTTAAAATTCGAAAATTTTAGTTCCAAAAAATCTGAA
AAAAATATACAATAAAACAAGGATGTGATTCGTGTGTGTGTGTAAAATTTCAGGTCGAAA
TACTTTGAAACGCGATCTGTATAAAAAAGACAAATTCATGGTCTGAAATGATGAATAGTA
ACATGTGTTAAAAGGTTTCAGATTTATCTTTTTTGCACAGCCCTTATTTCAACGTATTTT
GTTCTGAAAATTTAAACACTTGTGTATTATACCTTCATCTATCTATTTATTTATTTTCAG
AATTTTTTGAAATATTAAAATACGAATTTTCATTGAATTTGAAGTTTGAATTTAAAGGCC
TCCAGTGAGCTCGGGAGCCAAAAGCAATATTCGCTTAATGAAACATTAAAAAGACTTATA
TTTAGGAACGGAGGGAGTAGTATCTAACTCAGTAACTGAAGCAACATCTAACTGTAGTTT
TGAGATTCTTGGAGATATGAAGTGACGTGTGTACAGTAGTCGTTGTACGTTGTCCTAAAC
GGTTACAAGTGTGACTGAACTTCTAGCACTAGATGAAATCATGTCTTGTTTATACAAAAC
CCCCAAAAGATTACGGGAAAAACAGTGCACTACGCTCTAGTCCAGGCCGGTAATTCTATG
CGCGCGCCAGCAAGCACGTTACAACGTTCGACTAGATAGAAACTGGTCGTACACGACATG
ACACACCTAGATGCTACTTCTAGTAGGTTTTGGTCGGCCGATCTGACGTACGTACCTACT
ATTATATTGCTCAGCTCAATTAAACATGATGTATACGTATGGCGCCAAAACATCTAACAT
ACGTAGGTGTAGTACTATCGTACTATACACTTGATGGCTGCTACATACATGTGGCCGTCA
AATCTTGCAGTCTTGTCTGACGTCGCTAGGTGTATATGCTCAGCGAACAAGTCGACGACG
GCGGCGGCCTTCTCGCCGAGCGCCAGCAGCTTCAGCTGCTTTCAGCAGCAGCAGCATGCG
CTCGACAACGGCGGCCAGTCGAACAACGCCACGCCAACGTCGGCGCCTCCGACGCTGTCC
CTGGCGGCGCCACCATCGCCGCCTCAGCAGAACGACGGCGACTTCGGCTCCGGCCTTGAC
ACCATGCTGCAGATGCAGCCGCAAGAGGCCGATGAGCAGGGGGAACAGCAGCAGCATCTC
ATGATGACCTCGCTCGTGCAGTCGCAGCGGCAGCAGCAGCACCGCGACAGCGGGAACATC
TTATCTTGCTCAACGACGTCGGCGTCGGATCAGCGTCACCACCACAACAACGGCGGCAGC
TGCTGCAACGACAACAGCATGCAGCATTTCTTCGAGGTGGACTTCATGTAGTGCGACCCG
TGCATGCATTGCATGAATGAACCTAGCATAGCTGTGCCTAGAAGTGGTTTCACTGCATTA
TCGCGAGGGGTGTGTGCATGAAGCCATGAACTGAAGAAAGAACAAGCTGCTTGCATGCAC
TAGGCTGTGCACTGCATAGAGTATAGGTGTGTGTAGGCGTGCAGCCTTTTTCTTTTCAGA
TTTAATTTCCTGACTTTTTCTGCTTACTGTGTCATTTGCTTGTGGTACATTCCTCAGTGG
TACCTTCTTACAGGGGGAATAGTGTATGTACTCCGGTATGCTTTTATATTTGGTATAATG
TAATTATCTTAGTATTCATGCTAACACTATGATCCGTTTGGTTGGAGACTAATTTTGCCA
AGCCAAAGTGTGGCAAGCCATCAAAGTGTGGTTGAAAAAACGAATGTCAAACTTTGGTAA
AAGTTGGCAAAATTTTTAACTCTATGACAAATGGGCCATAGGGACAATAAAGTGTGGCAA
GCCAAAGTATGGCAAAAACCAAACATTTGCCAACTAAACTGTAGCTGCCAAATTTTGGCT
TGGCAAAGTGTGGCTGGAAACCAAACAGGCCCTATATATATGTTAGCCTCCTGTTCTTCC
ATAAGCCATGTTTTGTAGTAGTATTTTATTTAAGAGAGGACTCTATTGTTTACCCGGTAG
TTGTATGTTTTGTGACATATAATACTCCATATATAATGGAACTTCTGTTGAAATATCCGA
TTTTAGAGACTTTTAACACTGTTTTATTCCAGTTTTGCATTTTGGTTGTTTATGTTAGAT
AGAGTAGGCATGTTGCGTCAATGATTCATAAAAAAAATATGTAAAGAACGAAGGACATCA
TTGACAATCATGTCAAGTCTTCAGTCTTCTCTTGTCGATCTGTTCCATTCCTCAT
>HORVU6Hr1G019700.3 genomic sequence
TGTATCCGGAAGAGGACACTGTGGGATTATGTATGACGTAAGTAGGTGTTCAGGATCATT
CATTTGATCATTATTAGTTCACGTTCGTTTATGTGTAGCTCATCTCCCTCTTATTCTTGT
ACTCGTAAGTTAGCCACCTCATATAAATGCCTAGTCGCTTGCTGCAGCCTCACCACTTAA
CCATACCTCACCCGTTAAGCTTTGCTAGTCTTGATACCTTTGGAAATGAGATTGATGAGT
CCCTGTGGCTCACGGATTACTACAACAACAGTTGCAGGTATAGGTAAAGCGATACTTTGA
CGTGAGCACGATGATTGTTCTATTTGGAGTTTCTTTTTCTTCCTCTTCATCGATCTAGGA

TGGGTTCCAGGCCGGCAGGCTGGGATAACAAGGATGGACGTCGTTCTTTTATCGTTTGTT
TTTGTCCGTAATCGGACCCTGCTCTTCTATATGATAACTGTATATTGTTGTATTAATGTG
ATGTTGATGTAGCTTGTGGCGAGTGTAAGCCAACTCCTTATGCTCATCTTTTTAGTGCAT
GTACTTGTAACGATATCCATTCTTGCGAAATGACGAGATGCGTTTCTATCCCTATTGAGG
CCTCGCGCCAAAATAAAGATAGGATCGCATCTTAGACGTTACAAGTTGGTATCAGAGCAG
TACCGACCAGGAGCCCCTTGATTGATCGAACTTGGCCGAGTCGAGTCTAGTGAAAAACTG
CTTTGAGTCTAGTTATATATCGGAGAGTAGGATTCTTTTTTATCCTCTTCTATGCTCTGG
TGAGGAATCTTGTCGTAATAATTTTAATTCTACTCCTCTTCTCACTCAAAAATATTTTAG
GATCACGCGGATATTTTTGGAATCTCTATGATGTCGATGTGAGGGAGTTTTGGTGGTGCC
TCCTGTCTGACTTGATTTCTTCCGGGGAGTTGAGCTCCAGGGGATTCTTGAGTACATCGT
TATCATTCAGATTTAAAGGTTAGCCTAATACACAATTTTAGGTACTAATACAATTACAAA
ACAAAGGAAATTGCAAGTACTCGGTACCCCAACTCACATTAGTCTTAACAAAACACAAGA
ATTAAGTTTAATTTTTTTAGTTCACAGTAAACAATTGTATTTAAGAATAAACAATTTAAG
TTAACAAATTCTATTAATCAATAGAATTTCAGTTAACCTAAGCCCGACCCTATGCCTAAT
GCACAATTTAAGTTAATACAACGCTGGTTCCTCAAGCCTAATTGGAAGAACAAAGCATAA
TCCCCAATTTAAAAGCAGAGGAGGAAGGAAGATTAGGAACACTTAGAGTAAGCCGTAGCC
GTCGATTCCATTCGTCCAATCTTGCATGCAGCTAGGGTTCGCCACCCAAATCTATGCCAT
CCGCAACCGCTGCTCTACCGACAACGTTGACTCCGAAAAATTATCGTCGATGTTCGTGTA
CGCTGAGCTGGAGTCATCATTGCCATGGCCGTCGATTCCCCCTGACGCACCACACCCTGA
GCATGGGAGGTCTCCGCCGCCATCGTCCCCCTCACCGACGCTCGGGTGAGCTAGGGTTCG
AGGGGAGAGGTGCGAGGTGGGCTGGACGAACCGGTTTGATCGAACCTGGCTAAGAGCGAC
CAAACCGGACTCGCCCTCGTCAGTGTGCGAGCACGTGCCGATTGGCCAACGTGGCACCTG
CCGGGCGGGCCTAACCGGTTAAAACCAGTGTGAATACGTATAAACATTCAGTTTAAGCCT
TTTTGCAGCGGCTAGCCCGAGAGTTGGTACCGACACGTGAAAATTACCACTCTTGATACC
AATCTAAACGTACTACCCCAATTATGATACCTTCATGCAATTATCTCTCGTAACTTCTAT
AGCAGCTGGAAAAGGGCAGCAATAAATCTTGTAGCGCGCTATTTTTCATTGGTTCAGATG
TGTTGTGCTTTGTCATTCTCTGAGGAGGCGATGATGCATCTGCTGCTGCTGAGGACCTCG
TAGTTCGGTGCTGCTTTTCCACGGAAGATTTTGCTGTACAGTAGTAGATGCATATAAGTA
TCACTACTAGTAGTAGAAGTAGTAACAGTAAACATGTGGTGAGATGAGTGCGTTAAGCCT
AGCTGTCTCTCTTGACAACTGAGCCCTCTTGGGGATGGTGTCAGGGAGGGCATGTGCCTG
GTCGTTGCTGTGTGTGTGAGGATGCAGTACAGTAGCAGCAGAGGCATCCAAATCCATCCC
CACTTGACACTTTCTGTTCATTCCAGGCCTACTCAGGTCAGGTGGGGGCATCATCTCGTT
CATCAGGGCATGGCATGAGAAAAAGTTAGCTCATTTGTGTGTGTGTGGATTATTGTGGGT
CTTGTCTTTCTTATCTGGTGGTAGTAGTAGCCCTCCTTTGATTGATTTTGTGAGGCCCTC
ATCCTCCCAAGTGCCTGCCAGGCAGAGGGAGGGATACACTAGAGACTACACCAAGGAGAC
AAAGATTCTCCAGCAAAAAGGAGGAGGCGGCAGTAAGATTCTTCATCGATCTCCCTTTCC
ATTGCATTGCATTGCATACAGTAGGCTGTACACCATGGGGCGGCATGAGCAGTTCTTTCC
CTTCCCTCTAGACGGGTAGATAGAAAGTGTGTGTGCCCCTGCCCCCACAGAAAAAGAAAG
CAGCTGACTTGATGTGGTATAGGTTCTCCCCCTCCCTGCCCCTCCGCCGTCCCAAAGGGG
TTTGATTTGATCTGCATCTCTGTGTGTGTGGAGGGTGGCGCAGAGGCAGCGTGGTGCGTG
GAGGAAATAATGCAGCCCTGAGGTGAGGTGAGGTGAGAGGTGGTACCACTGGCTCTTAGG
GTGGTGGTGAAAGATCTTGATTGGGGCGAGTGAAGCCGGGGTGGTTGTCATGATGCGCCG
TAGGGTCCAGCGCCGGGGTGGTTGCTGATGCCGTGATTGGGTTTGGTTTTCCTTTTCTTT
CAAGCTGATATTAGATCCCATCTAGAGGTGAGATTTGAGGGGGGGAGGCAAACCTTGCGCC
GAGAGGGCCTGAGGCAGAGCTTCTTCTCTCCGGTTGATGGGTGCGGCACCACGGCTCGCC
CGGGTCGAGGGGGAGCTCCGTGGTTGTTCTCCGGCGTGCTGCTGATCGGGGAGGGAGCTG
CTCCGGGCTTCCCGGAGGAGGAGGAGCAGGAATTCTGCTGGCTTGCCTGTTATTCTGGGG
GAGATACAACAACTTGATCTTTGGTCCCTCCAGGTACTTGCGTGTTCTCCTTTTTTGTCT
CTCTTGCTGCTGGGAAATGGAATCATGAATTGATCATCTTCTTCTTGCCATAACTGACGA
TTCCACTTGTGAAGAAAGGTACTACTCTGCATGCTCTCTGCTGCGCAGTGGTACACGGCA
TTCATTCATTCGTTTGATGATAAGTAGATACTACCAGCAGTGCATCATCTTTGTGTACTT
CCTCTCTCCTGCCCTCGGTGCTGCTCATGTGTGTTCTAAGCTGTTCATCATCACTGATTT

TTCCAAGTGCAAAAAAAATTACTCCTGATTTTTCAATCTATCTAATTTTGCTAACACCTC
TTTTCTTCCATTTTGTTGCTTCAGTGTCAGCAGCTTTGACACACAAGTAGGAGGAGGAGC
GGCGCCGGAGATCTTGGGCTCTGGCATGGGCTCGTTTGGGATGGAGTGGAACCAGAGGAG
CTCCGTGCTGTGGGACTGGGAGAATTTCCCGCCGATAGGCGGCGACAACCCCAAGAATGC
GATGCAGGCCGAGCCAAGATTCGCTGCCGTTGCGGCTGCCATGGGGAATGAACCGCTCAA
TTCTTCTGGCGGCAGCGGCACCTTCTCTTCCAGCTCGGAGATGGGGTATGGCTCCTCCAA
GAGCTCCATATCCGCGTCGATCGATTCTTCGTCCAAGGCTGGGAGCAACATGGAGTTCAG
ATTTGCGCCCGTCAAAAACCCTGACAGGAACACGAGCGAAAACACTGAGTTGGGTAAAGT
TGACAACACGAGGACTGGAACGTCTCCGTCGTCTGTGGTGGCAGTGAGCAGTGGAGAGCC
GGTGATCGGCCTGAAGCTCGGCAAGAGAACCTACTTTGAGGATGTCTGTGGAGGGCAGAG
TGTCAAGAGCTCGCCATCGGGTGCTGCGAGTGTGCCGAACAAATCTCCTGCTTTGAGCAA
GAAGGCAAAGGCGGAACAACAGAAGGCACATAACTCATACTGTCAGGTTGAAGGCTGCAA
GGTTGATCTCTCTTCTGTTAAAGAGTACCATCGAAAGCATAGAGTATGTGAACTTCACTC
TAAGGCTCCCAAAGTTGTTGTCGCTGGTCTGGAGCGGCGCTTTTGCCAACAGTGCAGCCG
GTGAGATGCTTTAGATTGCAAATAGTGGCATCCTGTAGGACAGAAAAAAAACCGTTGTTC
TGATCTGTTTCTTTCCCTTCTTTGATTTTCCGCTTCTTTCTAGGTTTCATGCTTTGTCTG
AGTTTGACCAGAAAAAGCGAAGCTGCCGTAGGCGTCTCAATGATCATAATTCCCGCAGGC
GGAAGCCACAGCCAGAAGCAATTTCCTTCAGTTCGTCAAGGATGTCTACGATGTTTTATG
GTAGGGTACTAAGCTTGTATCTGCCTTATGTTTTGTGAAATTCATGCATTAAGTAAGATG
CTTACATATGTCCTTTTAAAACATCCACGTATGCTCACACCAACTCATGGGCACAAAAAA
CTACTAGGGCAGAGCATTTTTTTGTGTGGGAAATGGTCAATTTCTTTGTTGGTTCAAATT
TCCTCACTAATCATAATGTAAAAAATTAGTCACGATTTTCGTAAGTGTGATATCGGAAAG
GCTCTTTGTGATCCTGGTGAATCCCATTTGTGAGGGTTTCTGTTGATAGGAACAATTTAT
GATGATTCCTGTTGATGGGAATTAATGGATAACAATTATAGTTGTCCTTATTCTAAGGAG
TTGTGCCCAATGTACTATTCATTTTACTTTATACTATTACTCAAAACATTTTTGTACTTT
TGTCTGTTAATAATTATGTTATCGTGTCAATGGTATAGTGATTCAGAAAAGAAAAAACAA
ATGCATAATTGTACAAACAAATTGAGGATTTGGAGTTATGGAAGCAGTTACATTTCTGAC
TTATGGACATTTTGTGCTAAAACTCTCGTAATGACTTGAACAGATGCAAGGCAACAGCCA
AATTTCCTATTCGGTCAGGCTCCATATGTTCAAATGAGAAGCTGTGGAAGTTCTTCATGG
GATGAACCAGGAGGCTTCAAAGTTACACACACAAAAGCTCCTTGGTTAAAGCCAACAACT
GCTGCAGGTGTTGATGGGATGCATTTATCTAGTCAGCAGATGTCGGACAATGTTATGCCG
CATGGTGCACATCATGGTTTCGATGGGTTCATGGCATTCAAGGGAACTTCTACAAAGTTC
CCTAATCAAGGTATTTCCCCACTTATGAATGAATTTCTGATTAGACGGAGAACAGTGGCC
ATATAAAGGGTCATCACTTAAATGTAAACTTTTCTTCCTAAAATTCCTAACTAAAACAAG
CATAGATAAATGCACCACTTCGTGTTTTTTTCACGCACCACTTCATAGAATCATACCTTT
TGGATGTTTCTTCTACGAGTAATTTTCAACTGTTTGTCAGTTGTTCTTTTGAGTTAACAT
AACTGACACGAAGCTTTCTGCACTTTCTGAACATATAGCTGTATCATTTCAACATTTCAT
TAGGTAAGTTTAGTTTGCAACATGCGACTATACACATGGTCCTTATTTTTGTGATCCGGG
GACCTGATTGCTATGTCACCTACCCTTATTTTAGCAACCTTTGGTTAATCACACCCTTTT
ACTGAATTTGCTTCTTCGTAAATGATTGTACTTCCTGTCCTACAGTTATTCACAATCTTT
GACTGATCCACCTTAGAAGAGCTGTCAGGCTCTCAACATCTTCTTAGTACTGATTTGATT
CTGACATTTACCAGAAACTGTGATTAGTACCATTTGAGACTATAGCTTTGGGTCCGGAAG
AGACCAGGAAGAGACCAGGCGGTGGATTACTTTATTTGTCATAATCTGGCATGATTGAGT
TGCTCAGCACAAATACTTAGTTAATAGTTTTTTTTTCATGTGGAAAGTCGTTGATAATGA
TGCACATGCAGGCAAGCTTTGGGGTCAGAATAAATAAATAAAACAGTAGTCTGTTATAGA
ATCATGAAAATTTTGGTTCCATCTATGCTAGGATTATCCTTACTACTCCCTCCGTTCCAA
AATAATTAAAGTCATAGGATTTTCCTAAGTCAAACCTTTTAAAGTTTGACCAAGTCTATA
GAAAAAATCACAAATATATATAATGTGAAATATATTTCATAATGAATCTAATTAAGTAAT
TTTAGTTTTGTAAATGTTGATTTTGTTTTTATATAAACTTGGTCAAACTCAATGAAGTTT
GACTTGGGACAATCCTAGAACTTCAATTATTTTGCACGGTGGTAGTATTAACTACTCCCT
CCGTCTCAAAATAAGTGTCTTAACTTTGTACTAGCTCTAGTACTTGTATTAAGCTTAAGA
CACTTATTTTGGGACGGAGGGAATATTAAGTAATGAGAACTTCAGAAAGGATTGCTAGTC

AAAAATAAATAAATTCAGAACGGATTGAGATTTTACATTGCGCATTAACCCTGGATATGT
GGCGATTATATTCTTAAGTATGTTTAGGCACTCCAAAGAATCTTTTCCTTGGTGATGGAC
ATTAACACTAAAAGTTCTGCTTGTTTACCGTTTCCAGGTGTCCAAGCTTCCGCTGTCGCC
CCCAACTCCAGTGGCGCCCCGGATCTTCAGCATGCTCTCTCTCTTCTGTCAAGCAACCCA
GTGGGTGCTGCCAACCTCCAGCCAACTTCCCAGATGCACTCTGGGGTGGCTGCCATTGCC
GGCACCCCCAACCCCGCGATGCACGTGCTGGGATCATCGGCAGGGCTCTGGCTAGACGGC
GGGCCACCCCTAGATGATCACCCGCGGTTCCAGGTCTTCGAGCGCTTGGGGGACCACGAC
AGCGAGCTCCAGCTCCCAAAGCCTTCCTACGACCACGCCTCGCACTTCGACCGGATGCAC
TGATGACGACGGCGCTCCGATCCGGTCACCCCGCTCGGTGCGTGATTTTATCACGCGATC
GACGCCGTTTGTTCGGTCTGAAACCCGGGATCCTGCATTTTCTTTGCTTGGTGCCGTTGC
CTGCCTAGAGGCTTATGGTTTGGCAATGCCTAGAATAAAGGCGCACACGAACAGACTTAG
GATCAAATTGACCCGGTTGTGTTGCCATGGAAGAAGATCTTGATCCATCATTTAGCCACT
TGTCTGAATAATTCTGAACTTGCTATGTTTCTGAATTGCTTCTGCAACTGTAAGTGATCA
CAGTTTTAAATTCTTGATGATGGCGTAGCGGTGACCCATGGCACGGTGCCAGCCATTGCA
AGAGGACCGGCGATGGCTGGGACAGAGATCCAGTGACCACGGCGTAACATGGCCAGGCAC
TGCACATCGTCCCCGTAGCGCCGGGCACTGTTCATGCACTGTCATGTGGGTACCGTTCCT
GTCGATCCGTCCGCTGATCTTAAATTGGACGAGGGGGGCCATGTCAGCATTGCTGTCCCT
AAAGCCTAAGGCATTTGGTCTAAAAACATGAGCACATGATGACATTTTTCGNNNNNNNNN
NNNNNNNNNNNNNNNNNNNNNNNNNNNNNNNNNNNNNNNNNNNNNNNNNNNNNNNNNNNN
NNNNNNNNNNNNNNNNNNNNNNNNNNNNNNNNNNNNNNNNNNNNNNNNNNNNNNNNNNNN
NNNNNNNNNNNNNNNNNNNNNNNNNNNNNGGGGGGGGGGGGGGGAACATCCTCCTAAGAGG
CCAGTACAGTGGTGTTACAATGAAAAATGGGGGAGAACATCCTCCTAAGAGGCCAGTACA
GTGGTGTTACAAGTGAATCAATGGACGTCCCAGGAGGATGGCAGGGTGACTCTAAGCCCT
TTGGCTCCTGCCTTAGCCCAATTCGTGGACTCGTCCTTGATCGCAGCGTCTAGCTCGACT
AAGGATGGGGTTACATGGTTGAATACACACACGTTTCTATGCTTTCAGATCATCCAAGGA
ACCAGGAGCGCTACGGAGGCCAACGCTTTGCGGAGCAGGGGGCGTAGCATCGCGAGCCCG
CAGCCACCAGTCGTGGAGCGTGGCATCCTGGTCAGGCACCGGAGTCGGGAGATGCAGCCA
GGACATGATTCCGTGCCAGGTCTGCTTCGCGAATGGGCATGCGAGCAGCG
>HORVU2Hr1G048280.2 genomic sequence
ACAAAATATGATACATACTACCAAAAAAATAGTATATATACTACCTAGTACACATACATA
CTCTTCGATTTGATAGATAGTACATACAACATACAAAACGCAAGTGATGGTAACTACCAC
CGGTGGTAGATATGAAAAAATACATCCTACCATCCAGGGATANATATATAGGTATAGATA
TAGATATAGATAAATATTTGACACACTTATATATCTTTATTTATATGATAATATATGTCT
CATTTATAGTATAAAGATTAAATTATAAATCATGTAAAGACTGACATGATAAAACTAAAA
ATATATCAACATATTATTAAGCTTGACACCAACGTCCTTGCCTCCAACACAGCCGCTGCA
GCAACAACAAAAATATTAAATCACTTTATCACCCGAGCATAATGCGGCTCCGTCACTTAG
CTTGACACGCTTGTATACCTGGGTTTAACACATAAGGCATTATGTTTAGGTAAACGCTTT
CACCCGGTGCGCCGGCCCTTATGTTTACCCATGCTTACGTACATCGTATTCTGACGTATC
CAAAACCGTATTCTGTACAACTAGCGCTCCCACATCAACATGTGCACCCTGCCTCCACAC
ATACGGCACCCGCCTTTATATCCCTTTCGACCTTTCCCTTCCTTCCACCCCCTCCGCTTC
TTCTCCTTCTCCTTCCACTCATCAGCTAGCTCCACAGTTCCACTTCTGCGACAGCTCCCC
GAGAAGAGCCCTAGCTAGCTTCGTCTTCATCGCGCCTCACCGGAAGGCATGGACCGCAAG
GACAAGTCTCGCAAGTCCCCCTCAGCAGCGTCCATGGCAGCGCTCGCCGCGGCAGCTGNN
NNNNNNNNNNNNNNNNNNNNNNNNNNNNNNNNNNNNNNNNNNNNNNNNNNNNNNNNNNNN
NNNNNNNNNNNNNNNNNNNNNGAGAGGTGCGGCGCCGACCTCACCGAGGCGAAGCGGTA
CCACCGCAGGCACAAGGTGTGCGAGGCGCACTCAAAGGCTGCTGTCGTGGTCGTCGCCGG
TCTGCGCCAGCGCTTTTGTCAGCAATGCAGCCGGTAAGGACAAATTCTTCAGAATCACGT
CCGCTTAGGATTTTCTTTGTGATCGGTTCGTTCTTTCTAAAAGTACTCCGTACTCCATGT
ATGAACAACCGGATAGATTATCCATTTGGCTCTTATCTTTCTTGAAACCTTCATTAAAGT
CTTGAAGTGTAAATACTAAGACAATTTCATTTCATTTTATTTAGAACATGCGTGAGGCTA
TACCTCTTGGCTTCATTCTTGATAACCTTCCAATCAGCACCGATAGTCTGTGATAAGAAA
AGAAACGTATTAGTGCAGGTTACTGTTGATTTACGCCGTACACGTAGATAGTAACCCTGT

TTGTATTATCTCCGGCAAGGTGATAAGAAGTGGAATAAGAGAAACCTGCTATGTTTCACA
TCTAGTCCAGAGGATTCTTGCTTGCTTTGTTAGTGCACCATAGACCATAGTGCACATCAT
TGTCCTTTCTTGTTTGCTTGGAAAGGACAGTGGCTCTGTGGCTGTACATATTCTTTATAG
GACACAGACTTTTAGCTCCTGTCGGTGCAGATGATCACAACTTGACTTGGTCCTTCCCAT
TTGCTCAAACCGATGGCCAGGGCAGCTTCTGGCTCCCCAGTTTGGCTGCAAACAGTTTGT
GCCTTCACTGCTAGCTATGTTTGGGTGCTTGATAGAAGATGTGGTCAGTCCTAGATTAAT
TTGTTCACCCCTGGCCCGACCGGTTGCTGCATATTTTCAGATGCTTTATGTTTCTTGTGG
TTAAAAGACATCTTATCTCTGTATTATTATTAGAGATTGGATGTTACCTTGGCATTTTTC
CTGGACGAGTCATTCCAGCGTTTAGATTTTAACGCTTTATAGGCTTCTTGAATTGGTATG
TATCACGTGAAGCAGAGGTATTAACTGTGCTTTTCGGAAAGGAGGGTAAACCAGTGGGAT
TAGGAGTGTAGCTTGAAGTTAATCTAGCAGTAGTGGTACTGGTACTGGTCGACTTGGTCG
CTGGCTCTGTTCATGCTGTCCTAGTGCTGTTTAAGGTATCGACAAAGACATTGCTACAGC
AGTTCTTGATTATTCATAGGTGGCGAATTCAAGCGGAACAAACAAAGCAAATGTGGTCCT
CTCATACAGAAAGCCATCATGCTTTTCGTAACCTGTAATTTGAAGGAAAAAACTGAACCA
GGAACCGTAGAACTATGAATGATGTTCTGACGATTATGTACAAGAGTTTAGAACCACTCC
TGCAGAGGCACACACACACACATGCTCTGTTCTTCAGGTAATCGATCAATGCACCGTTGT
AAATTGCAAGCTAAAAATTGGAGATTAATTATTGGCGTTGTAGGGGAACTTGGGAGACTA
ATAAATACGAGTACTTATGGACTGTTGGTTTTGCCGTCTCTGCTTTTGCATGCACATGCA
TGAGCACGACACCAGATTCCGCCGGTACAGACGCAAAAATAGCCTCGTTTAATTCGCTCC
AGTTACACGGCAAAATTGATCCTAAAAAAAAGTTACACGGCAAAATTTCAATTTTGTATT
GCTGCTTCACTTCACGTGCTGCAAGTAGGAGTAGTTTTCATCTCTTGGTACTTTGTTTGG
TGTTGCTATCAATGTTGTATCTGTTTGTTGTCGTCTCTGTCTGAAAACTGCTACTGCTCT
TGATCGGCAGGTTCCACGAGCTTCTGGAGTTCGACGACCAAAAGCGCAGCTGCCGCCGGC
GCCTGGCCGGGCACAACGAGCGGCGGAGGAAGAGCTCGGCTGAGGCGAACGGNNNNNNNN
NNNNNNNNNNNNNNNNNNNNNNNNNNNNNNNNNNNNNNNNNNNNNNNNNNNNNNNNNNNN
NNNNNNNNNNNNNNNNNNNNNNNNNNNNNNNNNNNNNNNNNNNNNNNNNNNNNNNNNNNN
NNNNNNNNNNNNNNNNNNNNNNNNNNNNNNNNNNNNNNNNNNNNNNNNNNNNNNNNNNNN
NNNNNNNNNNNNNNNNNNNNNNNNNNNNNNNNNNNNNNNNNNNNNNNNNNNNNNNNNNNN
NNNNNNNNNNNNNNNNNNNNNNNNNNNNNNNNNNNNNNNNNNNNNNNNNNNNNNNNNNNN
NNNNNNNNNNNNNNNNNNNNNNNNNNNNNNNNNNNNNNNNNNNNNNNNNNNNNNNNNNNN
NNNNNNNNNNNNNNNNNNNNNNNNNNNNNNNNNNNNNNNNNNNNNNNNNNNNNNNNNNNN
NNNNNNNNNNNNNNNNNNNNNNNNNNNNNNNNNNNNNNNNNNNNNNNNNNNNNNNNNNNN
NNNNNNNNNNNNNNNNNNNNNNNNNNNNNNNNNNNNNNNNNNNNNNNNNNNNNNNNNNNN
NNNNNNNNNNNNNNNNNNNNNNNNNNNNNNNNNNNNNNNNNNNNNNNNNNNNNNNNNNNN
NNNNNNNNNNNNNNNNNNNNNNNNNNNNNNNNNNNNNNNNNNNNNNNNNNNNNNNNNNNN
NNNNNNNNNNNNNNNNNNNNNNNNNNNNNNNNNNNNNNNNNNNNNNNNNNNNNNNNNNNN
NNNNNNNNNNNNNNNNNNNNNNNNNNNNNNNNNNNNNNNNNNNNNNNNNNNNNNNNNNNN
NNNNNNNNNNNNNNNNNNNNNNNNNNNNNNNNNNNNNNNNNNNNNNNNNNNNNNNNNNNN
NNNNNNNNNNNNNNNNNNNNNNNNNNNNNNNNNNNNNNNNNNNNNNNNNNNNNNNNNNNN
NNNNNNNNNNNNNNNNNNNNNNNNNNNNNNNNNNNNNNNNNNNNNNNNNNNNNNNNNNNN
NNNNNNNNNNNNNNNNNNNNNNNNNNNNNNNNNNNNNNNNNNNNNNNNNNNNNNNNNNNN
NNNNNNNNNNNNNNNNNNNNNNNNNNNNNNNNNNNNNNNNNNNNNNNNNNNNNNNNNNNN
NNNNNNNNNNNNNNNNNNNNNNNNNNNNNNNNNNNNNNNNNNNNNNNNNNNNNNNNNNNN
NNNNNNNNNNNNNNNNNNNNNNNNNNNNNNNNNNNNNNNNNNNNNNNNNNNNNNNNNNNN
NNNNNNNNNNNNNNNNNNNNNNNNNNNNNNNNNNNNNNNNNNNNNNNNNNNNNNNNNNNN
NNNNNNNNNNNNNNNNNNNNNNNNNNNNNNNNNNNNNNNNNNNNNNNNNNNNNNNNNNNN
NNNNNNNNNNNNNNNNNNNNNNNNNNNNNNNNNNNNNNNNNNNNNNNNNNNNNNNNNNNN
NNNNNNNNNNNNNNNNNNNNNNNNNNNNNNNNNNNNNNNNNNNNNNNNNNNNNNNNNNNN
NNNNNNNNNNNNNNNNNNNNNNNNNNNNNNNNNNNNNNNNNNNNNNNNNNNNNNNNNNNN
NNNNNNNNNNNNNNNNNNNNNNNNNNNNNNNNNNNNNNNNNNNNNNNNNNNNNNNNNNNN
NNNNNNNNNNNNNNNNNNNNNNNNNNNNNNNNNNNNNNNNNNNNNNNNNNNNNNNNNNNN
NNNNNNNNNNNNNNNNNNNNNNNNNNNNNNNNNNNNNNNNNNNNNNNNNNNNNNNNNNNN

NNNNNNNNNNNNNNNNNNNNNNNNNNNNNNNNNNNNNNNNNNNNNNNNNNNNNNNNNNNNNNNN
NNNNNNNNNNNNNNNNNNNNNNNNNNNNNNNNNNNNNNNNNNNNNNNNNNNNNNNNNNNNNNNN
NNNNNNNNNNNNNNNNNNNNNNNNNNNNNNNNNNNNNNNNNNNNNNNNNNNNNNNNNNNNNNNN
NNNNNNNNNNNNNNNNNNNNNNNNNNNNNNNNNNNNNNNNNNNNNNNNNNNNNNNNNNNNNNNN
NNNNNNNNNNNNNNNNNNNNNNNNNNNNNNNNNNNNNNNNNNNNNNNNNNNNNNNNNNNNNNNN
NNNNNNNNNNNNNNNNNNNNNNNNNNNNNNNNNNNNNNNNNNNNNNNNNNNNNNNNNNNNNNNN
NNNNNNNNNNNNNNNNNNNNNNNNNNNNNNNNNNNNNNNNNNNNNNNNNNNNNNNNNNNNNNNN
NNNNNNNNNNNNNNNNNNNNNNNNNNNNNNNNNNNNNNNNNNNNNNNNNNNNNNNNNNNNNNNN
NNNNNNNNNNNNNNNNNNNNNNNNNNNNNNNNNNNNNNNNNNNNNNNNNNNNNNNNNNNNNNNN
NNNNNNNNNNNNNNNNNNNNNNNNNNNNNNNNNNNNNNNNNNNNNNNNNNNNNNNNNNNNNNNN
NNNNNNNNNNNNNNNNNNNNNNNNNNNNNNNNNNNNNNNNNNNNNNNNNNNNNNNNNNNNNNNN
NNNNNNNNNNNNNNNNNNNNNNNNNNNNNNNNNNNNNNNNNNNNNNNNNNNNNNNNNNNNNNNN
NNNNNNNNNNNNNNNNNNNNNNNNNNNNNNNNNNNNNNNNNNNNNNNNNNNNNNNNNNNNNNNN
NNNNNNNNNNNNNNNNNNNNNNNNNNNNNNNNNNNNNNNNNNNNNNNNNNNNNNNNNNNNNNNN
NNNNNNNNNNNNNNNNNNNNNNNNNNNNNNNNNNNNNNNNNNNNNNNNNNNNNNNNNNNNNNNN
NNNNNNNNNNNNNNNNNNNNNNNNNNNNNNNNNNNNNNNNNNNNNNNNNNNNNNNNNNNNNNNN
NNNNNNNNNNNNNNNNNNNNNNNNNNNNNNNNNNNNNNNNNNNNNNNNNNNNNNNNNNNNNNNN
NNNNNNNNNNNNNNNNNNNNNNNNNNNNNNNNNNNNNNNNNNNNNNNNNNNNNNNNNNNNNNNN
NNNNNNNNNNNNNNNNNNNNNNNNNNNNNNNNNNNNNNNNNNNNNNNNNNNNNNNNNNNNNNNN
NNNNNNNNNNNNNNNNNNNNNNNNNNNNNNNNNNNNNNNNNNNNNNNNNNNNNNNNNNNNNNNN
NNNNNNNNNNNNNNNNNNNNNNNNNNNNNNNNNNNNNNNNNNNNNNNNNNNNNNNNNNNNNNNN
NNNNNNNNNNNNNNNNNNNNNNNNNNNNNNNNNNNNNNNNNNNNNNNNNNNNNNNNNNNNNNNN
NNNNNNNNNNNNNNNNNNNNNNNNNNNNNNNNNNNNNNNNNNNNNNNNNNNNNNNNNNNNNNNN
NNNNNNNNNNNNNNNNNNNNNNNNNNNNNNNNNNNNNNNNNNNNNNNNNNNNNNNNNNNNNNNN
NNNNNNNNNNNNNNNNNNNNNNNNNNNNNNNNNNNNNNNNNNNNNNNNNNNNNNNNNNNNNNNN
NNNNNNNNNNNNNNNNNNNNNNNNNNNNNNNNNNNNNNNNNNNNNNNNNNNNNNNNNNNNNNNN
NNNNNNNNNNNNNNNNNNNNNNNNNNNNNNNNNNNNNNNNNNNNNNNNNNNNNNNNNNNNNNNN
NNNNNNNNNNNNNNNNNNNNNNNNNNNNNNNNNNNNNNNNNNNNNNNNNNNNNNNNNNNNNNNN
NNNNNNNNNNNNNNNNNNNNNNNNNNNNNNNNNNNNNNNNNNNNNNNNNNNNNNNNNNNNNNNN
NNNNNNNNNNNNNNNNNNNNNNNNNNNNNNNNNNNNNNNNNNNNNNNNNNNNNNNNNNNNNNNN
NNNNNNNNNNNNNNNNNNNNNNNNNNNNNNNNNNNNNNNNNNNNNNNNNNNNNNNNNNNNNNNN
NNNNNNNNNNNNNNNNNNNNNNNNNNNNNNNNNNNNNNNNNNNNNNNNNNNNNNNNNNNNNNNN
NNNNNNNNNNNNNNNNNNNNNNNNNNNNNNNNNNNNNNNNNNNNNNNNNNNNNNNNNNNNNNNN
NNNNNNNNNNNNNNNNNNNNNNNNNNNNNNNNNNNNNNNNNNNNNNNNNNNNNNNNNNNNNNNN
NNNNNNNNNNNNNNNNNNNNNNNNNNNNNNNNNNNNNNNNNNNNNNNNNNNNNNNNNNNNNNNN
NNNNNNNNNNNNNNNNNNNNNNNNNNNNNNNNNNNNNNNNNNNNNNNNNNNNNNNNNNNNNNNN
NNNNNNNNNNNNNNNNNNNNNNNNNNNNNNNNNNNNNNNNNNNNNNNNNNNNNNNNNNNNNNNN
NNNNNNNNNNNNNNNNNNNNNNNNNNNNNNNNNNNNNNNNNNNNNNNNNNNNNNNNNNNNNNNN
NNNNNNNNNNNNNNNNNNNNNNNNNNNNNNNNNNNNNNNNNNNNNNNNNNNNNNNNNNNNNNNN
NNNNNNNNNNNNNNNNNNNNNNNNNNNNNNNNNNNNNNNNNNNNNNNNNNNNNNNNNNNNNNNN
NNNNNNNNNNNNNNNNNNNNNNNNNNNNNNNNNNNNNNNNNNNNNNNNNNNNNNNNNNNNNNNN
NNNNNNNNNNNNNNNNNNNNNNNNNNNNNNNNNNNNNNNNNNNNNNNNNNNNNNNNNNNNNNNN
NNNNNNNNNNNNNNNNNNNNNNNNNNNNNNNNNNNNNNNNNNNNNNNNNNNNNNNNNNNNNNNN
NNNNNNNNNNNNNNNNNNNNNNNNNNNNNNNNNNNNNNNNNNNNNNNNNNNNNNNNNNNNNNNN
NNNNNNNNNNNNNNNNNNNNNNNNNNNNNNNNNNNNNNNNNNNNNNNNNNNNNNNNNNNNNNNN
NNNNNNNNNNNNNNNNNNNNNNNNNNNNNNNNNNNNNNNNNNNNNNNNNNNNNNNNNNNNNNNN
NNNNNNNNNNNNNNNNNNNNNNNNNNNNNNNNNNNNNNNNNNNNNNNNNNNNNNNNNNNNNNNN
NNNNNNNNNNNNNNNNNNNNNNNNNNNNNNNNNNNNNNNNNNNNNNNNNNNNNNNNNNNNNNNN
NNNNNNNNNNNNNNNNNNNNNNNNNNNNNNNNNNNNNNNNNNNNNNNNNNNNNNNNNNNNNNNN
NNNNNNNNNNNNNNNNNNNNNNNNNNNNNNNNNNNNNNNNNNNNNNNNNNNNNNNNNNNNNNNN
NNNNNNNNNNNNNNNNNNNNNNNNNNNNNNNNNNNNNNNNNNNNNNNNNNNNNNNNNNNNNNNN
NNNNNNNNNNNNNNNNNNNNNNNNNNNNNNNNNNNNNNNNNNNNNNNNNNNNNNNNNNNNNNNN

NNNNNNNNNNNNNNNNNNNNNNNNNNNNNNNNNNNNNNNNNNNNNNNNNNNNNNNNNNNNN
NNNNNNNNNNNNNNNNNNNNNNNNNNNNNNNNNNNNNNNNNNNNNNNNNNNNNNNNNNNNN
NNNNNNNNNNNNNNNNNNNNNNNNNNNNNNNNNNNNNNNNNNNNNNNNNNNNNNNNNNNNN
NNNNNNNNNNNNNNNNNNNNNNNNNNNNNNNNNNNNNNNNNNNNNNNNNNNNNNNNNNNNN
NNNNNNNNNNNNNNNNNNNNNNNNNNNNNNNNNNNNNNNNNNNNNNNNNNNNNNNNNNNNN
NNNNNNNNNNNNNNNNNNNNNNNNNNNNNNNNNNNNNNNNNNNNNNNNNNNNNNNNNNNNN
NNNNNNNNNNNNNNNNNNNNNNNNNNNNNNNNNNNNNNNNNNNNNNNNNNNNNNNNNNNNN
NNNNNNNNNNNNNNNNNNNNNNNNNNNNNNNNNNNNNNNNNNNNNNNNNNNNNNNNNNNNN
NNNNNNNNNNNNNNNNNNNNNNNNNNNNNNNNNNNNNNNNNNNNNNNNNNNNNNNNNNNNN
NNNNNNNNNNNNNNNNNNNNNNNNNNNNNNNNNNNNNNNNNNNNNNNNNNNNNNNNNNNNN
NNNNNNNNNNNNNNNNNNNNNNNNNNNNNNNNNNNNNNNNNNNNNNNNNNNNNNNNNNNNN
NNGAACGGCGGCGACGGGTGCCGCCACGCCGACCAGGACGGCCGTAGCCACCCTGGGAAC
CCGCCGCTGAACCATTTCCAGATCAGATAAACCTACCTAAGCATGCTCCCTCTCTTCTGT
CATGCAGCCATGCTACTAGCTACAAATATCTACATATGTATCCACCCACCTACGTAGGTA
CGTACCTACCTACCTATGTCTCCACTATCCAGTCCCAGTGTACTAAGTGGCAGTATATAC
TGTCTCCTGTACTGCTATATGCTTGCGTCTGTCCGCCAAAAACCTAAGCTAAGCTAATTG
TGACCCGGGAACTAGTCAGGTGTTAGAGTGATTGTCTGTGTGATGTGTAATTGTATGGCC
TGTGCAGCCAAGACTTCTACAGGCTGTGTAATATTGCTGCTCTGCTGGGATAATTTGTGG
CAATATATGCTATCTATTCAAGTAACTACTACGAGTACTAAGGATTACTCACAACTGGCC
AATTTATGTTTTCTTCCAAGTCCCTATACCCGATCTCGTGTTTTTTCTGTTGTGTTTTTT
GTTTGATACGTATTTGGTTTTCACTTGGCCTTCCCCAACCAACCAGACAATGTTAGGTTT
TTGAGCCTGATATTTTCATATAAACAACTCATCGTCTCTGTTCTTCCAACGATTCGGGTC
GTGTGTAGCTACCACCATGGGTAGCAAATTATTTCCGATAAAACTAACCCCGTAAACAGA
ATGATGTTGCACTTATCTCCTTGCTAACACTTCTCTACCTTCTAGAATGGGCACAACATG
CTATGCAGGAATGTCATCCTTTTGCTGAGTTATTCTTCTTTCGAGAAGGTGATCGGGACA
CTTCGGATGGCCGCCTATGTCACTCCTGCAAAAATGACAAATGGGTATCTTCGAATAACC
AAATATACAATTCTTAAGAGTGTAGAAGTGTTAATCAATGTTGTGATAAGGGTTTATGGA
GCTCGTTACCTACGGGCACCCAACGTCGAGGACACTGCGAGGCTTTCAACATTCAGTGAA
GTAAGAAGTGGGCTAGGGATGCTTGGGAGCATTGACTGTATGTGTTGGAGGCGAAAGAAT
TGCAAGGTGGCATGACACAATCAGTTCACAACGCGTTGCTGTGATCCAGCTATTATTTTT
GAAGCAATCGCCTTCGAGAATTTGTGAATTTGGCATTGCAACTTTGTCTTGCTTGATTGT
CACAATGATATTAATGTCTTGCAGAGGTATCTACTTTATGCTAGACCATCATGTGGTGAT
GTTGTGACATGCAATGTCAAGCTCAACAAACATGATTATGACCAATGCTATTATCTAGCC
GATGTAATCTACCCAGCCTAGTCAACATTTGTGAAGACTATATTTCTATTCCTTGGTGTA
AGAAAAAAACAATTCACAAAGTGTCAGGAATCGATGGGTATGGACACTGAGAGGGCAATG
GAGTGTGGCAAGCTTGGTTTGCAATTGTACGGGGTCCTACTCATTTCTCAAACAGGAATA
CCTCGACAAAAATCACTACTTGTTGCATGATTCTGTGCAACATTATCACCGAGGATGAGA
GAGAGAGAGATATTGATGGGCTACGAGTATCACTAGTAGAAAAAGGCCCATTTGTCCCGG
TTCCGGAGGCCCATTAGCCCCGGTTCCGGAACCGGGACTAAAGGGTCGGGACTAAAGGCC
CAAACCTTTAGTGGTTGACTTACGAACCGGGCACAAAGGGTCTCCACGTGGCCGCTGCGT
GGAGCCCAGGCAGGAGGAACTTTATTCCCGGTTGGTCACACCAACCGGGACCAAATGGCA
TCCACGCGTGAGCATCTGGCCGGACCTGGGGTTTTTTTTGAATGGGGGTGGGGGGTTGG
GTGGTTTTTGGAGGGTTAATTTAGTGGTTTCATACTGTGTTAGCAACCTAATAGAGAGGT
GTCTACTACTAGGGAAAAGCCTAGTAGTAGCGCGGGTTTAGTGCC
>HORVU3Hr1G094730.1 genomic sequence
GGAAGCGTCCATGGCGGTGGAGAAGCCCCCGGAGGTCAATCCTCCCTCCGGCAGGGTGCC
GGGAAGAAGTCCCCTAACGCTCCCGATCTTGGAAGCGCTGCGGCGGCGGAACGATGGAGA
AATTCGCGATTCCAGAAAGTCTACGAGGGTTTCCTCACGGAACATTAAATATGGGCCAAA
GGAGGGCACCGGAGGAGGTGTGGACCACCCAGGCGACTTCCTGGCGCGGCCTGGGCACTG
GCCGCGCCAGCAGGCCGCCTGGGAGGTCCCTGCCCCCCTCTGGCCCTCCTTCGGTGATCC
CAAAGCTTCTGTTTCGCTGATTTTTTATATATTTTTCTGGATTTTTTCGGGCTTCGGAAA
ATTGGATAAAAGCCCCCTGCAAAAAAGACATCAGCAAACACACACTGGCATTGGGTGCAC

TGAGTTAGTAGGTTAGTCCAAATATGTGTAAAATGATATAAAAGTGTAGCAAAACACATA
ACATTGTCACCCAANNNNNNNNNNNNNNNNNNNNNNNNNNNNNNNNNNNNNNNNNNNNNN
NNNNNNNNNNNNNNNNNNNNNNNNNNNNNNNNNNNNNNNNNNNNNNNNNNNNNNNTAGGGC
GAATTCTCTGGACACAGAGAGACGTGCGTGCGTGCGCGTGTATTTGGCATGGATTCTTGG
GAGCCCAAGATGCCTTCTTGGGACCTCGGCACGGTGGTCGCGCCCAGCGGCGGCGGGGGA
GGAGGAGGCGGCGGCGGGGCGCTGGACCTGAAGCTCGGCGCGCCGACGAGCTGGAGGCCG
GTCCCGGCGCCGGTTGTATCGGTGCAGCAGCAGCAGCAGCAGCAGCCGCCTTCCGCGGCG
ACGGCGAAGCGTGCCCGGGCGGGGCAGGGGCAGCAGACGGTGCCGGCGTGCTCCGTGGAG
GGGTGCACGGCCGACCTGTCCCGGTGCCGCGAGTACCACCGCCGGCACAAGGTCTGCGAG
GCGCACTCCAAGACGCCCGTCGTCGCCGTCGCCGGCCAGCAGCAGCGCTTCTGCCAGCAG
TGCAGCAGGTACACCCTACACTTCTCTCTCTCTTTTTCCATTTTTTTTTGGTCGTGCATT
CCCTACCACCAACTTGTTCCTTGCCTTATGACATGCATCCATGCTCCGCTTTTGCCACTA
CTAGTAACATCTTTACCGTGTAGTAGAGTAGTAAGTAGCAAGATCCTTCCACGAGTTACC
ACTGTTGCACGAGAGTTGCAGCGAATCGTTTCAACCCCGCCGAAAACCTCGCCGCGGACA
CTGTAGCAGCTGCTGCAGCAGCGGCGGCGGTAGTACGCGATGGCTAACCAACTACGGCGA
CCGTTTTAATCGCAGCAATTCGCTGTCAGCCGGAGCGGAGCAGTTCGCAGTCCCTTGGGC
CATCATCATGGCTCGCTTGGCGCTAGCGACTAATCGTCCGACGTGGACCACACCAAAAAC
CCCTGTTCCTGCCGCCCCCATCTGTTCCCCACTCACCGTAAATTCCACAGCGAAAAAATC
ACTCAAAACTATCTCCAAAAGTTTATTTCCATGCAAAACATTTGCACTGGTACTCCTAC
TCCTACCTGAAAATGAAAGCAGTATCAGAGGAGAAGCAAGAGAATTTAACCGACGTGGTC
GTGGAGACGGATAAAATTCCGGGCGGGTCGGAGTCTGGTCCCGGGCCCGGGCCCGGGCTG
GTGGCCGGATGTGTGTATGAACGAGACAAGCGCACACGCGCCACCATTAAGGTGCCGTGG
CGCAGCCCGACCCGGCCCGTGCACCTGCCCTACAACGACGCTCACGCCGGAACTGGACCT
TTCAGGTCGTCGTTCTCATGGCGTGGCTGCTGCTGCTGCTCAACGGCCGCGTCCCTGTCC
TCCACCGCTCCCTGTCGCCGCCGCCCCTTGTCGCGACGCACGCTTTCCACGTCCGTCCTC
GGTGGTTTCGAAACTGGGAATTCATCCCATATGCCTGTTCTAGAAGGCAGGGATCATGGA
TCTCAACGGTCAACCGTGATTGTGGACGTCTGATTAAGATTCTTGCGTGAAGGTTACGGG
TGTGGTTAAGCTCGTCTGGAGAGGGGCTTGGGAGATTGGGGCGTGTATCTTCATTTCCAT
GTGCTTGATTTCTCCTAGATCTGTGGCATGTGGTTGGAGGAGCATCTGACCGGATCTACT
ATATGACAGGAGCACTCTTTTTTTTTGTGTTAGCATCTGATCAGGATTTGTATAGTAGTT
TTCACGCTGAATTTGTAGTAGTCTATCTTGAGAAATGCTTGTTGCCTGATCAAACTGTTG
TTTGCAGGTTCCATTTGCTCGGGGAGTTCGACGAGGTGAAGAGGAGCTGCAGGAAGCGGC
TCGACGGCCACAACCGGCGCCGCCGGAAGCCGCAGCCGGATCCCCTCAACCCTGCCGGCC
TGTTCGCTAATCACCACGGTACAATCCCTAAAAACATCTCTTGCACCCCGTGGAAATTGG
TCATCCTACCTAGAAAGCACTCTCATTTTGATTTTGTAAGATCCAACCATTCGATGAGCA
AAGTAATCGAGATTTTTCACACTCCCATTTTGTCGGAAACAAGTGGAAAAATGTGATGCA
TTTGATGGATCATGTGATCTTAGCTAGTTTTCTTCTCAGCTTTATCCCCTTTCTGTTTAT
TTAGACTGTTTCCGATTTTCAGCTGACTGCTAGTATTATCCAGAAAAAACACTTCACACG
TGCTTGCTGAGTATAGAGTAGCACAAATTTTTCTTCCTTCCGCAAAAGGATGGGAACGGG
AGGTTTGACTACAGAGGTGCTTAAACTAGCAGTGGTATGAACGTCTTGTTGGTGTAGCAG
ACCAAGATAGGTTTTCAGCCTTTGGCTGGCATTTCCAGGAACTTCACGTGGGATCCAAAG
CAAGAGAATATCTAGGCCTGAAGTTTCTTCTGAACTTTCTGTCATGATACAGCCGTATTC
ATGTGAAAGCCAGTTAATGATGTCACAGTGAATTGAATACCTATGACATGAAACTACTAC
TAGTAGCACTCCTATACCTTTGAAGATTTTTACATGGAATTTACCATGTTACAATGCAAA
AGTAGCAGGGTGAATGCACTGGGAATTTACCGCTTGTCTTTGTAATTTTAGTCTTTTGTT
TTTTTCAATCAGTTGGTCGACATGGCTGTTGTCATGCAGGTTTGCAACACTGACGTCCGA
GCTAACTGTTTTCTCCCATCATTTTCCGTGAACAGGAGTGCCAAGATTTGCGTCGTACCC
GCAGATCTTCTCCCCGACGTCCATGGCGGAGCCCAAGTGGCCAGGCGGCATCGCCGTGAA
GACGGAGGCCGACGCGTTCCACGAGCAGTACTACTCCTTCAACGGCGCCGCCTCCCTCTT
CCACCACGGCAAGCCGGAGAGGAAGCACTTCCCCTTCCTGACCGACGGCGGCGACGCCAC
GTTCGGCTGCCAGCCGCCGGCGTTCACCATCACCCCTTCCTCGGAGAGCAGCAGCAACAG
CAGCCGGCACAGCAACGGCAAGACCACCATGTTCGCCCACGACGGGGGCCCGGACCACAA

CTGTGCTCTCTCTCTTCTGTCAGACAACCCGGCGCCGGCGCACATCATGAACCCCGCGGA
GGCGCATCATCTCGGTGGCGGCGGCGGCGTGACCATACAGTACGGCGGTGGAGGCGGCGG
CAAGGTGGCGCGGCTGCACAACAACGGCGACGTCTCGCTCACCGGGCTGTCTTACGTGAG
CCTGGGTGACAAGGGCGCGCCCATGTTGTTGCACACGTCTAACAGATCACAACACGCCGC
TGCTACCGCTACCACTGCCGTTACTACAAGCACAGCTCCAGCTCCGACGGCGTCGCAGAT
GCAGCAGTACCATGGCTACTACCACCACCAACACCAAGTGGGCGCTGATCAGGGGAACCA
CCAGGATGCTGGTGGGATGCATGCCCTTCCCTTCTCGTCATGGTAGTTGTCTCCCTCATC
AGTCAGATCCTAGGCAGTTGGTGGTGGTCATCAATCATCGAATATGCCCCCTGATAGTGT
TAGCCCTGATGATCCTCAAGAAATCACCCGAGATATGTCCATTTTGCAACTTCTCATGAT
ACTTCTAACTTATGGAAAATCCCTCCTGCTTATGATTCAGAATTGGATTACGACGATTCT
ATTTGTACCGGTTGGTTTTTGTTTCATTTTGGCCGGCGTGCACTTGGTTTGCTAGGGCAG
CATCAGCAGAAAGTAGACAGAAGGATTTATTTAACTGTACTAAACAGTGGAATTAGCATA
AAAGTATTCAGATAATTGATCATTCTAATCACGCGTCTGCCTGTTCCTCACATGTTGTGA
ATCATTAGAGATATTGTTCAGCAACAGTAATTAGATTATCCAGGATTAGGGGCCACTTCC
ATAGCCTGGTTCTAGATAGGCCCATATGCTTTCAGAATTCAGATAGCTACACCATGTTGT
GGAACTGTGCTTGTTTGCAAGTACTCTTTGTCAAAAATTAAACAGTAAAGTACATGTTGA
AGATGTGGCAACTGGGAGTCCACATAATTCGTTTGAAGTCCATGTTCATGGGAGAGTGAT
TGTTTGAGGATCTAGGGGGTATCATGTTGACAAGTAACCAAACAGGCCAACTCTACTACA
GGGCACTCCCTCCGTTCCTAAATATATATAGATTCATTAATGTGCTTCATATATACTTCC
TCCGTAAACTAATATCAAAGCTTTTAGAACACTATTTTAGTAATCTAAACACTCTTATAT
TAGTTTACAGAGGGAGTAGTTGTCAGTGGAATCTCTACAAAGACTTTTTTCGGAGCGGAG
GGAGTACATAAAACTGAAATTTTACCAATGACGCGTGGCGATAATAGTTATCAGTTAAAA
AATTGTGGCTGAACAGATCATATTTG
>HORVU7Hr1G110980.3 genomic sequence
TTTCCCGATTCGTGTGCGAGGCTGAAGGCATGTACGAGCCGAGGAGCGACACCGACGGGC
GTTGGCCATGGCAATTCCGAGCCCTGAGCGAGCGGAAGTTGGCGGGAGGGAGTACAAAAC
TTGTTTGTGCTGGTCGGGAGTGTAGGGGTTGCCGGAGTTGCTGCAGTTTGGAGGAATTTA
GAGGGATGGTTGCGACGGTGAACAACACGGCGATGGGTGGAGGTGGGTGGGGTGGGGGTG
TTGGGNNNNNNNNNNNNNNNNNNNNNNNNNNNNNNNNNNNNNNNNNNNNNNNNNNNNNNN
NNNNNNNNNNNNNNNNNNNNNNNNNNNNNNNNNNNNNNNNNNNNNGATCTAGAGGTAAAA
CCGTGGAGGTATGTGGTCGGGCTGCCGTGGCAAAAGTTGTCTCAAATCAGCCCTAATACC
TCAGTATATATAGGAGGGAGGGGGAGGGAAGAGGCAGCCTCAAACCCTCAAGGTTTGGCC
GAAATTGGAGGTGGAGGAGTCCTACTCCAATCCTACTTGGAGTAGGATTCCACCTTCCCA
CTTGGAAACTCTTTCCACCTTGTGTTTTTTCCTTCTCAAACCTTATGGGCCTTAGTGGGA
ACTTATTCCAGCCCACTAGGGGCTGGTTTATATCTTCCCATAGCCCATGAGACCCCTTGG
GGCGTGACACCCCTCCCGATGGTCCCCGGCACCCCTCCCGGCACTCCCGGTACACTACCG
ATGAGCCCGAAACCTTTCCGGTAATGCACGAAAACCTTCCGGTAACCAAATGAGGTCATC
CTATATATCAATCTTCGTTTCCGGACCATTCCGGAAACCCTCGTGACGTCCGTGATCTCA
TCCGGGACTCCGAACAACATTCGGTAACCAACCATATAACTCAAATACGCATAAAGCAAC
GTCGAACCTTAAGTGTGCAGACCCTGCGGGTTCGAGATCTATGTAGACATGACCCGAGAG
ACTCCTCGGTCAATATCCAATAGCGGGACCTGGATGCCCATATTGGATCCTACATATTCT
ACGAAGATCTTATCGTTTGAACCTCAGTGCCAAGGATTCATATAATCCCGTATGTCATTC
CCTTTGTCCTTCGGTATGTTACTTGCCCGAGATTCGATCGTCAGTATNNNNNNNNNNNNN
NNNNNNNNNNNNNNNNNNNNNNNNNNNNNNNNNNNNNNNNNNNNNNNNNNNNNNNNNNNN
NNNNNNNNNNNNNNNNNNNNNNNNNNNCGGTGGGAGCGACAGGGGCGGCGACGGGGGCGG
CGACACGACGGTGGTGGCGGCGGCAGGTGGTCGTCGGGGATTGGGAGAGAGAGACAGAGG
GGCGGGGTGCCGTTACACAATGTGGGTGTGAGAGAGAGGGGCGGGGTGGGGGGAAAGAGC
CGTTAACATAGTTAGCTCTTTGCCGTTTGCCGGCGGACGGCAAAGAGTCTANNNNNNNNN
NNNNNNNNNNNNNNNNNNNNNNNNNNNNNNNNNNNNNNNNNNNNNNNNNNNNNNNNNNNN
NNNNNNNNNNNNNNNNNNNNNNNNNNNNNNNNNNNNNNNNNNNNNNNNNAAGAGTCTAGA
CTCTTTGCCGTCTGCCTCCGGACTCTTTGTCGTCCGCTAGCAGACGGCAAAGAAGGGACT
CCAGTTTGACCTAACCACCCCGCGGTCAGGTGGACCCATCTACATCTTTGTCGTCCGCTG

TGAGACTCTTTGTCGTCTGCTAGCTGACGGCAAAGATGTGACTGAAGGCAAAAACACTCT
ATGCCATCAGCCAGTGCTTTGCCGTCTGATTTGTTGAAGCTGACGGCAAATACACTCTTT
GCCAGCAGCTAGCACACGGCAAAGATTAGGCAGATGGCAAAAAACTGTTTTCCAGTAGTG
ACGGTCCCAACAGAACACACAAACAATTTTTTGATAATATGTGTCACGTCATCGAGACGC
CAAGGTCCTTGGCGTTAGGTATGACCAAATGCCGGTGTCCTTGACGTCTTGTCCATGACC
AGGCGCTAGGGTCCCTGACGTCTCGGTCTTTGCCACGTCTGCGAGTTAATGGGTCAGCCA
ATCGTGCAGGAACGTATCAGACGTCAGGCACCTTGACGTCTCGCATGTAAAACCAGACGA
GATGGATGTTGAAGTCTAAAAAAAAAAGTCAGATTCGTTTTTTTGGAAATATCAATCTTG
AAATTGTTAGCACAAAAGGTCAAAAAAAAAATATATCGTCCTCCTTCTCCCCCGCTCCAG
TACACTCACTCTCACTCTCTCTCCTTGGCCGGCCGGCTCCCTTTTTAACCACCTCTGCGC
CCTCCACCACCTTACACAGCACAGCACAACCACACTCATTCACCACCCAGCGAGCTCATC
ACAACCACACGCGCCTTCTCCCAGTCCCAGCTCACTCGGGCCGGCCATGATGAGCGGCGG
CAGGATGAACGGCCCGGCGAGCGACGACTTCCCGTTCGGGGCAATGCAGCCGCCGCCCTA
CGTCGGCTTCGAGCACGCCGGCGGAGGAGGTGGCCAGCGCCATCAGGGCGCGATGATGTA
CGACAACTTCGACTTCGCGGTCGCGGCGGCCGCCTTCGGCCAGTTCCAGGACGCGCCGCA
CCACCAGATGCTGGCGATGCCGCCGAACGGGAGCGGAGCAGGCGGGCTGGTCCCCATGGC
GCCGCCGCCCATGCCCATGCCCGGGATGCAGCTGCAGATGCCGCCCATGCCCCACGGTAT
GCACGGCCACGGCGACGTGTGCCCGTCGATGGGGATGGTGAAGCGCGAGGGCGGCGCCGG
GGACGCGGGGAGGATCGGTCTGAACCTCGGCCGGCGGACCTACTTCTCCCCCGGAGACAT
GATGGCCGTGGACCGGGTGCTGATGCGGTCCCGTCTCGGCGGCGTGTTCGGGCTGGGATT
CGGCCGCGCCCACCAGCAGGCGCCCCGGTGCCAGGTGGAGGACTGCAAGGCGGACCTCTC
CGGCGCCAAGCACTACCACCGGCGCCACAAGGTCTGCGAGTACCACGCCAAGGCCGCCCT
CGTCTCCACCGCCGGCAAGCAGCAGCGCTTCTGCCAGCAATGCAGCAGGTCAGTCAACCC
CACATCCCACTCCACAGTATCCCTTCCTCTCCAGAAACGAAACGAGAGTACTGCGGAGCG
GACTTGGTCAGTATACTCCACCACGTATAGCCGAGCTAGGCTGCAAATTGTGCAGGCTAG
GCCAGTGAAAACTGCTTGCCTTTTCAGTCTTCACTCGGTTTTTGGCTCATGTCGCTTGTC
CGTTGCGGCGGCCATGGCAAGAAAGAGGGTCTGTGCCAAAGCAAGTCTCAGTCTGAGAGA
CGACCGGTCATGTCAAATACAGTAACAAAACACAACTCAAAACCCTAGTCCATGTAGCGC
AAACATGAGTTTTCCGGTCCTGGGGATCGACGTAGACAGGATCACGAAAAGAAAAGGAGA
TTCAAAAGGCAGCGTTTCTTTGGGCTGGCTGCTGCAGACTCCAGACTGCTGCGTGCTGCA
ACGAGCGGCTGATCCTGTGGCAGGCTTTTGCCATGCCAATGCCACATCTCTGCTTTTCTC
TCATTGACCAATGTCGTCTGGTGCTGCCAGTGGCGACTAGGCTTTTGCATCACTGGATTC
ACATGCATGCATGACTTGTTGAGCTAGCTACAGAACTTTATGTTTTTATGTCTTGTGAT
GTACGTGAAATGCGAATTTTATGTTTAGCTTAGCGTAAGTTGATATTGTGCTTTGGATCG
GCTTTAGGTTCCACGTGCTCATGGAGTTCGACGAGGCGAAGCGGAGCTGCCGGAGGCGGC
TCGCAGAGCACAACCGTCGCCGGAGGAAGCCGGCGGCCGGCAGCACGGCGACGTCGTCGA
AAGACTCGGCGCCGCCTTCCAAGAAACCCAACGCCGGCGCCATCTGCAGCTCCTACACCG
GCGACAACAGCAGTAAGCCTACACGTTCGCCTTATCTAGCTGCATCCTACCTGGTGCGAT
TGCTCATCATTTACCGGTGCTAATTTGCACGCACGTACGATCTATGGATCAGCTTTGAGC
GCGACGAAATCGACCATCTCCTCCAACACGAGCGCGATCAGCTGCCTCCAGCTGCAGGGC
CAGGCCAGGGCGGCAGCTGCGGCGAGGCCGACGGCGCTCACCCTCGGTGCGCCGCCGGAG
AAGGACGACCAGCAGCAGCAGTTCAGGAACGCCATGCAGCTCCACCACCATCAGGAGCAC
CAGCACTTCATCACCTCCCTCCTGCACGGCAACATCAATAACAGCAACATCCTGTCGTGT
TCCTCGGTGAGCTCCAACACGATGCCATCGGCGGTGGCGGCCAACGGCGAGGACGACAAC
AACAACATGCATATGTTTGAGGTGGACTTCATGTAGACGTTGGATCTTGAGCAGTAAGCT
TAACATAATTATCTTACCACATTAGATGGTAAGCCGAAGTGTTTTGTTTCCACTGCTTAT
ATGGCGAGGTCCAGTGCTACGGTGTATTGGGGCTTTAGCAACAAAGTTGGTAGGTAATCT
TGTCAGAGTCTTATAGTAATTATGATAGGTTGATAGAAGTACACGACTTGGAAATTGTAT
AAGCACAATCCTAGCACTGGTATTTGGAGTAGTAGTATAGCCCTGGTCTCCTAGATGGAG
GCGAGTAGTGTAAGCTTAGGATGGGTGTGTCCAGCTTGGTTACAGGATCAAGCTTTTGTT
ACGTGGGATTTTGAGAGAAATAAAGAGAACAGGCAGAGGCACATGACATCACGTGCCTTT
GGCAAAAGATCTTGTACACGTTTGTTCATCTAATTGGATGTGATCAATCTTGAGCGTAAA

TTCCAATGCAGAGTGGTGTACTAAGACTACACAAAGTAACATAGGTAGTAACATATATAC
CACTTAAATAAAAAATATGATGTGATAACTAATTAATGAAAAAAGAGAGAAATTGGGTAA
CTTAGTTATTAGTTACTCATATGAGTAACATAACGCATACCAAGGCAAAATTAGTCTATA
AGCTAATAAATAAAAGGTTCAACGTTAGTACTCTCTCCGTCTGGGTTTATAATTCGTTTT
AGGTTGTGCACCGCAACCAAGACGGAAAGGAAAATGAGAGAAATTAATGTTAATTTAGTA
ATTAATACTATTGCATGCAATGAATCCACCACTCCATATCGTGCTAGTTAGTCTCTAGTT
ATTAAAAAATATACACACCCCACGTCATTTATTGATTGATTTCTTTATACATATCAAAAA
ACAAAAAAAGAGGTGAAAGTTTGTGCACCACGCCTAAGTGTTTTGGGACTATTTCGTTTT
CGTAATATGACTTTTAAACCGAGACCGAGGGAGTACTTCTATGTTACTATTCATTATGAA
GGTAATAACTTTGACTTATAACATATGCATTTACTACTCTACTCCCCATTGTGAGTAGTC
TAGTTC
>HORVU5Hr1G117190.5 genomic sequence
TCATGCACCGCATACCTTGACATGGTTATGCCCAAGTGTCACGATACACCTGGATGGTTC
TTTAATGCATGGCCTCACTTACCATTTAACGTCGCGCCCCTTCTCCATACACACATATTT
TATATGTTATTTTCCTAATTTTCACCATCGTATGTCACGCAATTCATGCCGCGTGTAAAA
ACCCAGACTCAAAAGTTTGACCTCCTGAGCCCCCCCTTGAATGGGTCACATACGTTTCAA
GATGATATCCGCAACATTTGGCGTCGCCTGCTAGATATCAACCAGCCGATCATATACGAC
ATAACGACCGCTCGCCGCACCGCGTCAGCCGCGTCGAAAGTTCCCACCCCTCTTACGTTA
CCCCTTCCCGGTTGAGCGTCGCCGCATCGGTCCAACGGCTGCGAGTTTGACCATCCAGAA
GATCCACCTTCCATGGACTGCACACCTCGCCATCATGAACATTACCTAGTATAGTATAGT
ACCATCATAAAACACCAATAATAACTTTGTGATGATAATATAAAAATAAGGTTTGATAAT
AAACAATGAATATCTGCCCCCTGCCGGGCGTGTAGCCGCGCCCGCGTGCCCGTACAAATT
TGGCAGGCCACTTTCTTCTACTAACACACACACACACACACACACACACTCAACTCAGCC
GTCCCCGCTACGCGTCCCCATCGGCCTCCACCATCTCACGCGCCCCTCGCTCTTTCCCCT
CCCTCCTTCCCTCCTCCCCCAGTCCTCCGCGACTCCGCTGCTCCCCAGTCCACCGTTCCC
ACCACAATCCCGCTCCCGAATTCGCCCTGCTCCCGGCGGAACCCGCCGTCCCGACCCTCC
CGGAGCCCGATTCGCCCCCCTCACCCGCCGTCCGCGCCGTCGATTCGTCCGCCCCGTCGC
CCCCGCTGATCCACGGTATGGGCTCGGATTCGCGGCGGTTTTCCGAGTGAGTGATCCCCC
CCGCCCGCCGCGGGCATGTGGGTGTCGCTGGTCTGGATCCGCGGCTGCGTGGGCGTCACC
TTCGTCGACTGCCTGGGCGGTACGGGCGCCGACTAGATCTCTCGGCCCCCGGCGGCGGCC
GCGCGCTGCGGCGGTTTCAGGAGGGCTGCTTGTTTCGTGGGTGCTTTGCTCTAGGGTTTC
GTGGGGATGGACTTGGAGGCGGCCAGGGTAGGGTCGCAGAGCCGCCACCTGTACGGAGGC
GGGCTGGGTGGCGAGCTTGAGCAGGCCAGGCGCGAGAAGCGGGTGTTCGGCTGGGACCTC
AACGACTGGAGCTGGGACAGCGAGCGCTTTGTCGCCACGCCGGTGCCCGCGGCAGCGGGG
AATGGACTGTCGCTCAACAGTTCGCCGTCTTCCTCCGAGGAAGCCGGGCCTGAGGTGGCT
AGGAATGGTAACGTTAGAGTTGACTCTGATAAGAGGAAGCGAGTGGTTGTCATTCATGAT
GATGACAATGAGAAGGATGATGACCCAGTGGGGAACAGTAGCAATGTGCTCAGCTTGAGA
CTTGGCGGCGGCAACTCTGTTGCCGGCGGAGCGGCGGAGGATGGCGGCGTGAACGAGGAG
GACAGAAATGGTAAGAGGATCAGGGTGCAGGGCGGAAGCTCAAACGGTCCGGCGTGTCAG
GTGGAGGGCTGCTGCGCTGACCTTAGCGCGGCAAAGGATTACCATCGGCGGCACAAGGTC
TGCGAGATGCATGCTAAGGCCAACACTGCGGTGGTCGGAAATACCGTCCAGCGGTTCTGC
CAGCAATGCAGCAGGTCCGTTTTCCGATTGAGCTGCTATTACCTTTTATTTACATGTGTT
CTGCCAGCAATGTAGCAGGTCCGTTTTAGACTGAGCTGCTATTACCTTTTATTTACATGT
GATACATTTCCTCGTGTGGGAATGTATTGGTTGAACATGTTATGCACGTTTTTATTTTGT
TTCTATTTCGGGTCTGTTCGGAATGCAACGTGAGGCTGCACTACCAAACTTTTGGCGTTG
ACCATAGGTATTGGCGTCCGTGGATTGTAGCCAGGGTTTCCTGTCCACGGCAAATATTTT
TGTCGTTCATTTAACTTTAACGCCCGCACTCTAGCGAGACGGATGCGGCGAAACAGAATC
CTTCGCCAATAATTTCGCGAGCCTGATTTTGGCGGTGCATGACCTGGGCGTGAAGCAAAC
AGACCCTTTGAGACCAGAGCAAACCCCATAGCAAACTTCTGTCCATGATGATCTTTTAAG
CAATACCATTTGATATGTGAGAATCACCAAACTATATGTCAACTATATGAAAGCAAAGTA
AGCTCTCATTTGCAGATTGAAACGACATGTGGAGATTTTTTTAGCAAATATCTAGCAGA
AAGAAAACAGGAACATCCAACTTCCATAGAAGGAAGAGTTATGGTGTTGTCGCTGGGAGC

AAATGATCACATAGTACCACTGGGAATATGTTCTGAACTTCTGATTACACCTATTAGTTA
TTCATCTATATCAGGATTCGTGAATTTGGTTTATCGAACTTACGTGAGTTATAAGTCATA
ACCAATTCCCAAATACAAATGCACTGTTGTACTCTTAGTTTTCCCTTTTCAGTTGATATA
ATCGGTACAATTTCATTATTTGCATATGTGTGAGGATTAGTTGACGTTGATAAACTTGGA
AATCTCCGTCCTATTCAGATATTTAAGAAAAAAAGAAATTTTCGAAATTTGTATGTTGTT
CTCAGTAACTTAACCTTGTATTAGAGCGCAAAGATATGTTTCACACAGTCCAAATTTCCA
ACTAGACTAAGTTCCATCAAGTTTTTCATGCGATGTAATTGTCACATTGGCTCCTGCATG
TATGCATATTTGGGCATGACATGGCGAAAACTAATGGCCTTCCGTGGTTCATTTTTTCCT
GTTCTTGTACAAGTTATATCTCTAAAGATGGTGCCCACTTTTAGGAAATATTGAAGCCTG
GGCATGACCGGAGGACTACTTTTGAAAGCATTTGATTTACCTAATGTATTTTTTCTGGTA
TGATCATAGTGCATGCATTATGTGTACCTTCTAAAATATTGACAAATCCTCTTGACTACC
GTACATTATGTGCAGATTTCACCTTCTTCAAGAATTTGATGAAGGAAAGCGAAGTTGTCG
CCGGCGTTTAGCAGGCCATAATAAACGTAGGAGGAAAACCCGCCCTGAAAGTGCTGTTGG
TGGGGCTCCTATTGAGGAAAAAGTTAGCAGTTATTTATTGTTGAGTCTTCTTGGAATATG
CGCCAATTTTAACTGTAAGTACTGCTATGAAGGTTCCAAACTTGCTAGAGTACCATATAC
AGCTAATATTCTGTGAATAAGGGTACTAGTATTTGTATAGTTAGTTTTCCACTTAACCAT
CTGAAATTATGTTGACTCCGAGCTTTGACTGAATTTTATTTGGCCTTGACCTAAAAGAAG
ACTATTGTTGATATTGTTTATGATGTCCATTATATCTTTCTTAAATTCGCAGTTGCATAA
TCCTGTGATTTTTAGCGGTGTGAGCTTAGCATCTTTATACTACTTTACTGCCTAATATTT
GTTGTCTATGATGGTTTCTCCCAAATATAGGATAAGAGCATTGTTCTATGCCTAAATTGA
ACTATTATCAGCGTACTTAATGACTTTTAGAACTACTCTTATGGTAATAGTACTCAGGAA
GAGACATGATTCTTAACATTGTACTTGCAGCTGACAATGCTGAGCGTGTACAAGGTCAGG
AGTTGCTATCCAATCTTTGGAGAAACTTGGGGACTGTTGCAAAATCATTGGATCCAAAAG
AACTCTGTAAACTCCTGGAGGCGTGTCAGAACATGCAAAATGGATCAAATGCTGGGACCT
CGGAAGCAGCTAATGCTTTGGTGAATACTGCTGCAGCAGAGGCTGCAGGACCATCTAACT
CTAAGGCGCCTTTTGCGAATGGTGGTCAATGTGGGCAGTCGTCATCTGCTGTTGTACCAG
TACAATCAAATGTTCCCATGGTGGCAACTACCGGTAAGACATATAACCTTTTTAACTCCC
AAGAAATTATTGCTTATGAAAAAATAGCTGATATCGGTATTGCGTGTTTACAGAGATTCC
AGCATGCAAGTTAAAGAATTTTGATTTGAATGACACTTGCAACGATATGGAAGGCTTTGA
GGATGGTTCGAATTGCCCATCATGGATACGTCAAGATTCTACTCAAAGCCCACCACAGAC
TAGTGGTAATTCGGATTCAACATCAGCTCAGTCACTGTCAAGCTCAAATGGAGATGCTCA
GGTTTATTATTTTCATCCTTTCAATGTGCTCAGAAAAAATAAATCTAAATAGCTATTTTC
AACATCTTCGTACCTTTCTCTGACATACATTGGTACTGTTTTGCTTCAAGTGTCGGACTG
ATAAAATTGTATTCAAGCTTTTTGAGAAAGTTCCTAGTGAATTACCTCCAATTTTGCGAT
CACAGGTAAATATGCTCACTCTCGCAAATTAGAAGAGAGCATATCTTGATTTTAACACTT
TAACCCTGAATTTCAGATCCTTGGTTGGTTGTCCAGTAGCCCTACTGATATAGAGAGCTA
TATCAGACCTGGCTGTATTATCTTGACAGTTTATCTTCGTTTAGTTGAGTCTTCATGGAG
AGAAGTACATTTCTTTCATAAACATTTCATGTCATGGTAGTGTTCTTTTTTCTGGCTGGT
GCTTCACATTTCTTTTGTACAGCTCTCTGAGAATATGAGTGTATATCTGGATAAGCTCTC
AAGTAGTTCTGCTGATAACTTTTGGACATCTGGTTTGGTATTTGTGATGGTACAACATCA
AATTGCTTTTATGCACAATGGTGAGTCCAAGCTCTTATATATTCTTACATGGCTTGTCAG
GTTTCATTAAGCTGTACGTCAATATTTTGTAATATCACCTTATGGTCATTTTACTGCAGG
TCAAGTTATGTTGGACAAACCACTGGCACCTAATTCTCACCATTACTGCAAGGTTTTATG
TGTTAGTCCGGTTGCTGCTCCTTTTTCAGCAACAGTTAATTTCAGGGTAGAAGGCTTCAA
CCTAGTCAGTTCTTCCTCGAGGTACTTTATCATATACAATTTGCATAATGCTCATTCTTG
CAGAGGACAAGTACAGCTTGATGTTCACTTCAGCCAATACGCTGTTATCTAAAAATACAC
ATGTCATGTTCACAGGCTAATTTGCTCAATTGAAGGGCGTTCTATATTCGAAGAAGACAC
AGCTATCATGGCTGATGATACCGAGGATGAAGATATTGAATATCTCAACTTCTGTTGTTC
TCTCCCTGATACAAGAGGAAGAGGATTCATAGAGGTACAAGTTATAACGTGTTAACTTCT
GTTCGGGTGTAGTTTTTGTTTTAATAATAACTTCTAAATCATTCTAATTAATGGTATCAT
TGGTGATTTCAGGTTGAAGATAGTGGATTTAGTAATGGTTTCTTCCCCTTCATAGTTGCT
GAGCAGAATGTGTGCTCTGAGGTTTGTGAGCTGGAGGGCATATTTAAGTCATCCAGTCAT

GAGCAGGCAGACAATGACAATGTCATGAATCAAGCTTTAGAGTTTCTACATGAGCTGGGG
TGGCTTCTTCATAGAGTTAACATAATTTCTAAGCATGATAAAGCGGAGCTGCCTGTAGCG
GCCTTTAACCTGTTGAGATTCAGGAATCTTGGTATATTTGCGATGGAGCGGGAGTGGTGT
GCTGTGACCAAAATGCTGTTAGATTTGTTATTTGATGGATTTGTTGATGCTGGGTTGCAG
TCTCCCAAAGAGGTGGTATTGTCAGAAAATTTGTTACACTCTGCTGTGCGAGGAAACTCT
GCCCGAATGGTTAAATTTCTGCTGACATACAAGCCGAACAGAGGCCTGAATGAAACTGCA
GAGACATACCTATTCAGACCTGATGCTCGGGGCCCTTCTGCATTTACACCCCTACATATA
GCAGCCGCTACTAGTGATGCAGAGGATGTATTGGATGCATTGACTGATGACCCTGGACTG
GTACACCCTCATCTACTCATGGAATATGATCTCATGTACTTGCTTGTGTTCTATGTTGTC
TAGCTTTCTTGATCTTTTAAGTTTCATTGCTTGGATGTTTAAGCTTATTATTTAAGGCTT
TTCTTTTTGTTATCTTTTAGCTTCTGCACATGTTTGACATCAGTTTAATGATGAACAACA
CATTAATGATGCTATACTACTTATCCTCTTAGCTTAGTTTGTTTCCACTTCCTCCGTCCC
ATATTAGTTGTCGCTCAAACGGGTGTATCTAGCAGTGAAATACGTCTAAATACATCCGTT
TGAGTGACAGTTATTTCCATACGGAGGGAGTATTAATTAATTCTGAAGATCCTGTCTAAT
GCCCCTGCTGTTGCTTCTTGCAGGTGGGACTGAACGCATGGAGAAATGCGCGAGACGAGA
CAGGCTTTACCCCTGAAGACTATGCTCGCCAAAGAGGCAACGATGCTTACATCAATCTGG
TCCAGATGAAGATTGACAGGCATCTTGGCAAAGGTCATGTTGTCCTTGGTGTTCCCAGCA
GCATGTGCCCTGCAATTACCGATGGGGTGAAGGCAGGTGATATCAGCCTGGAGATCTGCA
AAGCCATGCCAGTGACAACAACATCCGCCGCAAGGTGCAACATTTGCAGTCGTCAGGCTA
AGATGTACCCAAGTTCCTTTGCGAGGACCTTCCTGTACAGGCCGGCAATGTTCACCGTGA
TGGGCGTCGCCGTGATCTGTGTTTGCGTCGGGATCCTCCTCCATACCCTCCCCAAGGTTT
ATGCCGCACCAAATTTCAGATGGGAGCTGTTAGAGCGCGGAGCCATGTGAGGGGTGCGTG
TCGAAATAAGGTTAGGGGTGTTGCTGTTAAGAGTTTTAGTGTGGTTGTTAGATCAAACAA
ACATGTAATTGTGTGAAGAAGTTCCGTTGTATTTAAAAATTGCAGAATCTTTGCCCTGAT
ATAAATTTTATATACCGTCAATAATAAATTGATTGTACATGTGCCCGTTTATTTCTATTT
GGTTGCAATTTTAGGCTGTGAAAACCATTTTTGAGAGTTGTGTGTTTAGAAGAGAATCAG
ATTAAAACATGTTCTTTAATCCTTCTTTGTAATCTGTATATATGCGAATATCCATGTCTG
AGCCGGGCTCATATACACCTGGTGAACAGTAAATTTTTAAAAGAAAAGAAAAAGGAAACA
AAAACAATTCAAAAAAAATCCTTTTGTTTTGCATGGAAGATGCTTTTGAGGTCGGTTTTA
AATTTCAGCTTATTTGAACATTTAAGTAGCTCTAAAAAAAATTGAATAAAAAAAGTATATA
AACAATAAACTGCTTCACAGATCCTAAATTTATCATGTTTTGTCAAAATCTATTCAGATA
TTCAAATGAGCTGAAATTTAGAGCGGACCTCACGCATCGAAGCATCTTTTATGCACAAAT
ATTAATCAGGTATCGTCATTTTTCCTAATATTTTTTGAATTTACTGTTCATGGTAGGTGT
TGAGGAGCTCGAGAGCCAAATAGCCGTGCTCGTATATATATGCTGAATTCTTATTGTTTA
TTTTCTCTTCTAGAGTGCCTAGGGACCTGTTTGGTTGCGTGCATCTAGCCCAACCAGGCT
CCGGGAAAGCGCAC
>HORVU1Hr1G060770.1 genomic sequence
TGAATTTTGGCATGAGCACGCCCTAATATTCCGTTCCTGTGCACTTGTCAGCGCTCGGAC
TAGAAAAACGATGAAGCTTGTGCCTTATCCGTGAATGTAGTAAACACTAATAACACGAAG
CCCAATGTCAACTCTCACGTTCCCCTAAAAAAAAAGAAAATGTCAACTCTCACGATAGTT
TTTCTTTTTCTTTTTAAAAGGCACTCTCATGATGGTTCTTTCCCTTTTATAAAAGGAAAA
AACTCGGGTAACGGGAAAAAAACACTCTCAAAACGGAAGACATTCCAGCGTCCATCCATC
CAACAAAAAAGCAGACTGTTTCTCGAAAAAAAAAGGAAGACCGTGTCGACACGATGAGAC
CATATCGGCACAACCCCTCCCGTCCGCCCGCGTCCCAATCCGATCCCCACCGCCCAGATA
CCGATCCAATGGCCGGCGGTCGCTGTCGTATCCGCCGTCGCGCGACGGCAGCAACAGGAC
GGGGGTCAGCGCGTGCGTGTCGACCCCTCCCCGCTCGCTGACACGGTAGGACCCACGCCT
CCGTCGCTGTGGCTTGAAGATTCGCCCACTCGCCAAACACCCGCACTGCGCCAGCAGCAC
CTGCTCTCCGTCACGGCGTCACGCCGTCACCAACCGCACGCGGCACGCCGAACTCGAGGG
CCCACGCGCCAGCAGTAGCGCGACTGGCCCCGGCCGCCCTACGCCGCTCACCTACCATTA
CCAAAGCCTTTCCTTCCTCCTCCCGCGACGAGACGACCAATTCGCCCGCCCTCGTCGCGT
CGCCGGCCGCCTTCTCCCCCCTCCCCACCACGCCCGACGCCTCCGCGGAACCGCGAACGA
GACCCAGCGTAGCGAGAGCCGATTGGGTCGCGCGCGCGCGCCGGGTTTTGGGGCTAGGG

CTTGGCTCGTGGTCGCGGTTTCCAGTTCCCAGGAAATGGACGCCTCCGACTCCGGCGGGG
GCGGCGCGGCGGCCGATGCGGGGGAACCCGTCTGGGACTGGGACAACCTCCTCGACTTCG
CCATCCCGGATGACGATTCCCTGCTCCTCCCGTGGGATGACCCACTCGGGATCGAGGGCG
ATCCCACCACCGAGACGGCGCTGCTCCCTGCCCCGCCTCCGCCGCAGCCAGTGGAGCCGG
CGCCTGCGCCACCGTCTCCATCTGTTGAGGCCGGGGCAAGCAGACGCGGCGTCAGAAAGC
GCGACCCGCGGCTGGTGTGCCCTAACTACCTCGCGGGGATTGTGCCCTGTGCGTGCCCCG
AGTTGGATGAGATGGCGGCCGCCGCGGAGGTCGAGGAGGTTGCCGCGGAGGTGCTTGCCG
GTCCGAGGAAGAAGCCCAAGGCCGCCAGCCGGGGTAGCAGCGGGGCGGTAGTCAAAACTG
GTGGTGGTGGCAGTGGCAGTGGAGTGGCTGCCCGTGGGGGAGCAGCAGAAATGAGGTGCC
AAGTGCCGGGCTGTGAGGCGGATATACGGGAGCTGAAAGGGTACCACAAGCGGCACCGGG
TGTGTCTGCGTTGCGCGCACGCGTCTGCCGTCATGCTCGATGGCGTCCAGAAGCGCTACT
GCCAGCAGTGCGGCAAGTAAGCTACATTTGCCCCTTGTTAGTTGCTGAATATAAGTTTGT
ACAAGACCCATTGTTATTATTAGGATACACAATTGAATACTTGAAATGTTTGTACAGTCT
TCATCCCCTGACTTAGTTGTAGTTCTTCGATAAGGCAGGTCGTTGGATCACTATTTCCAG
CTTCCTTCATTGAATTTGTCGAGTTAAGGACTAGTGCAACCGCTACATTTTTTATTTTTT
GAGTAGAGTTCCTCAGGCATATGCTGTCACGGTTGATTTGTCTTGAGTGTTCTTCAAGAT
TTATGCATTAATTATATTTTGTTTGGCTTAAACTTATCACGAAAGACATGCCGGAGCACA
ACTTTGTAGCTGTAATGAAGTAATTATGTGAAAAACCACTAGACTACCATGCTAGAGTTT
TTTTTTTCTTGTAGTAGGCTATACTAGTGCGGTGACATGCAATATTTGATGTATGAGCCC
CTGCAATTCAATTGAAAGCAATCCCGCCAAAAATATTCCGGATATGCTAGTTACATATTG
TTTTGTTTGGTCCCAGGATTCAGGCATCCTGGTGGAACTTATCTAGGATTTGTCGGGACT
CTCGTGATATTTCCAGTGAATTCCGCTTTCAAGTAGAGTTAATAACATAATAATTTTTTC
GATTAATGGTAGACACACAAACACATGCCTACACCACCACAAGCCACATGTGAGTGCATC
CCCAAGCACATGCCTAGAATTAGCAAGGGCCGGGAATACATTCGCTATCCCATGAAGTTC
CCACCGAAGCACATCCGCATCCGCATGAGCAAAAATGCGGGTGCTAGGATTTAAGCATTT
GTGGGTGGAGTCGTACACCCACAACTCCACCAAGAGGTGGTTCCAGAGTTAGTATGCTAT
AAATGGTCGAGAACTCTCACTGGGTGCAATGGTGGGAACACATGGTTGTGAACACACCCA
TCTGAGTTCGGGTACTCATCTCGTGTAATGGAAGACAGGTGCGGGGTGGTGGTGGTGATG
GTGGTGGNNNNNNNNNNNNNNNNNNNNNNNNNNNNNNNNNNNNNNNNNNNNNNNNN
NNNNNNNNNNNNNNNNNNNNNNNNNNNNNNNNNNNNNNNNNNNNNNNNNNNNNNNNNNNN
NNNNNNNNNNNNNNNNNNNNNNNNNNNNNNNNNNNNNNNNNNNNNNNNNNNNNNNNNNNN
NNNNNNNNNNNNNNNNNNNNNNNNNNNNNNNNNNNNNNNNNNNNNNNNNNNNNNNNNNNN
NNNNNNNNNNNNNNNNNNNNNNNNNNNNNNNNNNNNNNNNNNNNNNNNNNNNNNNNNNNN
NNNNNNNNNNNNNNNNNNNNNNNNNNNNNNNNNNNNNNNNNNNNNNNNNNNNNNNNNNNN
NNNNNNNNNNNNNNNNNNNNNNNNNNNNNNNNNNNNNNNNNNNNNNNNNNNNNNNNNNNN
NNNNNNNNNNNNNNNNNNNNNNNNNNNNNNNNNNNNNNNNNNNNNNNNNNNNNNNNNNNN
NNNNNNNNNNNGCTATGGATGTGAACATCAATATTCTTTTTGGTACTTAAAACCTTCTAG
AAGAAGACGAGAACATGCTACAACCTACAAGAGATAGAGCAAAGGAAATATCCCAATTTT
TGTGAACTTTTGCAACAAGAGCATTTTATAGGCAGGAGCTGTGTTCTTCACAATGTTTCA
CCACCAGAAAGTCAAGAGGAACATCTTTTACCACTTCAAACAAAGTCATAACACCACAAC
CAACAAAAACTAGAATTTAGTTGTTACTTCCAGCTTAAAGGGCACCTTACCTTTAAGCGC
TTATGCGGAGTTAGCAGCCTTGATATGCAGATGTGGATGGATTACATTCAACACTTTCTC
GGGTGCATATTAGGGTTGTTGGCTAAGGGGTACAACTGGACCTAGCCAACCAAATAAGGA
TATAATCCTATTATTAGGAAGCTAGTGATGAGTATATAAGGAAGCTGCCAGCCTTATGTT
ATTATCATTTTTAATGTGAAGGAACATGAAGAAAATAATCTAGTCTCTCTCTATGTTCTT
CATTCCAGTCCAGATCCCGTCCTTTTCCTGAGCCCTTCCTCCTGAGTACGCTAGTTAAAT
TGGTATCAGTGCCTACAGCGTGGCTTTTGCTGCCAACTTTATGGCGCACAGTGTGGTCTG
AGCACTGAATGAGTTATACATGGAGATCAACAGTGGGAAGAGCTTGCATGCGCGGAGAAA
ATATGTGGACTTGAGCCACTGCTTCTGTGCTAGCTGCACCAACCCTAGCGGCGTATTTGC
TGGCCACTTCCTCTTCAATGCCATCAGATGGGACTGTAGTACTGAGCCTGTAACCAGCAG
GAGTGGTATCTGCTCATCAAAAGGTGTTGGCTGTTCATGCTCCAGGCTTCCAGAAACCGG
GTTGATCCACCATGGGACGCCGAGTGTCTAGTCGTTTTCCACTGCACACAACTTCCTCCA

AGCTCTAACCTTTAGATGTGCTTGTGCCTCAGTGGCTCAGTTCAAATCCAGGCCAGGTGG
TCCTCCATTTAGCACTCAAGCCGAGCCCTCTGCTGTGGACCTTGGCCTGGCCTGCAGTAA
CAGGGCCAAGATTAGGTAAGTGCTTGGCACTGTTACCACACCCATACATGGGGGCAAATA
TCTTTGAAACATCACCTTATTCGTCTAGTGAGAATCTCTACATCCCATGAATGTGGGGGG
TATCAGAGACTTCAGCTAGGGATGAACAAATACTGTTCAACCATAAGTAAGCTGTGTTGC
AATTTTGTTAACATTGCATCAAGTCTATAAAAACTAAGAACACTGCAGCTCTAGCCATAA
TTTTGCAACTCGAGAATGAGCTGCTTATCCAGGAGGAGCGGAGTGTCAGGTCCAACCTAG
GAGTCAGGCGGCAAACTGAAGGAAGTATTAGCAGCTGGCCCTAATCAACTTAAAGTGTGG
ATATGTACCTTACAATTAGCAAATCTGAGAATAGTTATATTAGTTAGGTACATGATTGAG
ATTAGATACTTTAGGGGGAGACCCATCTATATAATGAGACTGACACATGCTATTACCATC
AAGAAGTCCTTGAGTTCTTCTACCTAAGTCTCCTCCTTCTTCGCCCTGACATCATTTTCT
GGTGGTATTTACCCTCATATCAACATAAATAAATAAACCATCTCATACATTTGAAGTTGT
TTCCAACAAATCAATAGCCTCACTTCAACATAGACATGGTTTTGTTTGTAGCAATGGATC
CACATGATCAAAAGAAAAATAACACTCCGAAGTGAAATACAACAAAGTTAAACACCCTCT
AGCCATTTGTTTAGAGCATGATCCAAAGAAAAAGAACACTCCGAAGTGAACTTGAACATA
GTTGGTATCTTTGTTGGAATTCTAAGTTTGACCCAATTGAGCTCCAGTAGCAGCAATATC
TTTATATATGCTTGTTCGGTAGGGTACATTTTAAATGCATATGTTGCTGTTCAGCTCCCT
TTCTTCTAATGTTCCTTTTTTGTGCACTCTGTAAGTATGCGCTGTCTAATCCGGAACATT
TTTGTTAATATCTTTTGGTATACAGAGAATATAGATGGCGTCGCCCGTGACATGCTTGAG
ACTGTTCTTAGCAACAAAGTTTTAGACAGAGCAACACCGGTGGGATCTGAAGATGCCGTT
AGTTCTCCAACTTGTACACAACTCAGCTTGCAAAATGACCAAAGTAAAAGCATAGTGACT
TTTGCAGCTTCTGATGAAGCTGCAAAACAGGAAAATGCGAAACTTACCAATTCCCCAGTG
CATGACAGCAAGAGTGCTTATTCATCCTCAGTGAGTTTTTCCCCCATACAAATTTGTTTT
ACTTCGATGAGATCGTTATTCGTTATTATTTTAGTTTGGTGGAACTTCCCCCCTCCCTCC
CTCCCTACCTCTATAATTGTAGCAGTAATGTTGTAGCACCATTCTAGTGCCCCACAGGAC
GCATTTCGTTCAAGTTGTACGACTGGAATCCCGCGGAGTTTCCTCGGCGTCTACGTAACC
AAGTATGTAACTGTCTATGACCTATAAGGCCTTGTACACTGCAAGGTGCTTAGTAGAATA
AACCGAGTTTTTCTCAAGCACCAATGCTCATCTGTGCAGGAGGGGTGCCTAATTAAGCGT
CTACCCTCTACAAATAAGCACCAGTGCTTAAGAAAAACTGGCCTGTTTTTCTAAGCACCT
CCCCTAAGCACCTTGCANNNNNNNNNNNNNNNNNNNNNNNNNNNNNNNNNNNNNNNNNNN
NNNNNNNNNNNNNNNNNNNNNNNNNNNNNNNNNNNNNNNNNNNNNNNNNNNNNNNNNNNN
NNNNNNNNNNNNNNNNNNNNNNNNNNNNNNNNNNNNNNNNNNNNNNNNNNNNNNNNNNNN
NNNNNNNNNNNNNNNNNNNNNNNNNNNNNNNNNNNNNNNNNNNNNNNNNNNNNNNNNNNN
NNNNNNNNNNNNNNNNNNNNNNNNNNNNNNNNNNNNNNNNNNNNNNNNNNNNNNNNNNNN
NNNNNNNNNNNNNNNNNNNNNNNNNNNNNNNNNNNNNNNNNNNNNNNNNNNNNNNCCTAA
GCACCTTGCATTGTACAAGGCCTAAGTTTTTAACAGTGGATGATATTTTCATGTCAAAGA
AATTATTCTAAGTCAGTGGTACATTTGAAATATGCAGATATTTGAGTGGTTGTCTAGCAT
GCCTGTTGAATTGGAGGGCTACATTCGTCCTGGCTGCACAATTTTAACTGTATTTATTGC
AATGCCACAACATATGTGGGACAAGGTATGGATGTTTCTGCTAAAACAAAATCATCAATT
ACTTTGATGTGAATGGAGAATTTTGCACACATCCAAATAAAGACAAACTATCAATATTTA
TTTAGCAAAAATCATTTTGTACCGTGTAACAATTATGCGAACTGCTAACTGTAGTACTGC
CATCGGTCCAGAATTGATGTCTTTATGGCTTGTGCGAAGTTAATTTTTACCACTTATGAG
ACTGATCATTTCAAAAGGAATTTGTTGGATTTGCTGTGAAGAATTTTATTATATGATAAC
CTTATTAACTTTTATCAACATATCATCGAGTGTCCCCAATTCATATCTGCATTTTTCTTG
ACAGTTATCAGAAGATACAGCAAATCTTGTCAGGAACTTGGTAAATGCTCCGAGCAGTCT
TCTATTGGATAAAGGGGCCTTTTTTGTTCATGTCAATAACACAATATTTCAAGTATTCAA
AGGTGTGTGCCATGTACTTTGCAATTCCATGACATAAATACACTAACCATACTGTTTCTA
TGTCATTCTTGAACTGTGTTGTTTGTACACAACATATGTAGTCCCTCCTTTGCTGATTGC
ATACTGTTTTTGGAATTTACTCTGCAACAAAGATGTATGCAGAAAAGACTGCATCGTGGA
AAGTGCCCTTCTGCTCCCGAGCTCANAAGGCGCGACAAAAAAAAACGATACTCTCAAAAT
GCACGTTTTCAGAGTATCGATTTTGTTTTTGTTTTTTTGTCGCGCCTTATAGGAATGTTA
TTTCAGACCAAAATTTTATATGCACTTAGAACTTTTGTCAATGTTGCTCACAAAAAAAAT

CTGAATTTTGGGAATTTTGGCCTGAAATTACTGTTCACCTAGCTCAAATGAGCTCGGGAG
CAGAAACTCTGCATCCCTGCATCCCTGCATCGCCCCCTTTATTGGACAACTTATTACATA
TGGAAGTTGTGTGTTACTCTAGTCCATATTAATAAATGATTGGAAATAGCCAAGATGGGT
ATGCAGTGGAAAATTGGTGTAAGGTTATCTTGAAATGTTAAAATGGCAAGCATTCATGAA
CAAAAAAATGTTTTTTGGAATGACATGTGAACAGGAACAGAAGGAGTAGCACTGCATGTG
CTACTGGGCTACTACATGCCGTAGACTCTTTGAATTATGTATCTGCCCAAAAGAATCCTC
TCATGACAATACAATCCCTTTGTTTTGAATTGTTCGATGCAAGGATCCTTGTCCCATTCC
TCTGACATTTGCAGTTTTAATCTATAATTATGTAGTGTTCAAATATTTGAGCATTTCCCA
GTTAATGTGGGAACTATTTCCATTTAACCTTTTATAGATTATTGCACCTTTTGTGTTAAA
CTTTTCATGACAAAAGTATTTGGTTACTAACTGAGGATTTGTAATATCCGTACTTAAGAT
GGAGCAACATTGATGAGTACCAGATTAGAGGTACAAGCCCCAAGGATCCATTGTGTTCAT
CCAACATGGTTTGAAGCAGGAAAGCCTATTGAACTCCTCCTTTGTGGAAGTTCTCTTGAT
CAGCCTAAATTCAGGTATAATATGCCCTTGGATGTTGTGGTGATTTCTGCTGGCTGTTTT
TCTACCATGTATCATCACGTGGTACAACACTTTTTGTTTGAAAACTTCTGTTGTATGTAT
CATTTTCTAAGCGTAGTGTTTATTGTCACAAATTCGTGATGTGGGCTTACCAGAGACTCC
ACGGTGAAATTCGATCTTAGCTTCTACTGAAATAAATATGCTCTGTTTCCCCATAACCTC
GTGTTTTGTATTTTTCCAATGATCTTGTTTAGTTGGTCAAGCCATTAAATGAACCATTCC
TGGGTTTTAGCCTGTTCCTATCACTTGGAGGAACTAAGCGAAGATGAATGCAATGTGACT
CCAGTATGCATTTCTTTGTTTGGTTTGTTTCAGGAACAAACTGTCTTGAGAGCTCCACGC
CCAATTTCTGCATGAAATAGCTTTAGGGTCCAAATACAAAAAAGTCATTTATCTCCTTTT
CACAATACCACCTGTTGGGTGGAAAATATGTGGTAATGGAAGTACCTTTTTTTTGTTGTC
CAAAAACAAAACAATCAGAAATTTACAGAATACAATGGAGATTAAGGAATGCGGTTGATG
TTGGCTTTAGAATTGTAACTATCTGAATTACTCCGCATTGCACTGCTAGCCTGGTTTTCT
TAGTCAAACCTGTATAAATCATTGAGGTAGATACTCTTGATAATTGTTGATCATCGGGAG
AACACTTCTGGTTTATGATCAATGCCCTTCTATTGATATACTTTATGGGTCTTAAGTTTT
CATTAATGGATTTTGTTTTCCTGGTTCTAACATTAAATTTGTGCATGAAGTGGATCCACA
TTAGATAGTTCCTGTATGTAAGCTTCATAGATGAACACTATTTATGCATTTGCTAGGGGT
TTGCAATTCTCTATGGGTGCTTGAATCACCACTCAGTTTATGTTCTCATGGCAGATCACT
TCTTTCGTTTGATGGAGAGTACTTGAAGCATGATTGTTGCCGTCTAACGTCTTATGAAAC
CTTTGGTCGCGTGAAAAGTGGTGATCCTACATTTGATTCTCAACAGGAAGTTTTCCGGAT
AAACATCACTCAAAAAAAGCTGGATACTCATGGACCTGGTTTTGTAGAAGTAAGTGAACT
TTGCCACAGAACCAATCAAATTCCTCCATACTTTACAGTCTGGTGCATTTTACCTACAGC
ATTTTTTCTGGTGGCATCCATCTAGGATGCATGTGTTTGACATAGTTCGGTTATGATACC
TTGTCAGTCCTATATATATTGTTACTAGTGTGTACGAACTTAAGCTACAGTTTTATATCT
TAGCCATGACCAGCCTGACATTTGTACTCCCTCCATCCCAAAATAAGTGTCTCAACTTTG
TACTAGCTCTAGTACAAACTTGTACTAAGTTTAAGACAGTTATTTTGGGATGGAGGGAGT
GCAATTTAATAGAGAACCCTGCTTAGGATGCTTAAGCTCAAGCTATCCCTCCCAAATAGT
GTGATGTTGAACTGATTACTGAAATAAGGCATCAATACTCGAGAGATATTAGCTGCTCTA
CAGTACACAGGGATTTTGGGAACTTTGGTTCATATGCACCAACTTTGGTTTGTGCACTTT
AAGAAGTGAAAAATAACAGCATAGAAGCTGGGCATAATAGAATTGCAAGTTTCTGTACAA
AAATTAGATCATATGCGCCCTTTGCAAAAACACGAGCAAAACAGAACTGCATCAGAAAAT
ATGTTTATCACAACGACTGGACGATGGAAAGCTATAGGAAAACAAAGTGCATAGTGGGGA
TTTTTCGGCTAGTAAGCTGTATTATTCTTGGCGCTAACAGTATAGGTGTATAAAACCGGA
TATTCAAATCGGCACCCAAGAGCATACGCTCCCGTGGTGAAACAAATGATGATGTTCTTA
ATGTCAAAGAGATCTGAAAAAGTTATACATGTACATACATGCCTGTCCTATGTGTAGATT
TAAATTTCATGGAAAAGGATAAGATTTCTGGTTTGTGTGGGAAAAAAATTGGGGCTTCAA
AATTTGATTTTTTAGAGGATTGGATTTTGTTTATTGTCCAGGACACAAATTATGTCCATG
AAACTTTGCAAGCAGATAAATAACATGAACAGGTATATTATAAAAAAAGAACAGAGTTGT
TTGAATTTTTAACGATTTTACTATTCATCTGGGAGTGTATGCTTGTGGGAGGCAAAACAC
CGCCTTTGAAAGTAGAGCCAAAATTTACACCTTGAAATAACATTATCAAAAGAAGACAAA
AAATTCATTCTTTGTGCTATAATTGCAATATTTAAGGGGTTCCAAAATGGTTCATTGTTG
CAAGAATAACAATTGCTCCCTCTGTAAACTTTCATAAGACGTTTTAGGTCACTAAAGACA

TTTTATAAAAGCTTACAGAGGGAGTGCCATTTATGTAACTGCATGACTGTTCCTGTTCAG
CTTATGTTGTCATTCTTTGACACTTTCTGTAGTCGTCGATTGCAGTTCTATATTTTCAGT
TCTGTCTTTTTCTTCTACAGGTGGAAAACGTGTTTGGGCTATCAAATTTTGTCCCTATCC
TTTTCGGTAGCAAACAGTTATGTTTTGAACTAGAGAGGATACAAGATGCTCTTTGTGGTT
CTTCCAAGTACAAGAGTGCAAATGGAGAGTTACCTGGTGTTACTTCTAACCCTTGCGAGC
TTTGGGAACTTCAACAAACTGCAATGTCGGGATTTCTGATAGAGATTGGGTGGTTATTAA
AGAAGCCCTCTCCAGATGAATTTAAAAATTTACTAAGTAAAACAAATATCAAGAGATGGA
TATGTGTGTTGAAGTTCTTGATACAGAACAATTTTATTAATGTTCTTGAAATGATTTTGA
TGTCATCGGACAACATCATAGGCTCTGAGGTTCTTTCTAACCTAGAAAAGGGGCGATTGG
AACATCATGTCACAGCATTCCTTGGATATATAAGACATGCTCGAAACATTGTTGATAATA
GAGCTAAACACAACGAGGAAACACAGCTTGGAACAAGGTGGTGTGGTGGTAGTGCTTCAG
ATCAGCCAAGCTTGGGCACTTCTGTGTCACTTGGTAAAGAAGTAAGTGCTATTGTGTTGC
AAACCCTATTAATTCGAAGCTAAATATTCCTCTGGAACTCTGCTAATGCCAGAATTTCTG
CATGTTTATGCATTTTACATTAGAGCAGTTACAGATTTTCCCTTTTTTAAGCAGAGCGAG
TTCAAAAGATTACATTTAAATGATTTTCAATGTTCAGTTGTGGGTTTTATTGAGATTTTT
CTTGTGTAGATGTAATCATCAGCTCATTACCATTGCGTGCTACAAGCATGCAAACACACG
GTCAGCAGTAAGAAAATCAGGGAAATCATGATACCGAGCACAATTCTTAGAGGAACATCT
ATGTATAATGTGTCCTATTTTGTTTCATTTTAACTTCTATTCCCTCCGTTCCTAAATATA
CGTCTTTCTAGAGATTCAACTAAATAATCTTTTTGCAGAATGTTGCTGCCAGCGATGAAT
CTTGTTTTCCCTCGACCAATGAGTGTGAAGAGGAAGAAAGTGTGCCGCTTGTGACTAATG
AAGCTGTGTCACACAGGCAGTGCTGCCAGCCTGAGATGAATGCAAGATGTCTTAGTCCCG
CCTTGGTTGCTCCTTTTCCAGGTGGTGCTATGAGAATGCGACTCATCACGACTGTGGCAG
TGGCTGCTGTATTGTGTTTCACGGCCTGTGTTGCCGTGTTCCACCCAGATAGAGTAGGGG
TGCTTGCAGCTCCAGTAAAGAGGTACTTGTTTAGCGACTGCCCACCATGGAACCATAGTG
GATGTTAGATTAGATTAACTTTCTTGACTTGTTGTTGCTCTCCCCTGTGCCATGTGTATA
CCGTTGTGTCGAGGCATAGATTGTTTGTATCAAGGATAAAATACCTTTTCCAATGGACAA
TGCAAACTTGCATCTTTTTGACAGAAACTATGGGCATATATGCATAAAGTTCTGAACCAC
CAGGTGTATCAGAGGGTTTGTTAGAAGTGCTTCATTGAAGAAGAGACCGGGTTTCAAAAC
TTCTTTTGTTCGCATGTAGTTGTGCTATTTAGCCTTTTATAACTTTTGACAAGTGAGAAG
TTTGGAAGAATCTAACTCGAACAGTTTCTCAAATCCTGTGTCAGAAGATTCATCTGATTT
GGGGCTTGAATATGATCTGAAGAGTACTCCGTTTCAAAACAAAAATCCTATTCTGCTCTT
GAGCTCATATGCTCCCTAATGAACAGCAGAATCAAGAAAAATAGTAAAATAATAAAAAAT
TCTGATTTTTGGGGGTAAACTTTGTATGACATTTGTGGGAAGTGTGGCAAAAAAAAAGCA
CTCTAAATGTATTCAAAACTAGTAGCATTTTTGGAACATCAATTTTGTTTTTTTGCCACA
ACTTCCACTAATGCCATTTCATCCTGAAATTTTACAAGCATATAAAACATCTGTGGAAGT
TATCACAACAAAATTCAGATTTTTTTGAATTATTTTACTATTTTTTGGGTGTTATTGTTC
ATCAGGGAGCATATACACCAGACCCGTTTCAAATTGTTTTTGAGGAGTTACCTGTTTCAA
ATTACATTAAGTTCTAGCTTTGTTTGT
>HORVU0Hr1G039150 genomic sequence
CGAGTTATATGGCTCACGTTCTTCTCGTGCAGGGCTCCGGATTTTCGAAGCTGGTCGGTCGACCGGC
CCTCGGATGTCTGGAGGGAGCCGGAAATCCGAG
TTNNNNNNNNNNTGGTCGGTCGACCGGCCCTCGGATGTCCGGAGGGAGCCGGAAATCCGAGTTATA
GGGCTCACGTTCTTCTGGTGCAGGGCTCCGGATT
TCCGAAGCCTGTCGGTCGTCCGACCCTTGACCGGCCCTCGGACGTCCGAAGGGAGCTGGAAGTCCG
AGGCATTAGACCTGTTTTGAGATAGGAGCAGAGT
AGAGAATATGTGGTATTGAGCAGAATAGTGGATATCTAGTTTGAGCAAGTTCATCACAAAAACCTG
TGATCCCCTCTTAATAGTGCGGGATCCCTAAAGA
ATCAAGAATTGTAAAAGGGGTACCGATGATCCATACTTGAGTGTATACTTTTATTCGCTGACCATCA
CTCCGCACCGCTAACATCAAAGTAACTGATACC
TTGAGTTAGCCCTTTCACTTGAGCTTGATGTTGTTGTTCCTTCTTGGCTCAATGTTGAAAGCACTACA
TGATGAAGTCTTCAAGTAGCTCTCCCATACAC
AATGTGGAAAGCCTAGCTTATATATTTATCTTTATTGTCCACCATGTGAACATCCACAAGGATCAA

GCATGTAGTGCTCAGGAATNNNNNNNNNNNNNN
NNNNNNNNNNNNNNNNNNNNNNNNNNNNNNNNNNNNNNNNNNNNNNNNNNNNNNNNNNNN
NNNNNNNNNNNNNNNNNNNNNNNNNNNNNNNNNNN
NNNNNNNNNNNNNNNNNNNNNNNNNNNNNNNNNNNNNNNNNNNNNNNNNNNNNNNNNNNN
NNNNNNNNNNNNNNNNNNNNNNNNNNNNNNNNNNN
NNNNNNNNNNNNNNNNNNNNNNNNNNNNNNNNNNNNNNNNNNNNNNNNNNNNNNNNNNNN
NNNNNNNNNNNNNNNNNNNNNNNNNNACCACCACC
TCGGTGGGTACAGCCTCGAGCCCAGCTCCCTGGCGCTCCTCCCCCCGTCCAGCCACGCCACCATCGC
TGCCCACAGCCCCCACGACATCCTCCAGTTCTA
CCACCACCCCACCTCGCACCACTACCTCGCCGCCGCCGCGGCAGGAGGCAACGGCAGCCCCTACGG
CCAATTCGGCGGGGGCGGCGGCGGCGGCGGCGGC
TTCCAGTCTTACTACCACCAGCAGCCGGGGACGGGCGCGCCCGAGTACTACTTCCCCACCTTGGTCA
GCTCGGCGGAGGAGAACATGGCGAGCTTCGCGG
CCACGCAGCTGGGGCTCAACCTCGGTTACCGGACCTACTTCCCGCCCAGAGGCGGCTACACCTACG
GCCATCACCCGCCCCGGTGCCAGGCCGAGGGCTG
TAAGGCCGACCTCTCGGGTGCCAAGCGCTACCACCGCCGCCACAAGGTCTGCGAGCACCACTCCAA
GGCGCCCGTCGTCGTCACCGCCGGCGGCCTCCAC
CAGAGGTTCTGCCAGCAGTGCAGCAGGTTAGTAGCCACACTTGATGCTATATATATCAATTCGCCGG
AATTCGTTGATGAATTGCGTGTCTGATCGATCG
ATTTGCTAATCTAAAGAGAGATGGGTAGATCTTGTAGAGCCGTGTTGGGTTGGAAAATGTTCTCCCT
TTCTTGAATCATGACTTGCATTTAAGTGGCCTG
CAAGTTGCACTCAGTTTGATTGAATGGTTTGGAACTTCAAACTATCCACCTCGTCGTTCGATCGCAA
AAGTAGCCTCCAAAATACCCATTTAATTAGTTC
GATGGAACGAGTAGAAAAGTTTTGAGATCTTGTTAGTTTGAGCTCCTGGATGGTGAGTACCTCGAGT
AATTGCTTCTAGTTGGAAAATTAAACAAAGATG
AAATTATGGCCTTGAACATGATGCTATATTTCTTTGCCACATTTTCTGTTACCCTCCTTTCTCTAGCTG
GATCTTAGAAGTATCCAATCTACATTGCACC
TATTTAGATTCCTCTTCCAAGGATGTTCATACAGTACCTGTCCGACCCTTAACTGCTAGATCGAAAC
AAAACAAATATGGGTGAAAACAAAACAAATATG
AGTGAAAATTCCACATCATACCCTGTTCTTTCACTGTTCTCCTCCAATAAATTCCAAAGGAAGCACT
ATAGCTTTTAAGCAAGCAAAACAACTGGTGTAC
TGTACAATACCGCGACCCACAGCAAACCCCTCGATTACTGGTACATGGAAATTTTGCAATGAACAA
ATTTAGCCCTTTTAATTTCGTCATGTAACGATAC
TATACACTGCCGTGATCCAGTGGAACGAAGAACTTGGCAACGACCAATTTTCCAGTAGTGGCCAGA
CGTCATATTGAGTGTTCCCTCAGGCTGCTTGGTT
GATCACACCAGTACAGCTGAGATCACACAGCTAGGTGGAGCCGAGTCGCGTCGTAAGACTTGGCTC
GACAATCCACATGTTGGCAAGCAAGAATTGTCGT
TTTATTTTGCAGTGTACATGTAAAATTTGCGTGCTGCAGTTTTGGATTTATCATTGTGCAAATTAGGC
AGAATTACTGGTTAAAATGAGATATTTAGAAT
TAGCAAAATCGCCGGAAGTTTATCTATATATTAATCACACGCAATCACAGTTTATTTTTTAAAGGAA
CTGAAAGATAATTTATGTGGTGCTCAGTGATTC
AACCATCCAAATGATTAAGATTCATGGCATCTCTTCCTTTCTACATATTTCGATCTGCCTTTACTTGA
CAAGAGTCATCCATGTATCACATCACATGTAG
GGAACACTTACATATGCAGACATGTTGTCCTTGAGTTGCCCCTTCTTTCTCTCCCCCTTTGCTTGCTG
CTTTGCTTCTTAGTTGTTGCTCCTTCCTCACA
TGTAGTGTAGGGGAACACTTCTATACAGACTTTATTTTGCTTCACTTCTCCTTCGCATGCATGTTTGA
GTGGTTACTTCAAACAGTGTGGGCTGTATGTA
CATGCATTGGCCTCCCAAAGTCAGAGAAGCACGGCTACTTTCTCCCTAAGTATTGCAAGTTTGTACA
CCCCACGTACGAACGTACGTATGTACCAGAACA
GGCAGGTTGCAGTTGCAAGCAACTCTCTCTCCCTCTCTCAGTTCAAAAAAAAAAAAAAAAACTGCGT
GTGTGTGTGTGTGACTGTGTGTGTGTGTGTGTGT
GTGTGTGTGTGTGGCTGTGGTAGTAACAATGCATGTGTTGGACAGACAGCGTAAAGGCATTTCTCTC

AGACCATCTCTTTTACCTGCTCTTTCAGTGAGA
TCTCTGTTAGTATGCACGTGTGAAGAGTAGCAATGCATTCAAAGGGAAACAGTGGGATCACTTGCA
GTGTGACACACCATATGGAGACACTCCATATGAG
TCGGCTGCAAAGCTCCAAGCTGTCTGCGTGGGATCGATGGCAACAGTTTGGGATATATGCTTCTCAT
GTTCAGGATCATCATTGCCTAGCTACTGCATTG
GTCCCCCAGCCGGAATGTTCTTTCGGTCCAGAATTGATCTCTGATCATGCACTAATTTGTGTCTTACC
TAGGATTAGGATTTGCAGCCAGCTCATGCATG
CATGCTATGCTATGGATAGATTGATGATACACCATACACATATGTCCATACTTTTATGTGTATTGTG
AGTCACACATTAGTATTTCATGTGTATGCTGTC
TTTATATAGTTATGTGTTAATTTGTTCTAACCTGTTTTCATTTTTCGTTCAGATTCCATCTGCTTGATG
AGTTCGACGACGCGAAGAAGAGCTGCAGGAA
GCGCCTCGCAGACCACAACCGCCGTCGAAGGAAGTCAAAGCCGTCCGACGCCGATGCCGCTGATAA
AAGGAGGACACAGGCCAGCAAAGCAGCAGCAAGT
ACCAAAGGCAGTACGTGAAATGCATCCGTCCATGTTTCTCTACCTAATCATGCATTCTCTTGACACA
TATTCATCAATATTAACCTAGGTTTTTCCTTTA
ACTTACCAAGTAATTAACACCTCCAAAAACTAACAAAGTATTGTGAACAGAAGCAGCCGGAAGCAG
CAGCAAGAGCACCGGCACCGGAGACGGGATGGAC
ATTCAGGTGCAGCTGGGGAGCGGAGACCTGTCCAAAGATCAGGACGAAACCATGGGGCAGGGAGA
GGTGGTGAAAGAAATGCAGGTGGATCCCAAGGGGA
AAGCGTCAATGCAGCTGCAGCAGCAGCAACAGCAAGGACACCACGTCCATGGTCTTCACCTGCAGA
GCCAACATGGCTTCCCTTTCCCTTCGACCTCTGC
AGGCTCGTGCTTCCCTCAGAGCCAAGCTGTCTCGAGCACTGACAACACATCAAATATCGGCCAAGT
GCAGCAAGAACAGCTAGGCTTGGGGTTCCATCAG
CACAGCAACATCCTTCAGCTGGGACAGGCCATGTTTGATCTCGACTTCGATCACTAGCAAATTAGCT
AATATATAATTAGTAATATTCAATGCTCCCTCT
CGTACGTGTGTGTACGTCGATCGGATCATCTCTTTCTATCTTGTCTGTATTAATTACCGTAATATGAT
ATAATAATGGTAGTTTGTCCAGTGGCGATTTC
CATATATATTTGGTTAGAGGTGCTTCAATGCCCTCCCAAAAGGTGGTACTGGTACAATTCATACATT
TAGTACCTTTAATTTGCCTCATGTACTTGACAG
TAGTTATGCTCTTTGTACGTCACTGGCTCACAGTCACAGCTGATTAATGAGTTGGGGCATCAAAACG
TTCACAGTTTCATATATATGAG
>HORVU7Hr1G110950 genomic sequence
CCCGGATAGGCTGGGACGGATCCAATGACGATGAACTAGATCCACCATTGTTCCACCCATGCTGCG
GTGAGGAGGACAAACGACTGGTGAGTATGGGCAT
CGGCCCTGCGTTGATGGATCGCTTTTCCTCGAGCCCTGGCGTGTGTGTGTTNNNNNNNNNNNNNNN
NNNNNNNNNNNNNNNNNNNNNNNNNNNNNNNNN
NNNNNNNNNNNNNNNNNNNNNNNNNNNNNNNNNNNNNNNNNNNNNNNNNNNNNNNNNNNNNNN
NNNNNNNNNNNNNNNNNNNNNNNNNNNNNNGGGGGGGG
GGGTTGCTTGACCAATGCATCTGCACTGGGATAGCAGGGACGACGCCTGGCCGCGGGTCTTCAACC
GGGTCTGGCGGAGCACGAGGTGGAGGGATAGCTG
CCAATCGTCATGGGCCTCGAGGAGCCCCCAATCCACCTTGCCTGAGTCTAATCATAACAGGGGCAG
GAGACCTCAAGGACAGAGAGGAGCGAGAAGTGGG
AATGAGAGCTAGGTTAGGCTTTGTGGTGGATCGGGCGGAGCGAAATTTTGTGGTGTCTGAGTGGGG
CCAACCTGTCGGACATGTCCGGACAACCCTATAT
CCATCCCATATTNNNNNNNNNNNNNNNNNNNNNNNNNNNNNNNNNNNNNNNNNNNNNNNNNNNN
NNNNNNNNNNNNNNNNNNNNNNNNNNNNNNNNNNN
NNNNNNNNNNNNNTGCAATATATTGCAAATATTTTCACAACAACGTGTGGGGTATAACCTAGTTCGT
CAAGTTCCTAAATTTTCTTGCCAACCGTCGATTT
TCAGGGCCGTTGGATCATCATCCGACGGCCAAGATCACGCTACAATCTATTCATCATCTTCCTCCCG
CTCACGACGATTCCACCTCTGCCCCCGCGACCA
ATCCGACCCTAACCACCTACCTCTGCCTTCCCCGCGGTAGGGTGGCACTGCCACACACCACCTTGAC
ACCCTGACCCTCCCCTNGGCGGCGCCGGGGACG

CGGGGAGGATCGGTCTGAACCTCGGCCGGCGGACCTACTTCTCCCCGGGCGACATGATGGCCGTGG
ACCGGCTCCTGATGCGGTCCCGCCTGGGCGGCGT
GTTCGGGCTCGGGTTCGGCGGCGCCCACCACCAGCCGCCGCGCTGCCAGGCCGAGGGCTGCAAGGC
CGACCTCTCCGGCGCCAAGCACTACCACCGGCGC
CACAAGGTGTGCGAGTACCACGCCAAGGCGTCCCTCGTCGCCGCCAACGGCAAGCAGCAGCGCTTC
TGCCAGCAATGCAGCAGGTCAGAGCCAGACCCTC
CCTCTCTCTCTCTCTCTCCCTCCGTAGAAATGGAACAGTAGTTCTCGATCGTGAGGCGACCTTCTTGC
AGACGCCTAGTATAGCCAGAGCTAGGTTGCGG
CCAGGGCTAGGGTAGGGCCACCGGAAGCGGGCTGCCTTTTTCAGTTCGCGCTCTGACTTGGCTCATG
TCCATTGTCCGTTGTGGGCTGCCATGGAAAAAG
CTCTCCGGCCGGCGCGCACTGAGACGACCAGTCATGTCAACCAAAAAAGACAAGAGAAAACACTG
CTCCATGCCATGGCTTTTGCCCTGGCACGGTGGAG
TGCTCTACTAGGTCATGCACCTGCGCGCATCTGCACATGGCCTTCTCTTTCCTTGGTGGTTTTCCGGG
GGTCGACCGGAGAAGGGATCGTGGAAACGGAG
AGAATCAAAATGGGGAGGGGAAAAGGCTGCGTGCTTCGGGATGCCGCTGCAGAGTGCAGAAGACT
ATGGTGAGAGGAGGGAGACGATGGCTGATCCGGTG
CCAGGCTTTTGCCCTGACCATATCTCTGCGTCGGCCTTTTCTCTTTATCCTCCCATTGACCAATGTTGT
GGCCGCAGTGCTGCCAGTGGCGACTAGNNNN

Table S4. SBP domain sequences of barley, Arabidopsis, rice and wheat
>HORVU0Hr1G039170
SCAVDGCRADLSRCRDYHRRHKVCEAHSKTPVVTVAGREMRFCQQCSRFHLLTEFDEAKRSCRKRLDG
HNRRRRKPQPD
>HORVU7Hr1G051400
MCQVDDCRADLTSAKDYHRRHKVCEIHSKTTKAVVANQMQRFCQQCSRFHPLSEFDEGKRSCRRRLA
GHNRRRRKTQPT
>HORVU5Hr1G076380
SCAVDGCKADLSKCRDYHRRHKVCEAHSKTPLVVVAGREMRFCQQCSRFHMLAEFDEAKRSCRKRLD
GHNRRRRKPQVD
>HORVU6Hr1G031450
RCQVEGCGTELAAAKEYHRKHRVCEAHTKSPRVVVAGQERRFCQQCSRFHGLSEFDQKKRSCRRRLSD
HNARRRKPQPD
>HORVU7Hr1G042370
SCLVDGCHADLRDGRDYHRRHKVCEVHTKSTLVRIKNIEHRFCQQCSRFHLVQEFDEGKKSCRSRLEKH
NGRRRKAQAQ
>HORVU5Hr1G073440
RCQVEGCGVDLSGGKTYYCRHKVCSMHSKAPLVVVAGIEQRFCQQCSRFHQLPEFDQGKRSCRRRLAG
HNERRRKPPPG
>HORVU2Hr1G097610
RCQVEGCHMVLAGAKEYHRRHKVCEAHSKAPRVIVHGAEQRFCQQCSRFHAMAEFDDAKRSCRRRLA
GHNERRRKSNAN
>HORVU2Hr1G097580
RCQVEGCHMVLAGAKEYHRRHKVCEAHSKAPRVIVHGAEQRFCQQCSRFHAMAEFDDAKRSCRRRLA
GHNERRRKSNAN
>HORVU6Hr1G030490
RCQAEGCKTDLSAAKHYHRRHKVCEYHAKAATVAASGKQQRFCQQCSRYVVLAEFDEAKRSCRKRLT
EHNRRRRKPAGA
>HORVU6Hr1G019700
YCQVEGCKVDLSSVKEYHRKHRVCELHSKAPKVVVAGLERRFCQQCSRFHALSEFDQKKRSCRRRLND
HNSRRRKPQPE
>HORVU2Hr1G048280
XXXXERCGADLTEAKRYHRRHKVCEAHSKAAVVVVAGLRQRFCQQCSRFHELLEFDDQKRSCRRRLA
GHNERRRKSSAE
>HORVU3Hr1G094730
ACSVEGCTADLSRCREYHRRHKVCEAHSKTPVVAVAGQQQRFCQQCSRFHLLGEFDEVKRSCRKRLDG
HNRRRRKPQPD
>HORVU7Hr1G110980
RCQVEDCKADLSGAKHYHRRHKVCEYHAKAALVSTAGKQQRFCQQCSRSHVLMEFDEAKRSCRRRLA
EHNRRRRKPAAG
>HORVU5Hr1G117190
ACQVEGCCADLSAAKDYHRRHKVCEMHAKANTAVVGNTVQRFCQQCSRFHLLQEFDEGKRSCRRRLA
GHNKRRRKTRPE
>HORVU1Hr1G060770
RCQVPGCEADIRELKGYHKRHRVCLRCAHASAVMLDGVQKRYCQQCGKXXXXXXXXXXXXXXXXXX
XXXXXXXXXXXXXX
>HORVU0Hr1G039150
RCQAEGCKADLSGAKRYHRRHKVCEHHSKAPVVVTAGGLHQRFCQQCSRFHLLDEFDDAKKSCRKRL
ADHNRRRRKSKP
>HORVU7Hr1G110950
RCQAEGCKADLSGAKHYHRRHKVCEYHAKASLVAANGKQQRFCQQCSRSEPxxxxxxxxxxxxxxxxxxxx
xxxxxxx

>AtSPL1
VCQVENCEADLSKVKDYHRRHKVCEMHSKATSATVGGILQRFCQQCSRFHLLQEFDEGKRSCRRRLAG
HNKRRRKTNPE
>AtSPL2
HCQVEGCNLDLSSAKDYHRKHRICENHSKFPKVVVSGVERRFCQQCSRFHCLSEFDEKKRSCRRRLSDH
NARRRKPNPG
>AtSPL3
VCQVESCTADMSKAKQYHKRHKVCQFHAKAPHVRISGLHQRFCQQCSRFHALSEFDEAKRSCRRRLAG
HNERRRKSTTD
>AtSPL4
LCQVDRCTADMKEAKLYHRRHKVCEVHAKASSVFLSGLNQRFCQQCSRFHDLQEFDEAKRSCRRRLA
GHNERRRKSSGE
>AtSPL5
LCQVDRCTVNLTEAKQYYRRHRVCEVHAKASAATVAGVRQRFCQQCSRFHELPEFDEAKRSCRRRLAG
HNERRRKISGD
>AtSPL6
LCQVYGCSKDLSSSKDYHKRHRVCEAHSKTSVVIVNGLEQRFCQQCSRFHFLSEFDDGKRSCRRRLAGH
NERRRKPAFY
>AtSPL7
RCQVPDCEADISELKGYHKRHRVCLRCATASFVVLDGENKRYCQQCGKFHLLPDFDEGKRSCRRKLER
HNNRRKRKPVD
>AtSPL8
RCQAEGCNADLSHAKHYHRRHKVCEFHSKASTVVAAGLSQRFCQQCSRFHLLSEFDNGKRSCRKRLAD
HNRRRRKCHQS
>AtSPL9
RCQVEGCGMDLTNAKGYYSRHRVCGVHSKTPKVTVAGIEQRFCQQCSRFHQLPEFDLEKRSCRRRLAG
HNERRRKPQPA
>AtSPL10
RCQIDGCELDLSSSKDYHRKHRVCETHSKCPKVVVSGLERRFCQQCSRFHAVSEFDEKKRSCRKRLSHH
NARRRKPQGV
>AtSPL11
RCQIDGCELDLSSAKGYHRKHKVCEKHSKCPKVSVSGLERRFCQQCSRFHAVSEFDEKKRSCRKRLSHH
NARRRKPQGV
>AtSPL12
CCQVDNCGADLSKVKDYHRRHKVCEIHSKATTALVGGIMQRFCQQCSRFHVLEEFDEGKRSCRRRLAG
HNKRRRKANPD
>AtSPL13A
ICLVDGCDSDFSNCREYHKRHKVCDVHSKTPVVTINGHKQRFCQQCSRFHALEEFDEGKRSCRKRLDGH
NRRRRKPQPE
>AtSPL14
MCQVDNCTEDLSHAKDYHRRHKVCEVHSKATKALVGKQMQRFCQQCSRFHLLSEFDEGKRSCRRRLA
GHNRRRRKTTQP
>AtSPL15
RCQVEGCRMDLSNVKAYYSRHKVCCIHSKSSKVIVSGLHQRFCQQCSRFHQLSEFDLEKRSCRRRLACH
NERRRKPQPT
>AtSPL16
KCQVDNCKEDLSIAKDYHRRHKVCEVHSKATKALVGKQMQRFCQQCSRFHLLSEFDEGKRSCRRRLDG
HNRRRRKTQPD
>OsSPL1
CCQVDGCTVNLSSARDYNKRHKVCEVHTKSGVVRIKNVEHRFCQQCSRFHFLQEFDEGKKSCRSRLAQ
HNRRRRKVQVQ
>OsSPL2

ACSVEGCAADLSKCVRDYHRRHKVCEAHSKTAVVTVAGQQQRFCQQCSRFHLLGEFDEEKRSCRKRL
DGHNKRRRKPQPD
>OsSPL3
HCQVEGCNVDLSSAKPYHRKHRVCEPHSKTLKVIVAGLERRFCQQCSRFHGLAEFDQKKRSCRRRLHD
HNARRRKPQPE
>OsSPL4
RCQVEGCGVELVGVKDYHRKHRVCEAHSKFPRVVVAGQERRFCQQCSRFHALSEFDQKKRSCRRRLY
DHNARRRKPQTD
>OsSPL5
RCQAEGCKADLSAAKHYHRRHKVCDFHAKAAAVLAAGKQQRFCQQCSRFHVLAEFDEAKRSCRKRLT
EHNRRRRKPTAG
>OsSPL6
ACQVEGCTADLTGVRDYHRRHKVCEMHAKATTAVVGNTVQRFCQQCSRFHPLQEFDEGKRSCRRRLA
GHNRRRRKTRPE
>OsSPL7
RCQVEGCDITLQGVKEYHRRHKVCEVHAKAPRVVVHGTEQRFCQQCSRFHVLAEFDDAKKSCRRRLA
GHNERRRRSNAS
>OsSPL8
RCQAEGCKADLSSAKRYHRRHKVCEHHSKAPVVVTAGGLHQRFCQQCSRFHLLDEFDDAKKSCRKRL
ADHNRRRRKSKPS
>OsSPL9
KCQVPGCEADIRELKGYHRRHRVCLRCAHAAAVMLDGVQKRYCQQCGKFHILLDFDEDKRSCRRKLE
RHNKRRRRKPDS
>OsSPL10
RCQAEGCKADLSGAKHYHRRHKVCEYHAKASVVAASGKQQRFCQQCSRFHVLTEFDEAKRSCRKRLA
EHNRRRRKPAAA
>OsSPL11
RCQVEGCGLELGGYKEYYRKHRVCEPHTKCLRVVVAGQDRRFCQQCSRFHAPSEFDQEKRSCRRRLSD
HNARRRKPQTD
>OsSPL12
YCQVEGCKVDLSSAREYHRKHKVCEAHSKAPKVIVSGLERRFCQQCSRFHGLAEFDQKKKSCRRRLSD
HNARRRKPQQE
>OsSPL13
RCQVERCGVDLSEAGRYNRRHKVCQTHSKEPVVLVAGLRQRFCQQCSRFHELTEFDDAKRSCRRRLAG
HNERRRKSAAD
>OsSPL14
RCQVEGCGADLSGIKNYYCRHKVCFMHSKAPRVVVAGLEQRFCQQCSRFHLLPEFDQGKRSCRRRLAG
HNERRRRPQTP
>OsSPL15
MCQVDDCRADLTNAKDYHRRHKVCEIHGKTTKALVGNQMQRFCQQCSRFHPLSEFDEGKRSCRRRLA
GHNRRRRKTQPT
>OsSPL16
SCAVDGCKEDLSKCRDYHRRHKVCEAHSKTPLVVVSGREMRFCQQCSRFHLLQEFDEAKRSCRKRLDG
HNRRRRKPQPD
>OsSPL17
RCQVEGCGVDLSGVKPYYCRHKVCYMHAKEPIVVVAGLEQRFCQQCSRCSVHMVRFHQLPEFDQEKK
SCRRRLAGHNERRRKPTPG
>OsSPL18
SCAVDGCKADLSKHRDYHRRHKVCEPHSKTPVVVVSGREMRFCQQCSRFHLLGEFDEAKRSCRKRLDG
HNRRRRKPQAD
>TaSPL1
SCQVDGCHADLSDDRDYHRRHKVCEPHTKSTLVRIKNIEHRFCQQCSRFHLVQEFDEGKKSCRSRLATH

NRRRRKAPAE
>TaSPL3
YCQVEGCKVDLSSVKDYHRKHRVCELHSKAPKVVVAGLERRFCQQCSRFHALAEFDQKKRSCRRRLN
DHNSRRRKPQPE
>TaSPL6
ACQVEGCCADLSAAKDYHRRHKVCEMHAKANTAVVGNTVQRFCQQCSRFHLLQEFDEGKRSCRRRLA
GHNKRRRKTRPE
>TaSPL8
RCQAEGCKADLSGAKRYHRRHKVCEHHSKAPVVVTAGGLHQRFCQQCSRFHLLDEFDDAKKSCRKRL
ADHNRRRRKSKPS
>TaSPL15
MCQVDDCRADLTSAKDYHRRHKVCEIHSKTTKAVVGNQMQRFCQQCSRFHPLSEFDEGKRSCRRRLA
GHNRRRRKTQPT
>TaSPL17
RCQVEGCGVDLSGAKQYHSRHKVCSMHTKEPRVVVAGLEQRFCQQCSRFHQLPEFDQGKRSCRRRLA
GHNERRRKAPPG
>TaSPL20
RCQVXDCXADLSGAKHYHRRHKVCEYHAKAXLVSAAGKQQRFCQQCSRFHVLTEFDEAKRSCRRRLA
EHNRRRRKPAAG
>TaSPL21
RCQAEGCKADLSAAKHYHRRHKVCEYHAKAATVAASGKQQRFCQQCSRFHVLAEFDEAKRSCRKRLT
EHNRRRRKPAGA
>TaSPL22
RCQAEGCKADLSGAKHYHRRHKVCEYHAKASLVAAAGKQQRFCQQCSRFHVLTEFDEAKRSCRKRLA
EHNRRRRKPASS
>TaSPL23
PCSVEGCTADLSRCREYHRRHKVCEAHSKTPVVAVAGQQQRFCQQCSRFHLLGEFDEVKRSCRKRLDG
HNRRRRKPQPD

Table S5. Details of miR156 complementary site in *HvSPL* genes.

miRNA_Acc.	Target_Acc.	Expectation	UPE$	miRNA_start	miRNA_end	Target_start	Target_end	miRNA_aligned_fragment	Target_aligned_fragment	Inhibition	Multiplicity
hvu-miR156a	HORVU5Hr1G076380	1	-1	1	21	4216	4236	UGACAGAAGAGAGUGAGCACA	UGUGCUCUCUCUCUUCUGUCA	Cleavage	1
hvu-miR156a	HORVU3Hr1G094730.	1	-1	1	21	3542	3562	UGACAGAAGAGAGUGAGCACA	UGUGCUCUCUCUCUUCUGUCA	Cleavage	1
hvu-miR156a	HORVU0Hr1G039170.	1	-1	1	21	5953	5973	UGACAGAAGAGAGUGAGCACA	UGUGCUCUCUCUCUUCUGUCA	Cleavage	1
hvu-miR156a	HORVU5Hr1G073440.	1	-1	1	21	3463	3483	UGACAGAAGAGAGUGAGCACA	UGUGCUCUCUCUCUUCUGUCA	Cleavage	1
hvu-miR156a	HORVU6Hr1G031450.	1	-1	1	21	4119	4139	UGACAGAAGAGAGUGAGCACA	CGUGCUCUCUCUCUUCUGUCA	Cleavage	1
hvu-miR156a	HORVU6Hr1G019700.	1	-1	1	21	6811	6831	UGACAGAAGAGAGUGAGCACA	CAUGCUCUCUCUCUUCUGUCA	Cleavage	1
hvu-miR156a	HORVU2Hr1G048280.	1	-1	1	21	8382	8402	UGACAGAAGAGAGUGAGCACA	CAUGCUCCCUCUCUUCUGUCA	Cleavage	1
hvu-miR156b	HORVU5Hr1G076380	1	-1	1	21	4216	4236	UGACAGAAGAGAGUGAGCACA	UGUGCUCUCUCUCUUCUGUCA	Cleavage	1
hvu-miR156b	HORVU5Hr1G073440.	1	-1	1	21	3463	3483	UGACAGAAGAGAGUGAGCACA	UGUGCUCUCUCUCUUCUGUCA	Cleavage	1
hvu-miR156b	HORVU0Hr1G039170.	1	-1	1	21	5953	5973	UGACAGAAGAGAGUGAGCACA	UGUGCUCUCUCUCUUCUGUCA	Cleavage	1
hvu-miR156b	HORVU3Hr1G094730.	1	-1	1	21	3542	3562	UGACAGAAGAGAGUGAGCACA	UGUGCUCUCUCUCUUCUGUCA	Cleavage	1
hvu-miR156b	HORVU6Hr1G031450.	1	-1	1	21	4119	4139	UGACAGAAGAGAGUGAGCACA	CGUGCUCUCUCUCUUCUGUCA	Cleavage	1
hvu-miR156b	HORVU6Hr1G019700.	1	-1	1	21	6811	6831	UGACAGAAGAGAGUGAGCACA	CAUGCUCUCUCUCUUCUGUCA	Cleavage	1
hvu-miR156b	HORVU2Hr1G048280.	1	-1	1	21	8382	8402	UGACAGAAGAGAGUGAGCACA	CAUGCUCCCUCUCUUCUGUCA	Cleavage	1
hvu-miR5049c	HORVU6Hr1G019700.	2.5	-1	1	23	6478	6500	AGACAAUUAUUUUGGGACGGAGG	CCUCCGUCUCAAAAUAAGUGUCU	Cleavage	1
hvu-miR5049c	HORVU1Hr1G060770.	2.5	-1	1	23	9210	9232	AGACAAUUAUUUUGGGACGGAGG	CCUCCAUCCCAAAAUAAGUGUCU	Cleavage	1

Table S6. Details of splice variants produced from *HvSPL* genes.

**HORVU0Hr1G039170**	Chromosome chrUn: 248,141,188-248,147,114 reverse strand.			
Name	Transcript ID	bp	Protein	Biotype
Novel	HORVU0Hr1G039170.1	2625	290aa	Protein coding
Novel	HORVU0Hr1G039170.3	2507	227aa	Protein coding
Novel	HORVU0Hr1G039170.5	1845	394aa	Protein coding
Novel	HORVU0Hr1G039170.7	1631	52aa	Protein coding
Novel	HORVU0Hr1G039170.8	1456	58aa	Protein coding
Novel	HORVU0Hr1G039170.4	1369	212aa	Protein coding
Novel	HORVU0Hr1G039170.6	1249	400aa	Protein coding
Novel	HORVU0Hr1G039170.2	985	272aa	Protein coding
Novel	HORVU0Hr1G039170.9	365	120aa	Protein coding
**HORVU7Hr1G051400**	Chromosome chr7H: 192,879,289-192,885,430 reverse strand.			
Name	Transcript ID	bp	Protein	Biotype
Novel	HORVU7Hr1G051400.1	4960	732aa	Protein coding
Novel	HORVU7Hr1G051400.3	4884	1130aa	Protein coding
Novel	HORVU7Hr1G051400.2	4874	1130aa	Protein coding
Novel	HORVU7Hr1G051400.5	4723	612aa	Protein coding
Novel	HORVU7Hr1G051400.4	4624	612aa	Protein coding
Novel	HORVU7Hr1G051400.6	4444	613aa	Protein coding
Novel	HORVU7Hr1G051400.7	3837	508aa	Protein coding
Novel	HORVU7Hr1G051400.9	3372	609aa	Protein coding
Novel	HORVU7Hr1G051400.8	3321	1106aa	Protein coding
Novel	HORVU7Hr1G051400.10	1131	237aa	Protein coding
**HORVU5Hr1G076380**	Chromosome chr5H: 551,053,196-551,057,389 reverse strand.			
Name	Transcript ID	bp	Protein	Biotype
Novel	HORVU5Hr1G076380.2	3828	159aa	Protein coding
Novel	HORVU5Hr1G076380.1	1727	159aa	Protein coding
Novel	HORVU5Hr1G076380.3	1652	159aa	Protein coding
Novel	HORVU5Hr1G076380.6	1636	159aa	Protein coding
Novel	HORVU5Hr1G076380.9	1179	392aa	Protein coding
Novel	HORVU5Hr1G076380.8	1137	264aa	Protein coding
Novel	HORVU5Hr1G076380.4	1124	306aa	Protein coding
Novel	HORVU5Hr1G076380.7	1084	279aa	Protein coding
Novel	HORVU5Hr1G076380.11	1002	333aa	Protein coding
Novel	HORVU5Hr1G076380.10	996	331aa	Protein coding
Novel	HORVU5Hr1G076380.5	917	159aa	Protein coding
**HORVU6Hr1G031450**	Chromosome chr6H: 133,169,150-133,173,306 reverse strand.			
Name	Transcript ID	bp	Protein	Biotype

Novel	HORVU6Hr1G031450.3	2870	209aa	Protein coding
Novel	HORVU6Hr1G031450.7	2659	113aa	Protein coding
Novel	HORVU6Hr1G031450.10	2571	182aa	Protein coding
Novel	HORVU6Hr1G031450.4	2196	191aa	Protein coding
Novel	HORVU6Hr1G031450.1	1740	104aa	Protein coding
Novel	HORVU6Hr1G031450.9	1640	184aa	Protein coding
Novel	HORVU6Hr1G031450.2	1523	184aa	Protein coding
Novel	HORVU6Hr1G031450.6	1429	326aa	Protein coding
Novel	HORVU6Hr1G031450.5	1030	104aa	Protein coding
Novel	HORVU6Hr1G031450.8	1020	104aa	Protein coding
**HORVU7Hr1G042370**	Chromosome chr7H: 122,553,615-122,558,679 forward strand.			
Name	Transcript ID	bp	Protein	Biotype
Novel	HORVU7Hr1G042370.5	3086	328aa	Protein coding
Novel	HORVU7Hr1G042370.10	2775	245aa	Protein coding
Novel	HORVU7Hr1G042370.7	2685	541aa	Protein coding
Novel	HORVU7Hr1G042370.8	2526	841aa	Protein coding
Novel	HORVU7Hr1G042370.14	2251	328aa	Protein coding
Novel	HORVU7Hr1G042370.6	2247	389aa	Protein coding
Novel	HORVU7Hr1G042370.13	2104	328aa	Protein coding
Novel	HORVU7Hr1G042370.9	2008	512aa	Protein coding
Novel	HORVU7Hr1G042370.20	1988	328aa	Protein coding
Novel	HORVU7Hr1G042370.18	1957	328aa	Protein coding
Novel	HORVU7Hr1G042370.12	1929	328aa	Protein coding
Novel	HORVU7Hr1G042370.15	1915	328aa	Protein coding
Novel	HORVU7Hr1G042370.19	1825	328aa	Protein coding
Novel	HORVU7Hr1G042370.16	1781	500aa	Protein coding
Novel	HORVU7Hr1G042370.11	1747	328aa	Protein coding
Novel	HORVU7Hr1G042370.1	1701	221aa	Protein coding
Novel	HORVU7Hr1G042370.3	1443	306aa	Protein coding
Novel	HORVU7Hr1G042370.4	1417	221aa	Protein coding
Novel	HORVU7Hr1G042370.2	1302	221aa	Protein coding
Novel	HORVU7Hr1G042370.17	1081	189aa	Protein coding
**HORVU5Hr1G073440**	Chromosome chr5H: 539,014,598-539,018,210 forward strand.			
Name	Transcript ID	bp	Protein	Biotype
Novel	HORVU5Hr1G073440.12	3082	196aa	Protein coding
Novel	HORVU5Hr1G073440.13	2977	301aa	Protein coding
Novel	HORVU5Hr1G073440.4	2513	198aa	Protein coding
Novel	HORVU5Hr1G073440.7	1616	416aa	Protein coding
Novel	HORVU5Hr1G073440.6	1613	415aa	Protein coding
Novel	HORVU5Hr1G073440.14	1483	82aa	Protein coding
Novel	HORVU5Hr1G073440.10	1413	363aa	Protein coding

Novel	HORVU5Hr1G073440.9	1413	363aa	Protein coding
Novel	HORVU5Hr1G073440.11	1261	196aa	Protein coding
Novel	HORVU5Hr1G073440.8	1185	394aa	Protein coding
Novel	HORVU5Hr1G073440.5	1153	384aa	Protein coding
Novel	HORVU5Hr1G073440.1	647	215aa	Protein coding
Novel	HORVU5Hr1G073440.2	644	214aa	Protein coding
Novel	HORVU5Hr1G073440.3	636	212aa	Protein coding
**HORVU2Hr1G097610**	Chromosome chr2H: 679,689,970-679,692,277 forward strand.			
Name	Transcript ID	bp	Protein	Biotype
Novel	HORVU2Hr1G097610.1	2156	203aa	Protein coding
Novel	HORVU2Hr1G097610.2	559	125aa	Protein coding
**HORVU2Hr1G097580**	Chromosome chr2H: 679,640,052-679,642,809 forward strand.			
Name	Transcript ID	bp	Protein	Biotype
Novel	HORVU2Hr1G097580.1	2606	209aa	Protein coding
Novel	HORVU2Hr1G097580.2	552	122aa	Protein coding
**HORVU6Hr1G030490**	Chromosome chr6H: 127,986,525-127,990,179 forward strand.			
Name	Transcript ID	bp	Protein	Biotype
Novel	HORVU6Hr1G030490.3	1727	473aa	Protein coding
Novel	HORVU6Hr1G030490.2	1711	491aa	Protein coding
Novel	HORVU6Hr1G030490.1	884	294aa	Protein coding
Novel	HORVU6Hr1G030490.4	797	142aa	Protein coding
**HORVU6Hr1G019700**	Chromosome chr6H: 53,909,817-53,916,886 reverse strand.			
Name	Transcript ID	bp	Protein	Biotype
Novel	HORVU6Hr1G019700.1	7070	232aa	Protein coding
Novel	HORVU6Hr1G019700.2	4686	474aa	Protein coding
Novel	HORVU6Hr1G019700.17	4575	384aa	Protein coding
Novel	HORVU6Hr1G019700.10	4312	232aa	Protein coding
Novel	HORVU6Hr1G019700.4	3625	474aa	Protein coding
Novel	HORVU6Hr1G019700.16	3273	384aa	Protein coding
Novel	HORVU6Hr1G019700.11	3240	232aa	Protein coding
Novel	HORVU6Hr1G019700.3	3212	507aa	Protein coding
Novel	HORVU6Hr1G019700.9	3133	232aa	Protein coding
Novel	HORVU6Hr1G019700.6	3118	474aa	Protein coding
Novel	HORVU6Hr1G019700.13	2933	474aa	Protein coding
Novel	HORVU6Hr1G019700.18	2912	366aa	Protein coding
Novel	HORVU6Hr1G019700.12	2788	474aa	Protein coding
Novel	HORVU6Hr1G019700.5	2553	474aa	Protein coding
Novel	HORVU6Hr1G019700.15	2203	474aa	Protein coding
Novel	HORVU6Hr1G019700.7	2158	474aa	Protein coding
Novel	HORVU6Hr1G019700.14	1868	474aa	Protein coding
Novel	HORVU6Hr1G019700.8	1063	180aa	Protein coding

Novel	HORVU6Hr1G019700.19	949	289aa	Protein coding
Novel	HORVU6Hr1G019700.20	897	283aa	Protein coding
HORVU2Hr1G048280	Chromosome chr2H: 269,391,017-269,400,061 reverse strand.			
Name	Transcript ID	bp	Protein	Biotype
Novel	HORVU2Hr1G048280.1	1375	105aa	Protein coding
Novel	HORVU2Hr1G048280.2	588	195aa	Protein coding
Novel	HORVU2Hr1G048280.3	571	190aa	Protein coding
Novel	HORVU2Hr1G048280.4	235	78aa	Protein coding
**HORVU3Hr1G094730**	Chromosome chr3H: 647,368,957-647,372,642 reverse strand.			
Name	Transcript ID	bp	Protein	Biotype
Novel	HORVU3Hr1G094730.5	2272	227aa	Protein coding
Novel	HORVU3Hr1G094730.9	1733	328aa	Protein coding
Novel	HORVU3Hr1G094730.8	1663	256aa	Protein coding
Novel	HORVU3Hr1G094730.1	1630	429aa	Protein coding
Novel	HORVU3Hr1G094730.2	1630	429aa	Protein coding
Novel	HORVU3Hr1G094730.3	1610	426aa	Protein coding
Novel	HORVU3Hr1G094730.7	1582	357aa	Protein coding
Novel	HORVU3Hr1G094730.6	1477	227aa	Protein coding
Novel	HORVU3Hr1G094730.4	1344	295aa	Protein coding
Novel	HORVU3Hr1G094730.10	1307	345aa	Protein coding
Novel	HORVU3Hr1G094730.11	610	199aa	Protein coding
Novel	HORVU3Hr1G094730.12	356	118aa	Protein coding
**HORVU7Hr1G110980**	Chromosome chr7H: 631,945,657-631,949,862 forward strand.			
Name	Transcript ID	bp	Protein	Biotype
Novel	HORVU7Hr1G110980.2	1749	365aa	Protein coding
Novel	HORVU7Hr1G110980.5	1726	424aa	Protein coding
Novel	HORVU7Hr1G110980.4	1724	423aa	Protein coding
Novel	HORVU7Hr1G110980.3	1710	449aa	Protein coding
Novel	HORVU7Hr1G110980.7	1431	171aa	Protein coding
Novel	HORVU7Hr1G110980.6	1214	292aa	Protein coding
Novel	HORVU7Hr1G110980.1	904	237aa	Protein coding
**HORVU5Hr1G117190**				
	Chromosome chr5H: 650,559,269-650,565,702 reverse strand.			
Name	Transcript ID	bp	Protein	Biotype
Novel	HORVU5Hr1G117190.3	3817	473aa	Protein coding
Novel	HORVU5Hr1G117190.4	3774	473aa	Protein coding
Novel	HORVU5Hr1G117190.2	3723	498aa	Protein coding
Novel	HORVU5Hr1G117190.1	3661	473aa	Protein coding
Novel	HORVU5Hr1G117190.5	3584	964aa	Protein coding
Novel	HORVU5Hr1G117190.6	2333	442aa	Protein coding
**HORVU1Hr1G060770**	Chromosome chr1H: 441,051,831-441,063,137 forward strand.			

Name	Transcript ID	bp	Protein	Biotype
Novel	HORVU1Hr1G060770.2	2940	573aa	Protein coding
Novel	HORVU1Hr1G060770.3	2631	235aa	Protein coding
Novel	HORVU1Hr1G060770.4	2544	641aa	Protein coding
Novel	HORVU1Hr1G060770.6	2535	643aa	Protein coding
Novel	HORVU1Hr1G060770.5	2531	567aa	Protein coding
Novel	HORVU1Hr1G060770.10	2366	486aa	Protein coding
Novel	HORVU1Hr1G060770.8	2283	598aa	Protein coding
Novel	HORVU1Hr1G060770.9	1906	468aa	Protein coding
Novel	HORVU1Hr1G060770.1	1287	325aa	Protein coding
Novel	HORVU1Hr1G060770.7	880	83aa	Protein coding
Novel	HORVU1Hr1G060770.11	832	277aa	Protein coding
**HORVU0Hr1G039150**	chrUn:248118980..248122768 forward			
Name	Transcript ID	bp	Protein	Biotype
Novel	HORVU0Hr1G039150.3	1423	362	Protein coding
Novel	HORVU0Hr1G039150.2	1420	361	Protein coding
Novel	HORVU0Hr1G039150.4	1162	349	Protein coding
Novel	HORVU0Hr1G039150.5	994	120	Protein coding
Novel	HORVU0Hr1G039150.1	1152	383	Protein coding

Table S7. miR156 targeted *HvSPL* splice variants.

HORVU3Hr1G094730

miRNA_Acc.	Target_Acc.	Expectation	UPE$	miRNA_start	miRNA_end	Target_start	Target_end	miRNA_aligned_fragment	Target_aligned_fragment	Inhibition	Target_Desc.	Multiplicity
hvu-miR156a	HORVU3Hr1G094730.5	1	-1	1	21	1577	1597	UGACAGAAGAGAGUGAGCACA	UGUGCUCUCUCUCUUCUGUCA	Cleavage	cdna:protein_coding	1
hvu-miR156a	HORVU3Hr1G094730.2	1	-1	1	21	886	906	UGACAGAAGAGAGUGAGCACA	UGUGCUCUCUCUCUUCUGUCA	Cleavage	cdna:protein_coding	1
hvu-miR156a	HORVU3Hr1G094730.1	1	-1	1	21	886	906	UGACAGAAGAGAGUGAGCACA	UGUGCUCUCUCUCUUCUGUCA	Cleavage	cdna:protein_coding	1
hvu-miR156a	HORVU3Hr1G094730.8	1	-1	1	21	992	1012	UGACAGAAGAGAGUGAGCACA	UGUGCUCUCUCUCUUCUGUCA	Cleavage	cdna:protein_coding	1
hvu-miR156a	HORVU3Hr1G094730.6	1	-1	1	21	782	802	UGACAGAAGAGAGUGAGCACA	UGUGCUCUCUCUCUUCUGUCA	Cleavage	cdna:protein_coding	1
hvu-miR156a	HORVU3Hr1G094730.10	1	-1	1	21	636	656	UGACAGAAGAGAGUGAGCACA	UGUGCUCUCUCUCUUCUGUCA	Cleavage	cdna:protein_coding	1
hvu-miR156a	HORVU3Hr1G094730.7	1	-1	1	21	887	907	UGACAGAAGAGAGUGAGCACA	UGUGCUCUCUCUCUUCUGUCA	Cleavage	cdna:protein_coding	1
hvu-miR156a	HORVU3Hr1G094730.3	1	-1	1	21	878	898	UGACAGAAGAGAGUGAGCACA	UGUGCUCUCUCUCUUCUGUCA	Cleavage	cdna:protein_coding	1
hvu-miR156a	HORVU3Hr1G094730.9	1	-1	1	21	1062	1082	UGACAGAAGAGAGUGAGCACA	UGUGCUCUCUCUCUUCUGUCA	Cleavage	cdna:protein_coding	1
hvu-miR156a	HORVU3Hr1G094730.4	1	-1	1	21	629	649	UGACAGAAGAGAGUGAGCACA	UGUGCUCUCUCUCUUCUGUCA	Cleavage	cdna:protein_coding	1
hvu-miR156b	HORVU3Hr1G094730.3	1	-1	1	21	878	898	UGACAGAAGAGAGUGAGCACA	UGUGCUCUCUCUCUUCUGUCA	Cleavage	cdna:protein_coding	1
hvu-miR156b	HORVU3Hr1G094730.8	1	-1	1	21	992	1012	UGACAGAAGAGAGUGAGCACA	UGUGCUCUCUCUCUUCUGUCA	Cleavage	cdna:protein_coding	1
hvu-miR156b	HORVU3Hr1G094730.5	1	-1	1	21	1577	1597	UGACAGAAGAGAGUGAGCACA	UGUGCUCUCUCUCUUCUGUCA	Cleavage	cdna:protein_coding	1
hvu-miR156b	HORVU3Hr1G094730.9	1	-1	1	21	1062	1082	UGACAGAAGAGAGUGAGCACA	UGUGCUCUCUCUCUUCUGUCA	Cleavage	cdna:protein_coding	1
hvu-miR156b	HORVU3Hr1G094730.4	1	-1	1	21	629	649	UGACAGAAGAGAGUGAGCACA	UGUGCUCUCUCUCUUCUGUCA	Cleavage	cdna:protein_coding	1
hvu-miR156b	HORVU3Hr1G094730.1	1	-1	1	21	886	906	UGACAGAAGAGAGUGAGCACA	UGUGCUCUCUCUCUUCUGUCA	Cleavage	cdna:protein_coding	1
hvu-miR156b	HORVU3Hr1G094730.2	1	-1	1	21	886	906	UGACAGAAGAGAGUGAGCACA	UGUGCUCUCUCUCUUCUGUCA	Cleavage	cdna:protein_coding	1
hvu-miR156b	HORVU3Hr1G094730.7	1	-1	1	21	887	907	UGACAGAAGAGAGUGAGCACA	UGUGCUCUCUCUCUUCUGUCA	Cleavage	cdna:protein_coding	1
hvu-miR156b	HORVU3Hr1G094730.6	1	-1	1	21	782	802	UGACAGAAGAGAGUGAGCACA	UGUGCUCUCUCUCUUCUGUCA	Cleavage	cdna:protein_coding	1
hvu-miR156b	HORVU3Hr1G094730.10	1	-1	1	21	636	656	UGACAGAAGAGAGUGAGCACA	UGUGCUCUCUCUCUUCUGUCA	Cleavage	cdna:protein_coding	1

HORVU5Hr1G076380

Target_Acc.	Expectation	UPE$	miRNA_start	miRNA_end	Target_start	Target_end	miRNA_aligned_fragment	Target_aligned_fragment	Inhibition	Target_Desc.	Multiplicity
HORVU5Hr1G076380.5	1	-1	1	21	392	412	UGACAGAAGAGAGUGAGCACA	UGUGCUCUCUCUCUUCUGUCA	Cleavage	cdna:protein_coding	1
HORVU5Hr1G076380.8	1	-1	1	21	617	637	UGACAGAAGAGAGUGAGCACA	UGUGCUCUCUCUCUUCUGUCA	Cleavage	cdna:protein_coding	1
HORVU5Hr1G076380.6	1	-1	1	21	1111	1131	UGACAGAAGAGAGUGAGCACA	UGUGCUCUCUCUCUUCUGUCA	Cleavage	cdna:protein_coding	1
HORVU5Hr1G076380.1	1	-1	1	21	1149	1169	UGACAGAAGAGAGUGAGCACA	UGUGCUCUCUCUCUUCUGUCA	Cleavage	cdna:protein_coding	1
HORVU5Hr1G076380.10	1	-1	1	21	667	687	UGACAGAAGAGAGUGAGCACA	UGUGCUCUCUCUCUUCUGUCA	Cleavage	cdna:protein_coding	1
HORVU5Hr1G076380.9	1	-1	1	21	850	870	UGACAGAAGAGAGUGAGCACA	UGUGCUCUCUCUCUUCUGUCA	Cleavage	cdna:protein_coding	1
HORVU5Hr1G076380.7	1	-1	1	21	561	581	UGACAGAAGAGAGUGAGCACA	UGUGCUCUCUCUCUUCUGUCA	Cleavage	cdna:protein_coding	1
HORVU5Hr1G076380.11	1	-1	1	21	673	693	UGACAGAAGAGAGUGAGCACA	UGUGCUCUCUCUCUUCUGUCA	Cleavage	cdna:protein_coding	1
HORVU5Hr1G076380.3	1	-1	1	21	1121	1141	UGACAGAAGAGAGUGAGCACA	UGUGCUCUCUCUCUUCUGUCA	Cleavage	cdna:protein_coding	1
HORVU5Hr1G076380.2	1	-1	1	21	3250	3270	UGACAGAAGAGAGUGAGCACA	UGUGCUCUCUCUCUUCUGUCA	Cleavage	cdna:protein_coding	1
HORVU5Hr1G076380.4	1	-1	1	21	593	613	UGACAGAAGAGAGUGAGCACA	UGUGCUCUCUCUCUUCUGUCA	Cleavage	cdna:protein_coding	1
HORVU5Hr1G076380.4	1	-1	1	21	593	613	UGACAGAAGAGAGUGAGCACA	UGUGCUCUCUCUCUUCUGUCA	Cleavage	cdna:protein_coding	1
HORVU5Hr1G076380.8	1	-1	1	21	617	637	UGACAGAAGAGAGUGAGCACA	UGUGCUCUCUCUCUUCUGUCA	Cleavage	cdna:protein_coding	1
HORVU5Hr1G076380.5	1	-1	1	21	392	412	UGACAGAAGAGAGUGAGCACA	UGUGCUCUCUCUCUUCUGUCA	Cleavage	cdna:protein_coding	1
HORVU5Hr1G076380.10	1	-1	1	21	667	687	UGACAGAAGAGAGUGAGCACA	UGUGCUCUCUCUCUUCUGUCA	Cleavage	cdna:protein_coding	1
HORVU5Hr1G076380.7	1	-1	1	21	561	581	UGACAGAAGAGAGUGAGCACA	UGUGCUCUCUCUCUUCUGUCA	Cleavage	cdna:protein_coding	1
HORVU5Hr1G076380.9	1	-1	1	21	850	870	UGACAGAAGAGAGUGAGCACA	UGUGCUCUCUCUCUUCUGUCA	Cleavage	cdna:protein_coding	1
HORVU5Hr1G076380.11	1	-1	1	21	673	693	UGACAGAAGAGAGUGAGCACA	UGUGCUCUCUCUCUUCUGUCA	Cleavage	cdna:protein_coding	1
HORVU5Hr1G076380.6	1	-1	1	21	1111	1131	UGACAGAAGAGAGUGAGCACA	UGUGCUCUCUCUCUUCUGUCA	Cleavage	cdna:protein_coding	1
HORVU5Hr1G076380.3	1	-1	1	21	1121	1141	UGACAGAAGAGAGUGAGCACA	UGUGCUCUCUCUCUUCUGUCA	Cleavage	cdna:protein_coding	1
HORVU5Hr1G076380.1	1	-1	1	21	1149	1169	UGACAGAAGAGAGUGAGCACA	UGUGCUCUCUCUCUUCUGUCA	Cleavage	cdna:protein_coding	1
HORVU5Hr1G076380.2	1	-1	1	21	3250	3270	UGACAGAAGAGAGUGAGCACA	UGUGCUCUCUCUCUUCUGUCA	Cleavage	cdna:protein_coding	1

HORVU0Hr1G039170

Target_Acc.	Expectation	UPE$	miRNA_start	miRNA_end	Target_start	Target_end	miRNA_aligned_fragment	Target_aligned_fragment	Inhibition	Target_Desc.	Multiplicity
HORVU0Hr1G039170.6	1	-1	1	21	923	943	UGACAGAAGAGAGUGAGCACA	UGUGCUCUCUCUCUUCUGUCA	Cleavage	cdna:protein_coding	1
HORVU0Hr1G039170.3	1	-1	1	21	2041	2061	UGACAGAAGAGAGUGAGCACA	UGUGCUCUCUCUCUUCUGUCA	Cleavage	cdna:protein_coding	1
HORVU0Hr1G039170.2	1	-1	1	21	495	515	UGACAGAAGAGAGUGAGCACA	UGUGCUCUCUCUCUUCUGUCA	Cleavage	cdna:protein_coding	1
HORVU0Hr1G039170.1	1	-1	1	21	2051	2071	UGACAGAAGAGAGUGAGCACA	UGUGCUCUCUCUCUUCUGUCA	Cleavage	cdna:protein_coding	1
HORVU0Hr1G039170.4	1	-1	1	21	990	1010	UGACAGAAGAGAGUGAGCACA	UGUGCUCUCUCUCUUCUGUCA	Cleavage	cdna:protein_coding	1
HORVU0Hr1G039170.5	1	-1	1	21	1495	1515	UGACAGAAGAGAGUGAGCACA	UGUGCUCUCUCUCUUCUGUCA	Cleavage	cdna:protein_coding	1
HORVU0Hr1G039170.6	1	-1	1	21	923	943	UGACAGAAGAGAGUGAGCACA	UGUGCUCUCUCUCUUCUGUCA	Cleavage	cdna:protein_coding	1
HORVU0Hr1G039170.2	1	-1	1	21	495	515	UGACAGAAGAGAGUGAGCACA	UGUGCUCUCUCUCUUCUGUCA	Cleavage	cdna:protein_coding	1
HORVU0Hr1G039170.5	1	-1	1	21	1495	1515	UGACAGAAGAGAGUGAGCACA	UGUGCUCUCUCUCUUCUGUCA	Cleavage	cdna:protein_coding	1
HORVU0Hr1G039170.1	1	-1	1	21	2051	2071	UGACAGAAGAGAGUGAGCACA	UGUGCUCUCUCUCUUCUGUCA	Cleavage	cdna:protein_coding	1
HORVU0Hr1G039170.3	1	-1	1	21	2041	2061	UGACAGAAGAGAGUGAGCACA	UGUGCUCUCUCUCUUCUGUCA	Cleavage	cdna:protein_coding	1
HORVU0Hr1G039170.4	1	-1	1	21	990	1010	UGACAGAAGAGAGUGAGCACA	UGUGCUCUCUCUCUUCUGUCA	Cleavage	cdna:protein_coding	1

HORVU5Hr1G073440

miRNA_Acc.	Target_Acc.	Expectation	UPE$	miRNA_start	miRNA_end	Target_start	Target_end	miRNA_aligned_fragment	Target_aligned_fragment	Inhibition	Target_Desc.	Multiplicity
hvu-miR156a	HORVU5Hr1G073440.13	1	-1	1	21	2271	2291	UGACAGAAGAGAGUGAGCACA	UGUGCUCUCUCUCUUCUGUCA	Cleavage	cdna:protein_coding	1
hvu-miR156a	HORVU5Hr1G073440.12	1	-1	1	21	2393	2413	UGACAGAAGAGAGUGAGCACA	UGUGCUCUCUCUCUUCUGUCA	Cleavage	cdna:protein_coding	1
hvu-miR156a	HORVU5Hr1G073440.5	1	-1	1	21	818	838	UGACAGAAGAGAGUGAGCACA	UGUGCUCUCUCUCUUCUGUCA	Cleavage	cdna:protein_coding	1
hvu-miR156a	HORVU5Hr1G073440.8	1	-1	1	21	799	819	UGACAGAAGAGAGUGAGCACA	UGUGCUCUCUCUCUUCUGUCA	Cleavage	cdna:protein_coding	1
hvu-miR156a	HORVU5Hr1G073440.7	1	-1	1	21	866	886	UGACAGAAGAGAGUGAGCACA	UGUGCUCUCUCUCUUCUGUCA	Cleavage	cdna:protein_coding	1
hvu-miR156a	HORVU5Hr1G073440.10	1	-1	1	21	707	727	UGACAGAAGAGAGUGAGCACA	UGUGCUCUCUCUCUUCUGUCA	Cleavage	cdna:protein_coding	1
hvu-miR156a	HORVU5Hr1G073440.9	1	-1	1	21	707	727	UGACAGAAGAGAGUGAGCACA	UGUGCUCUCUCUCUUCUGUCA	Cleavage	cdna:protein_coding	1
hvu-miR156a	HORVU5Hr1G073440.6	1	-1	1	21	863	883	UGACAGAAGAGAGUGAGCACA	UGUGCUCUCUCUCUUCUGUCA	Cleavage	cdna:protein_coding	1
hvu-miR156a	HORVU5Hr1G073440.11	1	-1	1	21	673	693	UGACAGAAGAGAGUGAGCACA	UGUGCUCUCUCUCUUCUGUCA	Cleavage	cdna:protein_coding	1
hvu-miR156b	HORVU5Hr1G073440.10	1	-1	1	21	707	727	UGACAGAAGAGAGUGAGCACA	UGUGCUCUCUCUCUUCUGUCA	Cleavage	cdna:protein_coding	1
hvu-miR156b	HORVU5Hr1G073440.9	1	-1	1	21	707	727	UGACAGAAGAGAGUGAGCACA	UGUGCUCUCUCUCUUCUGUCA	Cleavage	cdna:protein_coding	1
hvu-miR156b	HORVU5Hr1G073440.11	1	-1	1	21	673	693	UGACAGAAGAGAGUGAGCACA	UGUGCUCUCUCUCUUCUGUCA	Cleavage	cdna:protein_coding	1
hvu-miR156b	HORVU5Hr1G073440.7	1	-1	1	21	866	886	UGACAGAAGAGAGUGAGCACA	UGUGCUCUCUCUCUUCUGUCA	Cleavage	cdna:protein_coding	1
hvu-miR156b	HORVU5Hr1G073440.13	1	-1	1	21	2271	2291	UGACAGAAGAGAGUGAGCACA	UGUGCUCUCUCUCUUCUGUCA	Cleavage	cdna:protein_coding	1
hvu-miR156b	HORVU5Hr1G073440.8	1	-1	1	21	799	819	UGACAGAAGAGAGUGAGCACA	UGUGCUCUCUCUCUUCUGUCA	Cleavage	cdna:protein_coding	1
hvu-miR156b	HORVU5Hr1G073440.12	1	-1	1	21	2393	2413	UGACAGAAGAGAGUGAGCACA	UGUGCUCUCUCUCUUCUGUCA	Cleavage	cdna:protein_coding	1
hvu-miR156b	HORVU5Hr1G073440.5	1	-1	1	21	818	838	UGACAGAAGAGAGUGAGCACA	UGUGCUCUCUCUCUUCUGUCA	Cleavage	cdna:protein_coding	1
hvu-miR156b	HORVU5Hr1G073440.6	1	-1	1	21	863	883	UGACAGAAGAGAGUGAGCACA	UGUGCUCUCUCUCUUCUGUCA	Cleavage	cdna:protein_coding	1

HORVU6Hr1G031450

miRNA_Acc.	Target_Acc.	Expectation	UPE$	miRNA_start	miRNA_end	Target_start	Target_end	miRNA_aligned_fragment	Target_aligned_fragment	Inhibition	Target_Desc.	Multiplicity
hvu-miR156a	HORVU6Hr1G031450.1	1	-1	1	21	1102	1122	UGACAGAAGAGAGUGAGCACA	CGUGCUCUCUCUCUUCUGUCA	Cleavage	cdna:protein_coding	1
hvu-miR156a	HORVU6Hr1G031450.8	1	-1	1	21	549	569	UGACAGAAGAGAGUGAGCACA	CGUGCUCUCUCUCUUCUGUCA	Cleavage	cdna:protein_coding	1
hvu-miR156a	HORVU6Hr1G031450.4	1	-1	1	21	1565	1585	UGACAGAAGAGAGUGAGCACA	CGUGCUCUCUCUCUUCUGUCA	Cleavage	cdna:protein_coding	1
hvu-miR156a	HORVU6Hr1G031450.7	1	-1	1	21	2111	2131	UGACAGAAGAGAGUGAGCACA	CGUGCUCUCUCUCUUCUGUCA	Cleavage	cdna:protein_coding	1
hvu-miR156a	HORVU6Hr1G031450.9	1	-1	1	21	1187	1207	UGACAGAAGAGAGUGAGCACA	CGUGCUCUCUCUCUUCUGUCA	Cleavage	cdna:protein_coding	1
hvu-miR156a	HORVU6Hr1G031450.6	1	-1	1	21	878	898	UGACAGAAGAGAGUGAGCACA	CGUGCUCUCUCUCUUCUGUCA	Cleavage	cdna:protein_coding	1
hvu-miR156a	HORVU6Hr1G031450.2	1	-1	1	21	885	905	UGACAGAAGAGAGUGAGCACA	CGUGCUCUCUCUCUUCUGUCA	Cleavage	cdna:protein_coding	1
hvu-miR156a	HORVU6Hr1G031450.5	1	-1	1	21	412	432	UGACAGAAGAGAGUGAGCACA	CGUGCUCUCUCUCUUCUGUCA	Cleavage	cdna:protein_coding	1
hvu-miR156a	HORVU6Hr1G031450.3	1	-1	1	21	2232	2252	UGACAGAAGAGAGUGAGCACA	CGUGCUCUCUCUCUUCUGUCA	Cleavage	cdna:protein_coding	1
hvu-miR156b	HORVU6Hr1G031450.3	1	-1	1	21	2232	2252	UGACAGAAGAGAGUGAGCACA	CGUGCUCUCUCUCUUCUGUCA	Cleavage	cdna:protein_coding	1
hvu-miR156b	HORVU6Hr1G031450.1	1	-1	1	21	1102	1122	UGACAGAAGAGAGUGAGCACA	CGUGCUCUCUCUCUUCUGUCA	Cleavage	cdna:protein_coding	1
hvu-miR156b	HORVU6Hr1G031450.4	1	-1	1	21	1565	1585	UGACAGAAGAGAGUGAGCACA	CGUGCUCUCUCUCUUCUGUCA	Cleavage	cdna:protein_coding	1
hvu-miR156b	HORVU6Hr1G031450.8	1	-1	1	21	549	569	UGACAGAAGAGAGUGAGCACA	CGUGCUCUCUCUCUUCUGUCA	Cleavage	cdna:protein_coding	1
hvu-miR156b	HORVU6Hr1G031450.7	1	-1	1	21	2111	2131	UGACAGAAGAGAGUGAGCACA	CGUGCUCUCUCUCUUCUGUCA	Cleavage	cdna:protein_coding	1
hvu-miR156b	HORVU6Hr1G031450.2	1	-1	1	21	885	905	UGACAGAAGAGAGUGAGCACA	CGUGCUCUCUCUCUUCUGUCA	Cleavage	cdna:protein_coding	1
hvu-miR156b	HORVU6Hr1G031450.5	1	-1	1	21	412	432	UGACAGAAGAGAGUGAGCACA	CGUGCUCUCUCUCUUCUGUCA	Cleavage	cdna:protein_coding	1
hvu-miR156b	HORVU6Hr1G031450.9	1	-1	1	21	1187	1207	UGACAGAAGAGAGUGAGCACA	CGUGCUCUCUCUCUUCUGUCA	Cleavage	cdna:protein_coding	1
hvu-miR156b	HORVU6Hr1G031450.6	1	-1	1	21	878	898	UGACAGAAGAGAGUGAGCACA	CGUGCUCUCUCUCUUCUGUCA	Cleavage	cdna:protein_coding	1

HORVU6Hr1G019700

miRNA_Acc.	Target_Acc.	Expectation	UPE$	miRNA_start	miRNA_end	Target_start	Target_end	miRNA_aligned_fragment	Target_aligned_fragment	Inhibition	Target_Desc.	Multiplicit
hvu-miR156a	HORVU6Hr1G019700.3	1	-1	1	21	2579	2599	UGACAGAAGAGAGUGAGCACA	CAUGCUCUCUCUCUUCUGUCA	Cleavage	cdna:protein_coding	1
hvu-miR156a	HORVU6Hr1G019700.12	1	-1	1	21	2289	2309	UGACAGAAGAGAGUGAGCACA	CAUGCUCUCUCUCUUCUGUCA	Cleavage	cdna:protein_coding	1
hvu-miR156a	HORVU6Hr1G019700.8	1	-1	1	21	516	536	UGACAGAAGAGAGUGAGCACA	CAUGCUCUCUCUCUUCUGUCA	Cleavage	cdna:protein_coding	1
hvu-miR156a	HORVU6Hr1G019700.6	1	-1	1	21	2538	2558	UGACAGAAGAGAGUGAGCACA	CAUGCUCUCUCUCUUCUGUCA	Cleavage	cdna:protein_coding	1
hvu-miR156a	HORVU6Hr1G019700.4	1	-1	1	21	3040	3060	UGACAGAAGAGAGUGAGCACA	CAUGCUCUCUCUCUUCUGUCA	Cleavage	cdna:protein_coding	1
hvu-miR156a	HORVU6Hr1G019700.10	1	-1	1	21	3806	3826	UGACAGAAGAGAGUGAGCACA	CAUGCUCUCUCUCUUCUGUCA	Cleavage	cdna:protein_coding	1
hvu-miR156a	HORVU6Hr1G019700.15	1	-1	1	21	1725	1745	UGACAGAAGAGAGUGAGCACA	CAUGCUCUCUCUCUUCUGUCA	Cleavage	cdna:protein_coding	1
hvu-miR156a	HORVU6Hr1G019700.2	1	-1	1	21	3827	3847	UGACAGAAGAGAGUGAGCACA	CAUGCUCUCUCUCUUCUGUCA	Cleavage	cdna:protein_coding	1
hvu-miR156a	HORVU6Hr1G019700.7	1	-1	1	21	1582	1602	UGACAGAAGAGAGUGAGCACA	CAUGCUCUCUCUCUUCUGUCA	Cleavage	cdna:protein_coding	1
hvu-miR156a	HORVU6Hr1G019700.14	1	-1	1	21	1385	1405	UGACAGAAGAGAGUGAGCACA	CAUGCUCUCUCUCUUCUGUCA	Cleavage	cdna:protein_coding	1
hvu-miR156a	HORVU6Hr1G019700.13	1	-1	1	21	2441	2461	UGACAGAAGAGAGUGAGCACA	CAUGCUCUCUCUCUUCUGUCA	Cleavage	cdna:protein_coding	1
hvu-miR156a	HORVU6Hr1G019700.5	1	-1	1	21	1968	1988	UGACAGAAGAGAGUGAGCACA	CAUGCUCUCUCUCUUCUGUCA	Cleavage	cdna:protein_coding	1
hvu-miR156a	HORVU6Hr1G019700.11	1	-1	1	21	2734	2754	UGACAGAAGAGAGUGAGCACA	CAUGCUCUCUCUCUUCUGUCA	Cleavage	cdna:protein_coding	1
hvu-miR156a	HORVU6Hr1G019700.9	1	-1	1	21	2598	2618	UGACAGAAGAGAGUGAGCACA	CAUGCUCUCUCUCUUCUGUCA	Cleavage	cdna:protein_coding	1
hvu-miR156a	HORVU6Hr1G019700.1	1	-1	1	21	6211	6231	UGACAGAAGAGAGUGAGCACA	CAUGCUCUCUCUCUUCUGUCA	Cleavage	cdna:protein_coding	1
hvu-miR156b	HORVU6Hr1G019700.12	1	-1	1	21	2289	2309	UGACAGAAGAGAGUGAGCACA	CAUGCUCUCUCUCUUCUGUCA	Cleavage	cdna:protein_coding	1
hvu-miR156b	HORVU6Hr1G019700.2	1	-1	1	21	3827	3847	UGACAGAAGAGAGUGAGCACA	CAUGCUCUCUCUCUUCUGUCA	Cleavage	cdna:protein_coding	1
hvu-miR156b	HORVU6Hr1G019700.5	1	-1	1	21	1968	1988	UGACAGAAGAGAGUGAGCACA	CAUGCUCUCUCUCUUCUGUCA	Cleavage	cdna:protein_coding	1
hvu-miR156b	HORVU6Hr1G019700.9	1	-1	1	21	2598	2618	UGACAGAAGAGAGUGAGCACA	CAUGCUCUCUCUCUUCUGUCA	Cleavage	cdna:protein_coding	1
hvu-miR156b	HORVU6Hr1G019700.1	1	-1	1	21	6211	6231	UGACAGAAGAGAGUGAGCACA	CAUGCUCUCUCUCUUCUGUCA	Cleavage	cdna:protein_coding	1
hvu-miR156b	HORVU6Hr1G019700.10	1	-1	1	21	3806	3826	UGACAGAAGAGAGUGAGCACA	CAUGCUCUCUCUCUUCUGUCA	Cleavage	cdna:protein_coding	1
hvu-miR156b	HORVU6Hr1G019700.7	1	-1	1	21	1582	1602	UGACAGAAGAGAGUGAGCACA	CAUGCUCUCUCUCUUCUGUCA	Cleavage	cdna:protein_coding	1
hvu-miR156b	HORVU6Hr1G019700.3	1	-1	1	21	2579	2599	UGACAGAAGAGAGUGAGCACA	CAUGCUCUCUCUCUUCUGUCA	Cleavage	cdna:protein_coding	1
hvu-miR156b	HORVU6Hr1G019700.4	1	-1	1	21	3040	3060	UGACAGAAGAGAGUGAGCACA	CAUGCUCUCUCUCUUCUGUCA	Cleavage	cdna:protein_coding	1
hvu-miR156b	HORVU6Hr1G019700.14	1	-1	1	21	1385	1405	UGACAGAAGAGAGUGAGCACA	CAUGCUCUCUCUCUUCUGUCA	Cleavage	cdna:protein_coding	1
hvu-miR156b	HORVU6Hr1G019700.15	1	-1	1	21	1725	1745	UGACAGAAGAGAGUGAGCACA	CAUGCUCUCUCUCUUCUGUCA	Cleavage	cdna:protein_coding	1
hvu-miR156b	HORVU6Hr1G019700.13	1	-1	1	21	2441	2461	UGACAGAAGAGAGUGAGCACA	CAUGCUCUCUCUCUUCUGUCA	Cleavage	cdna:protein_coding	1
hvu-miR156b	HORVU6Hr1G019700.11	1	-1	1	21	2734	2754	UGACAGAAGAGAGUGAGCACA	CAUGCUCUCUCUCUUCUGUCA	Cleavage	cdna:protein_coding	1
hvu-miR156b	HORVU6Hr1G019700.8	1	-1	1	21	516	536	UGACAGAAGAGAGUGAGCACA	CAUGCUCUCUCUCUUCUGUCA	Cleavage	cdna:protein_coding	1
hvu-miR156b	HORVU6Hr1G019700.6	1	-1	1	21	2538	2558	UGACAGAAGAGAGUGAGCACA	CAUGCUCUCUCUCUUCUGUCA	Cleavage	cdna:protein_coding	1
hvu-miR5049c	HORVU6Hr1G019700.1	2.5	-1	1	23	5878	5900	AGACAAUUAUUUUGGGACGGAGG	CCUCCGUCUCAAAAUAAGUGUCU	Cleavage	cdna:protein_coding	1

HORVU2Hr1G048280

miRNA_Acc.	Target_Acc.	Expectatio	UPE$	miRNA_start	miRNA_end	Target_start	Target_end	miRNA_aligned_fragment	Target_aligned_fragment	Inhibition	Target_Desc.	Multiplicity
hvu-miR156a	HORVU2Hr1G048280.1	1	-1	1	21	112	132	UGACAGAAGAGAGUGAGCACA	CAUGCUCCCUCUCUUCUGUCA	Cleavage	cdna:protein_coding	1
hvu-miR156b	HORVU2Hr1G048280.1	1	-1	1	21	112	132	UGACAGAAGAGAGUGAGCACA	CAUGCUCCCUCUCUUCUGUCA	Cleavage	cdna:protein_coding	1

Table S8. Primer sequences used in this study.

**Primer Names**	**Primer sequences**
HvSPL3_qFP	GATGTCTGTGGAGGGCAGAG
HvSPL3_qRP	CTTGCAGCCTTCAACCTGAC
HvSPL6_qFP	GAAGGAAAGCGAAGTTGTCG
HvSPL6_qRP	AAATTGGCGCATATTCCAAG
HvSPL13_qFP	GTGACCCGGGAACTAGTCAG
HvSPL13_qRP	GCCACAAATTATCCCAGCAG
HvSPL15_qFP	CGGTAGCAGGGGTATAAATGG
HvSPL15_qRP	TCAAAACGGGAACTGGAGAG
HvSPL23_qFP	AGTGCAGCAGGTTCCATTTG
HvSPL23_qRP	CTCCGTGGTGATTAGCGAAC
HvSPL11.1V_FP	CGCTTCGAAATTAGGTGGAG
HvSPL11.1V_RP	TGGATTCATAGCCACTGCTG
HvSPL11.2V_FP	TGCAAACCTGTGGTTATACAGC
HvSPL11.2V_RP	ATTGCCCACCTTTCCTCATC
HvSPL20.1V_FP	AGGAGCACCAGCACTTCATC
HvSPL20.1V_RP	TGCTAAAGCCCCAATACACC
HvSPL20.2V_FP	GCAAGAAAGAGGGTCTGTGC
HvSPL20.2V_RP	GCATGTGAATCCAGTGATGC
HvSPL8.1V_FP	CAGCCGGAATGTTCTTTCGGTC
HvSPL8.1V_RP	GGTGTATCATCAATCTATCCATAGC
HvSPL8.2V_FP	TACTTCCCCACCTTGGTCAG
HvSPL8.2V_RP	ACTGCTGGCAGAACCTCTGG
HvSPL8.3V_FP	CCGTCCATGTTTCTCTACC
HvSPL8.3V_RP	CCGGCTGCTTCTGTTCAC
HvActinF	GCCGTGCTTTCCCTCTATG
HvActinR	GCTTCTCCTTGATGTCCCTTA
HvGAPDHF	GTGAGGCTGGTGCTGATTA
HvGAPDHR	CGTGGTGCAGCTAGCATTTGAGAC
HvAP2_FP	AGGCCAAGTTCGACCAGAA
HvAP2_RP	CTGGGACTGAACTGCATGTG
